# Adaptive phenotype drives resistance to androgen deprivation therapy in prostate cancer

**DOI:** 10.1186/s12964-017-0206-x

**Published:** 2017-12-08

**Authors:** Nicoletta Ferrari, Ilaria Granata, Matteo Capaia, Marina Piccirillo, Mario Rosario Guarracino, Roberta Venè, Antonella Brizzolara, Andrea Petretto, Elvira Inglese, Martina Morini, Simonetta Astigiano, Adriana Agnese Amaro, Francesco Boccardo, Cecilia Balbi, Paola Barboro

**Affiliations:** 1 Molecular Oncology and Angiogenesis, Ospedale Policlinico San Martino, L.go R. Benzi 10, 16132 Genoa, Italy; 2 Institute for High Performance Computing and Networking (ICAR), National Research Council (CNR), Via Pietro Castellino 111, 80131 Naples, Italy; 3 Academic Unit of Medical Oncology, Ospedale Policlinico San Martino, L.go R. Benzi 10, 16132 Genoa, Italy; 4 Core Facilities-Proteomics Laboratory, Giannina Gaslini Institute, L.go G. Gaslini 5, 16147 Genoa, Italy; 5 Laboratory of Molecular Biology, Giannina Gaslini Institute, L.go G. Gaslini 5, 16147 Genoa, Italy; 6 Immunology, Ospedale Policlinico San Martino, L.go R. Benzi 10, 16132 Genoa, Italy; 7 Molecular Pathology, Ospedale Policlinico San Martino, L.go R. Benzi 10, 16132 Genoa, Italy; 8 Department of Internal Medicine and Medical Specialties, School of Medicine, University of Genova, L.go R. Benzi 10, 16132 Genoa, Italy

**Keywords:** Castration-resistant prostate cancer, Androgen deprivation therapy, Drug resistance, Stress response, Target therapy, Bioinformatic analysis

## Abstract

**Background:**

Prostate cancer (PCa), the second most common cancer affecting men worldwide, shows a broad spectrum of biological and clinical behaviour representing the epiphenomenon of an extreme heterogeneity. Androgen deprivation therapy is the mainstay of treatment for advanced forms but after few years the majority of patients progress to castration-resistant prostate cancer (CRPC), a lethal form that poses considerable therapeutic challenges.

**Methods:**

Western blotting, immunocytochemistry, invasion and reporter assays, and in vivo studies were performed to characterize androgen resistant sublines phenotype in comparison to the parental cell line LNCaP. RNA microarray, mass spectrometry, integrative transcriptomic and proteomic differential analysis coupled with GeneOntology and multivariate analyses were applied to identify deregulated genes and proteins involved in CRPC evolution.

**Results:**

Treating the androgen-responsive LNCaP cell line for over a year with 10 μM bicalutamide both in the presence and absence of 0.1 nM 5-α-dihydrotestosterone (DHT) we obtained two cell sublines, designated PDB and MDB respectively, presenting several analogies with CRPC. Molecular and functional analyses of PDB and MDB, compared to the parental cell line, showed that both resistant cell lines were PSA low/negative with comparable levels of nuclear androgen receptor devoid of activity due to altered phosphorylation; cell growth and survival were dependent on AKT and p38MAPK activation and PARP-1 overexpression; their malignant phenotype increased both in vitro and in vivo. Performing bioinformatic analyses we highlighted biological processes related to environmental and stress adaptation supporting cell survival and growth. We identified 15 proteins that could direct androgen-resistance acquisition. Eleven out of these 15 proteins were closely related to biological processes involved in PCa progression.

**Conclusions:**

Our models suggest that environmental factors and epigenetic modulation can activate processes of phenotypic adaptation driving drug-resistance. The identified key proteins of these adaptive phenotypes could be eligible targets for innovative therapies as well as molecules of prognostic and predictive value.

**Electronic supplementary material:**

The online version of this article (10.1186/s12964-017-0206-x) contains supplementary material, which is available to authorized users.

## Background

The androgen receptor (AR) plays a crucial role in normal prostate cell growth and in almost all forms of PCa [1]. Androgen deprivation therapy (ADT) is the mainstay of treatment for advanced PCa, resulting in a significant clinical regression and improvement of quality of life in more than 80% of cases. Unfortunately, after a few years the majority of patients progress to CRPC, a lethal form with a median survival of 16–18 months for which no therapies are available.

To date, the molecular mechanisms by which hormone-sensitive PCa cells acquire the ability to resist to hormone deprivation remain largely unknown, thus preventing the development of effective therapies. AR reactivation has been shown to occur in many CRPC cell populations and, at the same time, AR-independent signaling pathways may be activated evading canonical cell growth control strategies [2–4]. During PCa evolution and progression, cell heterogeneity is generated by genomic rearrangements and rare mutations converging on specific biological processes and pathways [5, 6]. Emerging evidence has also suggested the role of phenotypic plasticity in survival and proliferation in response to therapies [7, 8]. Thus, drug-induced resistance may depend both on genetic mechanisms, mainly generated by genomic instability, and non-genetic cell state dynamics switching cell phenotype between multiple stable or metastable cell states, depending on environmental factors [9]. As a result, in the same patient various clonal PCa cell populations with different phenotypes, evolution and drug susceptibility can coexist [10].

PCa cells with low or null PSA expression have an important role in the progression to CRPC: their increase correlated with patients’ shorter survival [10] and ADT led to their expansion [11, 12]. Accordingly, among patients with high grade disease, low PSA plasmatic levels correlated with higher risk for cancer-specific death [13] and lower sensitivity to chemotherapy treatments [10, 12].

In our study, to simulate the clinical condition of PCa patients receiving medical or surgical castration combined with ADT, we generated two androgen-resistant LNCaP sublines by treating the cells for a prolonged time with bicalutamide (BIC) in presence (PDB) and absence (MDB) of 0.1 nM 5-α-dihydrosterone. Molecular and functional analyses performed on the two BIC-resistant sublines showed that these in vitro models recapitulated some phenotypic features of PCa evolution to CRPC. Integrative and comparative transcriptome and proteome analyses of the two sublines with parental cells, allowed to clarify the molecular alterations supporting CRPC evolution. Defining the molecular landscape of the various tumor cell populations with different drug sensitivities could allow the identification of novel therapeutic combinations for successful treatment outcomes.

## Methods

### Cell culture

The androgen-dependent human prostate cancer cell line LNCaP was from the American Type Culture Collection (ATCC) and cultured as described [14]. The passage numbers at which LNCaP cells were utilized ranged from 22 to 30. PDB and MDB subclones were obtained as described in Additional file 1: Figure S1.

Cell proliferation was assessed using the crystal violet assay. The data are presented as percentages of the absorbance value read at time 0, for cell proliferation curves, or as percentages of the absorbance of treated cells with respect to cell treated with DMSO, for dose-response curves. At least two independent experiments, each performed 10 times, were done. Two-tailed Student’s *t*-test was used to calculate the *p*-values and were considered statistically significant when *p < 0.05, **p < 0.01 and *** *p* < 0.001.

A commercially available kit (Cell Death Detection, Roche) was employed to measure enrichment of cytoplasmic histone-associated DNA fragments, indicative of apoptosis, using 30,000 cells per well seeded in 24-well plates.

### Western blot (WB) analysis and immunocytochemistry

Cell lysates were prepared and quantified as reported [15]. Equal amounts of protein extract (8 μg) were resolved by SDS-PAGE or by phosphate-affinity-PAGE (Mn^2+^-Phos-tag gel electrophoresis) [16], transferred to a Hybond-P membrane (GE Healthcare) and probed at 4 °C overnight with the antibodies reported in Additional file 1: Table S1. The relative amounts of immunoreactive bands, revealed by enhanced chemiluminescence (Immobilon, Millipore), were obtained as previously described [15] by normalizing the integrated optical density to the total density of the corresponding Sypro Ruby stained gel. Statistical significance was evaluated by the two-tailed Student’s *t*-test.

Immunocytochemistry was performed as described previously [17] using mouse anti-AR (DAKO, diluted 1: 100) or rabbit anti-PSA (Cell Signaling Technology, diluted 1:200). Cells were observed by light microscope and photographed under 20× or 40× magnification.

### Invasion assay

Cell invasion assays were carried out in cell-Matrigel chambers (BD Bio Coat) following manufacturer’s instructions.

### Reporter assay

Cells were transfected with Lipofectamine 2000 (Thermo) utilizing the Cignal androgen receptor dual-luciferase reporter-kit (Qiagen) following the manufacturer's instructions. DHT (10 nM) was added 15 h after transfection and luciferase activity was assayed in triplicates 48 h after transfection using the Dual-Luciferase reporter assay kit (Promega). To control for general effects on transcription, renilla luciferase was cotransfected in all reporter assays and luciferase values represent ratios of luciferase/renilla.

### In vivo studies

Six to eight-week-old NOD/SCID male mice were obtained from the breeding program of the Animal Care Facility and inoculated subcute with 5 × 10^6^ LNCaP, MDB or PBD cells, in a volume of 200 μl containing Matrigel (BD), and in the presence/absence of DHT and BIC. Twelve animals were inoculated for each cell line. To follow tumor growth all animals were regularly palpated, and all nodules were measured twice weekly with a caliper. Volumes were calculated by the formula *d*_*1*_ *xd*_*2*_ *xd*_*3*_ */2* were *d* represents the diameter. All animals were sacrificed when the nodule reached a volume of 400 mm^3^.

### ROS detection

Intracellular ROS were quantified by fluorimetry as described [18]. Briefly, cells were incubated for 30 min at 37 °C with 10 μM of H_2_DCFDA (Thermo Fisher Scientific) and fluorescence was measured in cell lysates by spectrofluorimetry (ex 480 nm, em 530 nm). Protein content from each sample was used to normalize fluorescence intensity. Data are expressed as relative fluorescence units (RFU) per μg proteins.

### RNA microarray

Total RNA was extracted using the miRNeasy Mini kit (Qiagen), according to manufacturer’s protocol. Gene expression profiles were obtained with the Affymetrix Human Genome-U133+ PM Array Strip run on the Gene Atlas System (Affymetrix - Thermo Fisher Scientific). Total RNA was prepared for the hybridization with the GeneChip 3′ IVT PLUS Reagent Kit according to the protocol provided by Affymetrix as previously described [19].

### Mass spectrometry (MS) analysis

Sample preparations for proteomic analysis were carried out as described [20]. All MS experiments were performed on a nanoscale high-performance liquid chromatography system connected to a hybrid linear trap quadrupole (LTQ) Orbitrap mass spectrometer. The MS instrument was operated in data-dependent mode to automatically switch between full-scan MS and MS/MS acquisition. Survey full-scan MS spectra were acquired in the Orbitrap analyzer with resolution *R* = 60,000. The 20 most intense peptide ions with charge states ≥2 were sequentially isolated and fragmented by collision-induced dissociation in the LTQ mass spectrometer. Raw mass spectrometric data were analyzed using the MaxQuant pipeline [21].

### Differential expression analysis

Microarray data pre-processing and differential expression analysis of both genes and proteins were performed using the Bioconductor Affy and Limma packages respectively [22, 23]. Microarray probe level data were corrected, quantile and Robust Multi-array Average normalized and converted into expression values. Pairwise comparisons between cell line groups were performed through the generation of three contrast matrices (PDBvsLNCaP, MDBvsLNCaP, MDBvsPDB). Differentially expressed genes and proteins having a |log_2_FC| ≥ 1 and the corresponding BH-adj. *p*-value ≤0.05 were considered significant.

### Hierarchical clustering

Hierarchical biclustering of samples was performed for both expressed genes and proteins using MATLAB’s *Clustergram* algorithm [24, 25].

### Principal component analysis (PCA)

PCA provided an overview of the variation in data and detected genes and/or proteins responsible for differences among the three cell lines. To this extent, the *prcomp* function of the *stats* R package [26] was used. Plots were made using the RGL library for R [27].

### Enrichment and generation of functional networks

The profiles of differentially expressed genes and proteins, in each group comparison, were represented as network and charts of over-represented Gene Ontology (GO) terms. Cytoscape plugin ClueGO 2.3.3 [28] was used to define interrelations among functional groups. The human GO Biological Processes were used with the following settings: Type of analysis: single; GO terms level: 3–10; GO term restriction: 3 genes and 4%; Evidence code: all. A significance threshold level of BH adjusted *p*-value ≤0.05 was applied. DEGs and DEPs were further intersected with list of genes belonging to pathways known to be affected in cancer. The lists were downloaded from KEGG pathway database [29].

## Results

### Characterization of PDB and MDB cell sublines

**Fig. 1:**
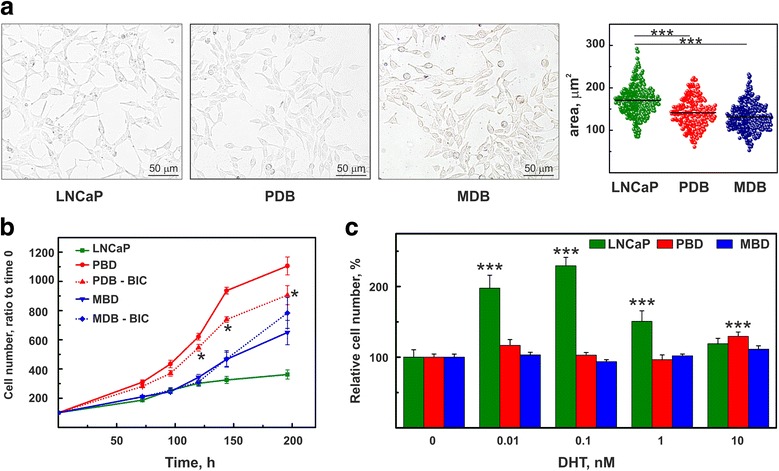
Characterization of PDB and MDB cell sublines. **a** Using phase contrast microscopy PDB and MDB cells show a morphology different from parental LNCaP cells: they are more rounded with a significantly smaller area. Horizontal lines indicate the mean values obtained by measuring at least 250 cells (right panel). **b** Cell Proliferation curves in the absence or presence of 10 μM BIC (PDB and MDB) compared to LNCaP parental cells. **c** Dose-response for DHT in LNCaP, PDB and MDB. The cells were treated for 3 days with the indicated concentration of DHT

The CRPC in vitro models currently available (i.e. PC3, DU145) marginally mimic the clinical condition observed in CRPC patients, as they do not express AR and survive in androgen-depleted medium. To study the evolution from androgen-responsive to androgen-independent PCa, we generated two BIC-resistant cell sublines treating the androgen-sensitive LNCaP cells with 10 μM BIC, the most commonly prescribed antiandrogen in advanced PCa. The PDB cell line, obtained by adding 0.1 nM DHT to cell medium, represents a model where androgens still exert their activity in the tumor microenvironment despite castrate levels of circulating androgens. The MDB cell line, instead, obtained by maintaining the cells in androgen-depleted medium, simulates the condition of maximal androgen blockade. The PDB and MDB cells showed a morphology different from that of the parental LNCaP line; they were rounded, significantly smaller (Fig. 1a) and produced a monolayer. They did not exhibit neuroendocrine features and, indeed, were negative for the neuroendocrine cell markers synaptophysin and neuron-specific enolase (Additional file 1: Figure S2).

Genotyping of the complete Short Tandem Repeat (STR) profile, carried out on total DNA by the Biological Bank of the IRCCS-AOU-San Martino-IST, ensured cell identity and excluded genomic variation caused by long-term exposure to anti-androgen. LNCaP profiles were in agreement with results published by international biobanks and the match between LNCaP and BIC-resistant cell sublines alleles was 88% for PDB and 94% for MDB (Additional file 1: Table S2).

When compared to naïve LNCaP cells, both BIC-resistant sublines exhibited a faster growth rate (Fig. 1b). However, while the growth of PDB cells was significantly inhibited by the absence of BIC, suggesting that the drug exerted an agonistic activity [30], the growth curve of MDB cells was not affected by BIC removal (Fig. 1b). When LNCaP, PDB and MDB cells were exposed to different concentrations of DHT (Fig. 1c), LNCaP cells showed a biphasic response with a maximum stimulation at 0.1 nM DHT. MDB growth was DHT-concentration independent, while PDB retained a weak androgen-dependence at high dosages.

**Fig. 2:**
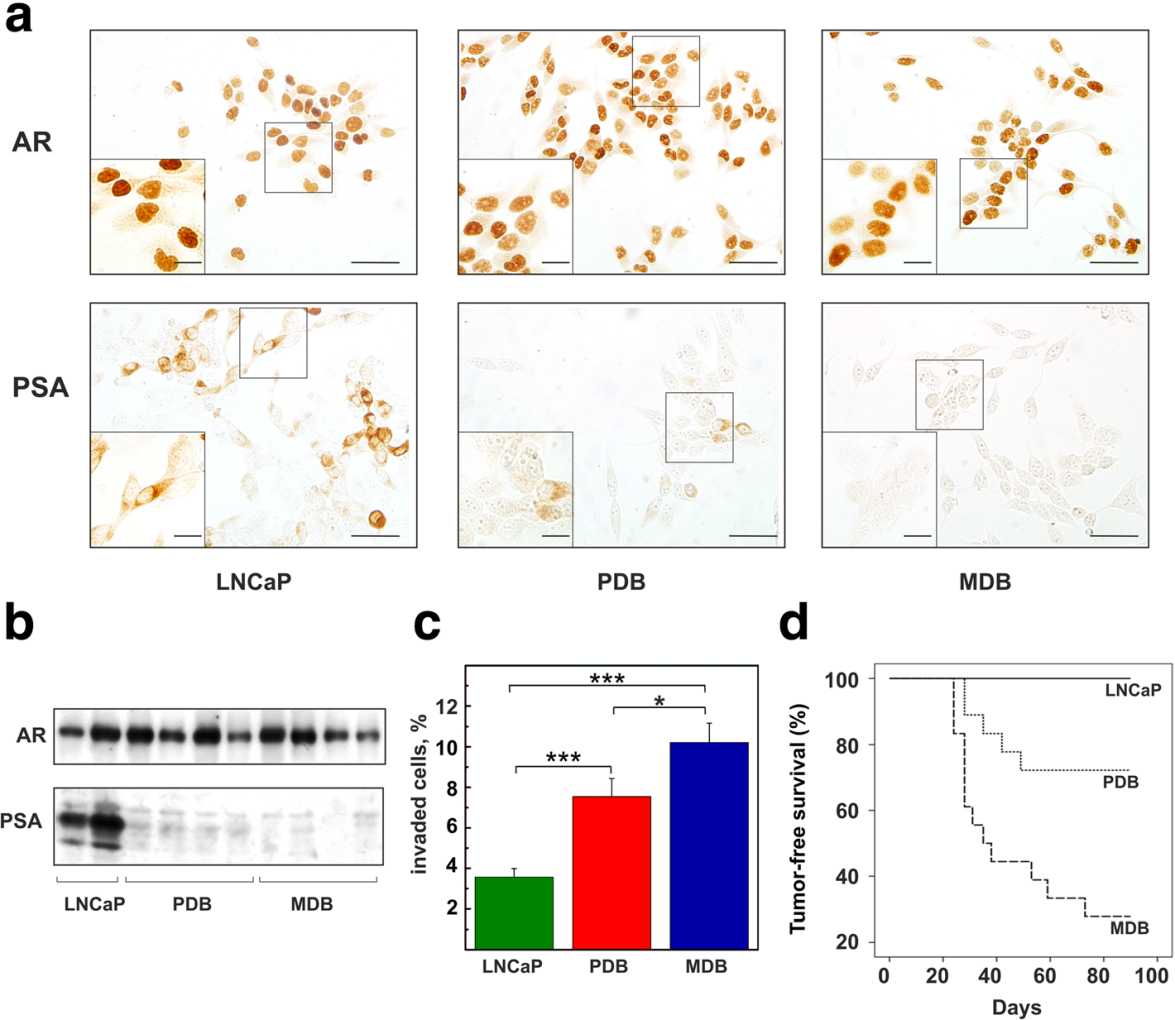
Comparison of AR and PSA expression and aggressiveness in LNCaP, PDB and MDB. **a** Immunolocalization of AR (top) and PSA (bottom) in the three cell lines. The scale bars correspond to 100 μm and 20 μm (inset). **b** WB analysis of AR and PSA gene expression in the three cell lines. Samples were probed with antibodies against AR and PSA. **c** Evaluation of the capability of the three cell lines to invade Matrigel in response to human fibroblast-conditioned medium (*p < 0.05; *** *p* < 0.001). **d** In vivo experiments to evaluate tumor growth in NOD SCID male mice by LNCaP, PDB and MDB cells. Twelve animals were inoculated subcute with each cell line

Immunocytochemistry of LNCaP, PDB and MDB cells revealed that AR was mainly localized in the nucleus (Fig. 2a, upper panel), indicating that the AR lack of function in MDB and PDB cells did not depend on its altered cellular localization. PSA (Fig. 2a, lower panel) was heterogeneously distributed in LNCaP and almost absent in both PDB and MDB cell lines. WB analysis confirmed that PSA was weakly expressed in PDB and MDB while AR remained constant in LNCaP, MDB and PDB cells (Fig. 2b). These results are in agreement with those reported for patients with poorly differentiated PCa (Gleason score 7) where PSA negative foci appear to be rare but become more abundant in advanced and recurrent PCa (Gleason score 9–10) [11].

As invasiveness is a key factor of cancer progression, we assessed the capability of the three cell lines to invade Matrigel in response to human fibroblast-conditioned medium. The results (Fig. 2c) show that PDB and MDB have an enhanced invasive activity reflecting a major aggressiveness compared to LNCaP cells. The acquisition of an aggressive phenotype by PBD and MDB cells was further confirmed by in vivo experiments (Fig. 2d). LNCaP cells were unable to induce tumors when inoculated subcutaneously in NOD/SCID male mice compared to the 100% tumor growth by MDB cells. PDB cells displayed an intermediate phenotype and produced tumors in 30% of animals. Interestingly, this aggressiveness was neither dependent on EMT nor on stem-like phenotypes (Additional file 1: Figure S2).

### The role of AR phosphorylation in CRPC insurgence

**Fig. 3:**
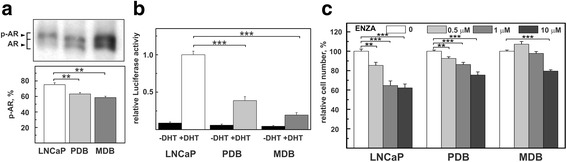
Characterization of phosphorylation and transcriptional activity of AR in LNCaP, PDB and MDB. **a** The overall degree of AR phosphorylation was evaluated for total proteins extracted from LNCaP, PDB and MDB by Phos-tag SDS-PAGE technique and immunoblotted for AR (upper panel). The relative amounts of phosphorylated AR (p-AR) were obtained by normalizing the integrated optical density by the relative amounts of immunoreactive AR bands (p-AR + AR) for each sample (lower panel). The bars represent the mean ± SE of three experiments. **b** AR transcriptional activity determined by the luciferase activity assay. **c** Cell viability of LNCaP, PDB and MDB cells incubated for 120 h with different concentrations of enzalutamide (ENZA)

Castration levels of hormone modulate AR activity through its phosphorylation at multiple sites [4, 31]. As the analysis of each phosphorylated residue can give discordant results depending on cellular context or tumor microenvironment, we evaluated the total AR phosphorylation status using the Phos-tag SDS-PAGE technique. In LNCaP cells over 73% of AR resulted phosphorylated while in the resistant cell lines PDB and MDB it significantly decreased to 66 and 60% respectively (Fig. 3a).

Since the AR transcriptional activity is positively regulated by its phosphorylation status [31], we determined AR activity by transfecting LNCaP, PDB and MDB cells with a plasmid expressing the luciferase reporter gene driven by a promoter containing an androgen response element (ARE). In the presence of 10 nM DHT, LNCaP cells increased AR activity approximately 11-fold, while PDB and MDB exhibited activity levels reduced by 61 and 80%, respectively, compared to LNCaP (Fig. 3b). Overall, our data show that, despite comparable expression levels of nuclear AR, decreased or absent AR function in PDB and MDB sublines correlated with an altered phosphorylation status.

To verify the AR contribution to the survival and growth of the two resistant cell lines, we tested enzalutamide, a new nonsteroidal antiandrogen approved as second-line option for CRPC therapy. This potent anti-AR dose-dependently affected LNCaP and PDB cell growth, while MDB cells appeared more resistant (Fig. 3c).

### Non-androgen signaling pathways regulate BIC-resistant cell growth

**Fig. 4:**
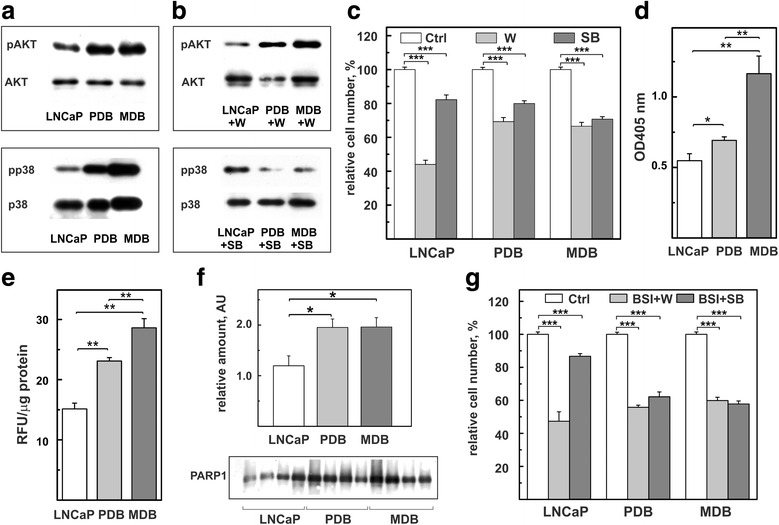
Prolonged BIC exposure activates non-androgen signaling pathways and enhances apoptosis, ROS levels and PARP-1 expression. Representative WB analysis of total proteins extracted from LNCaP, PDB and MDB untreated (**a**) or treated for 72 h (**b**) with 1 μM wortmannin (W, top panel) or 1 μM SB203580 (SB, bottom panel) and probed with antibodies against AKT, ppAKT, p38MAPK and pp38MAPK. One experiment, out of two with similar results, is reported. **c** LNCaP, PDB and MDB were treated with 1 μM W or 1 μM SB for 120 h. **d** Basal levels of apoptosis measured as cytoplasmic histone-associated DNA fragments. Values are representative of three independent experiments conducted in triplicate. Mean ± SE are reported. **e** Intracellular ROS quantified by fluorimetry as reported in materials and methods. Values are representative of three independent experiments conducted in triplicate. Mean ± SE are reported. **f** WB analysis of PARP-1 expression in four different preparations. Histogram represents the mean ± SE of the relative amounts of the proteins determined by quantitative analysis of WB. **g** Viability of LNCaP, PDB and MDB cells co-treated for 120 h with 10 μM BSI-201 in the presence of 1 μM W or 1 μM SB. *p < 0.05, **p < 0.01 and *** *p* < 0.001

Studies on CRPC tissues showed that genomic and transcriptomic changes also involved non-androgen pathways of which the most important are PI3K/AKT, RAF/MAPK/ERK and DNA repair [2, 32, 33]. LNCaP, PDB and MDB cell lysates were assessed for total and phosphorylated AKT and p38MAPK. As shown in Fig. 4a, total expression levels were similar in the three cell lines while AKT and p38MAPK activities (pAKT and pp38MAPK respectively) increased in both androgen-resistant sublines. ERK expression levels were similar in the three cell lines while pERK was almost undetectable (Additional file 1: Figure S3). When we examined the effects of the pAKT inhibitor Wortmannin (W) and pp38MAPK inhibitor SB203580 (SB) (Fig. 4b), we noticed that 72 h treatment with W reduced pAKT more efficiently in LNCaP than in PDB and MDB resistant cells. Conversely, SB for 72 h blocked p38MAPK phosphorylation in PDB and MDB cells but was inactive in LNCaP cells. As a confirmation, W efficiently reduced LNCaP cell growth, while PDB and MDB cells were equally susceptible to both treatments (Fig. 4c).

The stress protein kinase p38MAPK is involved in apoptosis regulation [34]. Indeed, the finding that the p38MAPK signaling pathway is more active in BIC-resistant subline, is paralleled by an increase in the fraction of apoptotic cells of about 20% in PDB and 50% in MDB compared to parental LNCaP cells (Fig. 4d).

Prolonged BIC exposure increased ROS levels in resistant cells (Fig. 4e) and, presumably, induced genome instability thus driving activation of the DNA repair pathway. This was confirmed by the upregulation, in resistant cells, of PARP-1, a key protein in DNA repair [35] (Fig. 4f). This is consistent with results obtained in our [14, 36] and other [35, 37] laboratories demonstrating both in vitro and in vivo PARP-1 overexpression during PCa tumor progression. Currently, several PARP-inhibitors are utilized in phase I and II clinical trials as mono or combination therapies in many human cancers including PCa [38]. To explore the role of PARP-1 in CRPC proliferation, we treated our cell lines with two PARP1-inhibitors: ABT-888, a PARP catalytic inhibitor [39] and BSI-201, a drug that disrupts the interaction between PARP-1 and DNA [40]. The results, reported in the Additional file 1: Figure S4, show that ABT-888 inhibited LNCaP cell line and only partially MDB, while BSI-201 selectively affected PDB and MDB growth in a dose-dependent manner. To find out a possible relationship between the AKT and p38MAPK signaling pathways and PARP-1 overexpression, cells were simultaneously treated for 120 h with BSI-201 in the presence of W or SB. Although these treatments only showed additive effects, they resulted more effective in resistant cells (Fig. 4g) and, importantly, more powerful than enzalutamide (Fig. 3c).

### Analysis of molecular alterations in CRPC insurgence

To investigate the phenotypic changes occurred in our androgen-independent cell lines, we performed an integrative transcriptomic and proteomic analysis followed by comparative expression profiling studies.

Gene expression analysis, performed on Affymetrix arrays, and proteomic profile, determined by MS, identified a total of 13,732 genes and 3943 proteins in LNCaP, PDB and MDB. The two approaches had in common 3629 elements, thus it was possible to correlate the gene expression values with approximately 92% of the identified proteins, allowing a good integration of the two data sets. Hierarchical clustering analysis based on transcriptomic and proteomic expression profiles showed differences among the three cell lines with a clear separation between parental LNCaP and BIC-resistant cell lines (Additional file 1: Figure S5). BIC treatment is a major driver of transcriptional differences in both BIC-resistant cell lines, whereas the absence of the androgen introduced remarkable changes at the protein level between MDB and both LNCaP and PDB cell lines.

We then identified the differentially expressed genes (DEGs) and proteins (DEPs) in order to define a molecular signature associated with anti-androgen resistance. We performed a comparative analysis of genomic and proteomic data, defining four groups: genes or proteins differentially expressed in PDB compared to LNCaP (PDBvsLNCaP), in MDB compared to LNCaP (MDBvsLNCaP), in MDB compared to PDB (MDBvsPDB) and in both PDB and MDB compared to LNCaP (MDB&PDBvsLNCaP) (Table 1). Overall, this analysis revealed that only 9.3% of genes and 63.2% of proteins were significantly modulated in LNCaP, PDB and MDB cell lines indicating that changes at mRNA level were not predictive of proteomic changes in prostate tumors, in line with previous observations [41]. In particular, the resistant-associated signature (MDB&PDBvsLNCaP) was characterized by only 181 DEGs and 18 DEPs while comparison between the two resistant cell lines (MDBvsPDB) showed that 55.3% proteins and 2.8% genes were deregulated. Finally, the common feature of DEGs and DEPs was the predominance of down-regulated gene (73.7%) or protein (96.4%) expression especially in the more aggressive MDB cell lines.

**Table 1:** Differentially expressed genes (DEGs) and proteins (DEPs)

Group	DEGs			DEPs		
	TOT (%)	UP	DOWN	TOT (%)	UP	DOWN
PDB vs LNCaP	245 (19.1)	54	191	29 (1.2)	11	18
MDB vs LNCaP	823 (64.0)	245	578	1068 (42.8)	46	1022
MDB vs PDB	36 (2.8)	11	25	1378 (55.3)	33	1345
MDB & PDB vs LNCaP	181 (14.1)	28	153	18 (0.7)	1	17
total	1285	338	947	2493	91	2402

**Fig. 5:**
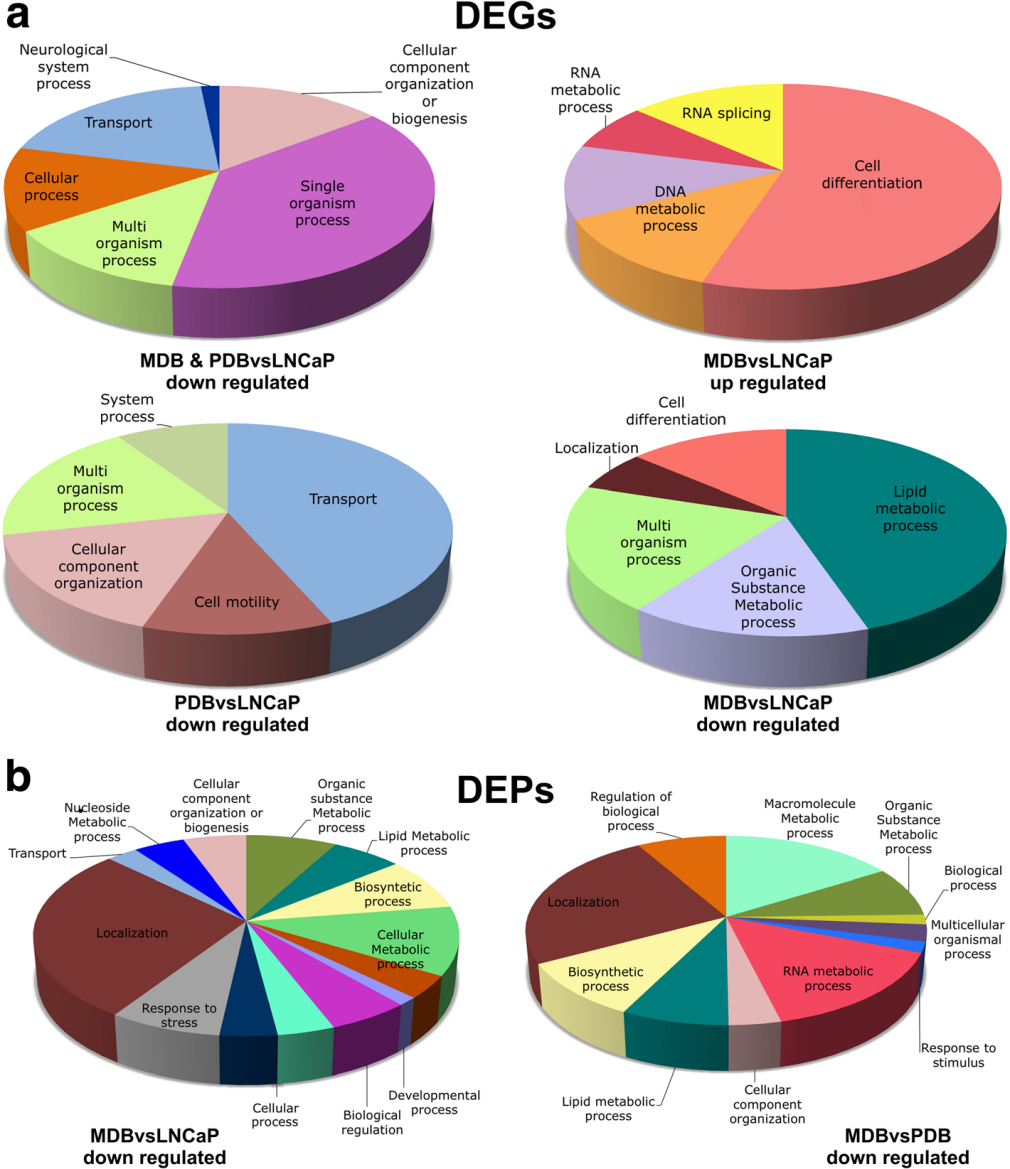
Pie charts representing the level 1 GO term of overrepresented GO-BPs obtained by the significantly up-regulated and down-regulated DEGs and DEPs. **a** Enriched terms from DEGs of MDB&PDB vs LNCaP (top-left), MDBvsLNCaP (top-right, bottom-right) and PDBvsLNCaP (lower-left) comparisons. **b** Enriched terms from DEPs of MDBvsLNCaP (left) and MDBvsPDB (right) comparisons. The pie charts were created using ClueGO Cytoscape plugin; the size of each section is the sum of the associated genes percentage for each GO group; the name and the color correspond to the level 1 GO-BP term and the network GO group, respectively

To identify the biological processes involved in CRPC insurgence, we investigated the functional annotation and interrelation of DEGs and DEPs in each of the four analyzed groups. We enriched, accordingly to Gene Ontology Biological Processes (GO-BP), terms inferred from experiments by using ClueGO (Additional file 1: Tables S3, S4). Charts (Fig. 5) and networks (Additional file 1: Figures S6, S7, S8) showing the functional groups and their connection were generated for a more visual comparative understanding of GO-BP distribution. Statistically significant enrichments were obtained for down-DEGs in PDBvsLNCaP and in MDB&PDBvsLNCaP groups as well as in MDBvsLNCaP up- or down-DEGs (Fig. 5a). Performing the same analysis on DEPs, we obtained a high number of clusters exclusively from down-regulated proteins in MDBvsLNCaP and MDBvsPDB comparisons (Fig. 5b).

Overall, GO-BP analysis revealed that: 1) down-DEGs in PDBvsLNCaP were mainly associated with tumor motility and ion transport; 2) in MDBvsLNCaP both down-DEGs and DEPs showed enrichment for biological processes related to cell differentiation, response to stress, regulation of metabolic pathways, cellular component localization and organization, while up-DEGs were involved in mRNA splicing and DNA repair; 3) down-DEPs in MDBvsPDB affected metabolic processes and their regulation by protein synthesis, localization and post-translational modifications (phosphorylation, methylation, acetylation); 4) most down-DEGs in MDB&PDBvsLNCaP were involved in single-organism processes associated with inflammation as regulation of tumor necrosis factor and chemokine production.

These analyses highlighted that prolonged BIC exposure mainly influenced biological processes involved in response to stress. Maximum AR function inhibition induced additional adaptation mechanisms showing the most consistent protein expression changes that shaped a complex interaction networks defining the highly-perturbed phenotype observed in MDB cells.

### Identification of CRPC-associated landscape in PDB and MDB

Since PDB and MBD cell lines appear a reliable model to study CRPC cell populations, we sought to identify genes and proteins discriminating LNCaP, MDB and PDB cell lines, and that might represent potential biomarkers and/or therapeutic targets for PCa progression during ADT.

By intersecting the DEGs and DEPs of the four groups we found limited concordance between change in mRNA and corresponding protein between all groups except for the MDBvsLNCaP (Additional file 1: Figure S9). We found three down-regulated genes/proteins discriminating BIC-resistant cell lines from LNCaP cells: ADAMTS1, identified as a possible tumor suppressor and two cell adhesion proteins involved in PCa progression, NCAM2 and UTRN. When compared to PDB, MDB cells showed up-regulation of the calcium-binding protein calnexin (CANX) and down-regulation of three genes/proteins: the ETS homologous factor EHF, an enzyme involved in oxidative stress tolerance, the ALDH3A2 implicated in metabolic signaling pathways and the RNA-binding protein quaking (QKI) (for more details see Additional file 1: Table S5). The reliability of this approach was confirmed by the low expression of the AR-regulated genes/proteins KLK3 and TMPRSS2 observed exclusively in the MDBvsLNCaP group as a result of a non-functioning AR.

**Fig. 6:**
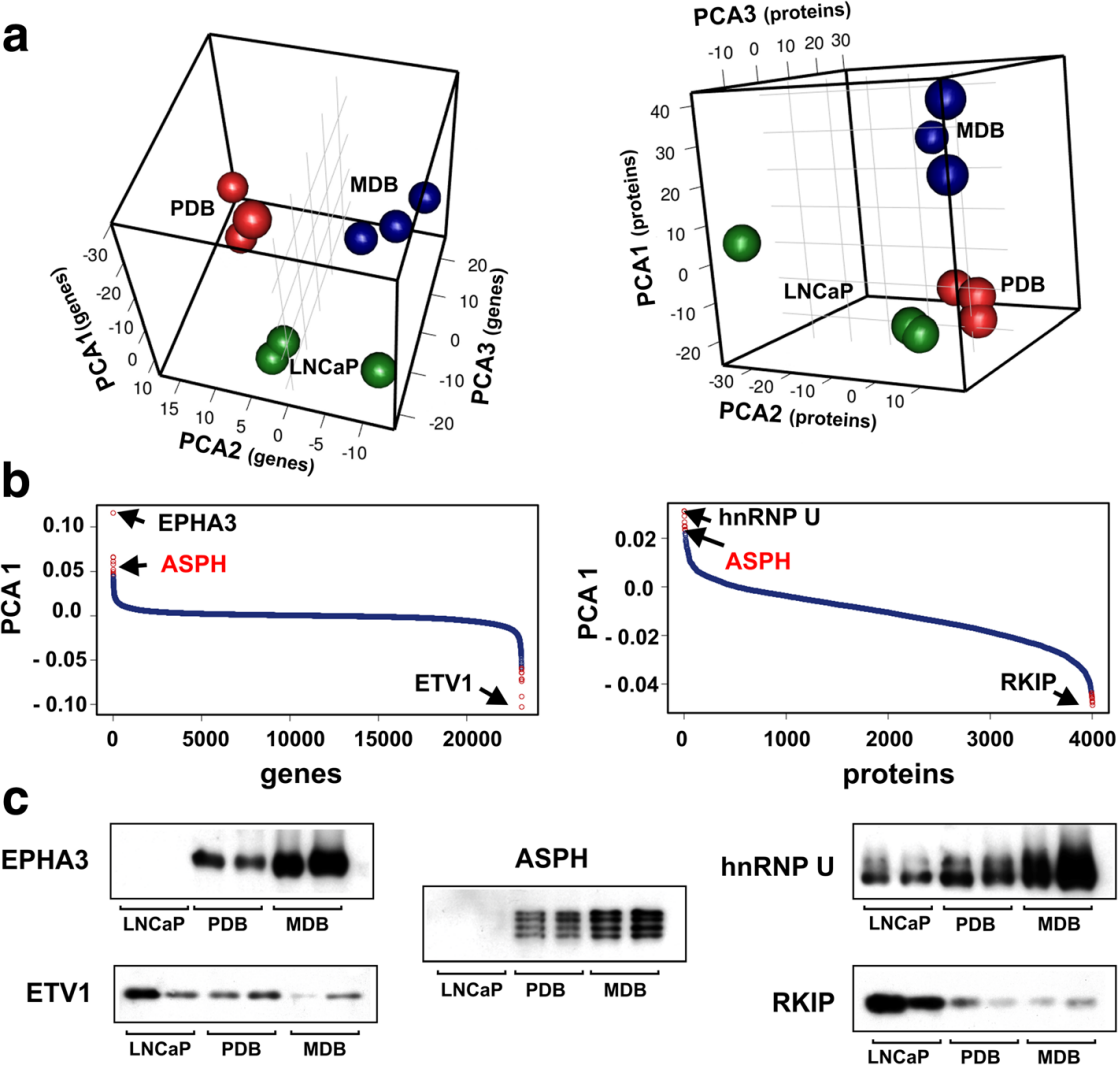
PCA of genes and proteins in LNCaP, MDB and PDB cell lines. **a** Three dimensional principal component sphere plots. Each sphere represents a technical replicate and the cell lines are associated to different colors. **b** Loading plot for the first principal component showing gene probes (left) and proteins (right) responsible for the clustering of samples. The scores were listed in descending order, having on the left the positive values and on the right the negative ones. The top genes (EPHA3, ETV1) and proteins (hnRNP U, RKIP) are indicated by arrows. In red the top ASPH gene/protein found in both datasets. **c** WB analysis of proteins from LNCaP, PDB and MDB. Expression level of the top five PCA gene/protein responsible for the maximal variation among data, was evaluated with antibodies to EPHA3, ETV1, hnRNP U, RKIP and ASPH

We next performed a multivariate principal component analysis (PCA) to identify genes and proteins that can represent a molecular signature associated with CRPC phenotype. Plotting the first three principal components obtained by analysis of transcriptomic and proteomic data for each technical replicate, we observed a good separation among the three groups indicating that their behaviors were significantly different (Fig. 6a). We then extracted the top loadings on the principal components to identify those elements responsible for the variation among data (Fig. 6b). Ranking them in a descending order we found that three genes, with a high loading score, were largely separated from all the others. The top positive was EPHA3, while the top negative was ETV1. Concerning the proteomic data, the separation was much less evident, and most of the proteins occupied the negative side of the graph, determining a different trend line with respect to genes. We considered the top positive hnRNP U and the top negative RKIP as more informative. Furthermore, ASPH was found in the top ranked list common to both datasets. To validate PCA results, we used immunoblotting analysis. As shown in Fig. 6c, and in agreement with the bioinformatic results, EPHA3, hnRNP U and ASPH were over expressed while ETV1 and RKIP expression was down-regulated in BIC-resistant cell lines.

Together, our results suggest that BIC-treatment induced molecular changes comparable to those highlighted in PCa patients (see references reported in Additional file 1: Table S5) and identify a protein set that can be associated to BIC-resistance (MDB&PDBvsLNCaP) and/or discriminating CRPC progression based on a compromised AR-signaling axis (MDBvsPDB).

## Discussion

The ‘omic’ technologies have delineated a highly heterogeneous landscape of molecular alterations in CRPC tissues that may influence patient prognosis and response to therapy [5, 33, 41]. While most CRPC still maintain AR-mediated signaling activity, AR-independent pathways are also involved in resistance to ADT, decreasing androgen receptor signaling inhibitors treatment effectiveness [2–4].

Besides genomic alterations identified both in PCa and CRPC patients, cancer treatments induce adaptive stress responses, associated with a combination of epigenetic and non-genetic alterations. Clonal heterogeneity and phenotypic plasticity, amplified by anticancer therapies, may further contribute to ADT failure through a complex regulation network strongly dependent on environmental factors driving a “non-genetic” heterogeneity [7, 8, 42]. The identification of the molecular alterations sustaining these adaptive states could offer new opportunities for CRPC patients selection and management, preventing disease progression and therapy resistance.

For this purpose, we developed in vitro models to mimic the CRPC cellular populations present in ADT patients by treating LNCaP cells for over one year with BIC, either in the presence or absence of DHT. We demonstrated that both resistant sublines: 1) do not express neuroendocrine, stem-like and EMT differentiation markers, although characterized by smaller size and faster growth rate; 2) have developed a major aggressiveness, both in vitro and in vivo; 3) express an AR able to translocate into the nucleus but with a reduced transcriptional activity correlated to a global dephosphorylation status; 4) their cell growth and survival is associated with AKT and p38MAPK activation and PARP-1 overexpression. The main differences observed between the two BIC-resistant cell lines were related to residual AR activity in PDB that displayed an intermediate cellular phenotype with LNCaP cells.

Since most PDB and MDB features are common with CRPC patients, BIC-resistant cell lines could be a useful tool for mechanistic studies and pharmacological screenings.

Through an integrative analysis of transcriptomic and proteomic data, we studied the molecular changes characterizing the three cell lines. Prolonged BIC-treatment induced non-genetically mediated alterations that determined adaptation to stress, while the absence of androgen during the MDB culture selection enhanced oxidative stress supporting the increase of migratory and metastasizing capacity and the resistance to the second generation anti-androgen drug enzalutamide.

**Fig. 7:**
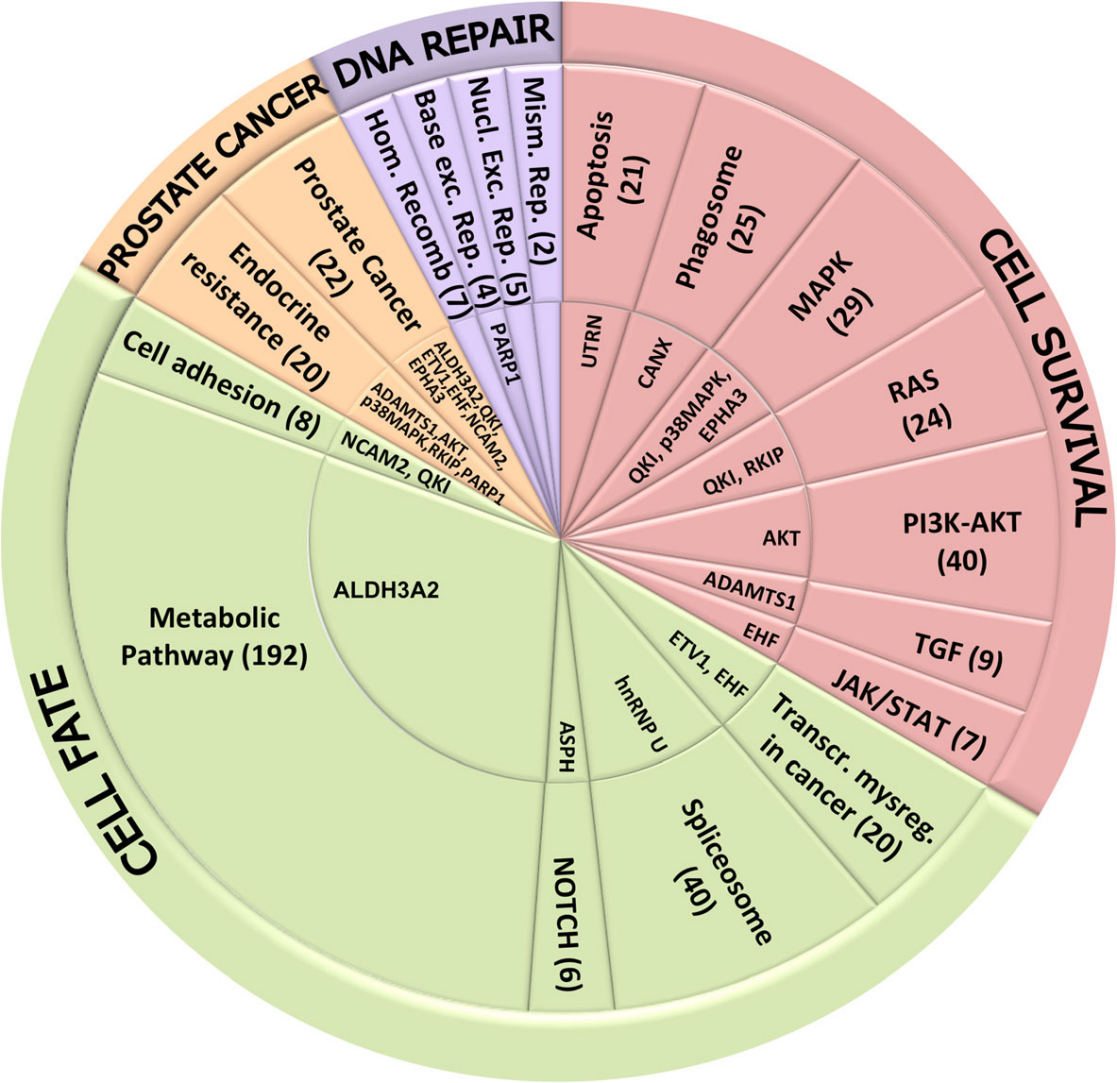
Cellular processes and pathways dysregulated in PDB and MDB. The 15 proteins found dysregulated in our CRPC models by in vitro experiments and bioinformatic analyses (inner circle) were distributed into one or more of 18 KEGG pathways affected in cancer (middle ring). These pathways, in turn, were grouped into four core cellular processes (outer ring). DEGs and DEPs number is shown in brackets (for more details see Additional file 1: Table S6)

BIC-resistant cell lines showed a global down-regulation of gene and protein expression that could reflect the phenotypic reversion of the resistant cell lines to a pluripotent state as described for mouse embryonic stem cells [43]. The maintenance of this undifferentiated phenotype capable of self-renewal depended both on gene regulatory networks and epigenetic processes and could drive the survival and the insurgence of resistance to therapeutic treatments [7, 43]. Studies have revealed that cell development depends on the coordinate activation of few pathways that regulate the expression of specific transcription factors involved in a broad range of cellular processes [6, 10]. In cancer cells this pathways network is dysregulated and the main genes “driving” tumorigenesis, classified into 12 signaling pathways, caused a selective growth advantage by regulating cell fate, survival and genome integrity [6]. Our results demonstrated that in PDB and MDB several pathways involved in cell fate, survival and DNA repair were dysregulated (Fig. 7 and Additional file 1: Table S6), supporting the hypothesis of their involvement in drug resistance. Androgen-resistant cell survival was supported by the activation of the two signaling pathways AKT and p38MAPK, in agreement with studies on tissues from CRPC patients [2, 3, 33]. The roles of PARP-1 in genome maintenance and transcriptional regulation during PCa progression have been reported [14, 36]. PARP-1 overexpression could thus explain the extensive gene expression modulation observed in our CRPC models. Prolonged BIC exposure also deregulated cellular and macromolecule metabolic processes and other adaptation mechanisms related to ionic transport, inflammation and extracellular matrix remodeling.

Selection in androgen depleted medium determined, in MDB cell line, an even more perturbed environment characterized by higher levels of apoptosis and ROS (Fig. 4d and e) and activation of additional adaptive pathways related to gene expression regulation (alternative splicing and post-transcriptional modifications). Importantly, many DEPs were associated with metabolic processes involved in histone modification and chromatin organization (Additional file 1: Table S4), that could contribute to heritable epigenetic changes via chromatin remodeling [44]. It was proposed that DNA damage give rise to mRNA splicing by post-translational modification of splicing factor [45] and alternative splicing of the components of the two critical pathways in PCa, AR and PI3K, was involved both in cancer development and in therapy escape [46]. The observation that over 40 DEGs/DEPs belonging to the spliceosome pathway (Additional file 1: Table S6) and the overexpression both in PDB and MDB of hnRNP U, a protein that exerts a global control of alternative splicing [47], were in line with these findings. Depending on the environmental pressure, ADT can produce different adaptive phenotypes leading to the heterogeneous drug resistance observed in CRPC patients.

Our study identified, by in vitro experiments and bioinformatic analyses, 15 proteins associated with a resistant phenotype that could represent potential effectors of AR dependent and independent adaptive mechanisms (innermost circle in Fig. 7, Additional file 1: Table S6). It is not surprising that 11 of these proteins are closely related to biological processes described for PCa evolution (see references in Additional file 1: Table S5). Deregulated expression of two ETS homologous transcription factor, ETV1, that cooperate with AR to regulate gene transcription, and EHF, that play a role in regulating epithelial cell differentiation and proliferation, were found associated with most aggressive PCa. ADAMTS1 and QKI were identified as possible PCa tumor suppressors, as their expression was down-regulated, respectively, in metastatic CRPC and in poorly differentiated PCa. Altered expression of the cell adhesion protein NCAM2, the EPHA3 receptor tyrosine kinase related to cell motility during carcinogenesis and the aldehyde dehydrogenase ALDH3A2, implicated in metabolic signaling pathways, were also involved in PCa progression. A possible role in CRPC insurgence was described for AKT, p38MAPK, PARP-1 and RKIP, a Raf kinase inhibitor. UTRN, CANX and ASPH expression changes were observed in some primary tumors indicating their possible involvement also in PCa evolution. As these proteins were dysregulated at the onset of the CRPC phenotype, it is conceivable they could be markers of predictive and prognostic value as well as targets for innovative therapies. We are thus planning to validate our findings on PCa and CRPC specimens or liquid biopsies. At the same time, specific inhibitors for some of the molecules/pathways identified in the present study will be tested alone or in combination therapies both in vitro and in vivo mouse models.

## Conclusions

Our results supports the hypothesis that the cytotoxic stress exerted by ADT models the adaptive phenotype of PCa cells promoting heterogeneous CRPC insurgence and development. Depending on the environmental pressure (i.e. residual androgen presence/absence), phenotypic adaptation can also originate from non-genetic dynamics, which in turn activate DNA repair, cell fate and survival pathways. RNA splicing and cellular metabolic processes may further amplify the effects creating highly connected regulatory networks in which key proteins participate in multiple interactions. Targeting these molecular alterations could improve CRPC therapy allowing a more tailored use of available resources and the development of new therapeutic strategies**.**

## Additional file

Additional file 1: Figure S1. Selection of BIC-resistant sublines. **Figure S2.** Neuroendocrine, stem-like phenotype and EMT cell markers expression in LNCaP, PDB and MDB. **Figure S3.** ERK expression and activity are not significantly affected by prolonged BIC exposure. **Figure S4.** Sensitivity of BIC-resistant sublines to PARP-1 inhibitors with different mechanisms of action. **Figure S5.** Heatmaps from hierarchical clustering of gene (left) and protein (right) mean expression data. **Figure S6.** Functionally grouped networks of GO-BPs (Gene Ontology Biological Processes) terms over-represented among the genes with elevated (red) or reduced (blue) expression in each comparison. **Figure S7.** Functionally grouped networks of GO-BPs (Gene Ontology Biological Processes) terms over-represented among the proteins with reduced expression in MDBvsLNCaP comparison. **Figure S8.** Functionally grouped networks of GO-BPs (Gene Ontology Biological Processes) terms over-represented among the proteins with reduced expression in MDBvsPDB comparison. **Figure S9.** Venn diagrams of significant differentially expressed genes (DEGs) and proteins (DEPs). **Table S1.** List of the antibodies used for WB analysis. **Table S2.** Alleles of 9 STR loci and amelogenin gene detected in DNA from LNCaP, PDB and MDB cell lines. **Table S3.** DEGs enriched accordingly to GO-BP terms inferred from experiments by using ClueGO **Table S4.** DEPs enriched accordingly to GO-BP terms inferred from experiments by using ClueGO **Table S5.** The 15 proteins associated with CRPC phenotype. **Table S6.** DEGs and DEPs grouped by KEGG pathways involved in cell survival, cell fate, prostate cancer and DNA repair mechanisms. (DOCX 4880 kb)

## Abbreviations

ADT: Androgen deprivation therapy
AR: Androgen receptor
BIC: Bicalutamide
CRPC: Castration resistant prostate cancer
DEG: Differentially expressed gene
DEP: Differentially expressed protein
DHT: 5-α-dihydrotestosterone
GO: Gene ontology
LTQ: Linear trap quadrupole
MS: Mass spectrometry
PCA: Principal component analysis
PCa: Prostate cancer
RFU: Relative fluorescence units
SB: SB203580
STR: Short tandem repeat
W: Wortmannin
WB: Western blotting
