# Supplementary material for: Adaptive phenotype drives resistance to androgen deprivation therapy in prostate cancer
Source: Cell Commun Signal. 2017 Dec 8;15:51. doi: 10.1186/s12964-017-0206-x (PMC5721601; doi:10.1186/s12964-017-0206-x)
Supplement: Additional file 1: Figure S1. — Selection of BIC-resistant sublines. Figure S2. Neuroendocrine, stem-like phenotype and EMT cell markers expression in LNCaP, PDB and MDB. Figure S3. ERK expression and activity are not significantly affected by prolonged BIC exposure. Figure S4. Sensitivity of BIC-resistant sublines to PARP-1 inhibitors with different mechanisms of action. Figure S5. Heatmaps from hierarchical clustering of gene (left) and protein (right) mean expression data. Figure S6. Functionally grouped networks of GO-BPs (Gene Ontology Biological Processes) terms over-represented among the genes with elevated (red) or reduced (blue) expression in each comparison. Figure S7. Functionally grouped networks of GO-BPs (Gene Ontology Biological Processes) terms over-represented among the proteins with reduced expression in MDBvsLNCaP comparison. Figure S8. Functionally grouped networks of GO-BPs (Gene Ontology Biological Processes) terms over-represented among the proteins with reduced expression in MDBvsPDB comparison. Figure S9. Venn diagrams of significant differentially expressed genes (DEGs) and proteins (DEPs). Table S1. List of the antibodies used for WB analysis. Table S2. Alleles of 9 STR loci and amelogenin gene detected in DNA from LNCaP, PDB and MDB cell lines. Table S3. DEGs enriched accordingly to GO-BP terms inferred from experiments by using ClueGO Table S4. DEPs enriched accordingly to GO-BP terms inferred from experiments by using ClueGO Table S5. The 15 proteins associated with CRPC phenotype. Table S6. DEGs and DEPs grouped by KEGG pathways involved in cell survival, cell fate, prostate cancer and DNA repair mechanisms. (DOCX 4880 kb) [file 12964_2017_206_MOESM1_ESM.docx]

**Supplementary figure 1:** LNCaP cells (passage 26) were cultured in phenol red-free RPMI containing 10% charcoal stripped FBS with (PDB) or without 0.1 nM DHT (MDB) in the presence of increasing  concentrations  of BIC. After one month, BIC was maintained at the constant concentration of 10 µM for up to 12 months.


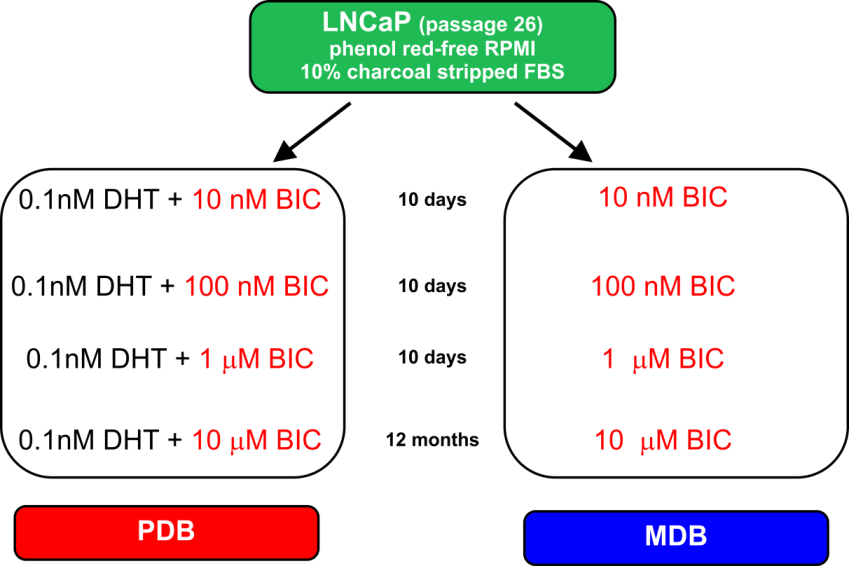


**Supplementary figure 2.** **A** WB analysis of synaptophysin (SYN), neuron-specific enolase (NSE), SOX2 and Nanog expression in the three cell lines. The corresponding Sypro Ruby stained gel is reported on the right. **B** Vimentin expression in LNCaP, PDB and MDB cells (bottom). PC3 and DU145 cell lines are reported as positive controls. The corresponding Sypro Ruby stained gel is reported (top panel).





**Supplementary figure 3.** Representative WB analysis of total proteins from LNCaP, PDB and MDB probed with antibodies against ERK and pERK. The corresponding Sypro Ruby stained gel is reported on the right.





**Supplementary figure 4.** Cell viability of LNCaP, PDB and MDB incubated for 120 hours with different concentration of ABT888 (**A**) and BSI201 (**B**).





**Supplementary figure 5.** Clustering analysis revealed different subgroups for the two datatsets: using normalized gene expression values, MBD and PDB formed one cluster, although we can note a unique profile for each cell line; in the case of normalized proteomics data, instead, one cluster is formed by LNCaP and PDB, while MDB showed a completely different profile.


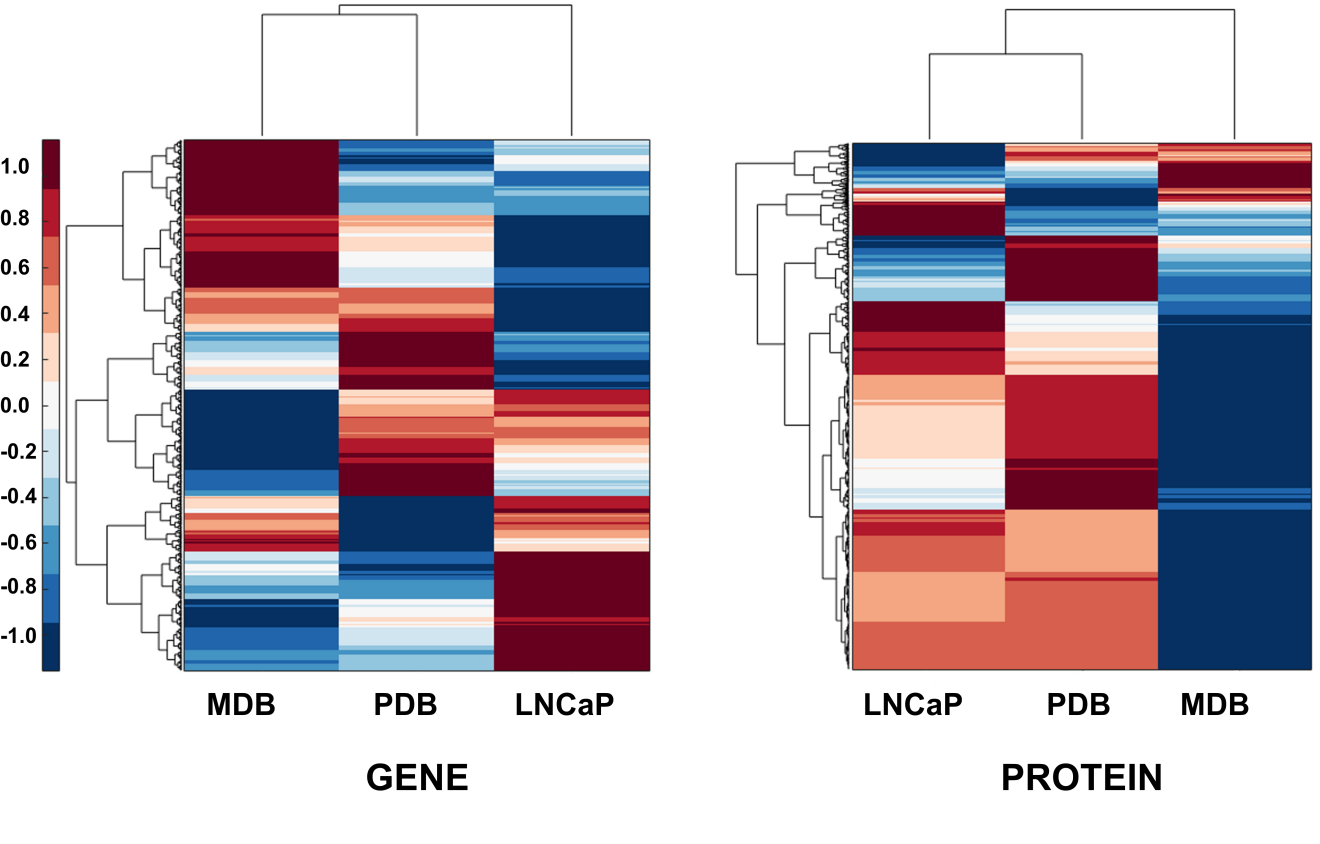


**Supplementary figure 6.** The nodes represent the BP, and the colors indicate the belonging GO groups (see Supplementary Table S4), with mixed colors for those sub-networks belonging to multiple GO groups. The enrichment significance of the GO terms is represented by the node size. The title of each sub-network represents the level 1 GO term and the relationship is recognizable by the colors.


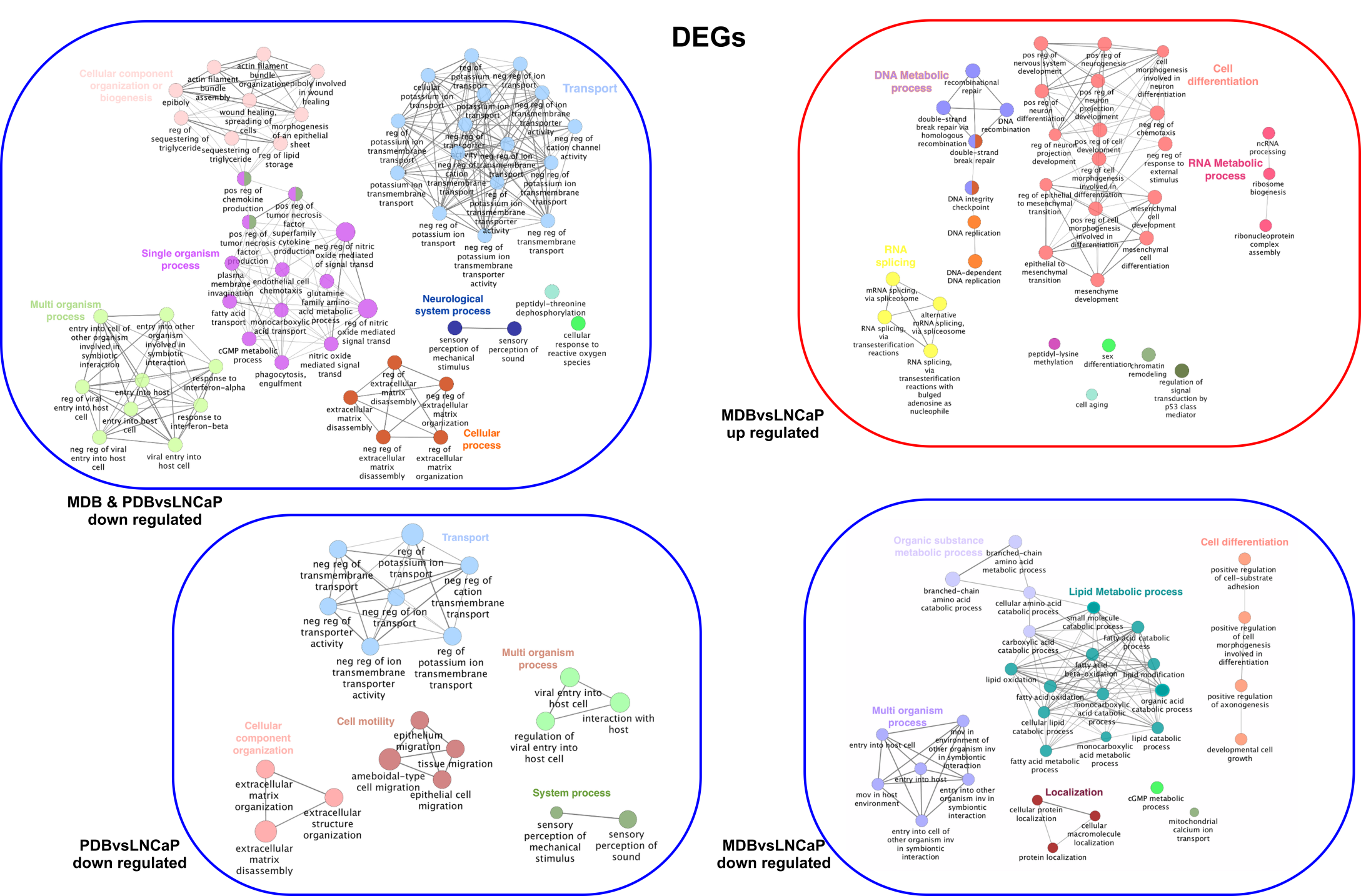


**Supplementary figure 7.** The nodes represent the BP, and the colors indicate the belonging GO groups (see Supplementary Table S5), with mixed coloring for those sub-networks belonging to multiple GO groups. The enrichment significance of the GO terms is represented by the node size. The title of each sub-network represents the level 1 GO term and the relationship is recognizable by the colors.

**
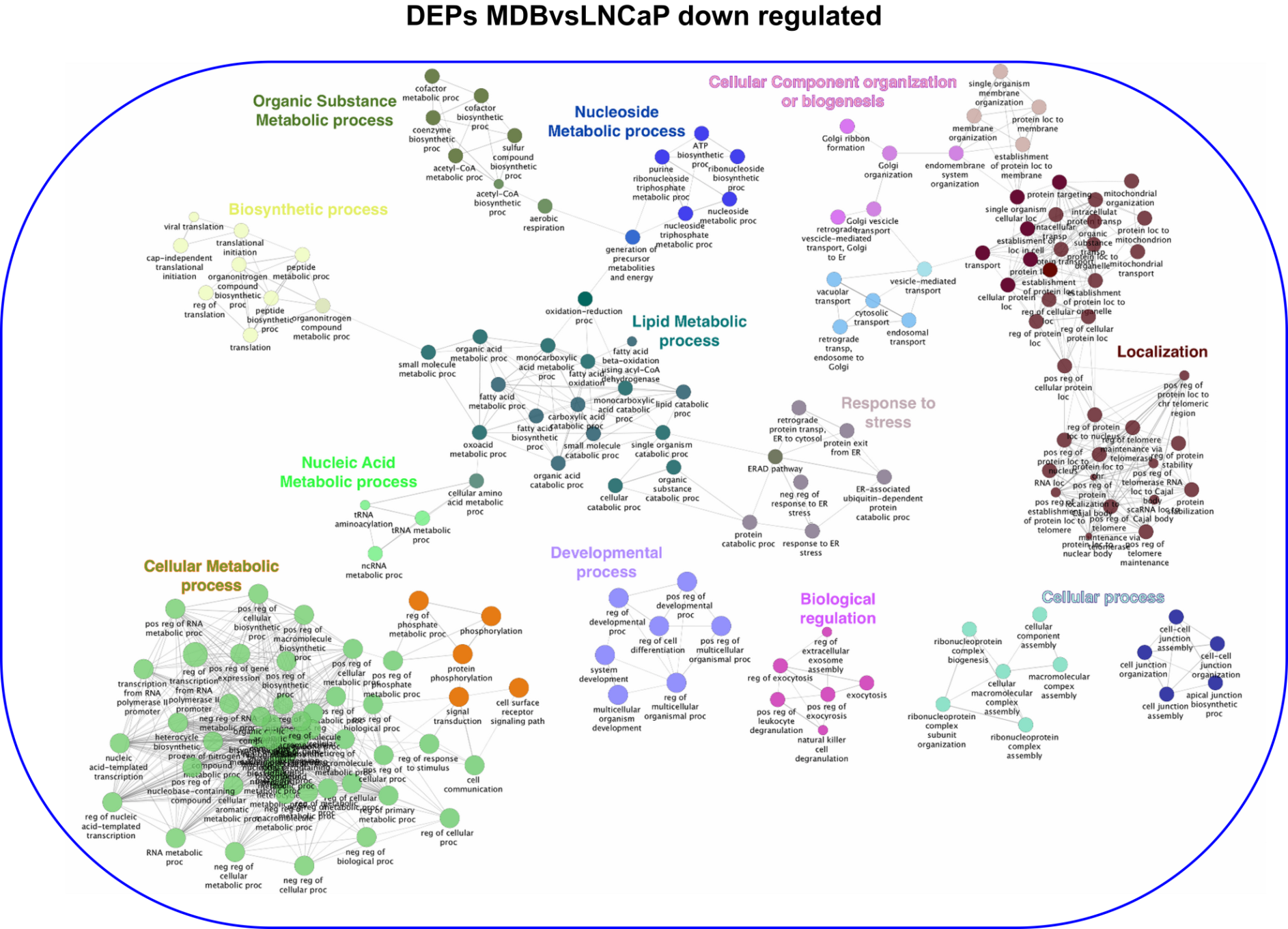
**

**Supplementary figure 8.** The nodes represent the BP, and the colors indicate the belonging GO groups (see Supplementary Table S5), with mixed coloring for those sub-networks belonging to multiple GO groups. The enrichment significance of the GO terms is represented by the node size. The title of each sub-network represents the level 1 GO term and the relationship is recognizable by the colors

**
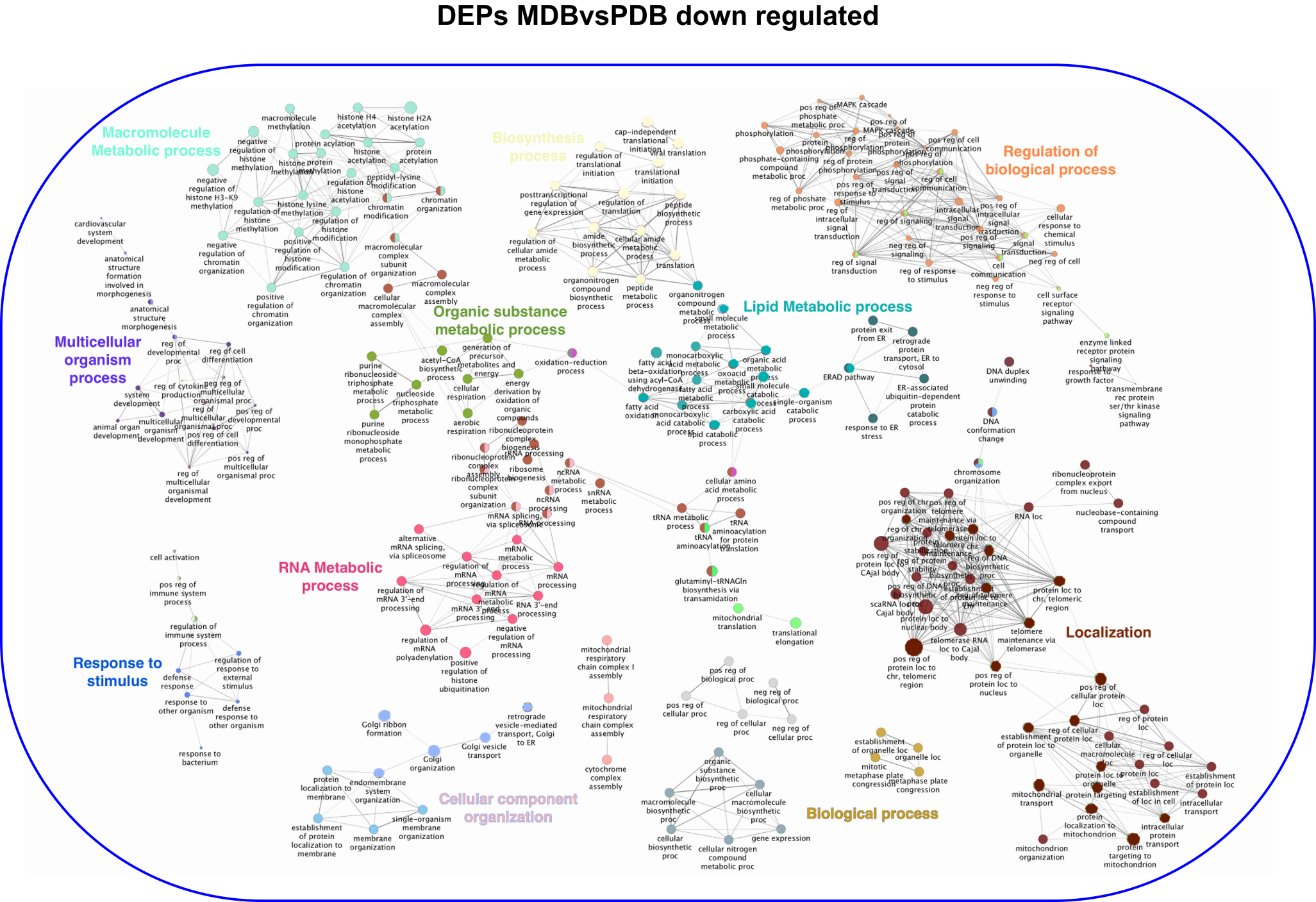
**

.

**Supplementary figure 9.** For each comparison we intersected the list of DEGs and DEPs having adjusted p-value ≤ 0.05 and |log2FC| ≥ 1. The number of common and specific elements are shown by the diagrams. On the right we reported the gene symbols corresponding to the DEGs and DEPs in common between the two experiments. In red genes/proteins discriminating BIC-resistant cell lines from LNCaP cells. In blue AR-regulated genes/proteins.


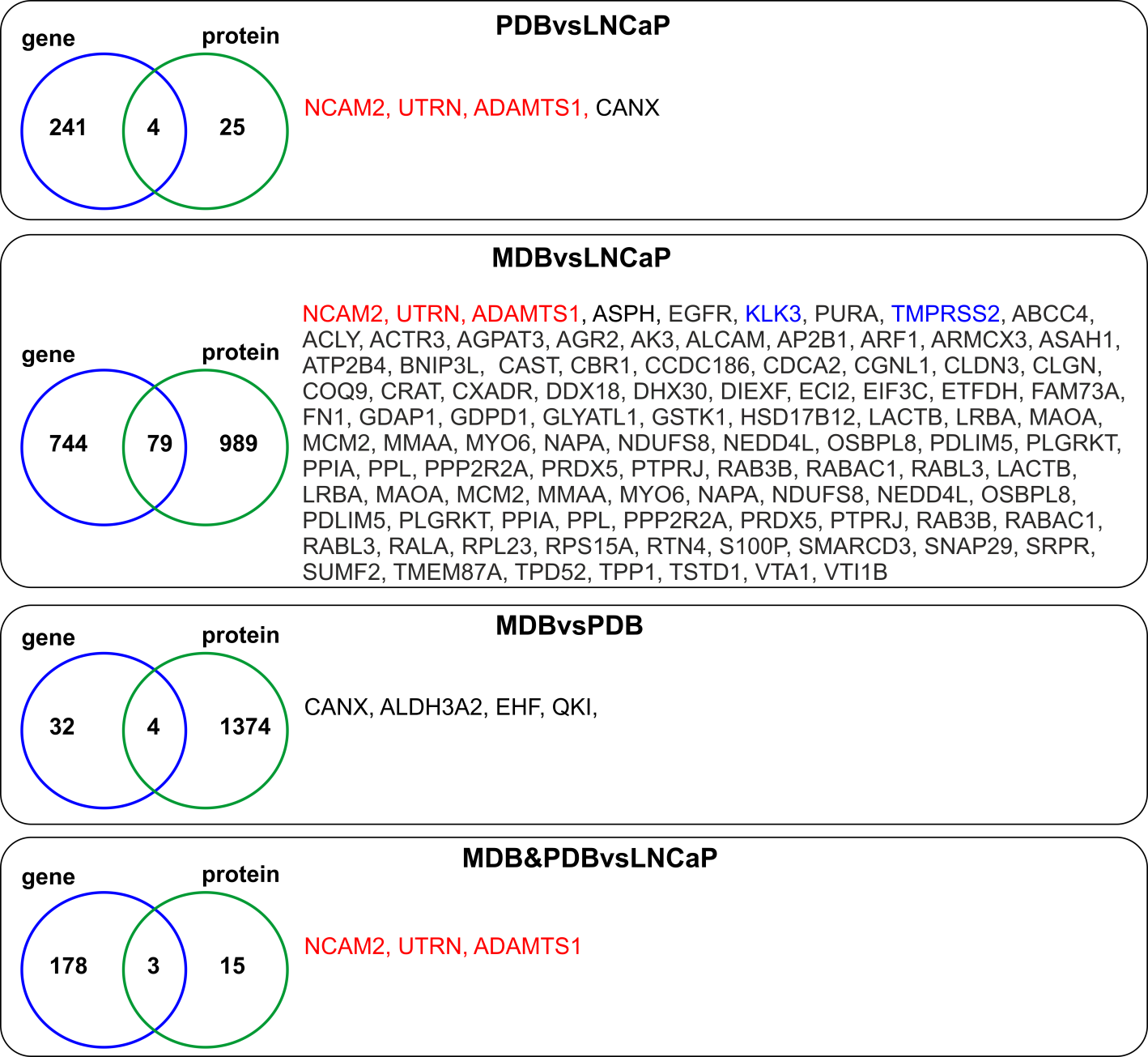


**Supplementary table 1**

| **Antigen** | **Type** | **Label** | **Host** | **Company** | **Dilution** |
| --- | --- | --- | --- | --- | --- |
| AKT | Polyclonal |  | Rabbit | Cell Signaling Technology (CST) | 1:1000 |
| AR | Monoclonal |  | Mouse | DAKO | 1:600 |
| EPHA3 | Polyclonal |  | Rabbit | Santa Cruz | 1:500 |
| ETV1 | Monoclonal |  | Mouse | Santa Cruz | 1:250 |
| hnRNP U | Monoclonal |  | Mouse | Santa Cruz | 1:3000 |
| Nanog | Monoclonal |  | Rabbit | CTS | 1:1000 |
| Neuron-specific enolase | Polyclonal |  | Rabbit | Zymed | 1:500 |
| PARP1 | Polyclonal |  | Rabbit | Santa Cruz | 1:500 |
| pAKT | Monoclonal |  | Rabbit | CTS | 1:1000 |
| p38MAPK | Polyclonal |  | Rabbit | CTS | 1:1000 |
| pp38MAPK | Polyclonal |  | Rabbit | CTS | 1:1000 |
| PSA | Polyclonal |  | Rabbit | CTS | 1:2000 |
| RKIP | Monoclonal |  | Rabbit | CTS | 1:2000 |
| SOX2 | Monoclonal |  | Rabbit | CTS | 1:1000 |
| Synaptophysin | Polyclonal |  | Rabbit | Zymed | 1:500 |
| Vimentin | Monoclonal |  | Mouse | SIGMA | 1:2000 |
| Mouse Ig G | Polyclonal | Peroxidase | Rabbit | DAKO | 1:1000 |
| Rabbit Ig G | Polyclonal | Peroxidase | Goat | CTS | 1:2000 |

**Supplementary table 2**

| **Locus** | **LNCaP** | **PDB** | **MDB** |
| --- | --- | --- | --- |
| Amelogenin | X, Y | X, Y | X, Y |
| D5S818 | 11, 12 | 10, 11, 13 | 10, 11, 13 |
| D13S317 | 10, 12 | 10, 11, 12 | 10, 11, 12 |
| D7S820 | 9 | - | 9 |
| D16S539 | 11 | 11 | 10, 11 |
| vWA | 16, 18 | 16, 18 | 16, 17, 18 |
| TH01 | 9 | 9 | 9 |
| TPOX | 8, 9 | 8, 9 | 8, 9 |
| SF1PO | 10, 11 | 10, 11 | 10, 11 |
| D21S11 | 29, 32.2 | 29, 32.2 | 29, 32.2 |

**Supplementary table 3**

| **Down DEGs in** **PDB vs LNCaP** | | | | | | | | | |  |
| --- | --- | --- | --- | --- | --- | --- | --- | --- | --- | --- |
| **Color Group** | **GOTerm** | **Term p-value** | **Term p-value Corrected with Benjamini-Hochberg** | **Group p-value** | **Group p-value Corrected with Benjamini-Hochberg** | **GO Levels** | **% Associated Genes** | **Nr. Genes** | **Associated Genes Found** |  |
|  | GO:0050954 sensory perception of mechanical stimulus | 16.0E-3 | 46.0E-3 | 16.0E-3 | 20.0E-3 | [5] | 6.38 | 3.00 | [ATP2B4, FAM65B, HOMER2] |  |
|  | GO:0007605 sensory perception of sound | 16.0E-3 | 46.0E-3 | 16.0E-3 | 20.0E-3 | [6] | 6.38 | 3.00 | [ATP2B4, FAM65B, HOMER2] |  |
|  | GO:0051701 interaction with host | 8.0E-3 | 27.0E-3 | 8.0E-3 | 12.0E-3 | [3, 4] | 5.80 | 4.00 | [FUCA2, IFITM2, IFITM3, TMPRSS2] |  |
|  | GO:0046718 viral entry into host cell | 9.3E-3 | 30.0E-3 | 8.0E-3 | 12.0E-3 | [5, 6, 7, 8] | 7.89 | 3.00 | [IFITM2, IFITM3, TMPRSS2] |  |
|  | GO:0046596 regulation of viral entry into host cell | 2.8E-3 | 31.0E-3 | 8.0E-3 | 12.0E-3 | [5, 6, 7, 8, 9] | 12.00 | 3.00 | [IFITM2, IFITM3, TMPRSS2] |  |
|  | GO:0090130 tissue migration | 4.0E-3 | 30.0E-3 | 1.7E-3 | 10.0E-3 | [3] | 3.98 | 7.00 | [CEL, DPP4, MARVELD3, NRP1, NUP93, RAB11A, THBS1] |  |
|  | GO:0090132 epithelium migration | 4.0E-3 | 30.0E-3 | 1.7E-3 | 10.0E-3 | [4] | 3.98 | 7.00 | [CEL, DPP4, MARVELD3, NRP1, NUP93, RAB11A, THBS1] |  |
|  | GO:0001667 ameboidal-type cell migration | 1.7E-3 | 26.0E-3 | 1.7E-3 | 10.0E-3 | [4, 5, 6] | 4.10 | 8.00 | [CEL, CENPV, DPP4, MARVELD3, NRP1, NUP93, RAB11A, THBS1] |  |
|  | GO:0010631 epithelial cell migration | 4.0E-3 | 30.0E-3 | 1.7E-3 | 10.0E-3 | [5, 6, 7] | 3.98 | 7.00 | [CEL, DPP4, MARVELD3, NRP1, NUP93, RAB11A, THBS1] |  |
|  | GO:0032410 negative regulation of transporter activity | 11.0E-3 | 34.0E-3 | 2.7E-3 | 8.2E-3 | [3, 4, 5, 6] | 7.32 | 3.00 | [GNB5, KCNRG, STK39] |  |
|  | GO:0034763 negative regulation of transmembrane transport | 1.4E-3 | 32.0E-3 | 2.7E-3 | 8.2E-3 | [3, 4, 5, 6] | 9.30 | 4.00 | [GNB5, KCNRG, STK39, THBS1] |  |
|  | GO:0043271 negative regulation of ion transport | 3.8E-3 | 34.0E-3 | 2.7E-3 | 8.2E-3 | [3, 4, 5, 6] | 7.14 | 4.00 | [GNB5, KCNRG, STK39, THBS1] |  |
|  | GO:0032413 negative regulation of ion transmembrane transporter activity | 6.8E-3 | 34.0E-3 | 2.7E-3 | 8.2E-3 | [4, 5, 6, 7, 8] | 8.82 | 3.00 | [GNB5, KCNRG, STK39] |  |
|  | GO:1901379 regulation of potassium ion transmembrane transport | 5.7E-3 | 32.0E-3 | 2.7E-3 | 8.2E-3 | [3, 4, 5, 6, 7, 8, 9, 10] | 9.38 | 3.00 | [KCNN2, KCNRG, STK39] |  |
|  | GO:1904063 negative regulation of cation transmembrane transport | 7.4E-3 | 33.0E-3 | 2.7E-3 | 8.2E-3 | [5, 6, 7, 8] | 8.57 | 3.00 | [GNB5, KCNRG, STK39] |  |
|  | GO:0043266 regulation of potassium ion transport | 8.0E-3 | 30.0E-3 | 2.7E-3 | 8.2E-3 | [6, 7, 8] | 8.33 | 3.00 | [KCNN2, KCNRG, STK39] |  |
|  | GO:0043062 extracellular structure organization | 7.6E-3 | 31.0E-3 | 7.6E-3 | 15.0E-3 | [3] | 5.88 | 4.00 | [CEL, DPP4, LCP1, PXDN] |  |
|  | GO:0030198 extracellular matrix organization | 7.6E-3 | 31.0E-3 | 7.6E-3 | 15.0E-3 | [4] | 5.88 | 4.00 | [CEL, DPP4, LCP1, PXDN] |  |
|  | GO:0022617 extracellular matrix disassembly | 4.3E-3 | 28.0E-3 | 7.6E-3 | 15.0E-3 | [4, 5] | 10.34 | 3.00 | [CEL, DPP4, LCP1] |  |
| **Down DEGs in** **MDB vs LNCaP** | | | | | | | | | |  |
|  | GO:0006338 chromatin remodeling | 5.7E-3 | 17.0E-3 | 5.7E-3 | 14.0E-3 | [5] | 6.45 | 4.00 | [CHD1L, CHD4, RBBP4, SMARCD3] |  |
|  | GO:0007548 sex differentiation | 16.0E-3 | 22.0E-3 | 16.0E-3 | 20.0E-3 | [3, 4] | 6.52 | 3.00 | [AMH, CBX2, ROBO2] |  |
|  | GO:0007569 cell aging | 38.0E-3 | 40.0E-3 | 38.0E-3 | 38.0E-3 | [4] | 4.69 | 3.00 | [AMH, LMNA, SMC5] |  |
|  | GO:0032102 negative regulation of response to external stimulus | 7.4E-3 | 14.0E-3 | 2.3E-3 | 11.0E-3 | [3, 4, 5] | 4.76 | 5.00 | [KANK2, NOTCH1, ROBO1, ROBO2, RPS19] |  |
|  | GO:0050922 negative regulation of chemotaxis | 1.2E-3 | 11.0E-3 | 2.3E-3 | 11.0E-3 | [3, 4, 5, 6] | 15.79 | 3.00 | [NOTCH1, ROBO1, ROBO2] |  |
|  | GO:0051962 positive regulation of nervous system development | 4.0E-3 | 13.0E-3 | 2.3E-3 | 11.0E-3 | [3, 4, 5, 6, 7] | 5.49 | 5.00 | [EPHA3, NLGN1, ROBO1, ROBO2, SMARCD3] |  |
|  | GO:0060485 mesenchyme development | 12.0E-3 | 18.0E-3 | 2.3E-3 | 11.0E-3 | [4, 5, 6] | 5.19 | 4.00 | [DAB2IP, LEF1, NOTCH1, TGFBR1] |  |
|  | GO:0010720 positive regulation of cell development | 1.4E-3 | 9.8E-3 | 2.3E-3 | 11.0E-3 | [4, 5, 6, 7] | 4.83 | 7.00 | [EPHA3, LEF1, NLGN1, NOTCH1, ROBO1, ROBO2, SMARCD3] |  |
|  | GO:0010769 regulation of cell morphogenesis involved in differentiation | 2.4E-3 | 13.0E-3 | 2.3E-3 | 11.0E-3 | [5, 6, 7] | 5.08 | 6.00 | [DAB2IP, LEF1, NLGN1, NOTCH1, ROBO1, ROBO2] |  |
|  | GO:0010770 positive regulation of cell morphogenesis involved in differentiation | 7.1E-3 | 14.0E-3 | 2.3E-3 | 11.0E-3 | [4, 5, 6, 7, 8] | 6.06 | 4.00 | [LEF1, NOTCH1, ROBO1, ROBO2] |  |
|  | GO:0014031 mesenchymal cell development | 7.5E-3 | 13.0E-3 | 2.3E-3 | 11.0E-3 | [4, 5, 6, 7, 8] | 5.97 | 4.00 | [DAB2IP, LEF1, NOTCH1, TGFBR1] |  |
|  | GO:0048762 mesenchymal cell differentiation | 7.9E-3 | 13.0E-3 | 2.3E-3 | 11.0E-3 | [5, 6, 7] | 5.88 | 4.00 | [DAB2IP, LEF1, NOTCH1, TGFBR1] |  |
|  | GO:0050769 positive regulation of neurogenesis | 3.3E-3 | 16.0E-3 | 2.3E-3 | 11.0E-3 | [4, 5, 6, 7, 8, 9] | 5.75 | 5.00 | [EPHA3, NLGN1, ROBO1, ROBO2, SMARCD3] |  |
|  | GO:0001837 epithelial to mesenchymal transition | 6.4E-3 | 15.0E-3 | 2.3E-3 | 11.0E-3 | [5, 6, 7, 8, 9] | 6.25 | 4.00 | [DAB2IP, LEF1, NOTCH1, TGFBR1] |  |
|  | GO:0010717 regulation of epithelial to mesenchymal transition | 20.0E-3 | 27.0E-3 | 2.3E-3 | 11.0E-3 | [4, 5, 6, 7, 8, 9, 10] | 6.00 | 3.00 | [DAB2IP, LEF1, NOTCH1] |  |
|  | GO:0045666 positive regulation of neuron differentiation | 10.0E-3 | 15.0E-3 | 2.3E-3 | 11.0E-3 | [5, 6, 7, 8, 9, 10] | 5.48 | 4.00 | [EPHA3, NLGN1, ROBO1, ROBO2] |  |
|  | GO:0048667 cell morphogenesis involved in neuron differentiation | 29.0E-3 | 34.0E-3 | 2.3E-3 | 11.0E-3 | [5, 6, 7, 8, 9, 10] | 5.17 | 3.00 | [NLGN1, ROBO1, ROBO2] |  |
|  | GO:0010975 regulation of neuron projection development | 26.0E-3 | 33.0E-3 | 2.3E-3 | 11.0E-3 | [5, 6, 7, 8, 9, 10, 11] | 4.12 | 4.00 | [EPHA3, NLGN1, ROBO1, ROBO2] |  |
|  | GO:0010976 positive regulation of neuron projection development | 6.4E-3 | 15.0E-3 | 2.3E-3 | 11.0E-3 | [5, 6, 7, 8, 9, 10, 11, 12] | 6.25 | 4.00 | [EPHA3, NLGN1, ROBO1, ROBO2] |  |
|  | GO:0018022 peptidyl-lysine methylation | 35.0E-3 | 38.0E-3 | 35.0E-3 | 39.0E-3 | [5, 6, 7, 8, 9] | 4.84 | 3.00 | [ASH1L, AUTS2, SMYD2] |  |
|  | GO:0031570 DNA integrity checkpoint | 3.8E-3 | 14.0E-3 | 150.0E-6 | 1.5E-3 | [4, 6] | 5.56 | 5.00 | [CDT1, DNA2, INTS7, POLR1B, RBBP8] |  |
|  | GO:0006260 DNA replication | 690.0E-6 | 23.0E-3 | 150.0E-6 | 1.5E-3 | [5, 6, 7] | 5.47 | 7.00 | [CDT1, DNA2, MCM2, POLR1B, PURA, RBBP6, RECQL4] |  |
|  | GO:0006261 DNA-dependent DNA replication | 6.4E-3 | 15.0E-3 | 150.0E-6 | 1.5E-3 | [6, 7, 8] | 6.25 | 4.00 | [CDT1, DNA2, MCM2, PURA] |  |
|  | GO:0031570 DNA integrity checkpoint | 3.8E-3 | 14.0E-3 | 4.8E-3 | 16.0E-3 | [4, 6] | 5.56 | 5.00 | [CDT1, DNA2, INTS7, POLR1B, RBBP8] |  |
|  | GO:0006302 double-strand break repair | 3.8E-3 | 16.0E-3 | 150.0E-6 | 1.5E-3 | [6, 7, 8] | 4.65 | 6.00 | [DNA2, POLR1B, RBBP8, SFPQ, SMC5, XRCC2] |  |
|  | GO:0042254 ribosome biogenesis | 32.0E-3 | 36.0E-3 | 6.9E-3 | 13.0E-3 | [4] | 5.00 | 3.00 | [DHX30, RPL35A, RPS19] | [DHX30, RPL35A, RPS19] |
|  | GO:0000725 recombinational repair | 850.0E-6 | 9.6E-3 | 4.8E-3 | 16.0E-3 | [6, 7, 8] | 7.81 | 5.00 | [POLR1B, RBBP8, SFPQ, SMC5, XRCC2] |  |
|  | GO:0006310 DNA recombination | 6.3E-3 | 16.0E-3 | 4.8E-3 | 16.0E-3 | [5, 6, 7] | 4.95 | 5.00 | [POLR1B, RBBP8, SFPQ, SMC5, XRCC2] |  |
|  | GO:0000724 double-strand break repair via homologous recombination | 790.0E-6 | 13.0E-3 | 4.8E-3 | 16.0E-3 | [7, 8, 9] | 7.94 | 5.00 | [POLR1B, RBBP8, SFPQ, SMC5, XRCC2] |  |
|  | GO:0022618 ribonucleoprotein complex assembly | 27.0E-3 | 33.0E-3 | 6.9E-3 | 13.0E-3 | [4, 5, 6] | 4.08 | 4.00 | [DHX30, RPS19, SCAF11, SRSF1] |  |
|  | GO:0034470 ncRNA processing | 24.0E-3 | 32.0E-3 | 6.9E-3 | 13.0E-3 | [6, 7, 8] | 4.21 | 4.00 | [INTS7, RPL35A, RPS19, SRRT] |  |
|  | GO:0000380 alternative mRNA splicing, via spliceosome | 5.9E-3 | 16.0E-3 | 7.1E-3 | 11.0E-3 | [8, 9, 10, 11, 12] | 9.38 | 3.00 | [HNRNPM, SFPQ, SRSF1] |  |
|  | GO:0000375 RNA splicing, via transesterification reactions | 7.1E-3 | 15.0E-3 | 7.1E-3 | 11.0E-3 | [7, 8, 9] | 4.81 | 5.00 | [DDX39A, HNRNPM, SCAF11, SFPQ, SRSF1] |  |
|  | GO:0000377 RNA splicing, via transesterification reactions with bulged adenosine as nucleophile | 6.8E-3 | 15.0E-3 | 7.1E-3 | 11.0E-3 | [8, 9, 10] | 4.85 | 5.00 | [DDX39A, HNRNPM, SCAF11, SFPQ, SRSF1] |  |
|  | GO:0000398 mRNA splicing, via spliceosome | 6.8E-3 | 15.0E-3 | 7.1E-3 | 11.0E-3 | [7, 8, 9, 10, 11] | 4.85 | 5.00 | [DDX39A, HNRNPM, SCAF11, SFPQ, SRSF1] |  |
|  | GO:1901796 regulation of signal transduction by p53 class mediator | 8.2E-3 | 13.0E-3 | 8.2E-3 | 11.0E-3 | [5, 6, 7] | 8.33 | 3.00 | [AMH, RNF34, SMYD2] |  |
| **Up DEGs in** **MDB vs LNCaP** | | | | | | | | | |  |
|  | GO:0006338 chromatin remodeling | 5.7E-3 | 17.0E-3 | 5.7E-3 | 14.0E-3 | [5] | 6.45 | 4.00 | [CHD1L, CHD4, RBBP4, SMARCD3] |  |
|  | GO:0007548 sex differentiation | 16.0E-3 | 22.0E-3 | 16.0E-3 | 20.0E-3 | [3, 4] | 6.52 | 3.00 | [AMH, CBX2, ROBO2] |  |
|  | GO:0007569 cell aging | 38.0E-3 | 40.0E-3 | 38.0E-3 | 38.0E-3 | [4] | 4.69 | 3.00 | [AMH, LMNA, SMC5] |  |
|  | GO:0032102 negative regulation of response to external stimulus | 7.4E-3 | 14.0E-3 | 2.3E-3 | 11.0E-3 | [3, 4, 5] | 4.76 | 5.00 | [KANK2, NOTCH1, ROBO1, ROBO2, RPS19] |  |
|  | GO:0050922 negative regulation of chemotaxis | 1.2E-3 | 11.0E-3 | 2.3E-3 | 11.0E-3 | [3, 4, 5, 6] | 15.79 | 3.00 | [NOTCH1, ROBO1, ROBO2] |  |
|  | GO:0051962 positive regulation of nervous system development | 4.0E-3 | 13.0E-3 | 2.3E-3 | 11.0E-3 | [3, 4, 5, 6, 7] | 5.49 | 5.00 | [EPHA3, NLGN1, ROBO1, ROBO2, SMARCD3] |  |
|  | GO:0060485 mesenchyme development | 12.0E-3 | 18.0E-3 | 2.3E-3 | 11.0E-3 | [4, 5, 6] | 5.19 | 4.00 | [DAB2IP, LEF1, NOTCH1, TGFBR1] |  |
|  | GO:0010720 positive regulation of cell development | 1.4E-3 | 9.8E-3 | 2.3E-3 | 11.0E-3 | [4, 5, 6, 7] | 4.83 | 7.00 | [EPHA3, LEF1, NLGN1, NOTCH1, ROBO1, ROBO2, SMARCD3] |  |
|  | GO:0010769 regulation of cell morphogenesis involved in differentiation | 2.4E-3 | 13.0E-3 | 2.3E-3 | 11.0E-3 | [5, 6, 7] | 5.08 | 6.00 | [DAB2IP, LEF1, NLGN1, NOTCH1, ROBO1, ROBO2] |  |
|  | GO:0010770 positive regulation of cell morphogenesis involved in differentiation | 7.1E-3 | 14.0E-3 | 2.3E-3 | 11.0E-3 | [4, 5, 6, 7, 8] | 6.06 | 4.00 | [LEF1, NOTCH1, ROBO1, ROBO2] |  |
|  | GO:0014031 mesenchymal cell development | 7.5E-3 | 13.0E-3 | 2.3E-3 | 11.0E-3 | [4, 5, 6, 7, 8] | 5.97 | 4.00 | [DAB2IP, LEF1, NOTCH1, TGFBR1] |  |
|  | GO:0048762 mesenchymal cell differentiation | 7.9E-3 | 13.0E-3 | 2.3E-3 | 11.0E-3 | [5, 6, 7] | 5.88 | 4.00 | [DAB2IP, LEF1, NOTCH1, TGFBR1] |  |
|  | GO:0050769 positive regulation of neurogenesis | 3.3E-3 | 16.0E-3 | 2.3E-3 | 11.0E-3 | [4, 5, 6, 7, 8, 9] | 5.75 | 5.00 | [EPHA3, NLGN1, ROBO1, ROBO2, SMARCD3] |  |
|  | GO:0001837 epithelial to mesenchymal transition | 6.4E-3 | 15.0E-3 | 2.3E-3 | 11.0E-3 | [5, 6, 7, 8, 9] | 6.25 | 4.00 | [DAB2IP, LEF1, NOTCH1, TGFBR1] |  |
|  | GO:0010717 regulation of epithelial to mesenchymal transition | 20.0E-3 | 27.0E-3 | 2.3E-3 | 11.0E-3 | [4, 5, 6, 7, 8, 9, 10] | 6.00 | 3.00 | [DAB2IP, LEF1, NOTCH1] |  |
|  | GO:0045666 positive regulation of neuron differentiation | 10.0E-3 | 15.0E-3 | 2.3E-3 | 11.0E-3 | [5, 6, 7, 8, 9, 10] | 5.48 | 4.00 | [EPHA3, NLGN1, ROBO1, ROBO2] |  |
|  | GO:0048667 cell morphogenesis involved in neuron differentiation | 29.0E-3 | 34.0E-3 | 2.3E-3 | 11.0E-3 | [5, 6, 7, 8, 9, 10] | 5.17 | 3.00 | [NLGN1, ROBO1, ROBO2] |  |
|  | GO:0010975 regulation of neuron projection development | 26.0E-3 | 33.0E-3 | 2.3E-3 | 11.0E-3 | [5, 6, 7, 8, 9, 10, 11] | 4.12 | 4.00 | [EPHA3, NLGN1, ROBO1, ROBO2] |  |
|  | GO:0010976 positive regulation of neuron projection development | 6.4E-3 | 15.0E-3 | 2.3E-3 | 11.0E-3 | [5, 6, 7, 8, 9, 10, 11, 12] | 6.25 | 4.00 | [EPHA3, NLGN1, ROBO1, ROBO2] |  |
|  | GO:0018022 peptidyl-lysine methylation | 35.0E-3 | 38.0E-3 | 35.0E-3 | 39.0E-3 | [5, 6, 7, 8, 9] | 4.84 | 3.00 | [ASH1L, AUTS2, SMYD2] |  |
|  | GO:0031570 DNA integrity checkpoint | 3.8E-3 | 14.0E-3 | 150.0E-6 | 1.5E-3 | [4, 6] | 5.56 | 5.00 | [CDT1, DNA2, INTS7, POLR1B, RBBP8] |  |
|  | GO:0006260 DNA replication | 690.0E-6 | 23.0E-3 | 150.0E-6 | 1.5E-3 | [5, 6, 7] | 5.47 | 7.00 | [CDT1, DNA2, MCM2, POLR1B, PURA, RBBP6, RECQL4] |  |
|  | GO:0006261 DNA-dependent DNA replication | 6.4E-3 | 15.0E-3 | 150.0E-6 | 1.5E-3 | [6, 7, 8] | 6.25 | 4.00 | [CDT1, DNA2, MCM2, PURA] |  |
|  | GO:0031570 DNA integrity checkpoint | 3.8E-3 | 14.0E-3 | 4.8E-3 | 16.0E-3 | [4, 6] | 5.56 | 5.00 | [CDT1, DNA2, INTS7, POLR1B, RBBP8] |  |
|  | GO:0006302 double-strand break repair | 3.8E-3 | 16.0E-3 | 150.0E-6 | 1.5E-3 | [6, 7, 8] | 4.65 | 6.00 | [DNA2, POLR1B, RBBP8, SFPQ, SMC5, XRCC2] |  |
|  | GO:0042254 ribosome biogenesis | 32.0E-3 | 36.0E-3 | 6.9E-3 | 13.0E-3 | [4] | 5.00 | 3.00 | [DHX30, RPL35A, RPS19] |  |
|  | GO:0000725 recombinational repair | 850.0E-6 | 9.6E-3 | 4.8E-3 | 16.0E-3 | [6, 7, 8] | 7.81 | 5.00 | [POLR1B, RBBP8, SFPQ, SMC5, XRCC2] |  |
|  | GO:0006310 DNA recombination | 6.3E-3 | 16.0E-3 | 4.8E-3 | 16.0E-3 | [5, 6, 7] | 4.95 | 5.00 | [POLR1B, RBBP8, SFPQ, SMC5, XRCC2] |  |
|  | GO:0000724 double-strand break repair via homologous recombination | 790.0E-6 | 13.0E-3 | 4.8E-3 | 16.0E-3 | [7, 8, 9] | 7.94 | 5.00 | [POLR1B, RBBP8, SFPQ, SMC5, XRCC2] |  |
|  | GO:0022618 ribonucleoprotein complex assembly | 27.0E-3 | 33.0E-3 | 6.9E-3 | 13.0E-3 | [4, 5, 6] | 4.08 | 4.00 | [DHX30, RPS19, SCAF11, SRSF1] |  |
|  | GO:0034470 ncRNA processing | 24.0E-3 | 32.0E-3 | 6.9E-3 | 13.0E-3 | [6, 7, 8] | 4.21 | 4.00 | [INTS7, RPL35A, RPS19, SRRT] |  |
|  | GO:0000380 alternative mRNA splicing, via spliceosome | 5.9E-3 | 16.0E-3 | 7.1E-3 | 11.0E-3 | [8, 9, 10, 11, 12] | 9.38 | 3.00 | [HNRNPM, SFPQ, SRSF1] |  |
|  | GO:0000375 RNA splicing, via transesterification reactions | 7.1E-3 | 15.0E-3 | 7.1E-3 | 11.0E-3 | [7, 8, 9] | 4.81 | 5.00 | [DDX39A, HNRNPM, SCAF11, SFPQ, SRSF1] |  |
|  | GO:0000377 RNA splicing, via transesterification reactions with bulged adenosine as nucleophile | 6.8E-3 | 15.0E-3 | 7.1E-3 | 11.0E-3 | [8, 9, 10] | 4.85 | 5.00 | [DDX39A, HNRNPM, SCAF11, SFPQ, SRSF1] |  |
|  | GO:0000398 mRNA splicing, via spliceosome | 6.8E-3 | 15.0E-3 | 7.1E-3 | 11.0E-3 | [7, 8, 9, 10, 11] | 4.85 | 5.00 | [DDX39A, HNRNPM, SCAF11, SFPQ, SRSF1] |  |
|  | GO:1901796 regulation of signal transduction by p53 class mediator | 8.2E-3 | 13.0E-3 | 8.2E-3 | 11.0E-3 | [5, 6, 7] | 8.33 | 3.00 | [AMH, RNF34, SMYD2] |  |
| **Down DEGs in** **MDB&PDB vs LNCaP** | | | | | | | | | |  |
|  | GO:0010883 regulation of lipid storage | 25.0E-3 | 39.0E-3 | 5.0E-3 | 10.0E-3 | [3, 4, 5] | 8.00 | 2.00 | [LCP1, OSBPL8] |  |
|  | GO:0030730 sequestering of triglyceride | 5.9E-3 | 17.0E-3 | 5.0E-3 | 10.0E-3 | [4, 5] | 16.67 | 2.00 | [LCP1, OSBPL8] |  |
|  | GO:0010889 regulation of sequestering of triglyceride | 3.3E-3 | 17.0E-3 | 5.0E-3 | 10.0E-3 | [4, 5, 6] | 22.22 | 2.00 | [LCP1, OSBPL8] |  |
|  | GO:0002011 morphogenesis of an epithelial sheet | 23.0E-3 | 39.0E-3 | 5.0E-3 | 10.0E-3 | [5, 6] | 8.33 | 2.00 | [LCP1, TMEFF2] |  |
|  | GO:0061572 actin filament bundle organization | 32.0E-3 | 47.0E-3 | 5.0E-3 | 10.0E-3 | [4, 6, 7] | 4.23 | 3.00 | [LCP1, PPM1E, TMEFF2] |  |
|  | GO:0032760 positive regulation of tumor necrosis factor production | 35.0E-3 | 47.0E-3 | 5.0E-3 | 10.0E-3 | [5, 6, 7] | 6.67 | 2.00 | [THBS1, TWIST1] |  |
|  | GO:0051017 actin filament bundle assembly | 32.0E-3 | 47.0E-3 | 5.0E-3 | 10.0E-3 | [4, 5, 7, 8] | 4.23 | 3.00 | [LCP1, PPM1E, TMEFF2] |  |
|  | GO:0044319 wound healing, spreading of cells | 16.0E-3 | 32.0E-3 | 5.0E-3 | 10.0E-3 | [4, 5, 6, 8, 9] | 10.00 | 2.00 | [LCP1, TMEFF2] |  |
|  | GO:0090504 epiboly | 17.0E-3 | 34.0E-3 | 5.0E-3 | 10.0E-3 | [6, 7] | 9.52 | 2.00 | [LCP1, TMEFF2] |  |
|  | GO:0090505 epiboly involved in wound healing | 16.0E-3 | 32.0E-3 | 5.0E-3 | 10.0E-3 | [5, 7, 8] | 10.00 | 2.00 | [LCP1, TMEFF2] |  |
|  | GO:0034614 cellular response to reactive oxygen species | 21.0E-3 | 39.0E-3 | 21.0E-3 | 21.0E-3 | [5] | 5.00 | 3.00 | [NUP93, PXDN, TSPAN1] |  |
|  | GO:0035970 peptidyl-threonine dephosphorylation | 5.0E-3 | 16.0E-3 | 5.0E-3 | 13.0E-3 | [7, 8] | 18.18 | 2.00 | [PPM1A, PPM1E] |  |
|  | GO:0050954 sensory perception of mechanical stimulus | 11.0E-3 | 24.0E-3 | 11.0E-3 | 12.0E-3 | [5] | 6.38 | 3.00 | [ATP2B4, FAM65B, HOMER2] |  |
|  | GO:0007605 sensory perception of sound | 11.0E-3 | 24.0E-3 | 11.0E-3 | 12.0E-3 | [6] | 6.38 | 3.00 | [ATP2B4, FAM65B, HOMER2] |  |
|  | GO:0051828 entry into other organism involved in symbiotic interaction | 740.0E-6 | 17.0E-3 | 3.0E-3 | 12.0E-3 | [4, 5] | 9.52 | 4.00 | [FUCA2, IFITM2, IFITM3, TMPRSS2] |  |
|  | GO:0035455 response to interferon-alpha | 5.9E-3 | 17.0E-3 | 3.0E-3 | 12.0E-3 | [5] | 16.67 | 2.00 | [IFITM2, IFITM3] |  |
|  | GO:0035456 response to interferon-beta | 11.0E-3 | 24.0E-3 | 3.0E-3 | 12.0E-3 | [5] | 11.76 | 2.00 | [IFITM2, IFITM3] |  |
|  | GO:0044409 entry into host | 740.0E-6 | 17.0E-3 | 3.0E-3 | 12.0E-3 | [5, 6] | 9.52 | 4.00 | [FUCA2, IFITM2, IFITM3, TMPRSS2] |  |
|  | GO:0051806 entry into cell of other organism involved in symbiotic interaction | 740.0E-6 | 17.0E-3 | 3.0E-3 | 12.0E-3 | [5, 6] | 9.52 | 4.00 | [FUCA2, IFITM2, IFITM3, TMPRSS2] |  |
|  | GO:0030260 entry into host cell | 740.0E-6 | 17.0E-3 | 3.0E-3 | 12.0E-3 | [6, 7] | 9.52 | 4.00 | [FUCA2, IFITM2, IFITM3, TMPRSS2] |  |
|  | GO:0046718 viral entry into host cell | 6.1E-3 | 16.0E-3 | 3.0E-3 | 12.0E-3 | [5, 6, 7, 8] | 7.89 | 3.00 | [IFITM2, IFITM3, TMPRSS2] |  |
|  | GO:0046596 regulation of viral entry into host cell | 1.8E-3 | 16.0E-3 | 3.0E-3 | 12.0E-3 | [5, 6, 7, 8, 9] | 12.00 | 3.00 | [IFITM2, IFITM3, TMPRSS2] |  |
|  | GO:0046597 negative regulation of viral entry into host cell | 10.0E-3 | 24.0E-3 | 3.0E-3 | 12.0E-3 | [5, 6, 7, 8, 9, 10] | 12.50 | 2.00 | [IFITM2, IFITM3] |  |
|  | GO:0022617 extracellular matrix disassembly | 2.8E-3 | 16.0E-3 | 6.1E-3 | 9.8E-3 | [4, 5] | 10.34 | 3.00 | [CEL, DPP4, LCP1] |  |
|  | GO:1903053 regulation of extracellular matrix organization | 17.0E-3 | 34.0E-3 | 6.1E-3 | 9.8E-3 | [4, 5] | 9.52 | 2.00 | [CEL, DPP4] |  |
|  | GO:1903054 negative regulation of extracellular matrix organization | 1.9E-3 | 15.0E-3 | 6.1E-3 | 9.8E-3 | [4, 5, 6] | 28.57 | 2.00 | [CEL, DPP4] |  |
|  | GO:0010715 regulation of extracellular matrix disassembly | 5.9E-3 | 17.0E-3 | 6.1E-3 | 9.8E-3 | [5, 6] | 16.67 | 2.00 | [CEL, DPP4] |  |
|  | GO:0010716 negative regulation of extracellular matrix disassembly | 940.0E-6 | 10.0E-3 | 6.1E-3 | 9.8E-3 | [5, 6, 7] | 40.00 | 2.00 | [CEL, DPP4] |  |
|  | GO:0032722 positive regulation of chemokine production | 26.0E-3 | 41.0E-3 | 5.0E-3 | 10.0E-3 | [4, 5, 6] | 7.69 | 2.00 | [LCP1, TWIST1] |  |
|  | GO:1903557 positive regulation of tumor necrosis factor superfamily cytokine production | 37.0E-3 | 49.0E-3 | 5.0E-3 | 10.0E-3 | [4, 5, 6] | 6.45 | 2.00 | [THBS1, TWIST1] |  |
|  | GO:0032722 positive regulation of chemokine production | 26.0E-3 | 41.0E-3 | 29.0E-6 | 230.0E-6 | [4, 5, 6] | 7.69 | 2.00 | [LCP1, TWIST1] |  |
|  | GO:0099024 plasma membrane invagination | 7.0E-3 | 17.0E-3 | 29.0E-6 | 230.0E-6 | [5] | 15.38 | 2.00 | [GULP1, THBS1] |  |
|  | GO:1903557 positive regulation of tumor necrosis factor superfamily cytokine production | 37.0E-3 | 49.0E-3 | 29.0E-6 | 230.0E-6 | [4, 5, 6] | 6.45 | 2.00 | [THBS1, TWIST1] |  |
|  | GO:0009064 glutamine family amino acid metabolic process | 26.0E-3 | 41.0E-3 | 29.0E-6 | 230.0E-6 | [5, 6] | 7.69 | 2.00 | [AADAT, ATP2B4] |  |
|  | GO:0006911 phagocytosis, engulfment | 1.9E-3 | 15.0E-3 | 29.0E-6 | 230.0E-6 | [5, 6, 7] | 28.57 | 2.00 | [GULP1, THBS1] |  |
|  | GO:0032760 positive regulation of tumor necrosis factor production | 35.0E-3 | 47.0E-3 | 29.0E-6 | 230.0E-6 | [5, 6, 7] | 6.67 | 2.00 | [THBS1, TWIST1] |  |
|  | GO:0007263 nitric oxide mediated signal transduction | 4.1E-3 | 17.0E-3 | 29.0E-6 | 230.0E-6 | [6, 7] | 20.00 | 2.00 | [ATP2B4, THBS1] |  |
|  | GO:0010749 regulation of nitric oxide mediated signal transduction | 96.0E-6 | 4.4E-3 | 29.0E-6 | 230.0E-6 | [5, 6, 7, 8] | 100.00 | 2.00 | [ATP2B4, THBS1] |  |
|  | GO:0010751 negative regulation of nitric oxide mediated signal transduction | 96.0E-6 | 4.4E-3 | 29.0E-6 | 230.0E-6 | [5, 6, 7, 8, 9] | 100.00 | 2.00 | [ATP2B4, THBS1] |  |
|  | GO:0015908 fatty acid transport | 23.0E-3 | 39.0E-3 | 29.0E-6 | 230.0E-6 | [5, 6, 8, 9] | 8.33 | 2.00 | [ABCC4, THBS1] |  |
|  | GO:0035767 endothelial cell chemotaxis | 28.0E-3 | 42.0E-3 | 29.0E-6 | 230.0E-6 | [5, 6, 7, 8, 9] | 7.41 | 2.00 | [NRP1, THBS1] |  |
|  | GO:0015718 monocarboxylic acid transport | 26.0E-3 | 41.0E-3 | 29.0E-6 | 230.0E-6 | [7, 8] | 7.69 | 2.00 | [ABCC4, THBS1] |  |
|  | GO:0046068 cGMP metabolic process | 23.0E-3 | 39.0E-3 | 29.0E-6 | 230.0E-6 | [7, 8, 9, 10] | 8.33 | 2.00 | [GUCY1B3, THBS1] |  |
|  | GO:0032410 negative regulation of transporter activity | 7.5E-3 | 18.0E-3 | 6.5E-3 | 8.7E-3 | [3, 4, 5, 6] | 7.32 | 3.00 | [GNB5, KCNRG, STK39] |  |
|  | GO:0034763 negative regulation of transmembrane transport | 810.0E-6 | 12.0E-3 | 6.5E-3 | 8.7E-3 | [3, 4, 5, 6] | 9.30 | 4.00 | [GNB5, KCNRG, STK39, THBS1] |  |
|  | GO:0043271 negative regulation of ion transport | 2.2E-3 | 14.0E-3 | 6.5E-3 | 8.7E-3 | [3, 4, 5, 6] | 7.14 | 4.00 | [GNB5, KCNRG, STK39, THBS1] |  |
|  | GO:0071804 cellular potassium ion transport | 23.0E-3 | 38.0E-3 | 6.5E-3 | 8.7E-3 | [3, 4, 5, 8] | 4.84 | 3.00 | [KCNN2, KCNRG, STK39] |  |
|  | GO:0034766 negative regulation of ion transmembrane transport | 740.0E-6 | 17.0E-3 | 6.5E-3 | 8.7E-3 | [4, 5, 6, 7] | 9.52 | 4.00 | [GNB5, KCNRG, STK39, THBS1] |  |
|  | GO:0032413 negative regulation of ion transmembrane transporter activity | 4.4E-3 | 17.0E-3 | 6.5E-3 | 8.7E-3 | [4, 5, 6, 7, 8] | 8.82 | 3.00 | [GNB5, KCNRG, STK39] |  |
|  | GO:0071805 potassium ion transmembrane transport | 23.0E-3 | 38.0E-3 | 6.5E-3 | 8.7E-3 | [4, 5, 6, 8, 9] | 4.84 | 3.00 | [KCNN2, KCNRG, STK39] |  |
|  | GO:0043267 negative regulation of potassium ion transport | 7.0E-3 | 17.0E-3 | 6.5E-3 | 8.7E-3 | [4, 5, 6, 7, 8, 9] | 15.38 | 2.00 | [KCNRG, STK39] |  |
|  | GO:1901379 regulation of potassium ion transmembrane transport | 3.7E-3 | 17.0E-3 | 6.5E-3 | 8.7E-3 | [3, 4, 5, 6, 7, 8, 9, 10] | 9.38 | 3.00 | [KCNN2, KCNRG, STK39] |  |
|  | GO:1904063 negative regulation of cation transmembrane transport | 4.8E-3 | 17.0E-3 | 6.5E-3 | 8.7E-3 | [5, 6, 7, 8] | 8.57 | 3.00 | [GNB5, KCNRG, STK39] |  |
|  | GO:0006813 potassium ion transport | 34.0E-3 | 47.0E-3 | 6.5E-3 | 8.7E-3 | [7] | 4.17 | 3.00 | [KCNN2, KCNRG, STK39] |  |
|  | GO:0043266 regulation of potassium ion transport | 5.2E-3 | 16.0E-3 | 6.5E-3 | 8.7E-3 | [6, 7, 8] | 8.33 | 3.00 | [KCNN2, KCNRG, STK39] |  |
|  | GO:1901380 negative regulation of potassium ion transmembrane transport | 5.9E-3 | 17.0E-3 | 6.5E-3 | 8.7E-3 | [3, 4, 5, 6, 7, 8, 9, 10, 11] | 16.67 | 2.00 | [KCNRG, STK39] |  |
|  | GO:2001258 negative regulation of cation channel activity | 21.0E-3 | 37.0E-3 | 6.5E-3 | 8.7E-3 | [5, 6, 7, 8, 9] | 8.70 | 2.00 | [GNB5, KCNRG] |  |
|  | GO:1901016 regulation of potassium ion transmembrane transporter activity | 25.0E-3 | 39.0E-3 | 6.5E-3 | 8.7E-3 | [4, 5, 6, 7, 8, 9, 10, 11] | 8.00 | 2.00 | [KCNRG, STK39] |  |
|  | GO:1901017 negative regulation of potassium ion transmembrane transporter activity | 3.3E-3 | 17.0E-3 | 6.5E-3 | 8.7E-3 | [4, 5, 6, 7, 8, 9, 10, 11, 12] | 22.22 | 2.00 | [KCNRG, STK39] |  |

**Supplementary table 4**

| **Down DEPs in** **MDBvsLNCaP** | | | | | | | | | | | | |
| --- | --- | --- | --- | --- | --- | --- | --- | --- | --- | --- | --- | --- |
| **Color Group** | **GOTerm** | **Term p-value** | **Term p-value Corrected with Benjamini-Hochberg** | | **Group p-value** | **Group p-value Corrected with Benjamini-Hochberg** | **GO Levels** | | **% Associated Genes** | **Nr. Genes** | | **Associated Genes Found** |
|  | GO 0034330 cell junction organization | 180.0E-6 | 2.0E-3 | | 180.0E-6 | 400.0E-6 | [3] | | 17.54 | 20.00 | | [ACTN1, CDH1, CLDN3, CTNNB1, DLG1, DSG2, JUP, LAMC1, MARVELD2, MPP7, MYADM, PKP3, PLEC, PTPN23, PTPRJ, RAP1A, RHOA, SLK, VCL, WDR1] |
|  | GO 0034329 cell junction assembly | 2.5E-3 | 14.0E-3 | | 180.0E-6 | 400.0E-6 | [4] | | 16.16 | 16.00 | | [ACTN1, CLDN3, CTNNB1, DLG1, JUP, LAMC1, MARVELD2, MPP7, PKP3, PLEC, PTPRJ, RAP1A, RHOA, SLK, VCL, WDR1] |
|  | GO 0045216 cell-cell junction organization | 1.5E-3 | 10.0E-3 | | 180.0E-6 | 400.0E-6 | [4] | | 16.50 | 17.00 | | [ACTN1, CDH1, CLDN3, CTNNB1, DLG1, DSG2, JUP, MARVELD2, MPP7, MYADM, PKP3, PTPN23, PTPRJ, RHOA, SLK, VCL, WDR1] |
|  | GO 0007043 cell-cell junction assembly | 3.2E-3 | 18.0E-3 | | 180.0E-6 | 400.0E-6 | [5] | | 20.93 | 9.00 | | [CLDN3, DLG1, JUP, MARVELD2, MPP7, PKP3, RHOA, VCL, WDR1] |
|  | GO 0043297 apical junction assembly | 7.1E-3 | 34.0E-3 | | 180.0E-6 | 400.0E-6 | [6] | | 21.88 | 7.00 | | [CLDN3, DLG1, MARVELD2, MPP7, RHOA, VCL, WDR1] |
|  | GO 0007165 signal transduction | 560.0E-6 | 4.9E-3 | | 270.0E-9 | 1.3E-6 | [3, 4] | | 5.58 | 110.00 | | [AAK1, ACTN4, AGR2, AHCYL1, ARHGDIA, ARL6IP5, ATP2A2, ATP2B4, BAG6, BAX, BCAP31, BRD4, CC2D1A, CDC37, CDC5L, CDK5RAP3, CIB1, CNOT1, CTNNB1, CTSH, CYFIP1, DDRGK1, DLG1, DNAJA3, DNM1L, DNMT1, EEF1D, EGFR, EPHB3, ERBB2, ERP29, ETFA, ETFDH, EZR, FAF1, FARP2, FASN, FLOT2, FN1, GARS, GNAI3, GRB2, HSPA1A, HTRA2, ILK, IMPA1, ITPR1, KMT2D, LAMP1, LAMTOR1, LARP1, MAP2K2, MARK2, MFN2, MMAB, MYADM, MYO6, NAMPT, NDRG1, NUP62, OPA1, PAK2, PARK7, PEA15, PHB, PHIP, PPIF, PRKD1, PRMT5, PRPF19, PTPN11, PTPRJ, RAB7A, RAP1A, RBFOX2, RHOA, RHOT1, RNF213, RNMT, RPL10, RPS3, RPS6, RPSA, RRAS, SDCBP, SFN, SHC1, SLC35B2, SLC44A2, SLC9A1, SMARCB1, SOD2, SRI, STRN, SYNJ2BP, TKFC, TMBIM1, TOLLIP, TOM1L1, TOM1L2, TP53BP1, TPD52L1, TRIM25, TSG101, UBE2V1, UFL1, VIMP, WFS1, WNK1, ZC3HAV1] |
|  | GO 0031325 positive regulation of cellular metabolic process | 72.0E-6 | 1.0E-3 | | 270.0E-9 | 1.3E-6 | [3, 4, 5] | | 5.11 | 86.00 | | [ABHD14B, ANKLE2, ARL6IP5, ATP2B4, BAX, BCAP31, BRD4, C14orf166, CCT2, CCT4, CCT5, CCT6A, CCT7, CCT8, CD276, CDH1, CDK5RAP3, CENPE, CNOT1, CTNNB1, CTSH, DDRGK1, DNAJA3, DNAJB2, DNMT1, EEF2, EGFR, ERBB2, ERP29, ETFA, ETFDH, EZR, GARS, GNL3, GRB2, HSPA1A, HTRA2, ILK, IST1, KMT2D, MAP2K2, MLYCD, MYDGF, MYO6, NAMPT, NBN, NCOA6, NSF, NUP62, PACSIN3, PAK2, PARK7, PELP1, PHB, PHIP, PNPLA2, PNPT1, PPP2CA, PRKD1, QARS, RAB3GAP2, REST, RHOA, RNMT, RPRD1B, RPS3, RPS4X, SCRIB, SDCBP, SHC1, SIN3A, SLC9A1, SMARCB1, SMARCC2, STRN3, SUPV3L1, TBC1D5, TCP1, TFAM, TOM1L1, TP53BP1, TPD52L1, VCP, WFS1, YTHDF2, ZC3HAV1] |
|  | GO 0016310 phosphorylation | 9.9E-3 | 42.0E-3 | | 270.0E-9 | 1.3E-6 | [5] | | 5.36 | 55.00 | | [AAK1, ADAR, ADCK3, AK1, ALDOA, ANKLE2, ARL6IP5, ATP2B4, BCCIP, BRD4, CDK11B, CDK5RAP3, CENPE, COA6, CTSH, DDRGK1, DLG1, DNAJA3, DNAJC3, EGFR, EPHB3, ERBB2, ERP29, ETFA, ETFDH, EZR, FN1, ILK, MAP2K2, MARK2, MMAB, MYADM, NBN, PAK2, PARK7, PFKP, PHB, PPP2CA, PRKACB, PRKD1, PTPRJ, RHOA, RPS3, RRAS, SDCBP, SHC1, SLK, SYNJ2BP, TKFC, TOLLIP, TOM1L1, TPD52L1, TSG101, VCP, WNK1] |
|  | GO 0019220 regulation of phosphate metabolic process | 9.2E-3 | 41.0E-3 | | 270.0E-9 | 1.3E-6 | [5, 6] | | 5.16 | 46.00 | | [ADAR, ANKLE2, ARFGEF3, ARL6IP5, ATP2B4, BCCIP, BRD4, CDK5RAP3, CENPE, DDRGK1, DLG1, DNAJA3, DNAJC3, EGFR, ERBB2, ERP29, ETFA, ETFDH, EZR, FN1, ILK, MAP2K2, ME2, MYADM, MYO1D, NBN, PAK2, PARK7, PHB, PPP2CA, PRKD1, PTPRJ, RHOA, RPS3, RRAS, RRP1B, SDCBP, SHC1, SLC27A1, SYNJ2BP, TOM1L1, TPD52L1, TSG101, VCP, WNK1, YWHAE] |
|  | GO 0045937 positive regulation of phosphate metabolic process | 3.4E-3 | 18.0E-3 | | 270.0E-9 | 1.3E-6 | [5, 6, 7] | | 4.36 | 25.00 | | [ANKLE2, ARL6IP5, ATP2B4, CENPE, DDRGK1, EGFR, ERBB2, ERP29, ETFA, ETFDH, ILK, MAP2K2, NBN, PAK2, PARK7, PHB, PPP2CA, PRKD1, RHOA, RPS3, SDCBP, SHC1, TOM1L1, TPD52L1, VCP] |
|  | GO 0006468 protein phosphorylation | 2.5E-3 | 14.0E-3 | | 270.0E-9 | 1.3E-6 | [6, 7] | | 4.93 | 46.00 | | [AAK1, ADAR, ARL6IP5, ATP2B4, BCCIP, BRD4, CDK11B, CDK5RAP3, CENPE, CTSH, DDRGK1, DLG1, DNAJA3, DNAJC3, EGFR, EPHB3, ERBB2, ERP29, ETFA, ETFDH, EZR, FN1, ILK, MAP2K2, MARK2, MMAB, MYADM, NBN, PAK2, PARK7, PHB, PPP2CA, PRKACB, PRKD1, PTPRJ, RHOA, RPS3, RRAS, SDCBP, SHC1, SLK, SYNJ2BP, TOM1L1, TPD52L1, TSG101, WNK1] |
|  | GO 0010256 endomembrane system organization | 9.0E-6 | 230.0E-6 | | 200.0E-9 | 1.2E-6 | [3] | | 14.68 | 43.00 | | [ACSL3, AGR2, ANKLE2, ATP1B3, BAG6, BNIP1, CDH1, CIB1, COG2, COG3, COG4, DLG1, DNM2, EZR, FLOT2, GBF1, GCC2, GOLGA5, HOOK1, JUP, LEMD2, LMAN1, MACF1, MYADM, MYO18A, MYO5A, PACSIN2, PACSIN3, PKP3, PPFIA1, PRKD1, PRMT5, RAB10, RAB2A, RAB3GAP2, RTN4, SEC61A1, STX18, STXBP1, TMBIM1, TMED5, VTI1B, ZW10] |
|  | GO 0007030 Golgi organization | 71.0E-6 | 1.0E-3 | | 200.0E-9 | 1.2E-6 | [4] | | 22.39 | 15.00 | | [COG2, COG3, COG4, GBF1, GCC2, GOLGA5, LMAN1, MYO18A, MYO5A, PRKD1, PRMT5, RAB2A, STX18, TMED5, ZW10] |
|  | GO 0016192 vesicle-mediated transport | 96.0E-6 | 1.2E-3 | | 200.0E-9 | 1.2E-6 | [4] | | 12.26 | 57.00 | | [AAK1, AP1G1, ATXN2, BET1L, CHMP1A, COG2, COG3, COG4, DCTN1, DNM2, EEA1, EGFR, EHD4, EPS15, EZR, GBF1, GCC2, GOLGA5, GOSR1, GRB2, HOOK1, LAMP1, LMAN1, MYO18A, MYO5A, MYO6, PACSIN2, PDCD6IP, PIK3C2A, PPT1, PTPN23, RAB11B, RAB2A, RAB7A, RAB9A, RALA, SCYL1, SDCBP, SNX1, SNX2, SNX27, SNX5, SPAG9, STEAP2, STX18, STXBP1, STXBP2, STXBP3, TBC1D5, TMEM115, TOR1A, TSG101, VAMP7, VPS33A, VPS35, VTA1, ZW10] |
|  | GO 0048193 Golgi vesicle transport | 50.0E-6 | 770.0E-6 | | 200.0E-9 | 1.2E-6 | [5] | | 19.23 | 20.00 | | [AP1G1, BET1L, COG2, COG3, COG4, EPS15, GBF1, GCC2, GOLGA5, LAMP1, LMAN1, MYO18A, MYO5A, RAB2A, SCYL1, SNX1, STEAP2, STX18, TMEM115, ZW10] |
|  | GO 0090161 Golgi ribbon formation | 6.1E-3 | 30.0E-3 | | 200.0E-9 | 1.2E-6 | [5] | | 36.36 | 4.00 | | [GCC2, MYO18A, PRMT5, TMED5] |
|  | GO 0006890 retrograde vesicle-mediated transport, Golgi to ER | 2.3E-3 | 14.0E-3 | | 200.0E-9 | 1.2E-6 | [6] | | 30.00 | 6.00 | | [BET1L, COG3, COG4, GBF1, SCYL1, TMEM115] |
|  | GO 0051186 cofactor metabolic process | 1.3E-3 | 9.6E-3 | | 1.0E-3 | 1.7E-3 | [3] | | 15.11 | 21.00 | | [ABCB6, ACAT1, ACLY, ACOT1, ADCK3, ALDOA, BLVRB, CBR1, COASY, COQ3, HSCB, ME2, MLYCD, NFS1, PDHA1, PDHB, PFKP, PGD, PPT1, PRDX5, VCP] |
|  | GO 0044272 sulfur compound biosynthetic process | 11.0E-3 | 46.0E-3 | | 1.0E-3 | 1.7E-3 | [4] | | 18.60 | 8.00 | | [ACAT1, ACLY, CTNNB1, HAGH, MGST2, MLYCD, PDHA1, PDHB] |
|  | GO 0051188 cofactor biosynthetic process | 760.0E-6 | 6.1E-3 | | 1.0E-3 | 1.7E-3 | [4] | | 22.00 | 11.00 | | [ABCB6, ACAT1, ACLY, ADCK3, COASY, COQ3, HSCB, MLYCD, NFS1, PDHA1, PDHB] |
|  | GO 0006637 acyl-CoA metabolic process | 9.5E-3 | 42.0E-3 | | 1.0E-3 | 1.7E-3 | [4, 5] | | 23.08 | 6.00 | | [ACAT1, ACLY, ACOT1, MLYCD, PDHA1, PDHB] |
|  | GO 0009108 coenzyme biosynthetic process | 1.6E-3 | 10.0E-3 | | 1.0E-3 | 1.7E-3 | [5] | | 25.00 | 8.00 | | [ACAT1, ACLY, ADCK3, COASY, COQ3, MLYCD, PDHA1, PDHB] |
|  | GO 0009060 aerobic respiration | 2.6E-3 | 15.0E-3 | | 1.0E-3 | 1.7E-3 | [5, 6] | | 44.44 | 4.00 | | [ACO2, PDHA1, PDHB, VCP] |
|  | GO 0006085 acetyl-CoA biosynthetic process | 190.0E-6 | 2.1E-3 | | 1.0E-3 | 1.7E-3 | [6, 7] | | 55.56 | 5.00 | | [ACAT1, ACLY, MLYCD, PDHA1, PDHB] |
|  | GO 0050793 regulation of developmental process | 6.4E-3 | 32.0E-3 | | 640.0E-6 | 1.2E-3 | [2, 3] | | 5.03 | 43.00 | | [ACTN4, ALDOA, CAPZB, CIB1, CNOT1, CTNNB1, DLG1, DNM1L, EEF1E1, EZR, FITM2, FLOT2, MACF1, MAP4, MAPT, MARK2, MYADM, MYDGF, MYH9, NEDD4L, PDCD4, PDZD8, PHIP, PNPT1, PPP2CA, PRKD1, PRMT1, REST, RHOA, RNH1, RPL10, RRAS, RTN4, SDCBP, SEPT9, SHTN1, SPEN, SYNJ2BP, TBC1D30, TBC1D5, TRIOBP, WARS, YWHAG] |
|  | GO 0051239 regulation of multicellular organismal process | 39.0E-6 | 670.0E-6 | | 640.0E-6 | 1.2E-3 | [2, 3] | | 4.34 | 45.00 | | [ATP2A2, ATP2B4, CD276, CIB1, CNOT1, CTNNB1, DSG2, DSP, EZR, FN1, HSPA1A, JUP, MACF1, MAPT, MARK2, MTPN, MYADM, MYDGF, NDUFA2, NEDD4L, PARK7, PFN1, PPP2CA, PRDX5, PRKD1, PRMT1, PTPN23, REST, RHOA, RNH1, RNMT, RPSA, RRAS, RTN4, SDCBP, SHTN1, SLC9A1, SPEN, SRI, SYNJ2BP, TKFC, VAMP7, WARS, YWHAG, ZC3HAV1] |
|  | GO 0007275 multicellular organism development | 3.3E-3 | 18.0E-3 | | 640.0E-6 | 1.2E-3 | [3] | | 5.38 | 69.00 | | [ABCB6, ACTN1, ALDH3A2, ALDH5A1, ANKLE2, ATP2B4, ATP5B, ATP5F1, ATXN10, CIB1, CKB, CNP, CTNNB1, CYFIP1, DDX1, DSG2, DSP, EIF2B4, EZR, FARP2, FLVCR1, FN1, IDE, IST1, LAMA3, LEMD2, LIG3, MACF1, MAP1S, MAPT, MARK2, MYADM, MYDGF, MYH14, MYH9, NAMPT, NCOA6, NEDD4L, PAFAH1B1, PIR, PPP2CA, PPT1, PRKD1, PRMT1, PTPN11, QARS, REST, RHOA, RNF213, RNH1, RNMT, RPL10, RPS4X, RRAS, RTN1, RTN4, SCRIB, SDCBP, SHTN1, SPEN, SYNJ2BP, TOR1A, TP53BP1, TPD52, TPP1, WARS, WFS1, YWHAE, YWHAG] |
|  | GO 0051094 positive regulation of developmental process | 10.0E-3 | 45.0E-3 | | 640.0E-6 | 1.2E-3 | [2, 3, 4] | | 4.27 | 19.00 | | [CIB1, CTNNB1, DNM1L, EEF1E1, MACF1, MAPT, MARK2, MYADM, MYDGF, NEDD4L, PRKD1, PRMT1, RHOA, RRAS, SDCBP, SEPT9, SHTN1, SPEN, TRIOBP] |
|  | GO 0051240 positive regulation of multicellular organismal process | 2.0E-3 | 12.0E-3 | | 640.0E-6 | 1.2E-3 | [2, 3, 4] | | 4.25 | 26.00 | | [CD276, CTNNB1, HSPA1A, MACF1, MAPT, MARK2, MTPN, MYDGF, NDUFA2, NEDD4L, PARK7, PFN1, PRDX5, PRKD1, PRMT1, RHOA, RNMT, RPSA, RRAS, SDCBP, SHTN1, SLC9A1, SPEN, TKFC, VAMP7, ZC3HAV1] |
|  | GO 0048731 system development | 1.0E-3 | 7.8E-3 | | 640.0E-6 | 1.2E-3 | [3, 4] | | 5.06 | 60.00 | | [ABCB6, ACTN1, ALDH3A2, ALDH5A1, ANKLE2, ATP2B4, ATP5B, ATP5F1, ATXN10, CIB1, CKB, CNP, CTNNB1, CYFIP1, DSG2, DSP, EIF2B4, EZR, FARP2, FLVCR1, LEMD2, LIG3, MACF1, MAP1S, MAPT, MARK2, MYDGF, MYH14, MYH9, NAMPT, NCOA6, NEDD4L, PAFAH1B1, PIR, PPP2CA, PPT1, PRKD1, PRMT1, PTPN11, QARS, REST, RHOA, RNF213, RNH1, RPL10, RRAS, RTN1, RTN4, SCRIB, SDCBP, SHTN1, SPEN, SYNJ2BP, TOR1A, TPD52, TPP1, WARS, WFS1, YWHAE, YWHAG] |
|  | GO 0045595 regulation of cell differentiation | 8.5E-3 | 39.0E-3 | | 640.0E-6 | 1.2E-3 | [3, 4, 5] | | 4.42 | 23.00 | | [ACTN4, CIB1, CTNNB1, FLOT2, MACF1, MAPT, MARK2, MYADM, MYDGF, NEDD4L, PDCD4, PPP2CA, PRKD1, PRMT1, REST, RHOA, RPL10, RTN4, SDCBP, SHTN1, SPEN, TRIOBP, YWHAG] |
|  | GO 0022607 cellular component assembly | 11.0E-3 | 46.0E-3 | | 5.2E-3 | 6.4E-3 | [3] | | 9.10 | 109.00 | | [ABCC4, ACAD9, ACSL3, ACTN1, ACTR3, ADAR, ALDH5A1, ATXN2, ATXN2L, BAX, CAND1, CCT2, CHMP1A, CLDN3, CNOT1, COA6, COG4, CTNNB1, CYFIP1, DDX6, DHRS4, DHX30, DLG1, DNAJB2, DNM1L, EHD4, EIF2A, EIF3A, EIF6, EPS15, EZR, FAF1, FARSA, FARSB, FASTKD2, FLOT2, FN1, FOXRED1, GARS, GEMIN5, GRHPR, HSCB, HSP90AA1, HSPA1A, HSPA4, IDE, JUP, LAMC1, MAP4, MAPT, MARVELD2, MFN2, MMAB, MPP7, MTPN, MYADM, NACC1, NDUFAF4, NDUFS8, NFS1, NUDT21, NUP153, PACSIN2, PDCD6IP, PFN1, PKP3, PLEC, PNPT1, PPFIA1, PQBP1, PRMT5, PRPF19, PTPN11, PTPRJ, RAB3GAP2, RAB7A, RALA, RAP1A, RBM5, RHOA, RNF213, RPS3, RTN4, SAMM50, SDCBP, SEPT9, SLK, SMIM20, SNAP29, SNRPB, SNX2, SOD2, STX12, STX18, TBC1D30, TBC1D5, TIMM21, TNPO1, TOR1A, TP53BP1, TSG101, TTC19, UXS1, VCL, VCP, VTA1, WASF2, WDR1, ZW10] |
|  | GO 0022613 ribonucleoprotein complex biogenesis | 1.6E-3 | 10.0E-3 | | 5.2E-3 | 6.4E-3 | [3] | | 14.79 | 21.00 | | [ADAR, ATXN2, ATXN2L, CNOT1, DDX6, DHX30, EIF2A, EIF3A, EIF6, FASTKD2, GEMIN5, MMAB, NSUN4, PQBP1, PRMT5, PRPF19, RBM5, RPL7, RPS28, RPS6, SNRPB] |
|  | GO 0065003 macromolecular complex assembly | 6.5E-3 | 31.0E-3 | | 5.2E-3 | 6.4E-3 | [4] | | 9.92 | 71.00 | | [ACAD9, ACSL3, ACTR3, ADAR, ALDH5A1, ATXN2, ATXN2L, BAX, CAND1, CCT2, CLDN3, CNOT1, COA6, COG4, CYFIP1, DDX6, DHRS4, DHX30, DNM1L, EHD4, EIF2A, EIF3A, EIF6, EPS15, FAF1, FARSA, FARSB, FASTKD2, FN1, FOXRED1, GARS, GEMIN5, GRHPR, HSP90AA1, HSPA4, IDE, LAMC1, MAPT, MMAB, MPP7, MTPN, MYADM, NACC1, NDUFAF4, NDUFS8, NUDT21, NUP153, PFN1, PNPT1, PQBP1, PRMT5, PRPF19, PTPN11, RBM5, RNF213, RPS3, RTN4, SAMM50, SEPT9, SMIM20, SNRPB, SNX2, SOD2, TIMM21, TNPO1, TOR1A, TP53BP1, TTC19, UXS1, VCP, ZW10] |
|  | GO 0071826 ribonucleoprotein complex subunit organization | 120.0E-6 | 1.4E-3 | | 5.2E-3 | 6.4E-3 | [4] | | 18.63 | 19.00 | | [ADAR, ATXN2, ATXN2L, CNOT1, DDX6, DHX30, EIF2A, EIF3A, EIF6, FASTKD2, GEMIN5, GFM2, MMAB, MRRF, PQBP1, PRMT5, PRPF19, RBM5, SNRPB] |
|  | GO 0022618 ribonucleoprotein complex assembly | 640.0E-6 | 5.4E-3 | | 5.2E-3 | 6.4E-3 | [4, 5, 6] | | 17.35 | 17.00 | | [ADAR, ATXN2, ATXN2L, CNOT1, DDX6, DHX30, EIF2A, EIF3A, EIF6, FASTKD2, GEMIN5, MMAB, PQBP1, PRMT5, PRPF19, RBM5, SNRPB] |
|  | GO 0034622 cellular macromolecular complex assembly | 7.0E-3 | 33.0E-3 | | 5.2E-3 | 6.4E-3 | [5] | | 10.91 | 43.00 | | [ACAD9, ACTR3, ADAR, ATXN2, ATXN2L, CAND1, CCT2, CNOT1, COA6, COG4, CYFIP1, DDX6, DHX30, DNM1L, EIF2A, EIF3A, EIF6, EPS15, FASTKD2, FOXRED1, GARS, GEMIN5, HSP90AA1, HSPA4, MAPT, MMAB, MTPN, MYADM, NDUFAF4, NDUFS8, NUP153, PFN1, PQBP1, PRMT5, PRPF19, RBM5, RPS3, RTN4, SAMM50, SMIM20, SNRPB, TIMM21, TTC19] |
|  | GO 0006091 generation of precursor metabolites and energy | 9.7E-3 | 42.0E-3 | | 110.0E-6 | 280.0E-6 | [3] | | 15.29 | 13.00 | | [ACO2, ALDOA, COA6, CROT, ETFDH, NDUFS1, PARK7, PDHA1, PDHB, PFKP, STBD1, TRAP1, VCP] |
|  | GO 0055114 oxidation-reduction process | 46.0E-6 | 740.0E-6 | | 110.0E-6 | 280.0E-6 | [3] | | 15.87 | 30.00 | | [ABCD3, ACAA1, ACADM, ACO2, ALDH3A2, BLVRA, CBR1, COA6, CROT, DHRS4, ETFA, ETFDH, GCDH, GRHPR, LONP2, MARC1, MLYCD, NDUFS1, PARK7, PDHA1, PDHB, PECR, PGD, PRDX5, SLC25A17, SLC27A2, SQRDL, STBD1, TRAP1, VCP] |
|  | GO 0032787 monocarboxylic acid metabolic process | 620.0E-6 | 5.2E-3 | | 110.0E-6 | 280.0E-6 | [6, 7] | | 13.94 | 29.00 | | [ABCD3, ACAA1, ACADM, ACOT1, ACSF3, ALDOA, AMACR, CROT, DECR2, ECI2, ELOVL5, ETFA, ETFDH, FAAH, GCDH, HOGA1, LONP2, LTA4H, MCAT, MGST2, MLYCD, PARK7, PDHA1, PDHB, PFKP, SCD, SLC25A17, SLC27A2, TECR] |
|  | GO 0019395 fatty acid oxidation | 55.0E-6 | 820.0E-6 | | 110.0E-6 | 280.0E-6 | [5, 6, 7, 8, 9] | | 28.95 | 11.00 | | [ABCD3, ACAA1, ACADM, CROT, ETFA, ETFDH, GCDH, LONP2, MLYCD, SLC25A17, SLC27A2] |
|  | GO 0072329 monocarboxylic acid catabolic process | 2.7E-6 | 100.0E-6 | | 110.0E-6 | 280.0E-6 | [6, 7, 8] | | 32.50 | 13.00 | | [ABCD3, ACAA1, ACADM, CROT, ECI2, ETFA, ETFDH, FAAH, GCDH, HOGA1, LONP2, SLC25A17, SLC27A2] |
|  | GO 0043436 oxoacid metabolic process | 920.0E-9 | 45.0E-6 | | 4.4E-6 | 18.0E-6 | [4, 5] | | 15.26 | 47.00 | | [ABCD3, ACAA1, ACADM, ACLY, ACO2, ACOT1, ACSF3, ALDH5A1, ALDH6A1, ALDOA, AMACR, ATP2B4, CARS, CROT, DECR2, ECI2, ELOVL5, ETFA, ETFDH, FAAH, FARSA, FARSB, GATB, GCDH, GOT1, HARS2, HOGA1, IDH1, KARS, LARS2, LONP2, LTA4H, MARC1, MARS2, MCAT, MGST2, MLYCD, MTHFD1, PARK7, PDHA1, PDHB, PFKP, QARS, SCD, SLC25A17, SLC27A2, TECR] |
|  | GO 0006520 cellular amino acid metabolic process | 1.3E-3 | 9.6E-3 | | 4.4E-6 | 18.0E-6 | [3, 4, 6, 7] | | 17.95 | 14.00 | | [ALDH5A1, ALDH6A1, ATP2B4, CARS, FARSA, FARSB, GATB, GOT1, HARS2, KARS, LARS2, MARS2, MTHFD1, QARS] |
|  | GO 0044248 cellular catabolic process | 89.0E-6 | 1.2E-3 | | 11.0E-6 | 37.0E-6 | [3] | | 11.30 | 75.00 | | [ABCD3, ABHD12, ABHD16A, ABHD6, ACAA1, ACADM, ACAT1, ACSF3, ALDH5A1, ALDH6A1, ATP2B4, BAX, BCAP31, BCKDHA, BLVRB, BNIP3L, CDK5RAP3, CLPX, CNOT1, CPQ, CROT, CTSH, DDRGK1, DNAJB2, ECI2, ETFA, ETFDH, EZR, FAAH, GCDH, GOT1, HOGA1, HTRA2, IDE, KIAA0368, LONP2, LTA4H, MAN1B1, MLYCD, NAGA, NEDD4L, OS9, PACSIN3, PARK7, PARN, PCNP, PCYOX1, PDCD6IP, PNPLA2, PNPT1, PON2, PRDX5, PRPF19, PSMC2, PTPN23, RAB7A, RNF213, SDCBP, SLC25A17, SLC27A2, SORD, STBD1, SUPV3L1, TMUB1, TPP1, TRIM25, TSG101, UBA6, UBXN1, UCHL5, UFL1, VCP, VIMP, WFS1, ZC3HAV1] |
|  | GO 0044712 single-organism catabolic process | 2.7E-6 | 100.0E-6 | | 11.0E-6 | 37.0E-6 | [3] | | 14.98 | 46.00 | | [ABCD3, ABHD12, ABHD16A, ABHD6, ACAA1, ACADM, ACAT1, ACSF3, ALDH5A1, ALDH6A1, ALDOA, ATP2B4, BAG6, BCAP31, BCKDHA, BLVRB, CROT, DNAJB2, ECI2, ETFA, ETFDH, FAAH, GCDH, GOT1, HOGA1, IMPA1, KIAA0368, LONP2, MAN1B1, NAGA, NPLOC4, OS9, PFKP, PNPLA2, PPT1, SLC25A17, SLC27A2, SORD, STBD1, TMUB1, TRIM25, UBAC2, UBXN1, VCP, VIMP, WFS1] |
|  | GO 1901575 organic substance catabolic process | 32.0E-6 | 620.0E-6 | | 11.0E-6 | 37.0E-6 | [3] | | 11.29 | 85.00 | | [ABCD3, ABHD12, ABHD16A, ABHD6, ACAA1, ACADM, ACAT1, ACSF3, ALDH5A1, ALDH6A1, ALDOA, ATP2B4, BAG6, BAX, BCAP31, BCKDHA, BLVRB, BNIP3L, CDK5RAP3, CLPX, CNOT1, CPQ, CROT, CTSH, DDRGK1, DNAJB2, ECI2, EGFR, ETFA, ETFDH, EZR, FAAH, FAF1, GCDH, GOT1, HOGA1, HTRA2, IDE, IMPA1, KIAA0368, LONP2, LTA4H, MAN1B1, MLYCD, NAGA, NEDD4L, NPLOC4, NSF, OS9, PACSIN3, PARK7, PARN, PCNP, PCYOX1, PDCD6IP, PFKP, PHB, PNPLA2, PNPT1, PPT1, PRPF19, PSMC2, PTPN23, RAB7A, RNF213, SDCBP, SLC25A17, SLC27A2, SORD, STBD1, SUPV3L1, TMUB1, TPP1, TRIM24, TRIM25, TSG101, UBA6, UBAC2, UBXN1, UCHL5, UFL1, VCP, VIMP, WFS1, ZC3HAV1] |
|  | GO 0034660 ncRNA metabolic process | 4.0E-3 | 20.0E-3 | | 4.4E-6 | 18.0E-6 | [5, 6, 7] | | 13.82 | 21.00 | | [ADAR, C14orf166, CARS, DDX1, FARSA, FARSB, GATB, HARS2, KARS, LARS2, MARS2, NSUN2, NSUN4, PNPT1, QARS, RPL7, RPS28, RPS6, RTCB, SMARCB1, TRMU] |
|  | GO 0006399 tRNA metabolic process | 6.3E-6 | 170.0E-6 | | 4.4E-6 | 18.0E-6 | [6, 7, 8] | | 28.57 | 14.00 | | [C14orf166, CARS, DDX1, FARSA, FARSB, GATB, HARS2, KARS, LARS2, MARS2, NSUN2, QARS, RTCB, TRMU] |
|  | GO 0043039 tRNA aminoacylation | 1.4E-6 | 68.0E-6 | | 4.4E-6 | 18.0E-6 | [5, 6, 7, 8, 9] | | 50.00 | 9.00 | | [CARS, FARSA, FARSB, GATB, HARS2, KARS, LARS2, MARS2, QARS] |
|  | GO 1901564 organonitrogen compound metabolic process | 11.0E-6 | 280.0E-6 | | 6.1E-9 | 83.0E-9 | [3] | | 11.60 | 84.00 | | [ABCB6, ACADM, ACAT1, ADAR, AK3, ALDH5A1, ALDH6A1, ALDOA, APEH, ATP2B4, ATP5B, ATP5O, BCKDHA, BLVRB, CAD, CARS, CNOT1, COA6, COASY, CPQ, CRAT, CTSH, DDAH1, DDAH2, DNAJC3, EEF2, EGFR, EIF2A, EIF2B4, EIF2S3, EIF3A, EIF3B, EIF3C, EIF3D, EIF3F, EIF3G, EIF4G2, EIF5, EIF5B, EIF6, ERBB2, FARSA, FARSB, FXR1, GATB, GFM1, GFM2, GNAI3, GOT1, GUK1, HAGH, HARS2, HOGA1, HSPA1A, IDE, KARS, LARP1, LARS2, LTA4H, MACROD1, MAP2K2, MARS2, ME2, MGST2, MTHFD1, MUT, MYCBP2, NDUFS1, OLA1, PARK7, PFKP, PGD, PRDX5, PTCD3, QARS, QTRT1, RPS3, RPS4X, SLC27A1, SNIP1, TPP1, TRAP1, VCP, YTHDF2] |
|  | GO 1901566 organonitrogen compound biosynthetic process | 170.0E-6 | 1.9E-3 | | 6.1E-9 | 83.0E-9 | [4] | | 12.14 | 55.00 | | [ABCB6, ACADM, ACAT1, ADAR, ALDOA, ATP2B4, ATP5B, ATP5O, CAD, CARS, CNOT1, COASY, DNAJC3, EEF2, EGFR, EIF2A, EIF2B4, EIF2S3, EIF3A, EIF3B, EIF3C, EIF3D, EIF3F, EIF3G, EIF4G2, EIF5, EIF5B, EIF6, ERBB2, FARSA, FARSB, FXR1, GATB, GFM1, GFM2, HAGH, HARS2, KARS, LARP1, LARS2, MAP2K2, MARS2, MGST2, MTHFD1, PARK7, PTCD3, QARS, QTRT1, RPS3, RPS4X, SLC27A1, SNIP1, TRAP1, VCP, YTHDF2] |
|  | GO 0006518 peptide metabolic process | 580.0E-12 | 460.0E-9 | | 6.1E-9 | 83.0E-9 | [4, 5] | | 18.63 | 49.00 | | [ADAR, APEH, CARS, CNOT1, CPQ, CTSH, DNAJC3, EEF2, EGFR, EIF2A, EIF2B4, EIF2S3, EIF3A, EIF3B, EIF3C, EIF3D, EIF3F, EIF3G, EIF4G2, EIF5, EIF5B, EIF6, ERBB2, FARSA, FARSB, FXR1, GATB, GFM1, GFM2, HAGH, HARS2, IDE, KARS, LARP1, LARS2, LTA4H, MAP2K2, MARS2, MGST2, MYCBP2, PARK7, PTCD3, QARS, RPS3, RPS4X, SNIP1, TPP1, TRAP1, YTHDF2] |
|  | GO 0043043 peptide biosynthetic process | 5.8E-9 | 2.3E-6 | | 6.1E-9 | 83.0E-9 | [5, 6] | | 19.25 | 41.00 | | [ADAR, CARS, CNOT1, DNAJC3, EEF2, EGFR, EIF2A, EIF2B4, EIF2S3, EIF3A, EIF3B, EIF3C, EIF3D, EIF3F, EIF3G, EIF4G2, EIF5, EIF5B, EIF6, ERBB2, FARSA, FARSB, FXR1, GATB, GFM1, GFM2, HAGH, HARS2, KARS, LARP1, LARS2, MAP2K2, MARS2, MGST2, PTCD3, QARS, RPS3, RPS4X, SNIP1, TRAP1, YTHDF2] |
|  | GO 0006412 translation | 19.0E-9 | 2.5E-6 | | 6.1E-9 | 83.0E-9 | [5, 6, 7] | | 19.02 | 39.00 | | [ADAR, CARS, CNOT1, DNAJC3, EEF2, EGFR, EIF2A, EIF2B4, EIF2S3, EIF3A, EIF3B, EIF3C, EIF3D, EIF3F, EIF3G, EIF4G2, EIF5, EIF5B, EIF6, ERBB2, FARSA, FARSB, FXR1, GATB, GFM1, GFM2, HARS2, KARS, LARP1, LARS2, MAP2K2, MARS2, PTCD3, QARS, RPS3, RPS4X, SNIP1, TRAP1, YTHDF2] |
|  | GO 0006413 translational initiation | 17.0E-6 | 380.0E-6 | | 6.1E-9 | 83.0E-9 | [3, 6, 7, 8] | | 25.00 | 15.00 | | [DNAJC3, EIF2B4, EIF2S3, EIF3A, EIF3B, EIF3C, EIF3D, EIF3F, EIF3G, EIF4G2, EIF5, EIF5B, EIF6, LARP1, YTHDF2] |
|  | GO 0019081 viral translation | 23.0E-6 | 490.0E-6 | | 6.1E-9 | 83.0E-9 | [4, 5, 6, 7, 8] | | 60.00 | 6.00 | | [EIF3A, EIF3B, EIF3D, EIF3F, EIF3G, EIF6] |
|  | GO 0006417 regulation of translation | 10.0E-3 | 44.0E-3 | | 6.1E-9 | 83.0E-9 | [5, 6, 7, 8] | | 13.16 | 20.00 | | [ADAR, CNOT1, DNAJC3, EEF2, EGFR, EIF2A, EIF3B, EIF4G2, EIF5, EIF5B, EIF6, ERBB2, FXR1, MAP2K2, QARS, RPS3, RPS4X, SNIP1, TRAP1, YTHDF2] |
|  | GO 0002190 cap-independent translational initiation | 410.0E-6 | 4.0E-3 | | 6.1E-9 | 83.0E-9 | [5, 8, 9, 10] | | 40.00 | 6.00 | | [EIF3A, EIF3B, EIF3D, EIF3F, EIF6, YTHDF2] |
|  | GO 0034976 response to endoplasmic reticulum stress | 35.0E-6 | 630.0E-6 | | 11.0E-6 | 37.0E-6 | [4] | | 17.78 | 24.00 | | [AGR2, BAG6, BAX, BCAP31, CDK5RAP3, DNAJB2, DNAJC3, ERP44, HSPA1A, KIAA0368, MAN1B1, NPLOC4, OPA1, OS9, PARK7, TMUB1, TP53BP1, TRIM25, UBAC2, UBXN1, UFL1, VCP, VIMP, WFS1] |
|  | GO 0030163 protein catabolic process | 9.0E-3 | 40.0E-3 | | 11.0E-6 | 37.0E-6 | [4, 5] | | 10.80 | 42.00 | | [BAG6, BCAP31, BNIP3L, CDK5RAP3, CLPX, DDRGK1, DNAJB2, EGFR, EZR, FAF1, HTRA2, IDE, KIAA0368, MAN1B1, NEDD4L, NPLOC4, NSF, OS9, PACSIN3, PARK7, PCNP, PCYOX1, PDCD6IP, PHB, PRPF19, PSMC2, PTPN23, RAB7A, RNF213, SDCBP, TMUB1, TRIM24, TRIM25, TSG101, UBA6, UBAC2, UBXN1, UCHL5, UFL1, VCP, VIMP, WFS1] |
|  | GO 0036503 ERAD pathway | 130.0E-6 | 1.6E-3 | | 11.0E-6 | 37.0E-6 | [3, 4, 5, 6] | | 22.22 | 14.00 | | [BAG6, BCAP31, DNAJB2, KIAA0368, MAN1B1, NPLOC4, OS9, TMUB1, TRIM25, UBAC2, UBXN1, VCP, VIMP, WFS1] |
|  | GO 1903573 negative regulation of response to endoplasmic reticulum stress | 540.0E-6 | 4.9E-3 | | 11.0E-6 | 37.0E-6 | [3, 4, 5, 6] | | 26.47 | 9.00 | | [DNAJC3, HSPA1A, OPA1, OS9, PARK7, UBAC2, UBXN1, VIMP, WFS1] |
|  | GO 0030970 retrograde protein transport, ER to cytosol | 7.8E-3 | 36.0E-3 | | 11.0E-6 | 37.0E-6 | [4, 5, 6, 7, 8] | | 24.00 | 6.00 | | [BCAP31, NPLOC4, OS9, UBAC2, VCP, VIMP] |
|  | GO 0032527 protein exit from endoplasmic reticulum | 1.0E-3 | 7.9E-3 | | 11.0E-6 | 37.0E-6 | [5, 6, 7] | | 24.32 | 9.00 | | [BCAP31, GCC2, LMAN1, NPLOC4, OS9, PREB, UBAC2, VCP, VIMP] |
|  | GO 0030433 ER-associated ubiquitin-dependent protein catabolic process | 4.5E-3 | 23.0E-3 | | 11.0E-6 | 37.0E-6 | [4, 5, 6, 7, 8, 9, 10, 11] | | 20.00 | 9.00 | | [BCAP31, DNAJB2, KIAA0368, MAN1B1, OS9, TMUB1, VCP, VIMP, WFS1] |
|  | GO 0070972 protein localization to endoplasmic reticulum | 190.0E-6 | 2.0E-3 | | 78.0E-6 | 230.0E-6 | [6] | | 33.33 | 8.00 | | [GBF1, OS9, RAB10, RAB3GAP2, SEC61A1, SEC63, SRP54, UBAC2] |
|  | GO 0072665 protein localization to vacuole | 11.0E-3 | 47.0E-3 | | 11.0E-3 | 11.0E-3 | [6] | | 22.22 | 6.00 | | [EZR, GCC2, PACSIN2, RAB7A, SCARB2, TOLLIP] |
|  | GO 1990542 mitochondrial transmembrane transport | 490.0E-6 | 4.5E-3 | | 3.7E-3 | 4.9E-3 | [5] | | 33.33 | 7.00 | | [ATP5O, CHCHD4, HSP90AA1, HSPA4, MICU2, SAMM50, TIMM21] |
|  | GO 0044743 intracellular protein transmembrane import | 9.5E-3 | 42.0E-3 | | 3.7E-3 | 4.9E-3 | [5, 6, 7, 8] | | 23.08 | 6.00 | | [CHCHD4, HSP90AA1, HSPA4, PEX13, SAMM50, TIMM21] |
|  | GO 0045040 protein import into mitochondrial outer membrane | 3.4E-3 | 18.0E-3 | | 3.7E-3 | 4.9E-3 | [4, 5, 6, 7, 8, 9] | | 60.00 | 3.00 | | [HSP90AA1, HSPA4, SAMM50] |
|  | GO 0006403 RNA localization | 270.0E-6 | 2.8E-3 | | 2.3E-9 | 48.0E-9 | [3] | | 20.00 | 15.00 | | [CCT2, CCT4, CCT5, CCT6A, CCT7, CCT8, EIF5A, FYTTD1, MMAB, NOP10, NUP133, NUP153, PNPT1, RUVBL1, TCP1] |
|  | GO 0006810 transport | 100.0E-6 | 1.3E-3 | | 2.3E-9 | 48.0E-9 | [3] | | 9.49 | 164.00 | | [AAK1, ABCB6, ABCC4, ACSL3, ACTN4, ADAR, AHCYL1, AHNAK, AIP, AP1G1, ATP1B3, ATP2A2, ATP2B4, ATP5B, ATP5O, ATP7A, ATXN2, BAX, BCAP31, BET1L, BORCS5, CDH1, CHCHD4, CHERP, CHMP1A, CIB1, COG2, COG3, COG4, COPA, CTNNB1, CXADR, CYB5R1, DCTN1, DLG1, DNAJC1, DNM1L, DNM2, EEA1, EGFR, EHD4, EIF5A, EPS15, EPT1, ERBB2, ERP29, EZR, FAF1, FLVCR1, FN1, FYTTD1, GBF1, GCC2, GDAP1, GDI1, GOLGA5, GOSR1, GRB2, HID1, HOOK1, HSP90AA1, HSPA1A, HSPA4, HTRA2, HUWE1, JUP, LAMP1, LAMTOR1, LMAN1, LONP2, LRRC8E, MACF1, MFN2, MICU2, MYADM, MYH9, MYO18A, MYO5A, MYO6, NEDD4L, NPEPPS, NPLOC4, NSF, NUP133, NUP153, OS9, OSBPL8, PACSIN2, PARK7, PDCD6IP, PEA15, PEX13, PIK3C2A, PMPCA, PNPT1, PPIA, PPIF, PPT1, PREB, PSMB7, PTPN11, PTPN23, RAB10, RAB11B, RAB2A, RAB3B, RAB7A, RAB9A, RALA, RAP1A, REST, RHOT1, RPL10, RPSA, RUVBL1, SAMM50, SCARB2, SCYL1, SDCBP, SEC61A1, SEC63, SLC25A17, SLC25A24, SLC27A2, SLC30A5, SLC35B2, SLC39A6, SLC9A1, SNIP1, SNX1, SNX2, SNX27, SNX5, SPAG9, SRI, SRP54, STEAP2, STX12, STX18, STXBP1, STXBP2, STXBP3, TAP1, TBC1D5, TIMM21, TMEM115, TMEM165, TNPO1, TOMM34, TOR1A, TSG101, U2AF1, UBAC2, VAMP7, VCP, VIMP, VPS33A, VPS35, VTA1, WDR46, WFS1, XPO7, YWHAE, ZW10] |
|  | GO 0008104 protein localization | 390.0E-9 | 34.0E-6 | | 2.3E-9 | 48.0E-9 | [3] | | 11.25 | 121.00 | | [AAK1, ACSL3, ADAR, AGR2, AIP, ATP1B3, BAG6, BCAP31, CCT2, CCT4, CCT5, CCT6A, CCT7, CCT8, CDH1, CDK5RAP3, CHCHD4, CHERP, CIB1, COG3, CTNNB1, CYB5R1, DCP1A, DDX1, DDX42, DLG1, DNAJB2, DNAJC1, DNM1L, DNM2, EGFR, EIF5A, EPT1, ERBB2, ERP29, EZR, FAF1, FLOT2, FN1, GBF1, GCC2, GDAP1, GDI1, GNL3, GNL3L, HID1, HOOK1, HSP90AA1, HSPA4, HTRA2, HUWE1, IST1, JUP, LAMP1, LAMTOR1, LMAN1, LONP2, MACF1, MFN2, MMAB, MPP7, MTCH2, MYADM, MYH9, MYO18A, NEDD4L, NPEPPS, NPLOC4, NSF, OS9, PACSIN2, PARK7, PEX13, PINX1, PKP3, PMPCA, PPFIA1, PPIA, PPT1, PREB, PRPF19, PSMB7, RAB10, RAB11B, RAB3GAP2, RAB7A, RAB9A, RAP1A, REST, RPL10, RUVBL1, SAMM50, SCARB2, SCRIB, SEC61A1, SEC63, SIN3A, SLC9A1, SNIP1, SNX1, SNX27, SRP54, STXBP1, TAP1, TCP1, TIMM21, TMBIM1, TOLLIP, TOMM34, TOR1A, TP53BP1, U2AF1, UBAC2, UFL1, VCL, VCP, VIMP, VTI1B, WDR46, XPO7, ZW10] |
|  | GO 0031647 regulation of protein stability | 170.0E-6 | 1.9E-3 | | 2.3E-9 | 48.0E-9 | [3] | | 15.43 | 27.00 | | [AAK1, ATP1B3, BAG6, CCT2, CCT4, CCT5, CCT6A, CCT7, CCT8, COG3, CTSA, CTSH, DNAJA3, FLOT2, GNL3L, HSPA1A, LSS, PARK7, PFN1, PHB, PINX1, PRKD1, STX12, TCP1, TRIM24, VPS35, WFS1] |
|  | GO 0051649 establishment of localization in cell | 540.0E-9 | 33.0E-6 | | 2.3E-9 | 48.0E-9 | [3] | | 11.90 | 97.00 | | [ACTN4, ADAR, AIP, AP1G1, ATP5O, BCAP31, BET1L, BORCS5, CDH1, CENPE, CHCHD4, CHERP, CHMP1A, CIB1, COG3, CYB5R1, DCTN1, DLG1, DYNC1H1, EGFR, EIF5A, EPS15, EPT1, ERBB2, EZR, FAF1, FYTTD1, GBF1, GCC2, GDAP1, GDI1, GOSR1, HID1, HOOK1, HSP90AA1, HSPA4, HTRA2, HUWE1, JUP, KIF2C, LAMP1, LMAN1, LONP2, MAP4, MFN2, MYADM, MYO5A, NPEPPS, NPLOC4, NSF, NUP133, NUP153, OS9, PAFAH1B1, PARK7, PDCD6IP, PEX13, PINX1, PMPCA, PREB, PSMB7, PTPN23, RAB2A, RAB3B, RAB7A, RHOT1, RPL10, RUVBL1, SAMM50, SCARB2, SEC61A1, SEC63, SLC9A1, SNIP1, SNX1, SNX2, SNX27, SNX5, SPAG9, SRI, SRP54, STX18, TAP1, TBC1D5, TIMM21, TOMM34, TOR1A, U2AF1, UBAC2, VAMP7, VCP, VIMP, VPS33A, VPS35, WDR46, XPO7, ZW10] |
|  | GO 1902580 single-organism cellular localization | 1.8E-3 | 11.0E-3 | | 2.3E-9 | 48.0E-9 | [3] | | 11.24 | 50.00 | | [ACSL3, ACTN4, ADAR, AGR2, ATP1B3, ATP5O, BAG6, BORCS5, CDH1, CHCHD4, CHERP, CIB1, DLG1, DYNC1H1, EGFR, ERBB2, EZR, FAF1, FLOT2, GCC2, GDI1, HSP90AA1, HSPA4, JUP, LAMP1, MACF1, MAP4, MYADM, MYO5A, PAFAH1B1, PEX13, PKP3, PPFIA1, RAB10, RAB11B, RAB3GAP2, RHOT1, RPL10, SAMM50, SEC61A1, SEC63, SLC9A1, STXBP1, TAP1, TIMM21, TMBIM1, TOR1A, VAMP7, VTI1B, ZW10] |
|  | GO 0032880 regulation of protein localization | 2.3E-3 | 14.0E-3 | | 2.3E-9 | 48.0E-9 | [3, 4] | | 10.68 | 60.00 | | [AAK1, ACSL3, AGR2, BCAP31, CCT2, CCT4, CCT5, CCT6A, CCT7, CCT8, CDH1, CDK5RAP3, CHERP, CIB1, CTNNB1, CYB5R1, DLG1, DNAJB2, DNAJC1, DNM1L, EGFR, EPT1, ERBB2, ERP29, EZR, FAF1, FN1, GBF1, GCC2, GDI1, GNL3, GNL3L, HTRA2, HUWE1, JUP, MYO18A, NEDD4L, NPEPPS, OS9, PARK7, PINX1, PPFIA1, PPIA, PSMB7, RAB11B, RAB9A, REST, RPL10, RUVBL1, SIN3A, SLC9A1, SNIP1, TCP1, TMBIM1, TOR1A, U2AF1, UBAC2, UFL1, VTI1B, WDR46] |
|  | GO 0045184 establishment of protein localization | 15.0E-6 | 360.0E-6 | | 2.3E-9 | 48.0E-9 | [3, 4] | | 11.30 | 91.00 | | [ACSL3, ADAR, AGR2, AIP, BAG6, BCAP31, CCT2, CCT4, CCT5, CCT6A, CCT7, CCT8, CDH1, CHCHD4, CHERP, CIB1, CYB5R1, DLG1, DNAJC1, DNM1L, DNM2, EGFR, EIF5A, EPT1, ERBB2, ERP29, EZR, FAF1, FLOT2, FN1, GCC2, GDAP1, GDI1, HID1, HOOK1, HSP90AA1, HSPA4, HTRA2, HUWE1, IST1, JUP, LAMP1, LMAN1, LONP2, MACF1, MFN2, MYADM, MYH9, MYO18A, NPEPPS, NPLOC4, NSF, OS9, PARK7, PEX13, PKP3, PMPCA, PPFIA1, PPIA, PPT1, PREB, PSMB7, RAB10, RAB11B, RAB3GAP2, RAB7A, RAP1A, REST, RPL10, RUVBL1, SAMM50, SCARB2, SEC61A1, SEC63, SLC9A1, SNIP1, SNX1, SNX27, SRP54, TAP1, TCP1, TIMM21, TMBIM1, TOMM34, TOR1A, U2AF1, UBAC2, VCP, VIMP, WDR46, XPO7] |
|  | GO 0046907 intracellular transport | 460.0E-9 | 30.0E-6 | | 2.3E-9 | 48.0E-9 | [3, 4] | | 12.45 | 85.00 | | [ACTN4, ADAR, AIP, AP1G1, ATP5O, BCAP31, BET1L, BORCS5, CDH1, CHCHD4, CHERP, CIB1, COG3, CYB5R1, DCTN1, EGFR, EIF5A, EPS15, EPT1, ERBB2, EZR, FAF1, FYTTD1, GBF1, GCC2, GDAP1, GDI1, GOSR1, HID1, HOOK1, HSP90AA1, HSPA4, HTRA2, HUWE1, JUP, LAMP1, LMAN1, LONP2, MFN2, MYADM, MYO5A, NPEPPS, NPLOC4, NSF, NUP133, NUP153, OS9, PARK7, PEX13, PMPCA, PREB, PSMB7, PTPN23, RAB2A, RAB7A, RHOT1, RPL10, RUVBL1, SAMM50, SCARB2, SEC61A1, SEC63, SLC9A1, SNIP1, SNX1, SNX2, SNX27, SNX5, SPAG9, SRP54, STX18, TAP1, TBC1D5, TIMM21, TOMM34, U2AF1, UBAC2, VAMP7, VCP, VIMP, VPS33A, VPS35, WDR46, XPO7, ZW10] |
|  | GO 0060341 regulation of cellular localization | 560.0E-6 | 4.9E-3 | | 2.3E-9 | 48.0E-9 | [3, 4] | | 11.52 | 56.00 | | [ACSL3, AGR2, BCAP31, CCT2, CCT4, CCT5, CCT6A, CCT7, CCT8, CDH1, CDK5RAP3, CHERP, CIB1, CTNNB1, CYB5R1, DLG1, EGFR, EPT1, ERBB2, EZR, FAF1, GBF1, GCC2, GDI1, GNL3, GNL3L, HTRA2, HUWE1, JUP, LAMP1, NEDD4L, NPEPPS, NUP153, OS9, PARK7, PINX1, PPFIA1, PSMB7, PTPN23, RAB11B, RAB3B, RPL10, RUVBL1, SIN3A, SLC9A1, SNIP1, SRI, STX18, TCP1, TMBIM1, TOR1A, U2AF1, UBAC2, VAMP7, VTI1B, WDR46] |
|  | GO 0006839 mitochondrial transport | 37.0E-6 | 650.0E-6 | | 2.3E-9 | 48.0E-9 | [4] | | 16.47 | 28.00 | | [AIP, ATP5O, CHCHD4, CYB5R1, EPT1, FLVCR1, GDAP1, HSP90AA1, HSPA1A, HSPA4, HTRA2, HUWE1, MFN2, MICU2, NPEPPS, PMPCA, PNPT1, PPIF, PSMB7, RHOT1, RUVBL1, SAMM50, SLC25A24, SNIP1, TIMM21, TOMM34, U2AF1, WDR46] |
|  | GO 0007005 mitochondrion organization | 1.9E-6 | 83.0E-6 | | 2.3E-9 | 48.0E-9 | [4] | | 14.72 | 48.00 | | [ACAD9, AIP, BAX, BNIP3L, CHCHD4, CYB5R1, DNM1L, EPT1, FOXRED1, GATB, GDAP1, GFM1, GFM2, HSP90AA1, HSPA1A, HSPA4, HTRA2, HUWE1, IMMT, LIG3, MFN1, MFN2, MYH14, NDUFAF4, NDUFS1, NDUFS8, NPEPPS, OPA1, PHB, PMPCA, PNPT1, PPIF, PSMB7, PTCD3, RHOT1, RUVBL1, SAMM50, SFN, SMIM20, SNIP1, SUPV3L1, TFAM, TIMM21, TOMM34, TP53BP1, TTC19, U2AF1, WDR46] |
|  | GO 0016192 vesicle-mediated transport | 96.0E-6 | 1.2E-3 | | 2.3E-9 | 48.0E-9 | [4] | | 12.26 | 57.00 | | [AAK1, AP1G1, ATXN2, BET1L, CHMP1A, COG2, COG3, COG4, DCTN1, DNM2, EEA1, EGFR, EHD4, EPS15, EZR, GBF1, GCC2, GOLGA5, GOSR1, GRB2, HOOK1, LAMP1, LMAN1, MYO18A, MYO5A, MYO6, PACSIN2, PDCD6IP, PIK3C2A, PPT1, PTPN23, RAB11B, RAB2A, RAB7A, RAB9A, RALA, SCYL1, SDCBP, SNX1, SNX2, SNX27, SNX5, SPAG9, STEAP2, STX18, STXBP1, STXBP2, STXBP3, TBC1D5, TMEM115, TOR1A, TSG101, VAMP7, VPS33A, VPS35, VTA1, ZW10] |
|  | GO 0034613 cellular protein localization | 12.0E-9 | 1.9E-6 | | 2.3E-9 | 48.0E-9 | [4] | | 12.61 | 102.00 | | [ACSL3, ADAR, AGR2, AIP, ATP1B3, BAG6, BCAP31, CCT2, CCT4, CCT5, CCT6A, CCT7, CCT8, CDH1, CDK5RAP3, CHCHD4, CHERP, CIB1, COG3, CTNNB1, CYB5R1, DCP1A, DDX1, DLG1, EGFR, EIF5A, EPT1, ERBB2, EZR, FAF1, FLOT2, GBF1, GCC2, GDAP1, GDI1, GNL3, GNL3L, HID1, HOOK1, HSP90AA1, HSPA4, HTRA2, HUWE1, JUP, LAMP1, LAMTOR1, LMAN1, LONP2, MACF1, MFN2, MMAB, MTCH2, MYADM, NEDD4L, NPEPPS, NPLOC4, NSF, OS9, PACSIN2, PARK7, PEX13, PINX1, PKP3, PMPCA, PPFIA1, PREB, PRPF19, PSMB7, RAB10, RAB11B, RAB3GAP2, RAB7A, RPL10, RUVBL1, SAMM50, SCARB2, SEC61A1, SEC63, SIN3A, SLC9A1, SNIP1, SNX1, SNX27, SRP54, STXBP1, TAP1, TCP1, TIMM21, TMBIM1, TOLLIP, TOMM34, TOR1A, TP53BP1, U2AF1, UBAC2, VCL, VCP, VIMP, VTI1B, WDR46, XPO7, ZW10] |
|  | GO 0050821 protein stabilization | 460.0E-6 | 4.4E-3 | | 2.3E-9 | 48.0E-9 | [4] | | 17.31 | 18.00 | | [AAK1, ATP1B3, CCT2, CCT4, CCT5, CCT6A, CCT7, CCT8, COG3, DNAJA3, FLOT2, HSPA1A, PARK7, PFN1, PHB, STX12, TCP1, WFS1] |
|  | GO 0071702 organic substance transport | 7.6E-3 | 35.0E-3 | | 2.3E-9 | 48.0E-9 | [4] | | 9.50 | 90.00 | | [ABCB6, ABCC4, ACSL3, ADAR, AIP, BCAP31, CDH1, CHCHD4, CHERP, CIB1, CYB5R1, DNAJC1, DNM1L, DNM2, EGFR, EIF5A, EPT1, ERBB2, ERP29, EZR, FAF1, FLVCR1, FN1, FYTTD1, GCC2, GDAP1, GDI1, HID1, HSP90AA1, HSPA4, HTRA2, HUWE1, JUP, LAMTOR1, LMAN1, LONP2, MACF1, MFN2, MYADM, MYH9, MYO18A, NPEPPS, NPLOC4, NSF, NUP133, NUP153, OS9, OSBPL8, PARK7, PEA15, PEX13, PMPCA, PNPT1, PPIA, PPT1, PREB, PSMB7, PTPN11, RAB3B, RAB7A, RAP1A, REST, RPL10, RPSA, RUVBL1, SAMM50, SCARB2, SEC61A1, SEC63, SLC25A17, SLC25A24, SLC27A2, SLC35B2, SLC9A1, SNIP1, SNX1, SNX27, SRP54, STX12, TAP1, TIMM21, TOMM34, TOR1A, U2AF1, UBAC2, VAMP7, VCP, VIMP, WDR46, XPO7] |
|  | GO 1903829 positive regulation of cellular protein localization | 460.0E-6 | 4.3E-3 | | 2.3E-9 | 48.0E-9 | [2, 3, 4, 5, 6] | | 13.49 | 34.00 | | [ACSL3, AGR2, BCAP31, CCT2, CCT4, CCT5, CCT6A, CCT7, CCT8, CDH1, CDK5RAP3, CHERP, CIB1, CYB5R1, DLG1, EGFR, EPT1, ERBB2, EZR, GNL3, GNL3L, HTRA2, HUWE1, JUP, NPEPPS, PARK7, PINX1, PSMB7, RUVBL1, SLC9A1, SNIP1, TCP1, U2AF1, WDR46] |
|  | GO 0015031 protein transport | 1.4E-3 | 9.9E-3 | | 2.3E-9 | 48.0E-9 | [4, 5] | | 10.53 | 71.00 | | [ACSL3, ADAR, AIP, BCAP31, CDH1, CHCHD4, CHERP, CIB1, CYB5R1, DNAJC1, DNM1L, DNM2, EGFR, EIF5A, EPT1, ERBB2, ERP29, EZR, FAF1, FN1, GCC2, GDAP1, GDI1, HID1, HSP90AA1, HSPA4, HTRA2, HUWE1, JUP, LMAN1, LONP2, MACF1, MFN2, MYADM, MYH9, MYO18A, NPEPPS, NPLOC4, NSF, OS9, PARK7, PEX13, PMPCA, PPIA, PPT1, PREB, PSMB7, RAB7A, RAP1A, REST, RPL10, RUVBL1, SAMM50, SCARB2, SEC61A1, SEC63, SLC9A1, SNIP1, SNX1, SNX27, SRP54, TAP1, TIMM21, TOMM34, TOR1A, U2AF1, UBAC2, VCP, VIMP, WDR46, XPO7] |
|  | GO 1903827 regulation of cellular protein localization | 72.0E-6 | 1.0E-3 | | 2.3E-9 | 48.0E-9 | [4, 5] | | 12.86 | 49.00 | | [ACSL3, AGR2, BCAP31, CCT2, CCT4, CCT5, CCT6A, CCT7, CCT8, CDH1, CDK5RAP3, CHERP, CIB1, CTNNB1, CYB5R1, DLG1, EGFR, EPT1, ERBB2, EZR, FAF1, GBF1, GCC2, GDI1, GNL3, GNL3L, HTRA2, HUWE1, JUP, NEDD4L, NPEPPS, OS9, PARK7, PINX1, PPFIA1, PSMB7, RAB11B, RPL10, RUVBL1, SIN3A, SLC9A1, SNIP1, TCP1, TMBIM1, TOR1A, U2AF1, UBAC2, VTI1B, WDR46] |
|  | GO 1904874 positive regulation of telomerase RNA localization to Cajal body | 390.0E-9 | 31.0E-6 | | 2.3E-9 | 48.0E-9 | [2, 3, 4, 5, 6, 7] | | 56.25 | 9.00 | | [CCT2, CCT4, CCT5, CCT6A, CCT7, CCT8, NOP10, RUVBL1, TCP1] |
|  | GO 0006886 intracellular protein transport | 44.0E-6 | 730.0E-6 | | 2.3E-9 | 48.0E-9 | [4, 5, 6] | | 12.61 | 56.00 | | [ADAR, AIP, BCAP31, CDH1, CHCHD4, CHERP, CIB1, CYB5R1, EGFR, EIF5A, EPT1, ERBB2, FAF1, GCC2, GDAP1, GDI1, HID1, HSP90AA1, HSPA4, HTRA2, HUWE1, JUP, LMAN1, LONP2, MFN2, MYADM, NPEPPS, NPLOC4, NSF, OS9, PARK7, PEX13, PMPCA, PREB, PSMB7, RAB7A, RPL10, RUVBL1, SAMM50, SCARB2, SEC61A1, SEC63, SLC9A1, SNIP1, SNX1, SNX27, SRP54, TAP1, TIMM21, TOMM34, U2AF1, UBAC2, VCP, VIMP, WDR46, XPO7] |
|  | GO 0033365 protein localization to organelle | 780.0E-9 | 44.0E-6 | | 2.3E-9 | 48.0E-9 | [5] | | 13.35 | 67.00 | | [ADAR, AIP, BAG6, CCT2, CCT4, CCT5, CCT6A, CCT7, CCT8, CDH1, CDK5RAP3, CHCHD4, CHERP, COG3, CYB5R1, DCP1A, DDX1, EGFR, EPT1, EZR, FAF1, GBF1, GCC2, GDAP1, GNL3, GNL3L, HOOK1, HSP90AA1, HSPA4, HTRA2, HUWE1, JUP, LAMP1, LONP2, MFN2, MMAB, MTCH2, NPEPPS, OS9, PACSIN2, PARK7, PEX13, PINX1, PMPCA, PSMB7, RAB10, RAB3GAP2, RAB7A, RPL10, RUVBL1, SAMM50, SCARB2, SEC61A1, SEC63, SIN3A, SLC9A1, SNIP1, SRP54, TCP1, TIMM21, TOLLIP, TOMM34, TOR1A, U2AF1, UBAC2, WDR46, ZW10] |
|  | GO 0072594 establishment of protein localization to organelle | 16.0E-6 | 370.0E-6 | | 2.3E-9 | 48.0E-9 | [4, 5, 6] | | 13.78 | 47.00 | | [ADAR, AIP, CCT2, CCT4, CCT5, CCT6A, CCT7, CCT8, CDH1, CHCHD4, CHERP, CYB5R1, EGFR, EPT1, FAF1, GCC2, GDAP1, HOOK1, HSP90AA1, HSPA4, HTRA2, HUWE1, JUP, LAMP1, LONP2, MFN2, NPEPPS, PEX13, PMPCA, PSMB7, RAB10, RAB3GAP2, RAB7A, RPL10, RUVBL1, SAMM50, SCARB2, SEC61A1, SEC63, SLC9A1, SNIP1, SRP54, TCP1, TIMM21, TOMM34, U2AF1, WDR46] |
|  | GO 0090666 scaRNA localization to Cajal body | 1.4E-3 | 10.0E-3 | | 2.3E-9 | 48.0E-9 | [5] | | 75.00 | 3.00 | | [CCT2, CCT4, TCP1] |
|  | GO 1900182 positive regulation of protein localization to nucleus | 960.0E-6 | 7.3E-3 | | 2.3E-9 | 48.0E-9 | [3, 4, 5, 6, 7, 8] | | 17.86 | 15.00 | | [CCT2, CCT4, CCT5, CCT6A, CCT7, CCT8, CDH1, CDK5RAP3, CHERP, EGFR, JUP, PARK7, PINX1, SLC9A1, TCP1] |
|  | GO 0006605 protein targeting | 1.7E-3 | 11.0E-3 | | 2.3E-9 | 48.0E-9 | [5, 6, 7] | | 11.94 | 40.00 | | [ADAR, AIP, CDH1, CHCHD4, CHERP, CIB1, CYB5R1, EGFR, EPT1, ERBB2, FAF1, GCC2, GDAP1, GDI1, HSP90AA1, HSPA4, HTRA2, HUWE1, JUP, LONP2, MFN2, MYADM, NPEPPS, PEX13, PMPCA, PSMB7, RAB7A, RPL10, RUVBL1, SAMM50, SCARB2, SEC61A1, SEC63, SLC9A1, SNIP1, SRP54, TIMM21, TOMM34, U2AF1, WDR46] |
|  | GO 0032206 positive regulation of telomere maintenance | 1.5E-3 | 10.0E-3 | | 2.3E-9 | 48.0E-9 | [3, 4, 5, 6, 7, 8, 9] | | 23.08 | 9.00 | | [CCT2, CCT4, CCT5, CCT6A, CCT7, CCT8, GNL3, NBN, TCP1] |
|  | GO 0034502 protein localization to chromosome | 570.0E-6 | 4.9E-3 | | 2.3E-9 | 48.0E-9 | [6] | | 21.43 | 12.00 | | [CCT2, CCT4, CCT5, CCT6A, CCT7, CCT8, GNL3, GNL3L, MMAB, PINX1, TCP1, ZW10] |
|  | GO 0070585 protein localization to mitochondrion | 330.0E-6 | 3.3E-3 | | 2.3E-9 | 48.0E-9 | [6] | | 16.18 | 22.00 | | [AIP, CHCHD4, CYB5R1, EPT1, GDAP1, HOOK1, HSP90AA1, HSPA4, HTRA2, HUWE1, MFN2, MTCH2, NPEPPS, PMPCA, PSMB7, RUVBL1, SAMM50, SNIP1, TIMM21, TOMM34, U2AF1, WDR46] |
|  | GO 1900180 regulation of protein localization to nucleus | 10.0E-3 | 45.0E-3 | | 2.3E-9 | 48.0E-9 | [5, 6, 7] | | 13.43 | 18.00 | | [CCT2, CCT4, CCT5, CCT6A, CCT7, CCT8, CDH1, CDK5RAP3, CHERP, EGFR, FAF1, JUP, PARK7, PINX1, RPL10, SIN3A, SLC9A1, TCP1] |
|  | GO 1904816 positive regulation of protein localization to chromosome, telomeric region | 9.8E-9 | 1.9E-6 | | 2.3E-9 | 48.0E-9 | [3, 4, 5, 6, 7, 8, 9] | | 75.00 | 9.00 | | [CCT2, CCT4, CCT5, CCT6A, CCT7, CCT8, GNL3, GNL3L, TCP1] |
|  | GO 1904851 positive regulation of establishment of protein localization to telomere | 330.0E-9 | 33.0E-6 | | 2.3E-9 | 48.0E-9 | [3, 4, 5, 6, 7, 8, 9, 10] | | 77.78 | 7.00 | | [CCT2, CCT4, CCT5, CCT6A, CCT7, CCT8, TCP1] |
|  | GO 0032210 regulation of telomere maintenance via telomerase | 1.0E-3 | 7.9E-3 | | 2.3E-9 | 48.0E-9 | [5, 6, 7, 8, 9, 10] | | 24.32 | 9.00 | | [CCT2, CCT4, CCT5, CCT6A, CCT7, CCT8, GNL3L, PINX1, TCP1] |
|  | GO 1904871 positive regulation of protein localization to Cajal body | 79.0E-9 | 8.9E-6 | | 2.3E-9 | 48.0E-9 | [4, 5, 6, 7, 8, 9, 10, 11] | | 87.50 | 7.00 | | [CCT2, CCT4, CCT5, CCT6A, CCT7, CCT8, TCP1] |
|  | GO 0032212 positive regulation of telomere maintenance via telomerase | 2.5E-3 | 14.0E-3 | | 2.3E-9 | 48.0E-9 | [5, 6, 7, 8, 9, 10, 11] | | 25.93 | 7.00 | | [CCT2, CCT4, CCT5, CCT6A, CCT7, CCT8, TCP1] |
|  | GO 1903405 protein localization to nuclear body | 79.0E-9 | 8.9E-6 | | 2.3E-9 | 48.0E-9 | [8] | | 87.50 | 7.00 | | [CCT2, CCT4, CCT5, CCT6A, CCT7, CCT8, TCP1] |
|  | GO 0002934 desmosome organization | 6.5E-3 | 32.0E-3 | | 6.5E-3 | 7.6E-3 | [5] | | 50.00 | 3.00 | | [DSG2, JUP, PKP3] |
|  | GO 0048518 positive regulation of biological process | 890.0E-6 | 6.9E-3 | | 1.0E-6 | 4.8E-6 | [1, 2, 3] | | 6.07 | 186.00 | | [AAK1, ABHD14B, ACSL3, ACTN4, ACTR3, ADAR, AGR2, AHCYL1, ANKLE2, AP1G1, ARL6IP5, ATP1B3, ATP2B4, ATP7A, BAG6, BAX, BCAP31, BNIP3L, BRD4, C14orf166, CC2D1A, CCT2, CCT4, CCT5, CCT6A, CCT7, CCT8, CD276, CDH1, CDK5RAP3, CENPE, CHERP, CIB1, CNOT1, CTNNB1, CTSH, CYB5R1, CYFIP1, DDRGK1, DLG1, DNAJA3, DNAJB2, DNM1L, DNMT1, EEF1D, EEF1E1, EEF2, EGFR, EIF5A, EPT1, ERBB2, ERP29, ETFA, ETFDH, EZR, FAF1, FASN, FLOT2, FN1, FXR1, GARS, GDF15, GIPC1, GNL3, GNL3L, GRB2, HSPA1A, HTRA2, HUWE1, IDE, ILK, IST1, JUP, KMT2D, LAMP1, LAMTOR1, LARP1, LMAN1, MACF1, MAP2K2, MAPT, MARK2, MLYCD, MMAB, MPP7, MTPN, MYADM, MYDGF, MYO18A, MYO6, NACC1, NAMPT, NBN, NCOA6, NDRG1, NDUFA2, NEDD4L, NOP10, NPEPPS, NSF, NUP62, PACSIN3, PAK2, PARK7, PDCD6IP, PELP1, PFN1, PHB, PHIP, PINX1, PNPLA2, PNPT1, PPIA, PPIE, PPP2CA, PPT1, PRDX5, PRKD1, PRMT1, PRMT5, PSMB7, PTPN11, PTPN23, PTPRJ, QARS, RAB3B, RAB3GAP2, RAB7A, RAB9A, RALA, RBM5, REST, RHOA, RNMT, RPL10, RPRD1B, RPS15A, RPS3, RPS4X, RPS6, RPSA, RRAS, RUVBL1, SCRIB, SDCBP, SEPT9, SHC1, SHTN1, SIN3A, SLC35B2, SLC44A2, SLC9A1, SMARCB1, SMARCC2, SNIP1, SPAG9, SPEN, SRI, STRN3, STX18, SUPV3L1, TBC1D5, TCP1, TFAM, TKFC, TMPRSS2, TNPO1, TOM1L1, TOR1A, TP53BP1, TPD52L1, TRIM25, TRIOBP, TSG101, U2AF1, UBE2V1, UFL1, USP47, UTRN, VAMP7, VCP, WASF2, WDR46, WFS1, YTHDF2, ZC3HAV1] |
|  | GO 0048583 regulation of response to stimulus | 3.5E-3 | 18.0E-3 | | 1.0E-6 | 4.8E-6 | [2, 3] | | 5.70 | 100.00 | | [AAK1, ACTN4, AGR2, ANXA5, AP1G1, ARHGDIA, ARL6IP5, ATP2B4, BAG6, BAX, BCAP31, BRD4, CC2D1A, CDC37, CDK5RAP3, CIB1, CNOT1, CTSH, DDRGK1, DLG1, DNAJA3, DNAJC3, DNM1L, EEF1D, EEF1E1, EGFR, ELMOD2, ERBB2, ERP29, ETFA, ETFDH, EZR, FAF1, FLOT2, FN1, HSPA1A, HTRA2, ILK, IST1, ITPR1, KMT2D, LAMP1, LAMTOR1, MAP2K2, MDH1, MFN2, MMAB, MTPN, MYADM, MYDGF, NUP62, OPA1, OS9, OTUB1, PAK2, PARK7, PEA15, PEX13, PHB, PHIP, PNPT1, PPIF, PRKD1, PRMT5, PTPN11, PTPRJ, RAB7A, RHOA, RNF213, RNMT, RPA2, RPL10, RPS3, RPSA, RRAS, SDCBP, SHC1, SIN3A, SLC35B2, SLC44A2, SLC9A1, SMARCB1, SNRPB, SOD2, SRI, SYNJ2BP, TKFC, TMBIM1, TP53BP1, TPD52L1, TRIM25, TSG101, UBAC2, UBE2V1, UBXN1, UFL1, VAMP7, VIMP, WFS1, ZC3HAV1] |
|  | GO 0007154 cell communication | 1.4E-3 | 10.0E-3 | | 1.0E-6 | 4.8E-6 | [3] | | 5.76 | 121.00 | | [AAK1, ACTN4, AGR2, AHCYL1, AIMP1, ARHGDIA, ARL6IP5, ATP2A2, ATP2B4, BAG6, BAX, BCAP31, BRD4, CC2D1A, CDC37, CDC5L, CDK5RAP3, CHMP1A, CIB1, CNOT1, CTNNB1, CTSH, CYFIP1, DDRGK1, DLG1, DNAJA3, DNM1L, DNMT1, DSG2, DSP, EEF1D, EGFR, EPHB3, ERBB2, ERP29, ETFA, ETFDH, EZR, FAF1, FARP2, FASN, FLOT2, FN1, GARS, GNAI3, GRB2, HSPA1A, HTRA2, ILK, IMPA1, ITPR1, JUP, KMT2D, LAMP1, LAMTOR1, LARP1, MAP2K2, MARK2, MFN2, MMAB, MYADM, MYH14, MYO6, NAMPT, NDRG1, NUP62, OPA1, PAK2, PARK7, PEA15, PHB, PHIP, PPIF, PRKD1, PRMT5, PRPF19, PTPN11, PTPRJ, RAB3B, RAB7A, RAP1A, RBFOX2, REST, RHOA, RHOT1, RNF213, RNMT, RPL10, RPS3, RPS6, RPSA, RRAS, SDCBP, SFN, SHC1, SLC35B2, SLC44A2, SLC9A1, SMARCB1, SOD2, SRI, STRN, SYNJ2BP, TKFC, TMBIM1, TOLLIP, TOM1L1, TOM1L2, TOR1A, TP53BP1, TPD52L1, TRIM25, TSG101, UBE2V1, UFL1, VAMP7, VIMP, WFS1, WNK1, YWHAG, ZC3HAV1] |
|  | GO 0048522 positive regulation of cellular process | 3.8E-3 | 20.0E-3 | | 1.0E-6 | 4.8E-6 | [2, 3, 4] | | 6.14 | 172.00 | | [AAK1, ABHD14B, ACSL3, ACTN4, ACTR3, ADAR, AGR2, ANKLE2, AP1G1, ARL6IP5, ATP1B3, ATP2B4, BAG6, BAX, BCAP31, BNIP3L, BRD4, C14orf166, CC2D1A, CCT2, CCT4, CCT5, CCT6A, CCT7, CCT8, CD276, CDH1, CDK5RAP3, CENPE, CIB1, CNOT1, CTNNB1, CTSH, CYB5R1, CYFIP1, DDRGK1, DLG1, DNAJA3, DNAJB2, DNM1L, DNMT1, EEF1D, EEF1E1, EEF2, EGFR, EIF5A, EPT1, ERBB2, ERP29, ETFA, ETFDH, EZR, FAF1, FASN, FLOT2, FN1, FXR1, GARS, GDF15, GIPC1, GNL3, GRB2, HSPA1A, HTRA2, HUWE1, IDE, ILK, IST1, KMT2D, LAMP1, LAMTOR1, LARP1, LMAN1, MACF1, MAP2K2, MAPT, MARK2, MLYCD, MMAB, MPP7, MYADM, MYDGF, MYO18A, MYO6, NACC1, NAMPT, NBN, NCOA6, NDRG1, NEDD4L, NPEPPS, NSF, NUP62, PACSIN3, PAK2, PARK7, PDCD6IP, PELP1, PFN1, PHB, PHIP, PNPLA2, PNPT1, PPIA, PPIE, PPP2CA, PPT1, PRKD1, PRMT1, PRMT5, PSMB7, PTPN11, PTPN23, PTPRJ, QARS, RAB3B, RAB3GAP2, RAB7A, RAB9A, RALA, RBM5, REST, RHOA, RNMT, RPL10, RPRD1B, RPS15A, RPS3, RPS4X, RPS6, RPSA, RUVBL1, SCRIB, SDCBP, SEPT9, SHC1, SHTN1, SIN3A, SLC35B2, SLC44A2, SLC9A1, SMARCB1, SMARCC2, SNIP1, SPAG9, SPEN, SRI, STRN3, STX18, SUPV3L1, TBC1D5, TCP1, TFAM, TMPRSS2, TOM1L1, TOR1A, TP53BP1, TPD52L1, TRIM25, TRIOBP, TSG101, U2AF1, UBE2V1, UFL1, USP47, VAMP7, VCP, WASF2, WDR46, WFS1, YTHDF2, ZC3HAV1] |
|  | GO 0007165 signal transduction | 560.0E-6 | 4.9E-3 | | 1.0E-6 | 4.8E-6 | [3, 4] | | 5.58 | 110.00 | | [AAK1, ACTN4, AGR2, AHCYL1, ARHGDIA, ARL6IP5, ATP2A2, ATP2B4, BAG6, BAX, BCAP31, BRD4, CC2D1A, CDC37, CDC5L, CDK5RAP3, CIB1, CNOT1, CTNNB1, CTSH, CYFIP1, DDRGK1, DLG1, DNAJA3, DNM1L, DNMT1, EEF1D, EGFR, EPHB3, ERBB2, ERP29, ETFA, ETFDH, EZR, FAF1, FARP2, FASN, FLOT2, FN1, GARS, GNAI3, GRB2, HSPA1A, HTRA2, ILK, IMPA1, ITPR1, KMT2D, LAMP1, LAMTOR1, LARP1, MAP2K2, MARK2, MFN2, MMAB, MYADM, MYO6, NAMPT, NDRG1, NUP62, OPA1, PAK2, PARK7, PEA15, PHB, PHIP, PPIF, PRKD1, PRMT5, PRPF19, PTPN11, PTPRJ, RAB7A, RAP1A, RBFOX2, RHOA, RHOT1, RNF213, RNMT, RPL10, RPS3, RPS6, RPSA, RRAS, SDCBP, SFN, SHC1, SLC35B2, SLC44A2, SLC9A1, SMARCB1, SOD2, SRI, STRN, SYNJ2BP, TKFC, TMBIM1, TOLLIP, TOM1L1, TOM1L2, TP53BP1, TPD52L1, TRIM25, TSG101, UBE2V1, UFL1, VIMP, WFS1, WNK1, ZC3HAV1] |
|  | GO 0007166 cell surface receptor signaling pathway | 46.0E-6 | 730.0E-6 | | 1.0E-6 | 4.8E-6 | [4, 5] | | 4.15 | 38.00 | | [AAK1, AGR2, BAX, CDC37, CTNNB1, DNAJA3, EGFR, EPHB3, ERBB2, ETFA, ETFDH, EZR, FASN, GARS, GRB2, HSPA1A, HTRA2, ILK, NAMPT, NUP62, PAK2, PARK7, PEA15, PHIP, PRKD1, PTPN11, PTPRJ, RAB7A, RHOA, RNF213, RNMT, RPL10, RPSA, SDCBP, STRN, SYNJ2BP, TMBIM1, TSG101] |
|  | GO 0006468 protein phosphorylation | 2.5E-3 | 14.0E-3 | | 1.0E-6 | 4.8E-6 | [6, 7] | | 4.93 | 46.00 | | [AAK1, ADAR, ARL6IP5, ATP2B4, BCCIP, BRD4, CDK11B, CDK5RAP3, CENPE, CTSH, DDRGK1, DLG1, DNAJA3, DNAJC3, EGFR, EPHB3, ERBB2, ERP29, ETFA, ETFDH, EZR, FN1, ILK, MAP2K2, MARK2, MMAB, MYADM, NBN, PAK2, PARK7, PHB, PPP2CA, PRKACB, PRKD1, PTPRJ, RHOA, RPS3, RRAS, SDCBP, SHC1, SLK, SYNJ2BP, TOM1L1, TPD52L1, TSG101, WNK1] |
|  | GO 0006091 generation of precursor metabolites and energy | 9.7E-3 | 42.0E-3 | | 890.0E-6 | 1.5E-3 | [3] | | 15.29 | 13.00 | | [ACO2, ALDOA, COA6, CROT, ETFDH, NDUFS1, PARK7, PDHA1, PDHB, PFKP, STBD1, TRAP1, VCP] |
|  | GO 0009060 aerobic respiration | 2.6E-3 | 15.0E-3 | | 890.0E-6 | 1.5E-3 | [5, 6] | | 44.44 | 4.00 | | [ACO2, PDHA1, PDHB, VCP] |
|  | GO 0006085 acetyl-CoA biosynthetic process | 190.0E-6 | 2.1E-3 | | 890.0E-6 | 1.5E-3 | [6, 7] | | 55.56 | 5.00 | | [ACAT1, ACLY, MLYCD, PDHA1, PDHB] |
|  | GO 0008104 protein localization | 390.0E-9 | 34.0E-6 | | 8.8E-12 | 360.0E-12 | [3] | | 11.25 | 121.00 | | [AAK1, ACSL3, ADAR, AGR2, AIP, ATP1B3, BAG6, BCAP31, CCT2, CCT4, CCT5, CCT6A, CCT7, CCT8, CDH1, CDK5RAP3, CHCHD4, CHERP, CIB1, COG3, CTNNB1, CYB5R1, DCP1A, DDX1, DDX42, DLG1, DNAJB2, DNAJC1, DNM1L, DNM2, EGFR, EIF5A, EPT1, ERBB2, ERP29, EZR, FAF1, FLOT2, FN1, GBF1, GCC2, GDAP1, GDI1, GNL3, GNL3L, HID1, HOOK1, HSP90AA1, HSPA4, HTRA2, HUWE1, IST1, JUP, LAMP1, LAMTOR1, LMAN1, LONP2, MACF1, MFN2, MMAB, MPP7, MTCH2, MYADM, MYH9, MYO18A, NEDD4L, NPEPPS, NPLOC4, NSF, OS9, PACSIN2, PARK7, PEX13, PINX1, PKP3, PMPCA, PPFIA1, PPIA, PPT1, PREB, PRPF19, PSMB7, RAB10, RAB11B, RAB3GAP2, RAB7A, RAB9A, RAP1A, REST, RPL10, RUVBL1, SAMM50, SCARB2, SCRIB, SEC61A1, SEC63, SIN3A, SLC9A1, SNIP1, SNX1, SNX27, SRP54, STXBP1, TAP1, TCP1, TIMM21, TMBIM1, TOLLIP, TOMM34, TOR1A, TP53BP1, U2AF1, UBAC2, UFL1, VCL, VCP, VIMP, VTI1B, WDR46, XPO7, ZW10] |
|  | GO 0010256 endomembrane system organization | 9.0E-6 | 230.0E-6 | | 8.8E-12 | 360.0E-12 | [3] | | 14.68 | 43.00 | | [ACSL3, AGR2, ANKLE2, ATP1B3, BAG6, BNIP1, CDH1, CIB1, COG2, COG3, COG4, DLG1, DNM2, EZR, FLOT2, GBF1, GCC2, GOLGA5, HOOK1, JUP, LEMD2, LMAN1, MACF1, MYADM, MYO18A, MYO5A, PACSIN2, PACSIN3, PKP3, PPFIA1, PRKD1, PRMT5, RAB10, RAB2A, RAB3GAP2, RTN4, SEC61A1, STX18, STXBP1, TMBIM1, TMED5, VTI1B, ZW10] |
|  | GO 0051649 establishment of localization in cell | 540.0E-9 | 33.0E-6 | | 8.8E-12 | 360.0E-12 | [3] | | 11.90 | 97.00 | | [ACTN4, ADAR, AIP, AP1G1, ATP5O, BCAP31, BET1L, BORCS5, CDH1, CENPE, CHCHD4, CHERP, CHMP1A, CIB1, COG3, CYB5R1, DCTN1, DLG1, DYNC1H1, EGFR, EIF5A, EPS15, EPT1, ERBB2, EZR, FAF1, FYTTD1, GBF1, GCC2, GDAP1, GDI1, GOSR1, HID1, HOOK1, HSP90AA1, HSPA4, HTRA2, HUWE1, JUP, KIF2C, LAMP1, LMAN1, LONP2, MAP4, MFN2, MYADM, MYO5A, NPEPPS, NPLOC4, NSF, NUP133, NUP153, OS9, PAFAH1B1, PARK7, PDCD6IP, PEX13, PINX1, PMPCA, PREB, PSMB7, PTPN23, RAB2A, RAB3B, RAB7A, RHOT1, RPL10, RUVBL1, SAMM50, SCARB2, SEC61A1, SEC63, SLC9A1, SNIP1, SNX1, SNX2, SNX27, SNX5, SPAG9, SRI, SRP54, STX18, TAP1, TBC1D5, TIMM21, TOMM34, TOR1A, U2AF1, UBAC2, VAMP7, VCP, VIMP, VPS33A, VPS35, WDR46, XPO7, ZW10] |
|  | GO 0090150 establishment of protein localization to membrane | 280.0E-6 | 2.8E-3 | | 8.8E-12 | 360.0E-12 | [4, 5, 6] | | 15.63 | 25.00 | | [ACSL3, AGR2, BAG6, CDH1, CIB1, DLG1, ERBB2, EZR, FLOT2, GCC2, GDI1, HSP90AA1, HSPA4, JUP, MACF1, MYADM, PKP3, PPFIA1, RAB10, RAB11B, RAB3GAP2, SAMM50, SEC61A1, SEC63, TMBIM1] |
|  | GO 0006605 protein targeting | 1.7E-3 | 11.0E-3 | | 8.8E-12 | 360.0E-12 | [5, 6, 7] | | 11.94 | 40.00 | | [ADAR, AIP, CDH1, CHCHD4, CHERP, CIB1, CYB5R1, EGFR, EPT1, ERBB2, FAF1, GCC2, GDAP1, GDI1, HSP90AA1, HSPA4, HTRA2, HUWE1, JUP, LONP2, MFN2, MYADM, NPEPPS, PEX13, PMPCA, PSMB7, RAB7A, RPL10, RUVBL1, SAMM50, SCARB2, SEC61A1, SEC63, SLC9A1, SNIP1, SRP54, TIMM21, TOMM34, U2AF1, WDR46] |
|  | GO 1902580 single-organism cellular localization | 1.8E-3 | 11.0E-3 | | 8.8E-12 | 360.0E-12 | [3] | | 11.24 | 50.00 | | [ACSL3, ACTN4, ADAR, AGR2, ATP1B3, ATP5O, BAG6, BORCS5, CDH1, CHCHD4, CHERP, CIB1, DLG1, DYNC1H1, EGFR, ERBB2, EZR, FAF1, FLOT2, GCC2, GDI1, HSP90AA1, HSPA4, JUP, LAMP1, MACF1, MAP4, MYADM, MYO5A, PAFAH1B1, PEX13, PKP3, PPFIA1, RAB10, RAB11B, RAB3GAP2, RHOT1, RPL10, SAMM50, SEC61A1, SEC63, SLC9A1, STXBP1, TAP1, TIMM21, TMBIM1, TOR1A, VAMP7, VTI1B, ZW10] |
|  | GO 0006810 transport | 100.0E-6 | 1.3E-3 | | 100.0E-6 | 270.0E-6 | [3] | | 9.49 | 164.00 | | [AAK1, ABCB6, ABCC4, ACSL3, ACTN4, ADAR, AHCYL1, AHNAK, AIP, AP1G1, ATP1B3, ATP2A2, ATP2B4, ATP5B, ATP5O, ATP7A, ATXN2, BAX, BCAP31, BET1L, BORCS5, CDH1, CHCHD4, CHERP, CHMP1A, CIB1, COG2, COG3, COG4, COPA, CTNNB1, CXADR, CYB5R1, DCTN1, DLG1, DNAJC1, DNM1L, DNM2, EEA1, EGFR, EHD4, EIF5A, EPS15, EPT1, ERBB2, ERP29, EZR, FAF1, FLVCR1, FN1, FYTTD1, GBF1, GCC2, GDAP1, GDI1, GOLGA5, GOSR1, GRB2, HID1, HOOK1, HSP90AA1, HSPA1A, HSPA4, HTRA2, HUWE1, JUP, LAMP1, LAMTOR1, LMAN1, LONP2, LRRC8E, MACF1, MFN2, MICU2, MYADM, MYH9, MYO18A, MYO5A, MYO6, NEDD4L, NPEPPS, NPLOC4, NSF, NUP133, NUP153, OS9, OSBPL8, PACSIN2, PARK7, PDCD6IP, PEA15, PEX13, PIK3C2A, PMPCA, PNPT1, PPIA, PPIF, PPT1, PREB, PSMB7, PTPN11, PTPN23, RAB10, RAB11B, RAB2A, RAB3B, RAB7A, RAB9A, RALA, RAP1A, REST, RHOT1, RPL10, RPSA, RUVBL1, SAMM50, SCARB2, SCYL1, SDCBP, SEC61A1, SEC63, SLC25A17, SLC25A24, SLC27A2, SLC30A5, SLC35B2, SLC39A6, SLC9A1, SNIP1, SNX1, SNX2, SNX27, SNX5, SPAG9, SRI, SRP54, STEAP2, STX12, STX18, STXBP1, STXBP2, STXBP3, TAP1, TBC1D5, TIMM21, TMEM115, TMEM165, TNPO1, TOMM34, TOR1A, TSG101, U2AF1, UBAC2, VAMP7, VCP, VIMP, VPS33A, VPS35, VTA1, WDR46, WFS1, XPO7, YWHAE, ZW10] |
|  | GO 0072657 protein localization to membrane | 3.0E-3 | 16.0E-3 | | 8.8E-12 | 360.0E-12 | [4, 5] | | 13.02 | 28.00 | | [ACSL3, AGR2, ATP1B3, BAG6, CDH1, CIB1, DLG1, ERBB2, EZR, FLOT2, GCC2, GDI1, HSP90AA1, HSPA4, JUP, MACF1, MYADM, PKP3, PPFIA1, RAB10, RAB11B, RAB3GAP2, SAMM50, SEC61A1, SEC63, STXBP1, TMBIM1, VTI1B] |
|  | GO 0061024 membrane organization | 820.0E-9 | 43.0E-6 | | 8.8E-12 | 360.0E-12 | [3] | | 14.29 | 57.00 | | [ACSL3, AGR2, ANKLE2, ATP1B3, BAG6, BAX, BNIP1, CD9, CDH1, CIB1, DLG1, DNM1L, DNM2, EEA1, ERBB2, EZR, FLOT2, GBF1, GCC2, GDAP1, GDI1, HSP90AA1, HSPA1A, HSPA4, IMMT, JUP, LEMD2, MACF1, MFN1, MFN2, MYADM, MYO18A, OPA1, PACSIN2, PACSIN3, PDCD6IP, PI4KA, PKP3, PPFIA1, PPIF, PPT1, RAB10, RAB11B, RAB3GAP2, RAB7A, RHOA, RHOT1, SAMM50, SEC61A1, SEC63, STX18, STXBP1, TMBIM1, TSG101, VAMP7, VTA1, VTI1B] |
|  | GO 0044802 single-organism membrane organization | 5.0E-6 | 150.0E-6 | | 8.8E-12 | 360.0E-12 | [3, 4] | | 14.08 | 50.00 | | [ACSL3, AGR2, ANKLE2, ATP1B3, BAG6, BAX, BNIP1, CD9, CDH1, CIB1, DLG1, DNM1L, DNM2, EEA1, ERBB2, EZR, FLOT2, GCC2, GDAP1, GDI1, HSP90AA1, HSPA1A, HSPA4, IMMT, JUP, LEMD2, MACF1, MFN1, MFN2, MYADM, OPA1, PACSIN2, PACSIN3, PKP3, PPFIA1, PPIF, PPT1, RAB10, RAB11B, RAB3GAP2, RAB7A, RHOT1, SAMM50, SEC61A1, SEC63, STX18, STXBP1, TMBIM1, VAMP7, VTI1B] |
|  | GO 0072599 establishment of protein localization to endoplasmic reticulum | 1.0E-3 | 7.8E-3 | | 78.0E-6 | 230.0E-6 | [5, 6, 7] | | 41.67 | 5.00 | | [RAB10, RAB3GAP2, SEC61A1, SEC63, SRP54] |
|  | GO 0016197 endosomal transport | 2.6E-3 | 15.0E-3 | | 100.0E-6 | 270.0E-6 | [5] | | 13.79 | 24.00 | | [AP1G1, BET1L, DCTN1, EHD4, EPS15, EZR, GBF1, GCC2, GOSR1, HOOK1, LMAN1, PTPN23, RAB10, RAB11B, RAB7A, SNX1, SNX2, SNX27, SNX5, SPAG9, TBC1D5, VAMP7, VPS33A, VPS35] |
|  | GO 0048193 Golgi vesicle transport | 50.0E-6 | 770.0E-6 | | 100.0E-6 | 270.0E-6 | [5] | | 19.23 | 20.00 | | [AP1G1, BET1L, COG2, COG3, COG4, EPS15, GBF1, GCC2, GOLGA5, LAMP1, LMAN1, MYO18A, MYO5A, RAB2A, SCYL1, SNX1, STEAP2, STX18, TMEM115, ZW10] |
|  | GO 0042147 retrograde transport, endosome to Golgi | 500.0E-6 | 4.5E-3 | | 100.0E-6 | 270.0E-6 | [5, 6] | | 20.63 | 13.00 | | [BET1L, DCTN1, GBF1, GCC2, GOSR1, LMAN1, RAB7A, SNX1, SNX2, SNX5, SPAG9, TBC1D5, VPS35] |
|  | GO 0045184 establishment of protein localization | 15.0E-6 | 360.0E-6 | | 8.8E-12 | 360.0E-12 | [3, 4] | | 11.30 | 91.00 | | [ACSL3, ADAR, AGR2, AIP, BAG6, BCAP31, CCT2, CCT4, CCT5, CCT6A, CCT7, CCT8, CDH1, CHCHD4, CHERP, CIB1, CYB5R1, DLG1, DNAJC1, DNM1L, DNM2, EGFR, EIF5A, EPT1, ERBB2, ERP29, EZR, FAF1, FLOT2, FN1, GCC2, GDAP1, GDI1, HID1, HOOK1, HSP90AA1, HSPA4, HTRA2, HUWE1, IST1, JUP, LAMP1, LMAN1, LONP2, MACF1, MFN2, MYADM, MYH9, MYO18A, NPEPPS, NPLOC4, NSF, OS9, PARK7, PEX13, PKP3, PMPCA, PPFIA1, PPIA, PPT1, PREB, PSMB7, RAB10, RAB11B, RAB3GAP2, RAB7A, RAP1A, REST, RPL10, RUVBL1, SAMM50, SCARB2, SEC61A1, SEC63, SLC9A1, SNIP1, SNX1, SNX27, SRP54, TAP1, TCP1, TIMM21, TMBIM1, TOMM34, TOR1A, U2AF1, UBAC2, VCP, VIMP, WDR46, XPO7] |
|  | GO 0007030 Golgi organization | 71.0E-6 | 1.0E-3 | | 8.8E-12 | 360.0E-12 | [4] | | 22.39 | 15.00 | | [COG2, COG3, COG4, GBF1, GCC2, GOLGA5, LMAN1, MYO18A, MYO5A, PRKD1, PRMT5, RAB2A, STX18, TMED5, ZW10] |
|  | GO 0034613 cellular protein localization | 12.0E-9 | 1.9E-6 | | 8.8E-12 | 360.0E-12 | [4] | | 12.61 | 102.00 | | [ACSL3, ADAR, AGR2, AIP, ATP1B3, BAG6, BCAP31, CCT2, CCT4, CCT5, CCT6A, CCT7, CCT8, CDH1, CDK5RAP3, CHCHD4, CHERP, CIB1, COG3, CTNNB1, CYB5R1, DCP1A, DDX1, DLG1, EGFR, EIF5A, EPT1, ERBB2, EZR, FAF1, FLOT2, GBF1, GCC2, GDAP1, GDI1, GNL3, GNL3L, HID1, HOOK1, HSP90AA1, HSPA4, HTRA2, HUWE1, JUP, LAMP1, LAMTOR1, LMAN1, LONP2, MACF1, MFN2, MMAB, MTCH2, MYADM, NEDD4L, NPEPPS, NPLOC4, NSF, OS9, PACSIN2, PARK7, PEX13, PINX1, PKP3, PMPCA, PPFIA1, PREB, PRPF19, PSMB7, RAB10, RAB11B, RAB3GAP2, RAB7A, RPL10, RUVBL1, SAMM50, SCARB2, SEC61A1, SEC63, SIN3A, SLC9A1, SNIP1, SNX1, SNX27, SRP54, STXBP1, TAP1, TCP1, TIMM21, TMBIM1, TOLLIP, TOMM34, TOR1A, TP53BP1, U2AF1, UBAC2, VCL, VCP, VIMP, VTI1B, WDR46, XPO7, ZW10] |
|  | GO 0042127 regulation of cell proliferation | 9.8E-3 | 42.0E-3 | | 9.8E-3 | 10.0E-3 | [3, 4] | | 4.83 | 32.00 | | [AIMP1, CD276, CHERP, CIB1, CTNNB1, DNAJA3, EGFR, EIF5A, ERBB2, FN1, FTH1, GDF15, JUP, KMT2D, MYDGF, NACC1, NUP62, PHB, PRKD1, PTPRJ, REST, RPL10, RPRD1B, RPS15A, RPS4X, SDCBP, SOD2, STRN, SYNJ2BP, TES, TP53BP1, UFL1] |
|  | GO 0019058 viral life cycle | 7.0E-3 | 33.0E-3 | | 7.0E-3 | 8.0E-3 | [4, 5] | | 12.32 | 26.00 | | [ADAR, DDX6, EEA1, EIF3A, EIF3B, EIF3D, EIF3F, EIF3G, EIF6, EPS15, IST1, LARP1, PDCD6IP, PI4KA, PPIA, PPIE, RAB7A, REST, SMARCB1, TAP1, TMPRSS2, TRIM25, TSG101, VCP, VTA1, ZC3HAV1] |
|  | GO 0006091 generation of precursor metabolites and energy | 9.7E-3 | 42.0E-3 | | 8.2E-6 | 30.0E-6 | [3] | | 15.29 | 13.00 | | [ACO2, ALDOA, COA6, CROT, ETFDH, NDUFS1, PARK7, PDHA1, PDHB, PFKP, STBD1, TRAP1, VCP] |
|  | GO 0055114 oxidation-reduction process | 46.0E-6 | 740.0E-6 | | 8.2E-6 | 30.0E-6 | [3] | | 15.87 | 30.00 | | [ABCD3, ACAA1, ACADM, ACO2, ALDH3A2, BLVRA, CBR1, COA6, CROT, DHRS4, ETFA, ETFDH, GCDH, GRHPR, LONP2, MARC1, MLYCD, NDUFS1, PARK7, PDHA1, PDHB, PECR, PGD, PRDX5, SLC25A17, SLC27A2, SQRDL, STBD1, TRAP1, VCP] |
|  | GO 0009116 nucleoside metabolic process | 3.6E-3 | 19.0E-3 | | 8.2E-6 | 30.0E-6 | [4, 5, 6] | | 15.38 | 16.00 | | [ACAT1, AK3, ALDOA, ATP5B, ATP5O, COA6, COASY, GNAI3, HSPA1A, MACROD1, NDUFS1, OLA1, PARK7, PFKP, QTRT1, VCP] |
|  | GO 0009060 aerobic respiration | 2.6E-3 | 15.0E-3 | | 8.2E-6 | 30.0E-6 | [5, 6] | | 44.44 | 4.00 | | [ACO2, PDHA1, PDHB, VCP] |
|  | GO 0009141 nucleoside triphosphate metabolic process | 6.8E-3 | 33.0E-3 | | 8.2E-6 | 30.0E-6 | [5, 6, 7] | | 16.46 | 13.00 | | [AK1, AK3, ALDOA, ATP5B, ATP5O, COA6, GNAI3, HSPA1A, NDUFS1, OLA1, PARK7, PFKP, VCP] |
|  | GO 0042455 ribonucleoside biosynthetic process | 2.5E-3 | 14.0E-3 | | 8.2E-6 | 30.0E-6 | [6, 7, 8] | | 25.93 | 7.00 | | [ACAT1, ALDOA, ATP5B, ATP5O, COASY, QTRT1, VCP] |
|  | GO 0009205 purine ribonucleoside triphosphate metabolic process | 2.2E-3 | 13.0E-3 | | 8.2E-6 | 30.0E-6 | [7, 8, 9] | | 18.46 | 12.00 | | [AK3, ALDOA, ATP5B, ATP5O, COA6, GNAI3, HSPA1A, NDUFS1, OLA1, PARK7, PFKP, VCP] |
|  | GO 0006754 ATP biosynthetic process | 8.6E-3 | 39.0E-3 | | 8.2E-6 | 30.0E-6 | [8, 9, 10, 11] | | 33.33 | 4.00 | | [ALDOA, ATP5B, ATP5O, VCP] |
|  | GO 0048518 positive regulation of biological process | 890.0E-6 | 6.9E-3 | | 43.0E-6 | 130.0E-6 | [1, 2, 3] | | 6.07 | 186.00 | | [AAK1, ABHD14B, ACSL3, ACTN4, ACTR3, ADAR, AGR2, AHCYL1, ANKLE2, AP1G1, ARL6IP5, ATP1B3, ATP2B4, ATP7A, BAG6, BAX, BCAP31, BNIP3L, BRD4, C14orf166, CC2D1A, CCT2, CCT4, CCT5, CCT6A, CCT7, CCT8, CD276, CDH1, CDK5RAP3, CENPE, CHERP, CIB1, CNOT1, CTNNB1, CTSH, CYB5R1, CYFIP1, DDRGK1, DLG1, DNAJA3, DNAJB2, DNM1L, DNMT1, EEF1D, EEF1E1, EEF2, EGFR, EIF5A, EPT1, ERBB2, ERP29, ETFA, ETFDH, EZR, FAF1, FASN, FLOT2, FN1, FXR1, GARS, GDF15, GIPC1, GNL3, GNL3L, GRB2, HSPA1A, HTRA2, HUWE1, IDE, ILK, IST1, JUP, KMT2D, LAMP1, LAMTOR1, LARP1, LMAN1, MACF1, MAP2K2, MAPT, MARK2, MLYCD, MMAB, MPP7, MTPN, MYADM, MYDGF, MYO18A, MYO6, NACC1, NAMPT, NBN, NCOA6, NDRG1, NDUFA2, NEDD4L, NOP10, NPEPPS, NSF, NUP62, PACSIN3, PAK2, PARK7, PDCD6IP, PELP1, PFN1, PHB, PHIP, PINX1, PNPLA2, PNPT1, PPIA, PPIE, PPP2CA, PPT1, PRDX5, PRKD1, PRMT1, PRMT5, PSMB7, PTPN11, PTPN23, PTPRJ, QARS, RAB3B, RAB3GAP2, RAB7A, RAB9A, RALA, RBM5, REST, RHOA, RNMT, RPL10, RPRD1B, RPS15A, RPS3, RPS4X, RPS6, RPSA, RRAS, RUVBL1, SCRIB, SDCBP, SEPT9, SHC1, SHTN1, SIN3A, SLC35B2, SLC44A2, SLC9A1, SMARCB1, SMARCC2, SNIP1, SPAG9, SPEN, SRI, STRN3, STX18, SUPV3L1, TBC1D5, TCP1, TFAM, TKFC, TMPRSS2, TNPO1, TOM1L1, TOR1A, TP53BP1, TPD52L1, TRIM25, TRIOBP, TSG101, U2AF1, UBE2V1, UFL1, USP47, UTRN, VAMP7, VCP, WASF2, WDR46, WFS1, YTHDF2, ZC3HAV1] |
|  | GO 0048519 negative regulation of biological process | 1.4E-3 | 10.0E-3 | | 43.0E-6 | 130.0E-6 | [1, 2, 3] | | 5.89 | 142.00 | | [ABCE1, ACTN1, ACTN4, ADAR, AGR2, AIMP1, ANKLE2, ANXA7, ARFGEF3, ATP2B4, ATXN2, BNIP3L, BRD4, CAST, CDC5L, CDH1, CDK5RAP3, CHERP, CHMP1A, CIB1, CNOT1, CTNNB1, CTSA, DLG1, DNAJA3, DNAJB2, DNAJC3, DNM2, DNMT1, EGFR, EIF6, ENO1, ERP29, ETF1, EZR, FAF1, FLOT2, FN1, FTH1, FXR1, GDI1, GNL3L, HSPA1A, ITPR1, LIG3, MAP2K2, MAP4, MFN2, MMAB, MTPN, MYADM, MYH9, MYO18A, MYO1D, NACC1, NAE1, NBN, NEDD4L, NUP153, NUP62, OPA1, OS9, OSBPL8, OTUB1, PAK2, PARK7, PDCD4, PEA15, PFN1, PHB, PHF21A, PHIP, PINX1, PNPLA2, PNPT1, PPFIA1, PPIF, PPP2CA, PPT1, PRCC, PRDX5, PRKD1, PRMT1, PRPF19, PTPN23, PTPRJ, RAB7A, RBFOX2, RCOR1, REST, RHOA, RNF213, RNMT, RPA2, RPL10, RPS3, RPSA, RRP1B, RTN4, SAP130, SCRIB, SDCBP, SERPINB6, SET, SFN, SIGIRR, SIN3A, SLC27A1, SMARCB1, SMARCC2, SNIP1, SOD2, SPEN, SRI, STRN, STRN3, STXBP3, SUPV3L1, SYNJ2BP, TBC1D30, TES, TKFC, TMBIM1, TOM1L1, TOM1L2, TP53BP1, TRAP1, TSG101, UBAC2, UBE2V1, UBXN1, UFL1, USP47, VIMP, VPS35, WASF2, WFS1, WNK1, YEATS2, YWHAE, ZC3HAV1, ZW10] |
|  | GO 0019222 regulation of metabolic process | 17.0E-6 | 370.0E-6 | | 43.0E-6 | 130.0E-6 | [2, 3] | | 5.65 | 165.00 | | [ABCE1, ABHD14B, ACSL3, ACTN4, ADAR, AGR2, AHCYL1, ANKLE2, ANXA7, ARFGEF3, ARL6IP5, ATP2B4, ATP7A, ATXN2, BAG6, BAX, BCAP31, BCCIP, BRD4, C14orf166, CCT2, CCT4, CCT5, CCT6A, CCT7, CCT8, CD276, CDH1, CDK11B, CDK5RAP3, CENPE, CHMP1A, CIB1, CNOT1, CTNNB1, CTSH, DDRGK1, DLG1, DNAJA3, DNAJB2, DNAJC3, DNMT1, EEF2, EGFR, EIF2A, EIF3B, EIF4G2, EIF5, EIF5B, EIF6, ENO1, ERBB2, ERP29, ETF1, ETFA, ETFDH, EZR, FAF1, FLOT2, FN1, FXR1, GARS, GNL3, GNL3L, GRB2, HSP90AA1, HSPA1A, HTRA2, ILK, IST1, JUP, KMT2D, LAMTOR1, LIG3, LONP2, MAP2K2, ME2, MLYCD, MMAB, MYADM, MYDGF, MYO1D, MYO6, NACC1, NAMPT, NBN, NCOA6, NSF, NUP62, OS9, OTUB1, PACSIN3, PAK2, PARK7, PDCD4, PELP1, PHB, PHF21A, PHIP, PINX1, PNPLA2, PNPT1, PPIE, PPP2CA, PQBP1, PRDX5, PRKD1, PTPRJ, QARS, RAB3GAP2, RAB7A, RBFOX2, RBM5, RCOR1, REST, RHOA, RNMT, RPA2, RPRD1B, RPS3, RPS4X, RRAS, RRP1B, SAP130, SCRIB, SDCBP, SERPINB6, SET, SFN, SHC1, SIGIRR, SIN3A, SLC27A1, SLC9A1, SMARCB1, SMARCC2, SNIP1, SOD2, SPEN, STRN3, SUPV3L1, SYNJ2BP, TBC1D5, TCP1, TFAM, TOM1L1, TP53BP1, TPD52L1, TRAP1, TRIM25, TSG101, UBAC2, UBE2V1, UBXN1, UCHL5, UFL1, USP47, VCP, VIMP, WFS1, WNK1, YEATS2, YTHDF2, YWHAE, ZC3HAV1] |
|  | GO 0048583 regulation of response to stimulus | 3.5E-3 | 18.0E-3 | | 43.0E-6 | 130.0E-6 | [2, 3] | | 5.70 | 100.00 | | [AAK1, ACTN4, AGR2, ANXA5, AP1G1, ARHGDIA, ARL6IP5, ATP2B4, BAG6, BAX, BCAP31, BRD4, CC2D1A, CDC37, CDK5RAP3, CIB1, CNOT1, CTSH, DDRGK1, DLG1, DNAJA3, DNAJC3, DNM1L, EEF1D, EEF1E1, EGFR, ELMOD2, ERBB2, ERP29, ETFA, ETFDH, EZR, FAF1, FLOT2, FN1, HSPA1A, HTRA2, ILK, IST1, ITPR1, KMT2D, LAMP1, LAMTOR1, MAP2K2, MDH1, MFN2, MMAB, MTPN, MYADM, MYDGF, NUP62, OPA1, OS9, OTUB1, PAK2, PARK7, PEA15, PEX13, PHB, PHIP, PNPT1, PPIF, PRKD1, PRMT5, PTPN11, PTPRJ, RAB7A, RHOA, RNF213, RNMT, RPA2, RPL10, RPS3, RPSA, RRAS, SDCBP, SHC1, SIN3A, SLC35B2, SLC44A2, SLC9A1, SMARCB1, SNRPB, SOD2, SRI, SYNJ2BP, TKFC, TMBIM1, TP53BP1, TPD52L1, TRIM25, TSG101, UBAC2, UBE2V1, UBXN1, UFL1, VAMP7, VIMP, WFS1, ZC3HAV1] |
|  | GO 0050794 regulation of cellular process | 1.6E-6 | 70.0E-6 | | 43.0E-6 | 130.0E-6 | [2, 3] | | 6.13 | 292.00 | | [AAK1, ABCE1, ABHD14B, ACSL3, ACTN1, ACTN4, ACTR3, ADAR, AGR2, AHCYL1, AIMP1, ALDOA, ANKLE2, ANXA5, AP1G1, ARFGEF3, ARHGDIA, ARL6IP5, ATP1B3, ATP2A2, ATP2B4, ATP6V0A1, ATXN2, ATXN2L, BAG6, BAIAP2, BAX, BCAP31, BCCIP, BET1L, BNIP3L, BRD4, C14orf166, CAPZB, CAST, CC2D1A, CCT2, CCT4, CCT5, CCT6A, CCT7, CCT8, CD276, CDC27, CDC37, CDC5L, CDH1, CDK11B, CDK5RAP3, CENPE, CHERP, CHMP1A, CIB1, CNOT1, CTNNB1, CTSA, CTSH, CYB5R1, CYFIP1, DDRGK1, DLG1, DNAJA3, DNAJB2, DNAJC1, DNAJC3, DNM1L, DNM2, DNMT1, DSG2, DSP, EEF1D, EEF1E1, EEF2, EGFR, EIF2A, EIF3B, EIF4G2, EIF5, EIF5A, EIF5B, EIF6, ELMOD2, ENO1, EPHB3, EPT1, ERBB2, ERP29, ETF1, ETFA, ETFDH, EZR, FAF1, FARP2, FASN, FITM2, FLOT2, FN1, FTH1, FXR1, GARS, GBF1, GDF15, GDI1, GIPC1, GNAI3, GNL3, GNL3L, GRB2, HSP90AA1, HSPA1A, HTRA2, HUWE1, IDE, ILK, IMPA1, IST1, ITPR1, JUP, KIF2C, KMT2D, LAMP1, LAMTOR1, LARP1, LIG3, LMAN1, LONP2, MACF1, MAP1S, MAP2K2, MAP4, MAPT, MARK2, MDH1, ME2, MFN2, MLYCD, MMAB, MPP7, MTPN, MYADM, MYDGF, MYH9, MYO18A, MYO1D, MYO5A, MYO6, NACC1, NAE1, NAMPT, NBN, NCOA6, NDRG1, NEDD4L, NPEPPS, NSF, NUP62, OPA1, OS9, OTUB1, PACSIN3, PAK2, PARK7, PDCD4, PDCD6IP, PDZD8, PEA15, PELP1, PEX13, PFN1, PHB, PHF21A, PHIP, PINX1, PNPLA2, PNPT1, PPFIA1, PPIA, PPIE, PPIF, PPP2CA, PPT1, PQBP1, PRCC, PRDX5, PRKD1, PRMT1, PRMT5, PRPF19, PSMB7, PTPN11, PTPN23, PTPRJ, QARS, RAB3B, RAB3GAP2, RAB7A, RAB9A, RALA, RAP1A, RBFOX2, RBM5, RCOR1, REST, RHOA, RHOT1, RNF213, RNMT, RPA2, RPL10, RPRD1B, RPS15A, RPS3, RPS4X, RPS6, RPSA, RRAS, RRP1B, RTN4, RUVBL1, SAP130, SCRIB, SDCBP, SEPT9, SERPINB6, SET, SFN, SHC1, SHTN1, SIGIRR, SIN3A, SLC25A24, SLC27A1, SLC35B2, SLC44A2, SLC9A1, SLK, SMARCB1, SMARCC2, SNIP1, SNRPB, SOD2, SPAG9, SPEN, SRI, STRN, STRN3, STX18, STXBP3, SUPV3L1, SYNJ2BP, TAP1, TBC1D30, TBC1D5, TCP1, TES, TFAM, TKFC, TMBIM1, TMPRSS2, TOLLIP, TOM1L1, TOM1L2, TOR1A, TP53BP1, TPD52L1, TRAP1, TRIM24, TRIM25, TRIOBP, TSG101, U2AF1, UBAC2, UBE2V1, UBXN1, UCHL5, UFL1, USP47, VAMP7, VCP, VIMP, VTI1B, WASF2, WDR1, WDR46, WFS1, WNK1, YEATS2, YTHDF2, YWHAE, YWHAG, ZC3HAV1, ZW10] |
|  | GO 0006725 cellular aromatic compound metabolic process | 8.7E-3 | 39.0E-3 | | 43.0E-6 | 130.0E-6 | [3] | | 6.13 | 149.00 | | [ABCB6, ABCE1, ABHD14B, ACAT1, ACTN4, ADAR, AHCYL1, AK1, AK3, ALDH6A1, ALDOA, ATP2B4, ATP5B, ATP5O, BAX, BLVRB, BRD4, C14orf166, C9orf142, CAD, CARS, CCT2, CCT4, CCT5, CCT6A, CCT7, CCT8, CDH1, CDK11B, CDK5RAP3, CHMP1A, CIB1, CMTR1, CNOT1, COA6, COASY, CTNNB1, DDRGK1, DDX1, DLG1, DNAJA3, DNMT1, EEA1, EEF1D, EGFR, ENO1, ERBB2, ETF1, EZR, FARSA, FARSB, GARS, GATB, GEMIN5, GNAI3, GNL3, GNL3L, GTF3C2, GUK1, HARS2, HSPA1A, HUWE1, ILK, JUP, KARS, KMT2D, LARS2, LIG3, MACROD1, MAP2K2, MARS2, MCM2, ME2, METTL14, MMAB, MYO6, NACC1, NAMPT, NBN, NCOA6, NDUFS1, NSUN2, NSUN4, NUDT21, NUP62, OLA1, OTUB1, PARK7, PARN, PDCD4, PELP1, PFKP, PGD, PHB, PHF21A, PHIP, PI4KA, PINX1, PNPT1, POLR2E, POLR2G, PON2, PPIE, PQBP1, PRDX5, PRKD1, PRMT5, PRPF19, PURA, QARS, QTRT1, RBFOX2, RBM5, RCOR1, REST, RNMT, RPA2, RPL7, RPRD1B, RPS28, RPS3, RPS6, RTCB, SAP130, SET, SIGIRR, SIN3A, SLC9A1, SMARCB1, SMARCC2, SNIP1, SNRPB, SOD2, SPEN, STRN3, SUPV3L1, TCP1, TFAM, TOM1L1, TP53BP1, TRIM25, TRMU, UBE2V1, UFL1, USP47, VCP, WFS1, YEATS2, ZC3HAV1] |
|  | GO 0009892 negative regulation of metabolic process | 8.7E-3 | 39.0E-3 | | 43.0E-6 | 130.0E-6 | [2, 3, 4] | | 5.58 | 73.00 | | [ABCE1, ADAR, ANKLE2, ANXA7, ARFGEF3, ATP2B4, ATXN2, CDK5RAP3, CHMP1A, CNOT1, CTNNB1, DLG1, DNAJA3, DNAJB2, DNAJC3, DNMT1, EGFR, EIF6, ENO1, ERP29, ETF1, EZR, FLOT2, FXR1, GNL3L, HSPA1A, LIG3, MAP2K2, MMAB, MYADM, MYO1D, NACC1, NBN, OS9, OTUB1, PAK2, PARK7, PDCD4, PHB, PHF21A, PINX1, PRDX5, PTPRJ, RBFOX2, RCOR1, REST, RPS3, RRP1B, SAP130, SDCBP, SERPINB6, SET, SFN, SIN3A, SLC27A1, SMARCB1, SMARCC2, SNIP1, SPEN, STRN3, SYNJ2BP, TP53BP1, TRAP1, UBAC2, UBE2V1, UBXN1, UFL1, USP47, VIMP, WFS1, WNK1, YEATS2, YWHAE] |
|  | GO 0009893 positive regulation of metabolic process | 68.0E-6 | 1.0E-3 | | 43.0E-6 | 130.0E-6 | [2, 3, 4] | | 5.19 | 93.00 | | [ABHD14B, ACSL3, AGR2, ANKLE2, ARL6IP5, ATP2B4, ATP7A, BAG6, BAX, BCAP31, BRD4, C14orf166, CCT2, CCT4, CCT5, CCT6A, CCT7, CCT8, CD276, CDH1, CDK5RAP3, CENPE, CNOT1, CTNNB1, CTSH, DDRGK1, DNAJA3, DNAJB2, DNMT1, EEF2, EGFR, ERBB2, ERP29, ETFA, ETFDH, EZR, FN1, GARS, GNL3, GRB2, HSPA1A, HTRA2, ILK, IST1, KMT2D, MAP2K2, MLYCD, MYDGF, MYO6, NAMPT, NBN, NCOA6, NSF, NUP62, PACSIN3, PAK2, PARK7, PELP1, PHB, PHIP, PNPLA2, PNPT1, PPP2CA, PRDX5, PRKD1, QARS, RAB3GAP2, RAB7A, REST, RHOA, RNMT, RPRD1B, RPS3, RPS4X, SCRIB, SDCBP, SHC1, SIN3A, SLC9A1, SMARCB1, SMARCC2, STRN3, SUPV3L1, TBC1D5, TCP1, TFAM, TOM1L1, TP53BP1, TPD52L1, VCP, WFS1, YTHDF2, ZC3HAV1] |
|  | GO 0046483 heterocycle metabolic process | 8.6E-3 | 39.0E-3 | | 43.0E-6 | 130.0E-6 | [3] | | 6.13 | 148.00 | | [ABCB6, ABCE1, ABHD14B, ACAT1, ACTN4, ADAR, AHCYL1, AK1, AK3, ALDH6A1, ALDOA, ATP2B4, ATP5B, ATP5O, BAX, BLVRB, BRD4, C14orf166, C9orf142, CAD, CARS, CCT2, CCT4, CCT5, CCT6A, CCT7, CCT8, CDH1, CDK11B, CDK5RAP3, CHMP1A, CIB1, CMTR1, CNOT1, COA6, COASY, CTNNB1, DDRGK1, DDX1, DLG1, DNAJA3, DNMT1, EEA1, EEF1D, EGFR, ENO1, ERBB2, ETF1, EZR, FARSA, FARSB, GARS, GATB, GEMIN5, GNAI3, GNL3, GNL3L, GTF3C2, GUK1, HARS2, HSPA1A, HUWE1, ILK, JUP, KARS, KMT2D, LARS2, LIG3, MACROD1, MAP2K2, MARS2, MCM2, ME2, METTL14, MMAB, MYO6, NACC1, NAMPT, NBN, NCOA6, NDUFS1, NSUN2, NSUN4, NUDT21, NUP62, OLA1, OTUB1, PARK7, PARN, PDCD4, PELP1, PFKP, PGD, PHB, PHF21A, PHIP, PI4KA, PINX1, PNPT1, POLR2E, POLR2G, PPIE, PQBP1, PRDX5, PRKD1, PRMT5, PRPF19, PURA, QARS, QTRT1, RBFOX2, RBM5, RCOR1, REST, RNMT, RPA2, RPL7, RPRD1B, RPS28, RPS3, RPS6, RTCB, SAP130, SET, SIGIRR, SIN3A, SLC9A1, SMARCB1, SMARCC2, SNIP1, SNRPB, SOD2, SPEN, STRN3, SUPV3L1, TCP1, TFAM, TOM1L1, TP53BP1, TRIM25, TRMU, UBE2V1, UFL1, USP47, VCP, WFS1, YEATS2, ZC3HAV1] |
|  | GO 0048522 positive regulation of cellular process | 3.8E-3 | 20.0E-3 | | 43.0E-6 | 130.0E-6 | [2, 3, 4] | | 6.14 | 172.00 | | [AAK1, ABHD14B, ACSL3, ACTN4, ACTR3, ADAR, AGR2, ANKLE2, AP1G1, ARL6IP5, ATP1B3, ATP2B4, BAG6, BAX, BCAP31, BNIP3L, BRD4, C14orf166, CC2D1A, CCT2, CCT4, CCT5, CCT6A, CCT7, CCT8, CD276, CDH1, CDK5RAP3, CENPE, CIB1, CNOT1, CTNNB1, CTSH, CYB5R1, CYFIP1, DDRGK1, DLG1, DNAJA3, DNAJB2, DNM1L, DNMT1, EEF1D, EEF1E1, EEF2, EGFR, EIF5A, EPT1, ERBB2, ERP29, ETFA, ETFDH, EZR, FAF1, FASN, FLOT2, FN1, FXR1, GARS, GDF15, GIPC1, GNL3, GRB2, HSPA1A, HTRA2, HUWE1, IDE, ILK, IST1, KMT2D, LAMP1, LAMTOR1, LARP1, LMAN1, MACF1, MAP2K2, MAPT, MARK2, MLYCD, MMAB, MPP7, MYADM, MYDGF, MYO18A, MYO6, NACC1, NAMPT, NBN, NCOA6, NDRG1, NEDD4L, NPEPPS, NSF, NUP62, PACSIN3, PAK2, PARK7, PDCD6IP, PELP1, PFN1, PHB, PHIP, PNPLA2, PNPT1, PPIA, PPIE, PPP2CA, PPT1, PRKD1, PRMT1, PRMT5, PSMB7, PTPN11, PTPN23, PTPRJ, QARS, RAB3B, RAB3GAP2, RAB7A, RAB9A, RALA, RBM5, REST, RHOA, RNMT, RPL10, RPRD1B, RPS15A, RPS3, RPS4X, RPS6, RPSA, RUVBL1, SCRIB, SDCBP, SEPT9, SHC1, SHTN1, SIN3A, SLC35B2, SLC44A2, SLC9A1, SMARCB1, SMARCC2, SNIP1, SPAG9, SPEN, SRI, STRN3, STX18, SUPV3L1, TBC1D5, TCP1, TFAM, TMPRSS2, TOM1L1, TOR1A, TP53BP1, TPD52L1, TRIM25, TRIOBP, TSG101, U2AF1, UBE2V1, UFL1, USP47, VAMP7, VCP, WASF2, WDR46, WFS1, YTHDF2, ZC3HAV1] |
|  | GO 0048523 negative regulation of cellular process | 4.6E-3 | 23.0E-3 | | 43.0E-6 | 130.0E-6 | [2, 3, 4] | | 5.97 | 134.00 | | [ABCE1, ACTN1, ACTN4, ADAR, AGR2, AIMP1, ANKLE2, ARFGEF3, ATP2B4, ATXN2, BNIP3L, BRD4, CAST, CDC5L, CDH1, CDK5RAP3, CHERP, CHMP1A, CIB1, CNOT1, CTNNB1, CTSA, DLG1, DNAJA3, DNAJB2, DNAJC3, DNM2, DNMT1, EGFR, EIF6, ENO1, ERP29, ETF1, EZR, FAF1, FN1, FTH1, FXR1, GDI1, GNL3L, HSPA1A, ITPR1, LIG3, MAP2K2, MAP4, MFN2, MMAB, MTPN, MYADM, MYH9, MYO18A, MYO1D, NACC1, NAE1, NBN, NEDD4L, NUP62, OPA1, OS9, OTUB1, PAK2, PARK7, PDCD4, PEA15, PFN1, PHB, PHF21A, PHIP, PINX1, PNPT1, PPFIA1, PPIF, PPP2CA, PPT1, PRCC, PRDX5, PRKD1, PRMT1, PRPF19, PTPN23, PTPRJ, RAB7A, RBFOX2, RCOR1, REST, RHOA, RNF213, RNMT, RPA2, RPL10, RPS3, RRP1B, RTN4, SAP130, SCRIB, SDCBP, SERPINB6, SET, SFN, SIN3A, SLC27A1, SMARCB1, SMARCC2, SNIP1, SOD2, SPEN, SRI, STRN, STRN3, STXBP3, SUPV3L1, SYNJ2BP, TBC1D30, TES, TKFC, TMBIM1, TOM1L1, TOM1L2, TP53BP1, TRAP1, TSG101, UBAC2, UBE2V1, UBXN1, UFL1, USP47, VIMP, WASF2, WFS1, WNK1, YEATS2, YWHAE, ZC3HAV1, ZW10] |
|  | GO 0006139 nucleobase-containing compound metabolic process | 10.0E-3 | 45.0E-3 | | 43.0E-6 | 130.0E-6 | [3, 4] | | 6.16 | 146.00 | | [ABCE1, ABHD14B, ACAT1, ACTN4, ADAR, AHCYL1, AK1, AK3, ALDH6A1, ALDOA, ATP2B4, ATP5B, ATP5O, BAX, BRD4, C14orf166, C9orf142, CAD, CARS, CCT2, CCT4, CCT5, CCT6A, CCT7, CCT8, CDH1, CDK11B, CDK5RAP3, CHMP1A, CIB1, CMTR1, CNOT1, COA6, COASY, CTNNB1, DDRGK1, DDX1, DLG1, DNAJA3, DNMT1, EEA1, EEF1D, EGFR, ENO1, ERBB2, ETF1, EZR, FARSA, FARSB, GARS, GATB, GEMIN5, GNAI3, GNL3, GNL3L, GTF3C2, GUK1, HARS2, HSPA1A, HUWE1, ILK, JUP, KARS, KMT2D, LARS2, LIG3, MACROD1, MAP2K2, MARS2, MCM2, ME2, METTL14, MMAB, MYO6, NACC1, NAMPT, NBN, NCOA6, NDUFS1, NSUN2, NSUN4, NUDT21, NUP62, OLA1, OTUB1, PARK7, PARN, PDCD4, PELP1, PFKP, PGD, PHB, PHF21A, PHIP, PI4KA, PINX1, PNPT1, POLR2E, POLR2G, PPIE, PQBP1, PRDX5, PRKD1, PRMT5, PRPF19, PURA, QARS, QTRT1, RBFOX2, RBM5, RCOR1, REST, RNMT, RPA2, RPL7, RPRD1B, RPS28, RPS3, RPS6, RTCB, SAP130, SET, SIGIRR, SIN3A, SLC9A1, SMARCB1, SMARCC2, SNIP1, SNRPB, SOD2, SPEN, STRN3, SUPV3L1, TCP1, TFAM, TOM1L1, TP53BP1, TRIM25, TRMU, UBE2V1, UFL1, USP47, VCP, WFS1, YEATS2, ZC3HAV1] |
|  | GO 0009889 regulation of biosynthetic process | 560.0E-6 | 4.9E-3 | | 43.0E-6 | 130.0E-6 | [3, 4] | | 5.46 | 99.00 | | [ABHD14B, ACSL3, ACTN4, ADAR, ATP2B4, BRD4, C14orf166, CCT2, CCT4, CCT5, CCT6A, CCT7, CCT8, CD276, CDH1, CDK5RAP3, CHMP1A, CIB1, CNOT1, CTNNB1, DDRGK1, DLG1, DNAJA3, DNAJC3, DNMT1, EEF2, EGFR, EIF2A, EIF3B, EIF4G2, EIF5, EIF5B, EIF6, ENO1, ERBB2, ETF1, EZR, FXR1, GARS, GNL3, GNL3L, HSPA1A, ILK, JUP, KMT2D, LIG3, MAP2K2, MMAB, MYDGF, MYO6, NACC1, NAMPT, NCOA6, NUP62, PARK7, PDCD4, PELP1, PHB, PHF21A, PHIP, PINX1, PPIE, PRDX5, PRKD1, QARS, RAB3GAP2, RBFOX2, RCOR1, REST, RNMT, RPRD1B, RPS3, RPS4X, SAP130, SET, SIGIRR, SIN3A, SLC27A1, SLC9A1, SMARCB1, SMARCC2, SNIP1, SOD2, SPEN, STRN3, TCP1, TFAM, TOM1L1, TP53BP1, TRAP1, TRIM25, UBE2V1, UFL1, USP47, VCP, VIMP, WFS1, YEATS2, YTHDF2] |
|  | GO 0031323 regulation of cellular metabolic process | 24.0E-6 | 500.0E-6 | | 43.0E-6 | 130.0E-6 | [3, 4] | | 5.63 | 155.00 | | [ABCE1, ABHD14B, ACTN4, ADAR, AHCYL1, ANKLE2, ARFGEF3, ARL6IP5, ATP2B4, ATXN2, BAX, BCAP31, BCCIP, BRD4, C14orf166, CCT2, CCT4, CCT5, CCT6A, CCT7, CCT8, CD276, CDH1, CDK11B, CDK5RAP3, CENPE, CHMP1A, CIB1, CNOT1, CTNNB1, CTSH, DDRGK1, DLG1, DNAJA3, DNAJB2, DNAJC3, DNMT1, EEF2, EGFR, EIF2A, EIF3B, EIF4G2, EIF5, EIF5B, EIF6, ENO1, ERBB2, ERP29, ETF1, ETFA, ETFDH, EZR, FN1, FXR1, GARS, GNL3, GNL3L, GRB2, HSP90AA1, HSPA1A, HTRA2, ILK, IST1, JUP, KMT2D, LAMTOR1, LIG3, LONP2, MAP2K2, ME2, MLYCD, MMAB, MYADM, MYDGF, MYO1D, MYO6, NACC1, NAMPT, NBN, NCOA6, NSF, NUP62, OTUB1, PACSIN3, PAK2, PARK7, PDCD4, PELP1, PHB, PHF21A, PHIP, PINX1, PNPLA2, PNPT1, PPIE, PPP2CA, PQBP1, PRDX5, PRKD1, PTPRJ, QARS, RAB3GAP2, RBFOX2, RBM5, RCOR1, REST, RHOA, RNMT, RPA2, RPRD1B, RPS3, RPS4X, RRAS, RRP1B, SAP130, SCRIB, SDCBP, SERPINB6, SET, SFN, SHC1, SIGIRR, SIN3A, SLC27A1, SLC9A1, SMARCB1, SMARCC2, SNIP1, SOD2, SPEN, STRN3, SUPV3L1, SYNJ2BP, TBC1D5, TCP1, TFAM, TOM1L1, TP53BP1, TPD52L1, TRAP1, TRIM25, TSG101, UBE2V1, UBXN1, UCHL5, UFL1, USP47, VCP, VIMP, WFS1, WNK1, YEATS2, YTHDF2, YWHAE, ZC3HAV1] |
|  | GO 0051171 regulation of nitrogen compound metabolic process | 2.0E-3 | 12.0E-3 | | 43.0E-6 | 130.0E-6 | [3, 4] | | 5.70 | 108.00 | | [ABCE1, ABHD14B, ACSL3, ACTN4, ADAR, AHCYL1, ATP2B4, BAX, BRD4, C14orf166, CCT2, CCT4, CCT5, CCT6A, CCT7, CCT8, CDH1, CDK11B, CDK5RAP3, CHMP1A, CIB1, CNOT1, CTNNB1, DDRGK1, DLG1, DNAJA3, DNAJC3, DNMT1, EEF2, EGFR, EIF2A, EIF3B, EIF4G2, EIF5, EIF5B, EIF6, ENO1, ERBB2, ETF1, EZR, FXR1, GARS, GNL3, GNL3L, HSPA1A, ILK, JUP, KMT2D, LIG3, MAP2K2, ME2, MMAB, MYO6, NACC1, NAMPT, NBN, NCOA6, NUP62, OTUB1, PARK7, PDCD4, PELP1, PHB, PHF21A, PHIP, PINX1, PNPT1, PPIE, PQBP1, PRDX5, PRKD1, QARS, RBFOX2, RBM5, RCOR1, REST, RNMT, RPA2, RPRD1B, RPS3, RPS4X, SAP130, SET, SIGIRR, SIN3A, SLC9A1, SMARCB1, SMARCC2, SNIP1, SOD2, SPEN, STRN3, SUPV3L1, TCP1, TFAM, TOM1L1, TP53BP1, TRAP1, TRIM25, UBE2V1, UFL1, USP47, VCP, VIMP, WFS1, YEATS2, YTHDF2, ZC3HAV1] |
|  | GO 0060255 regulation of macromolecule metabolic process | 27.0E-6 | 550.0E-6 | | 43.0E-6 | 130.0E-6 | [3, 4] | | 5.60 | 152.00 | | [ABCE1, ABHD14B, ACTN4, ADAR, AGR2, AHCYL1, ANKLE2, ANXA7, ARL6IP5, ATP2B4, ATXN2, BAG6, BAX, BCAP31, BCCIP, BRD4, C14orf166, CCT2, CCT4, CCT5, CCT6A, CCT7, CCT8, CD276, CDH1, CDK11B, CDK5RAP3, CENPE, CHMP1A, CIB1, CNOT1, CTNNB1, CTSH, DDRGK1, DLG1, DNAJA3, DNAJB2, DNAJC3, DNMT1, EEF2, EGFR, EIF2A, EIF3B, EIF4G2, EIF5, EIF5B, EIF6, ENO1, ERBB2, ERP29, ETF1, ETFA, ETFDH, EZR, FAF1, FLOT2, FN1, FXR1, GARS, GNL3, GNL3L, HSP90AA1, HSPA1A, HTRA2, ILK, IST1, JUP, KMT2D, LIG3, MAP2K2, MMAB, MYADM, MYDGF, MYO6, NACC1, NAMPT, NBN, NCOA6, NSF, NUP62, OS9, OTUB1, PACSIN3, PAK2, PARK7, PDCD4, PELP1, PHB, PHF21A, PHIP, PINX1, PNPT1, PPIE, PPP2CA, PQBP1, PRDX5, PRKD1, PTPRJ, QARS, RAB3GAP2, RAB7A, RBFOX2, RBM5, RCOR1, REST, RHOA, RNMT, RPA2, RPRD1B, RPS3, RPS4X, RRAS, SAP130, SCRIB, SDCBP, SERPINB6, SET, SFN, SHC1, SIGIRR, SIN3A, SLC9A1, SMARCB1, SMARCC2, SNIP1, SOD2, SPEN, STRN3, SUPV3L1, SYNJ2BP, TBC1D5, TCP1, TFAM, TOM1L1, TP53BP1, TPD52L1, TRAP1, TRIM25, TSG101, UBAC2, UBE2V1, UBXN1, UCHL5, UFL1, USP47, VCP, VIMP, WFS1, YEATS2, YTHDF2, YWHAE, ZC3HAV1] |
|  | GO 0080090 regulation of primary metabolic process | 33.0E-6 | 620.0E-6 | | 43.0E-6 | 130.0E-6 | [3, 4] | | 5.63 | 152.00 | | [ABCE1, ABHD14B, ACTN4, ADAR, AHCYL1, ANKLE2, ARL6IP5, ATP2B4, BAG6, BAX, BCAP31, BCCIP, BRD4, C14orf166, CCT2, CCT4, CCT5, CCT6A, CCT7, CCT8, CD276, CDH1, CDK11B, CDK5RAP3, CENPE, CHMP1A, CIB1, CNOT1, CTNNB1, CTSH, DDRGK1, DLG1, DNAJA3, DNAJB2, DNAJC3, DNMT1, EEF2, EGFR, EIF2A, EIF3B, EIF4G2, EIF5, EIF5B, EIF6, ENO1, ERBB2, ERP29, ETF1, ETFA, ETFDH, EZR, FAF1, FLOT2, FN1, FXR1, GARS, GNL3, GNL3L, HSP90AA1, HSPA1A, HTRA2, ILK, IST1, JUP, KMT2D, LAMTOR1, LIG3, LONP2, MAP2K2, ME2, MLYCD, MMAB, MYADM, MYDGF, MYO6, NACC1, NAMPT, NBN, NCOA6, NSF, NUP62, OS9, OTUB1, PACSIN3, PAK2, PARK7, PDCD4, PELP1, PHB, PHF21A, PHIP, PINX1, PNPLA2, PNPT1, PPIE, PPP2CA, PQBP1, PRDX5, PRKD1, PTPRJ, QARS, RAB3GAP2, RAB7A, RBFOX2, RBM5, RCOR1, REST, RHOA, RNMT, RPA2, RPRD1B, RPS3, RPS4X, RRAS, SAP130, SDCBP, SERPINB6, SET, SFN, SHC1, SIGIRR, SIN3A, SLC27A1, SLC9A1, SMARCB1, SMARCC2, SNIP1, SOD2, SPEN, STRN3, SUPV3L1, SYNJ2BP, TCP1, TFAM, TOM1L1, TP53BP1, TPD52L1, TRAP1, TRIM25, TSG101, UBAC2, UBE2V1, UBXN1, UCHL5, UFL1, USP47, VCP, WFS1, YEATS2, YTHDF2, YWHAE, ZC3HAV1] |
|  | GO 0009891 positive regulation of biosynthetic process | 250.0E-6 | 2.6E-3 | | 43.0E-6 | 130.0E-6 | [3, 4, 5] | | 4.64 | 48.00 | | [ABHD14B, ACSL3, BRD4, C14orf166, CCT2, CCT4, CCT5, CCT6A, CCT7, CCT8, CD276, CDH1, CTNNB1, EEF2, EGFR, ERBB2, GARS, GNL3, ILK, KMT2D, MAP2K2, MYDGF, MYO6, NAMPT, NCOA6, NUP62, PARK7, PELP1, PHB, PHIP, PRDX5, PRKD1, QARS, RAB3GAP2, REST, RNMT, RPRD1B, RPS4X, SIN3A, SLC9A1, SMARCB1, SMARCC2, STRN3, TCP1, TFAM, TP53BP1, VCP, YTHDF2] |
|  | GO 0010604 positive regulation of macromolecule metabolic process | 140.0E-6 | 1.6E-3 | | 43.0E-6 | 130.0E-6 | [3, 4, 5] | | 5.21 | 88.00 | | [ABHD14B, AGR2, ANKLE2, ARL6IP5, ATP2B4, BAG6, BAX, BCAP31, BRD4, C14orf166, CCT2, CCT4, CCT5, CCT6A, CCT7, CCT8, CD276, CDH1, CDK5RAP3, CENPE, CNOT1, CTNNB1, CTSH, DDRGK1, DNAJA3, DNAJB2, DNMT1, EEF2, EGFR, ERBB2, ERP29, ETFA, ETFDH, EZR, FN1, GARS, GNL3, HSPA1A, HTRA2, ILK, IST1, KMT2D, MAP2K2, MYDGF, MYO6, NAMPT, NBN, NCOA6, NSF, NUP62, PACSIN3, PAK2, PARK7, PELP1, PHB, PHIP, PNPT1, PPP2CA, PRDX5, PRKD1, QARS, RAB3GAP2, RAB7A, REST, RHOA, RNMT, RPRD1B, RPS3, RPS4X, SCRIB, SDCBP, SHC1, SIN3A, SLC9A1, SMARCB1, SMARCC2, STRN3, SUPV3L1, TBC1D5, TCP1, TFAM, TOM1L1, TP53BP1, TPD52L1, VCP, WFS1, YTHDF2, ZC3HAV1] |
|  | GO 0010605 negative regulation of macromolecule metabolic process | 10.0E-3 | 45.0E-3 | | 43.0E-6 | 130.0E-6 | [3, 4, 5] | | 5.52 | 65.00 | | [ABCE1, ADAR, ANXA7, ATXN2, CDK5RAP3, CHMP1A, CNOT1, CTNNB1, DLG1, DNAJA3, DNAJB2, DNAJC3, DNMT1, EGFR, EIF6, ENO1, ERP29, ETF1, EZR, FLOT2, FXR1, GNL3L, HSPA1A, LIG3, MAP2K2, MMAB, MYADM, NACC1, NBN, OS9, OTUB1, PAK2, PARK7, PDCD4, PHB, PHF21A, PINX1, PRDX5, PTPRJ, RBFOX2, RCOR1, REST, RPS3, SAP130, SDCBP, SERPINB6, SET, SFN, SIN3A, SMARCB1, SMARCC2, SNIP1, SPEN, STRN3, SYNJ2BP, TP53BP1, UBAC2, UBE2V1, UBXN1, UFL1, USP47, VIMP, WFS1, YEATS2, YWHAE] |
|  | GO 0018130 heterocycle biosynthetic process | 150.0E-6 | 1.7E-3 | | 43.0E-6 | 130.0E-6 | [4] | | 5.21 | 89.00 | | [ABCB6, ABHD14B, ACAT1, ACTN4, AK1, ALDOA, ATP2B4, ATP5B, ATP5O, BRD4, C14orf166, CAD, CCT2, CCT4, CCT5, CCT6A, CCT7, CCT8, CDH1, CDK5RAP3, CHMP1A, CIB1, CNOT1, COASY, CTNNB1, DDRGK1, DLG1, DNAJA3, DNMT1, EEF1D, EGFR, ENO1, ERBB2, ETF1, EZR, GARS, GNL3, GNL3L, GTF3C2, HSPA1A, ILK, JUP, KMT2D, MAP2K2, MYO6, NACC1, NAMPT, NCOA6, NUP62, PARK7, PDCD4, PELP1, PHB, PHF21A, PHIP, PINX1, POLR2E, POLR2G, PPIE, PRDX5, PRKD1, PRMT5, QTRT1, RBFOX2, RCOR1, REST, RNMT, RPRD1B, SAP130, SET, SIGIRR, SIN3A, SLC9A1, SMARCB1, SMARCC2, SOD2, SPEN, STRN3, TCP1, TFAM, TOM1L1, TP53BP1, TRIM25, UBE2V1, UFL1, USP47, VCP, WFS1, YEATS2] |
|  | GO 0019438 aromatic compound biosynthetic process | 80.0E-6 | 1.0E-3 | | 43.0E-6 | 130.0E-6 | [4] | | 5.15 | 88.00 | | [ABCB6, ABHD14B, ACAT1, ACTN4, AK1, ALDOA, ATP2B4, ATP5B, ATP5O, BRD4, C14orf166, CCT2, CCT4, CCT5, CCT6A, CCT7, CCT8, CDH1, CDK5RAP3, CHMP1A, CIB1, CNOT1, COASY, CTNNB1, DDRGK1, DLG1, DNAJA3, DNMT1, EEF1D, EGFR, ENO1, ERBB2, ETF1, EZR, GARS, GNL3, GNL3L, GTF3C2, HSPA1A, ILK, JUP, KMT2D, MAP2K2, MYO6, NACC1, NAMPT, NCOA6, NUP62, PARK7, PDCD4, PELP1, PHB, PHF21A, PHIP, PINX1, POLR2E, POLR2G, PPIE, PRDX5, PRKD1, PRMT5, QTRT1, RBFOX2, RCOR1, REST, RNMT, RPRD1B, SAP130, SET, SIGIRR, SIN3A, SLC9A1, SMARCB1, SMARCC2, SOD2, SPEN, STRN3, TCP1, TFAM, TOM1L1, TP53BP1, TRIM25, UBE2V1, UFL1, USP47, VCP, WFS1, YEATS2] |
|  | GO 0031324 negative regulation of cellular metabolic process | 8.3E-3 | 38.0E-3 | | 43.0E-6 | 130.0E-6 | [3, 4, 5] | | 5.49 | 67.00 | | [ABCE1, ADAR, ANKLE2, ARFGEF3, ATP2B4, ATXN2, CDK5RAP3, CHMP1A, CNOT1, CTNNB1, DLG1, DNAJA3, DNAJB2, DNAJC3, DNMT1, EGFR, EIF6, ENO1, ETF1, EZR, FXR1, GNL3L, HSPA1A, LIG3, MAP2K2, MMAB, MYADM, MYO1D, NACC1, NBN, OTUB1, PAK2, PARK7, PDCD4, PHB, PHF21A, PINX1, PRDX5, PTPRJ, RBFOX2, RCOR1, REST, RPS3, RRP1B, SAP130, SDCBP, SERPINB6, SET, SFN, SIN3A, SLC27A1, SMARCB1, SMARCC2, SNIP1, SPEN, STRN3, SYNJ2BP, TP53BP1, TRAP1, UBE2V1, UBXN1, UFL1, USP47, WFS1, WNK1, YEATS2, YWHAE] |
|  | GO 0031325 positive regulation of cellular metabolic process | 72.0E-6 | 1.0E-3 | | 43.0E-6 | 130.0E-6 | [3, 4, 5] | | 5.11 | 86.00 | | [ABHD14B, ANKLE2, ARL6IP5, ATP2B4, BAX, BCAP31, BRD4, C14orf166, CCT2, CCT4, CCT5, CCT6A, CCT7, CCT8, CD276, CDH1, CDK5RAP3, CENPE, CNOT1, CTNNB1, CTSH, DDRGK1, DNAJA3, DNAJB2, DNMT1, EEF2, EGFR, ERBB2, ERP29, ETFA, ETFDH, EZR, GARS, GNL3, GRB2, HSPA1A, HTRA2, ILK, IST1, KMT2D, MAP2K2, MLYCD, MYDGF, MYO6, NAMPT, NBN, NCOA6, NSF, NUP62, PACSIN3, PAK2, PARK7, PELP1, PHB, PHIP, PNPLA2, PNPT1, PPP2CA, PRKD1, QARS, RAB3GAP2, REST, RHOA, RNMT, RPRD1B, RPS3, RPS4X, SCRIB, SDCBP, SHC1, SIN3A, SLC9A1, SMARCB1, SMARCC2, STRN3, SUPV3L1, TBC1D5, TCP1, TFAM, TOM1L1, TP53BP1, TPD52L1, VCP, WFS1, YTHDF2, ZC3HAV1] |
|  | GO 0051173 positive regulation of nitrogen compound metabolic process | 2.2E-3 | 13.0E-3 | | 43.0E-6 | 130.0E-6 | [3, 4, 5] | | 5.03 | 53.00 | | [ABHD14B, ACSL3, BAX, BRD4, C14orf166, CCT2, CCT4, CCT5, CCT6A, CCT7, CCT8, CDH1, CNOT1, CTNNB1, DNMT1, EEF2, EGFR, ERBB2, GARS, GNL3, HSPA1A, ILK, KMT2D, MAP2K2, MYO6, NAMPT, NBN, NCOA6, NUP62, PARK7, PELP1, PHB, PHIP, PNPT1, PRKD1, QARS, REST, RNMT, RPRD1B, RPS3, RPS4X, SIN3A, SLC9A1, SMARCB1, SMARCC2, STRN3, SUPV3L1, TCP1, TFAM, TP53BP1, VCP, YTHDF2, ZC3HAV1] |
|  | GO 1901362 organic cyclic compound biosynthetic process | 480.0E-6 | 4.5E-3 | | 43.0E-6 | 130.0E-6 | [4] | | 5.42 | 95.00 | | [ABCB6, ABHD14B, ACAT1, ACTN4, AK1, ALDOA, ATP2B4, ATP5B, ATP5O, BRD4, C14orf166, CAD, CCT2, CCT4, CCT5, CCT6A, CCT7, CCT8, CDH1, CDK5RAP3, CHMP1A, CIB1, CNOT1, COASY, CTNNB1, CYP51A1, DDRGK1, DHCR7, DLG1, DNAJA3, DNMT1, EEF1D, EGFR, ENO1, ERBB2, ETF1, EZR, GARS, GLB1, GNL3, GNL3L, GTF3C2, HSPA1A, ILK, JUP, KMT2D, LSS, MAP2K2, MYO6, NACC1, NAMPT, NCOA6, NUP62, PARK7, PDCD4, PELP1, PHB, PHF21A, PHIP, PINX1, PMVK, POLR2E, POLR2G, PPIE, PRDX5, PRKD1, PRMT5, QTRT1, RBFOX2, RCOR1, REST, RNMT, RPRD1B, SAP130, SET, SIGIRR, SIN3A, SLC27A2, SLC9A1, SMARCB1, SMARCC2, SOD2, SPEN, STRN3, TCP1, TFAM, TOM1L1, TP53BP1, TRIM25, UBE2V1, UFL1, USP47, VCP, WFS1, YEATS2] |
|  | GO 0010468 regulation of gene expression | 9.2E-6 | 220.0E-6 | | 43.0E-6 | 130.0E-6 | [4, 5] | | 4.98 | 90.00 | | [ABHD14B, ADAR, AGR2, AHCYL1, ANXA7, ATP2B4, BRD4, C14orf166, CDH1, CDK11B, CDK5RAP3, CHMP1A, CIB1, CNOT1, CTNNB1, CTSH, DDRGK1, DLG1, DNAJA3, DNAJC3, DNMT1, EEF2, EGFR, EIF2A, EIF3B, EIF4G2, EIF5, EIF5B, EIF6, ENO1, ERBB2, ERP29, ETF1, EZR, FLOT2, FN1, FXR1, GARS, GNL3, HSPA1A, ILK, JUP, KMT2D, MAP2K2, MYADM, MYO6, NACC1, NAMPT, NCOA6, NUP62, PARK7, PDCD4, PELP1, PHB, PHF21A, PHIP, PPIE, PQBP1, PRDX5, PRKD1, QARS, RBFOX2, RBM5, RCOR1, REST, RNMT, RPRD1B, RPS3, RPS4X, SAP130, SET, SIGIRR, SIN3A, SLC9A1, SMARCB1, SMARCC2, SNIP1, SOD2, SPEN, STRN3, TFAM, TP53BP1, TRAP1, TRIM25, UBE2V1, UFL1, USP47, WFS1, YEATS2, YTHDF2] |
|  | GO 0010556 regulation of macromolecule biosynthetic process | 1.9E-3 | 12.0E-3 | | 43.0E-6 | 130.0E-6 | [4, 5] | | 5.60 | 96.00 | | [ABHD14B, ACTN4, ADAR, ATP2B4, BRD4, C14orf166, CCT2, CCT4, CCT5, CCT6A, CCT7, CCT8, CD276, CDH1, CDK5RAP3, CHMP1A, CIB1, CNOT1, CTNNB1, DDRGK1, DLG1, DNAJA3, DNAJC3, DNMT1, EEF2, EGFR, EIF2A, EIF3B, EIF4G2, EIF5, EIF5B, EIF6, ENO1, ERBB2, ETF1, EZR, FXR1, GARS, GNL3, GNL3L, HSPA1A, ILK, JUP, KMT2D, LIG3, MAP2K2, MMAB, MYDGF, MYO6, NACC1, NAMPT, NCOA6, NUP62, PARK7, PDCD4, PELP1, PHB, PHF21A, PHIP, PINX1, PPIE, PRDX5, PRKD1, QARS, RAB3GAP2, RBFOX2, RCOR1, REST, RNMT, RPRD1B, RPS3, RPS4X, SAP130, SET, SIGIRR, SIN3A, SLC9A1, SMARCB1, SMARCC2, SNIP1, SOD2, SPEN, STRN3, TCP1, TFAM, TOM1L1, TP53BP1, TRAP1, TRIM25, UBE2V1, UFL1, USP47, VIMP, WFS1, YEATS2, YTHDF2] |
|  | GO 0019219 regulation of nucleobase-containing compound metabolic process | 41.0E-6 | 700.0E-6 | | 43.0E-6 | 130.0E-6 | [4, 5] | | 5.12 | 91.00 | | [ABCE1, ABHD14B, ACTN4, AHCYL1, ATP2B4, BAX, BRD4, C14orf166, CCT2, CCT4, CCT5, CCT6A, CCT7, CCT8, CDH1, CDK11B, CDK5RAP3, CHMP1A, CIB1, CNOT1, CTNNB1, DDRGK1, DLG1, DNAJA3, DNMT1, EGFR, ENO1, ERBB2, ETF1, EZR, GARS, GNL3, GNL3L, HSPA1A, ILK, JUP, KMT2D, LIG3, MAP2K2, ME2, MMAB, MYO6, NACC1, NAMPT, NBN, NCOA6, NUP62, OTUB1, PARK7, PDCD4, PELP1, PHB, PHF21A, PHIP, PINX1, PNPT1, PPIE, PQBP1, PRDX5, PRKD1, RBFOX2, RBM5, RCOR1, REST, RNMT, RPA2, RPRD1B, RPS3, SAP130, SET, SIGIRR, SIN3A, SLC9A1, SMARCB1, SMARCC2, SOD2, SPEN, STRN3, SUPV3L1, TCP1, TFAM, TOM1L1, TP53BP1, TRIM25, UBE2V1, UFL1, USP47, VCP, WFS1, YEATS2, ZC3HAV1] |
|  | GO 0031326 regulation of cellular biosynthetic process | 430.0E-6 | 4.2E-3 | | 43.0E-6 | 130.0E-6 | [4, 5] | | 5.42 | 97.00 | | [ABHD14B, ACTN4, ADAR, ATP2B4, BRD4, C14orf166, CCT2, CCT4, CCT5, CCT6A, CCT7, CCT8, CD276, CDH1, CDK5RAP3, CHMP1A, CIB1, CNOT1, CTNNB1, DDRGK1, DLG1, DNAJA3, DNAJC3, DNMT1, EEF2, EGFR, EIF2A, EIF3B, EIF4G2, EIF5, EIF5B, EIF6, ENO1, ERBB2, ETF1, EZR, FXR1, GARS, GNL3, GNL3L, HSPA1A, ILK, JUP, KMT2D, LIG3, MAP2K2, MMAB, MYDGF, MYO6, NACC1, NAMPT, NCOA6, NUP62, PARK7, PDCD4, PELP1, PHB, PHF21A, PHIP, PINX1, PPIE, PRDX5, PRKD1, QARS, RAB3GAP2, RBFOX2, RCOR1, REST, RNMT, RPRD1B, RPS3, RPS4X, SAP130, SET, SIGIRR, SIN3A, SLC27A1, SLC9A1, SMARCB1, SMARCC2, SNIP1, SOD2, SPEN, STRN3, TCP1, TFAM, TOM1L1, TP53BP1, TRAP1, TRIM25, UBE2V1, UFL1, USP47, VCP, WFS1, YEATS2, YTHDF2] |
|  | GO 0090304 nucleic acid metabolic process | 3.5E-3 | 18.0E-3 | | 43.0E-6 | 130.0E-6 | [4, 5] | | 5.90 | 127.00 | | [ABCE1, ABHD14B, ACTN4, ADAR, AHCYL1, ATP2B4, BAX, BRD4, C14orf166, C9orf142, CARS, CCT2, CCT4, CCT5, CCT6A, CCT7, CCT8, CDH1, CDK11B, CDK5RAP3, CHMP1A, CIB1, CMTR1, CNOT1, CTNNB1, DDRGK1, DDX1, DLG1, DNAJA3, DNMT1, EEA1, EEF1D, EGFR, ENO1, ERBB2, ETF1, EZR, FARSA, FARSB, GARS, GATB, GEMIN5, GNL3, GNL3L, GTF3C2, HARS2, HSPA1A, HUWE1, ILK, JUP, KARS, KMT2D, LARS2, LIG3, MAP2K2, MARS2, MCM2, METTL14, MMAB, MYO6, NACC1, NAMPT, NBN, NCOA6, NSUN2, NSUN4, NUDT21, NUP62, OTUB1, PARK7, PARN, PDCD4, PELP1, PHB, PHF21A, PHIP, PI4KA, PINX1, PNPT1, POLR2E, POLR2G, PPIE, PQBP1, PRDX5, PRKD1, PRMT5, PRPF19, PURA, QARS, RBFOX2, RBM5, RCOR1, REST, RNMT, RPA2, RPL7, RPRD1B, RPS28, RPS3, RPS6, RTCB, SAP130, SET, SIGIRR, SIN3A, SLC9A1, SMARCB1, SMARCC2, SNIP1, SNRPB, SOD2, SPEN, STRN3, SUPV3L1, TCP1, TFAM, TOM1L1, TP53BP1, TRIM25, TRMU, UBE2V1, UFL1, USP47, VCP, WFS1, YEATS2, ZC3HAV1] |
|  | GO 0010557 positive regulation of macromolecule biosynthetic process | 930.0E-6 | 7.2E-3 | | 43.0E-6 | 130.0E-6 | [4, 5, 6] | | 4.78 | 46.00 | | [ABHD14B, BRD4, C14orf166, CCT2, CCT4, CCT5, CCT6A, CCT7, CCT8, CD276, CDH1, CTNNB1, EEF2, EGFR, ERBB2, GARS, GNL3, ILK, KMT2D, MAP2K2, MYDGF, MYO6, NAMPT, NCOA6, NUP62, PARK7, PELP1, PHB, PHIP, PRDX5, PRKD1, QARS, RAB3GAP2, REST, RNMT, RPRD1B, RPS4X, SIN3A, SLC9A1, SMARCB1, SMARCC2, STRN3, TCP1, TFAM, TP53BP1, YTHDF2] |
|  | GO 0010628 positive regulation of gene expression | 100.0E-6 | 1.2E-3 | | 43.0E-6 | 130.0E-6 | [4, 5, 6] | | 4.39 | 43.00 | | [ABHD14B, AGR2, BRD4, C14orf166, CDH1, CTNNB1, CTSH, DNMT1, EEF2, EGFR, ERBB2, ERP29, EZR, FN1, GARS, GNL3, HSPA1A, ILK, KMT2D, MAP2K2, MYO6, NAMPT, NCOA6, NUP62, PARK7, PELP1, PHB, PHIP, PRKD1, QARS, REST, RNMT, RPRD1B, RPS3, RPS4X, SIN3A, SLC9A1, SMARCB1, SMARCC2, STRN3, TFAM, TP53BP1, YTHDF2] |
|  | GO 0016070 RNA metabolic process | 2.5E-3 | 14.0E-3 | | 43.0E-6 | 130.0E-6 | [4, 5, 6] | | 5.70 | 105.00 | | [ABCE1, ABHD14B, ACTN4, ADAR, AHCYL1, ATP2B4, BRD4, C14orf166, CARS, CDH1, CDK11B, CDK5RAP3, CHMP1A, CIB1, CMTR1, CNOT1, CTNNB1, DDRGK1, DDX1, DLG1, DNAJA3, DNMT1, EEA1, EEF1D, EGFR, ENO1, ERBB2, ETF1, EZR, FARSA, FARSB, GARS, GATB, GEMIN5, GNL3, GTF3C2, HARS2, HSPA1A, ILK, JUP, KARS, KMT2D, LARS2, MAP2K2, MARS2, METTL14, MYO6, NACC1, NAMPT, NCOA6, NSUN2, NSUN4, NUDT21, NUP62, PARK7, PARN, PDCD4, PELP1, PHB, PHF21A, PHIP, PI4KA, PNPT1, POLR2E, POLR2G, PPIE, PQBP1, PRDX5, PRKD1, PRMT5, PRPF19, QARS, RBFOX2, RBM5, RCOR1, REST, RNMT, RPL7, RPRD1B, RPS28, RPS6, RTCB, SAP130, SET, SIGIRR, SIN3A, SLC9A1, SMARCB1, SMARCC2, SNIP1, SNRPB, SOD2, SPEN, STRN3, SUPV3L1, TFAM, TP53BP1, TRIM25, TRMU, UBE2V1, UFL1, USP47, WFS1, YEATS2, ZC3HAV1] |
|  | GO 0031328 positive regulation of cellular biosynthetic process | 130.0E-6 | 1.6E-3 | | 43.0E-6 | 130.0E-6 | [4, 5, 6] | | 4.50 | 46.00 | | [ABHD14B, BRD4, C14orf166, CCT2, CCT4, CCT5, CCT6A, CCT7, CCT8, CD276, CDH1, CTNNB1, EEF2, EGFR, ERBB2, GARS, GNL3, ILK, KMT2D, MAP2K2, MYDGF, MYO6, NAMPT, NCOA6, NUP62, PARK7, PELP1, PHB, PHIP, PRKD1, QARS, RAB3GAP2, REST, RNMT, RPRD1B, RPS4X, SIN3A, SLC9A1, SMARCB1, SMARCC2, STRN3, TCP1, TFAM, TP53BP1, VCP, YTHDF2] |
|  | GO 0045935 positive regulation of nucleobase-containing compound metabolic process | 710.0E-6 | 5.8E-3 | | 43.0E-6 | 130.0E-6 | [4, 5, 6] | | 4.77 | 48.00 | | [ABHD14B, BAX, BRD4, C14orf166, CCT2, CCT4, CCT5, CCT6A, CCT7, CCT8, CDH1, CNOT1, CTNNB1, DNMT1, EGFR, ERBB2, GARS, GNL3, HSPA1A, ILK, KMT2D, MAP2K2, MYO6, NAMPT, NBN, NCOA6, NUP62, PARK7, PELP1, PHB, PHIP, PNPT1, PRKD1, REST, RNMT, RPRD1B, RPS3, SIN3A, SLC9A1, SMARCB1, SMARCC2, STRN3, SUPV3L1, TCP1, TFAM, TP53BP1, VCP, ZC3HAV1] |
|  | GO 0051252 regulation of RNA metabolic process | 4.3E-6 | 130.0E-6 | | 43.0E-6 | 130.0E-6 | [4, 5, 6, 7] | | 4.66 | 72.00 | | [ABCE1, ABHD14B, ACTN4, AHCYL1, ATP2B4, BRD4, C14orf166, CDH1, CDK11B, CDK5RAP3, CHMP1A, CIB1, CNOT1, CTNNB1, DDRGK1, DLG1, DNAJA3, DNMT1, EGFR, ENO1, ERBB2, ETF1, EZR, GARS, GNL3, HSPA1A, ILK, JUP, KMT2D, MAP2K2, MYO6, NACC1, NAMPT, NCOA6, NUP62, PARK7, PDCD4, PELP1, PHB, PHF21A, PHIP, PNPT1, PPIE, PQBP1, PRDX5, PRKD1, RBFOX2, RBM5, RCOR1, REST, RNMT, RPRD1B, SAP130, SET, SIGIRR, SIN3A, SLC9A1, SMARCB1, SMARCC2, SOD2, SPEN, STRN3, SUPV3L1, TFAM, TP53BP1, TRIM25, UBE2V1, UFL1, USP47, WFS1, YEATS2, ZC3HAV1] |
|  | GO 2000112 regulation of cellular macromolecule biosynthetic process | 2.0E-3 | 12.0E-3 | | 43.0E-6 | 130.0E-6 | [5, 6] | | 5.55 | 92.00 | | [ABHD14B, ADAR, ATP2B4, BRD4, C14orf166, CCT2, CCT4, CCT5, CCT6A, CCT7, CCT8, CDH1, CDK5RAP3, CHMP1A, CIB1, CNOT1, CTNNB1, DDRGK1, DLG1, DNAJA3, DNAJC3, DNMT1, EEF2, EGFR, EIF2A, EIF3B, EIF4G2, EIF5, EIF5B, EIF6, ENO1, ERBB2, ETF1, EZR, FXR1, GARS, GNL3, GNL3L, HSPA1A, ILK, JUP, KMT2D, LIG3, MAP2K2, MMAB, MYO6, NACC1, NAMPT, NCOA6, NUP62, PARK7, PDCD4, PELP1, PHB, PHF21A, PHIP, PINX1, PPIE, PRDX5, PRKD1, QARS, RAB3GAP2, RBFOX2, RCOR1, REST, RNMT, RPRD1B, RPS3, RPS4X, SAP130, SET, SIGIRR, SIN3A, SLC9A1, SMARCB1, SMARCC2, SNIP1, SOD2, SPEN, STRN3, TCP1, TFAM, TOM1L1, TP53BP1, TRAP1, TRIM25, UBE2V1, UFL1, USP47, WFS1, YEATS2, YTHDF2] |
|  | GO 0045937 positive regulation of phosphate metabolic process | 3.4E-3 | 18.0E-3 | | 43.0E-6 | 130.0E-6 | [5, 6, 7] | | 4.36 | 25.00 | | [ANKLE2, ARL6IP5, ATP2B4, CENPE, DDRGK1, EGFR, ERBB2, ERP29, ETFA, ETFDH, ILK, MAP2K2, NBN, PAK2, PARK7, PHB, PPP2CA, PRKD1, RHOA, RPS3, SDCBP, SHC1, TOM1L1, TPD52L1, VCP] |
|  | GO 0051253 negative regulation of RNA metabolic process | 6.3E-3 | 31.0E-3 | | 43.0E-6 | 130.0E-6 | [4, 5, 6, 7, 8] | | 4.64 | 29.00 | | [ABCE1, CHMP1A, CNOT1, CTNNB1, DLG1, DNAJA3, DNMT1, ENO1, ETF1, EZR, NACC1, PDCD4, PHB, PHF21A, PRDX5, RBFOX2, RCOR1, REST, SAP130, SET, SIN3A, SMARCC2, SPEN, STRN3, TP53BP1, UBE2V1, USP47, WFS1, YEATS2] |
|  | GO 0051254 positive regulation of RNA metabolic process | 120.0E-6 | 1.4E-3 | | 43.0E-6 | 130.0E-6 | [4, 5, 6, 7, 8] | | 4.22 | 36.00 | | [ABHD14B, BRD4, C14orf166, CDH1, CNOT1, CTNNB1, EGFR, ERBB2, GARS, GNL3, HSPA1A, ILK, KMT2D, MAP2K2, MYO6, NAMPT, NCOA6, NUP62, PARK7, PELP1, PHB, PHIP, PNPT1, PRKD1, REST, RNMT, RPRD1B, SIN3A, SLC9A1, SMARCB1, SMARCC2, STRN3, SUPV3L1, TFAM, TP53BP1, ZC3HAV1] |
|  | GO 0097659 nucleic acid-templated transcription | 6.8E-6 | 180.0E-6 | | 43.0E-6 | 130.0E-6 | [6, 7, 8] | | 4.66 | 69.00 | | [ABHD14B, ACTN4, ATP2B4, BRD4, C14orf166, CDH1, CDK5RAP3, CHMP1A, CIB1, CNOT1, CTNNB1, DDRGK1, DLG1, DNAJA3, DNMT1, EEF1D, EGFR, ENO1, ERBB2, ETF1, EZR, GARS, GNL3, GTF3C2, HSPA1A, ILK, JUP, KMT2D, MAP2K2, MYO6, NACC1, NAMPT, NCOA6, NUP62, PARK7, PDCD4, PELP1, PHB, PHF21A, PHIP, POLR2E, POLR2G, PPIE, PRDX5, PRKD1, PRMT5, RBFOX2, RCOR1, REST, RNMT, RPRD1B, SAP130, SET, SIGIRR, SIN3A, SLC9A1, SMARCB1, SMARCC2, SOD2, SPEN, STRN3, TFAM, TP53BP1, TRIM25, UBE2V1, UFL1, USP47, WFS1, YEATS2] |
|  | GO 1903506 regulation of nucleic acid-templated transcription | 2.8E-6 | 100.0E-6 | | 43.0E-6 | 130.0E-6 | [6, 7, 8, 9] | | 4.48 | 64.00 | | [ABHD14B, ACTN4, ATP2B4, BRD4, C14orf166, CDH1, CDK5RAP3, CHMP1A, CIB1, CNOT1, CTNNB1, DDRGK1, DLG1, DNAJA3, DNMT1, EGFR, ENO1, ERBB2, ETF1, EZR, GARS, GNL3, HSPA1A, ILK, JUP, KMT2D, MAP2K2, MYO6, NACC1, NAMPT, NCOA6, NUP62, PARK7, PDCD4, PELP1, PHB, PHF21A, PHIP, PPIE, PRDX5, PRKD1, RBFOX2, RCOR1, REST, RNMT, RPRD1B, SAP130, SET, SIGIRR, SIN3A, SLC9A1, SMARCB1, SMARCC2, SOD2, SPEN, STRN3, TFAM, TP53BP1, TRIM25, UBE2V1, UFL1, USP47, WFS1, YEATS2] |
|  | GO 0006366 transcription from RNA polymerase II promoter | 32.0E-6 | 620.0E-6 | | 43.0E-6 | 130.0E-6 | [6, 7, 8, 9, 10] | | 4.14 | 39.00 | | [ABHD14B, ATP2B4, BRD4, C14orf166, CNOT1, CTNNB1, DLG1, DNAJA3, EGFR, ETF1, EZR, GARS, GNL3, KMT2D, MYO6, NAMPT, NCOA6, PARK7, PELP1, PHB, PHF21A, PHIP, POLR2E, POLR2G, PRKD1, REST, RNMT, RPRD1B, SAP130, SIN3A, SLC9A1, SMARCB1, SOD2, SPEN, STRN3, TFAM, TP53BP1, WFS1, YEATS2] |
|  | GO 0006357 regulation of transcription from RNA polymerase II promoter | 52.0E-6 | 790.0E-6 | | 43.0E-6 | 130.0E-6 | [6, 7, 8, 9, 10, 11] | | 4.13 | 37.00 | | [ABHD14B, ATP2B4, BRD4, C14orf166, CNOT1, CTNNB1, DLG1, DNAJA3, EGFR, ETF1, EZR, GARS, GNL3, KMT2D, MYO6, NAMPT, NCOA6, PARK7, PELP1, PHB, PHF21A, PHIP, PRKD1, REST, RNMT, RPRD1B, SAP130, SIN3A, SLC9A1, SMARCB1, SOD2, SPEN, STRN3, TFAM, TP53BP1, WFS1, YEATS2] |
|  | GO 0031579 membrane raft organization | 11.0E-3 | 47.0E-3 | | 11.0E-3 | 11.0E-3 | [4, 5] | | 30.77 | 4.00 | | [FLOT2, MYADM, PACSIN2, PPT1] |
|  | GO 0032508 DNA duplex unwinding | 8.6E-3 | 39.0E-3 | | 8.6E-3 | 9.3E-3 | [7] | | 33.33 | 4.00 | | [DDX1, NBN, PURA, SUPV3L1] |
|  | GO 0006996 organelle organization | 9.7E-9 | 2.5E-6 | | 9.7E-9 | 99.0E-9 | [3] | | 10.97 | 163.00 | | [ABCC4, ABCD3, ACAD9, ACBD5, ACTN1, ACTR3, AIP, ANKLE2, ARFIP2, ARHGDIA, ATXN2, ATXN2L, BAG6, BAIAP2, BAX, BNIP1, BNIP3L, CAPZB, CCT2, CCT4, CCT5, CCT6A, CCT7, CCT8, CDC27, CENPE, CHCHD4, CHMP1A, CIB1, CKAP5, CNOT1, COG2, COG3, COG4, CTNNB1, CXADR, CYB5R1, CYFIP1, DDX1, DDX6, DHX30, DLG1, DNM1L, DYNC1H1, EEA1, EIF2A, EIF6, EPT1, EZR, FASTKD2, FITM2, FOXRED1, GATB, GBF1, GCC2, GDAP1, GFM1, GFM2, GNL3, GNL3L, GOLGA5, HOOK1, HSP90AA1, HSPA1A, HSPA4, HTRA2, HUWE1, IMMT, KIF2C, LEMD2, LIG3, LMAN1, LONP2, MAP1S, MAP4, MAPT, MFN1, MFN2, MICAL3, MRRF, MTPN, MYADM, MYH10, MYH14, MYH9, MYO18A, MYO5A, NBN, NDUFAF4, NDUFS1, NDUFS8, NPEPPS, NUP133, NUP153, OPA1, PAFAH1B1, PDCD6IP, PDZD8, PEX13, PFN1, PHB, PHIP, PINX1, PMPCA, PNPT1, PPFIA1, PPIA, PPIF, PPT1, PQBP1, PRKD1, PRMT5, PSMB7, PTCD3, PURA, RAB10, RAB2A, RAB3GAP2, RAB7A, RALA, RHOA, RHOT1, RPA2, RPS3, RTN4, RUVBL1, SAMM50, SDCBP, SEC61A1, SEPT9, SFN, SLK, SMC2, SMIM20, SNAP29, SNIP1, STX12, STX18, SUPV3L1, TBC1D30, TBC1D5, TCP1, TFAM, TIMM21, TMED5, TMEM165, TOM1L1, TOM1L2, TOMM34, TOR1A, TP53BP1, TSG101, TTC19, U2AF1, VAMP7, VCP, VPS33A, VTA1, VTI1B, WASF2, WDR1, WDR46, ZW10] |
|  | GO 0033043 regulation of organelle organization | 11.0E-3 | 47.0E-3 | | 9.7E-9 | 99.0E-9 | [4, 5] | | 9.83 | 64.00 | | [ACTR3, ARHGDIA, ATXN2L, BAIAP2, BAX, CCT2, CCT4, CCT5, CCT6A, CCT7, CCT8, CDC27, CENPE, CHMP1A, CIB1, CNOT1, CTNNB1, CYB5R1, CYFIP1, DNM1L, EPT1, EZR, GNL3, GNL3L, HSPA1A, HTRA2, HUWE1, LIG3, LMAN1, MAP4, MAPT, MTPN, MYADM, MYO5A, NBN, NPEPPS, OPA1, PDCD6IP, PFN1, PINX1, PPFIA1, PPIF, PSMB7, RAB3GAP2, RAB7A, RHOA, RPS3, RUVBL1, SDCBP, SEPT9, SNIP1, STX18, TBC1D30, TBC1D5, TCP1, TOM1L1, TOM1L2, TP53BP1, TSG101, U2AF1, WASF2, WDR1, WDR46, ZW10] |
|  | GO 0033108 mitochondrial respiratory chain complex assembly | 1.3E-3 | 9.7E-3 | | 1.3E-3 | 2.1E-3 | [5, 6, 7] | | 25.81 | 8.00 | | [ACAD9, FOXRED1, NDUFAF4, NDUFS8, SAMM50, SMIM20, TIMM21, TTC19] |
|  | GO 0032981 mitochondrial respiratory chain complex I assembly | 9.9E-3 | 43.0E-3 | | 1.3E-3 | 2.1E-3 | [5, 6, 7, 8] | | 26.32 | 5.00 | | [ACAD9, FOXRED1, NDUFAF4, NDUFS8, TIMM21] |
|  | GO 0042398 cellular modified amino acid biosynthetic process | 4.1E-3 | 21.0E-3 | | 4.1E-3 | 5.2E-3 | [5] | | 40.00 | 4.00 | | [HAGH, MGST2, PARK7, SLC27A1] |
|  | GO 0043171 peptide catabolic process | 640.0E-6 | 5.3E-3 | | 640.0E-6 | 1.2E-3 | [4, 5, 6] | | 45.45 | 5.00 | | [CPQ, CTSH, IDE, LTA4H, TPP1] |
|  | GO 0043312 neutrophil degranulation | 6.5E-3 | 32.0E-3 | | 6.5E-3 | 7.6E-3 | [4, 6, 7, 8, 9, 10] | | 50.00 | 3.00 | | [STXBP2, STXBP3, VAMP7] |
|  | GO 0044248 cellular catabolic process | 89.0E-6 | 1.2E-3 | | 36.0E-9 | 300.0E-9 | [3] | | 11.30 | 75.00 | | [ABCD3, ABHD12, ABHD16A, ABHD6, ACAA1, ACADM, ACAT1, ACSF3, ALDH5A1, ALDH6A1, ATP2B4, BAX, BCAP31, BCKDHA, BLVRB, BNIP3L, CDK5RAP3, CLPX, CNOT1, CPQ, CROT, CTSH, DDRGK1, DNAJB2, ECI2, ETFA, ETFDH, EZR, FAAH, GCDH, GOT1, HOGA1, HTRA2, IDE, KIAA0368, LONP2, LTA4H, MAN1B1, MLYCD, NAGA, NEDD4L, OS9, PACSIN3, PARK7, PARN, PCNP, PCYOX1, PDCD6IP, PNPLA2, PNPT1, PON2, PRDX5, PRPF19, PSMC2, PTPN23, RAB7A, RNF213, SDCBP, SLC25A17, SLC27A2, SORD, STBD1, SUPV3L1, TMUB1, TPP1, TRIM25, TSG101, UBA6, UBXN1, UCHL5, UFL1, VCP, VIMP, WFS1, ZC3HAV1] |
|  | GO 0044281 small molecule metabolic process | 2.8E-6 | 98.0E-6 | | 36.0E-9 | 300.0E-9 | [3] | | 11.89 | 86.00 | | [ABCD3, ACAA1, ACADM, ACAT1, ACLY, ACO2, ACOT1, ACSF3, ADCK3, AK1, AK3, ALDH3A2, ALDH5A1, ALDH6A1, ALDOA, AMACR, ATP2B4, ATP5B, ATP5O, BCKDHA, CAD, CARS, CBR1, COA6, COASY, COQ3, CROT, DDAH1, DDAH2, DECR2, DHCR7, DHRS4, DPM1, ECI2, ELOVL5, ETFA, ETFDH, FAAH, FARSA, FARSB, GARS, GATB, GCDH, GLB1, GNAI3, GOT1, GUK1, HARS2, HOGA1, HSPA1A, IDH1, IMPA1, KARS, LARS2, LONP2, LSS, LTA4H, MACROD1, MARC1, MARS2, MCAT, ME2, MGST2, MLYCD, MTHFD1, MUT, NDUFS1, OLA1, OXCT1, PARK7, PDHA1, PDHB, PECR, PFKP, PGD, PMVK, PRDX5, QARS, QTRT1, REST, SCD, SLC25A17, SLC27A2, SORD, TECR, VCP] |
|  | GO 0044712 single-organism catabolic process | 2.7E-6 | 100.0E-6 | | 36.0E-9 | 300.0E-9 | [3] | | 14.98 | 46.00 | | [ABCD3, ABHD12, ABHD16A, ABHD6, ACAA1, ACADM, ACAT1, ACSF3, ALDH5A1, ALDH6A1, ALDOA, ATP2B4, BAG6, BCAP31, BCKDHA, BLVRB, CROT, DNAJB2, ECI2, ETFA, ETFDH, FAAH, GCDH, GOT1, HOGA1, IMPA1, KIAA0368, LONP2, MAN1B1, NAGA, NPLOC4, OS9, PFKP, PNPLA2, PPT1, SLC25A17, SLC27A2, SORD, STBD1, TMUB1, TRIM25, UBAC2, UBXN1, VCP, VIMP, WFS1] |
|  | GO 0055114 oxidation-reduction process | 46.0E-6 | 740.0E-6 | | 36.0E-9 | 300.0E-9 | [3] | | 15.87 | 30.00 | | [ABCD3, ACAA1, ACADM, ACO2, ALDH3A2, BLVRA, CBR1, COA6, CROT, DHRS4, ETFA, ETFDH, GCDH, GRHPR, LONP2, MARC1, MLYCD, NDUFS1, PARK7, PDHA1, PDHB, PECR, PGD, PRDX5, SLC25A17, SLC27A2, SQRDL, STBD1, TRAP1, VCP] |
|  | GO 1901575 organic substance catabolic process | 32.0E-6 | 620.0E-6 | | 36.0E-9 | 300.0E-9 | [3] | | 11.29 | 85.00 | | [ABCD3, ABHD12, ABHD16A, ABHD6, ACAA1, ACADM, ACAT1, ACSF3, ALDH5A1, ALDH6A1, ALDOA, ATP2B4, BAG6, BAX, BCAP31, BCKDHA, BLVRB, BNIP3L, CDK5RAP3, CLPX, CNOT1, CPQ, CROT, CTSH, DDRGK1, DNAJB2, ECI2, EGFR, ETFA, ETFDH, EZR, FAAH, FAF1, GCDH, GOT1, HOGA1, HTRA2, IDE, IMPA1, KIAA0368, LONP2, LTA4H, MAN1B1, MLYCD, NAGA, NEDD4L, NPLOC4, NSF, OS9, PACSIN3, PARK7, PARN, PCNP, PCYOX1, PDCD6IP, PFKP, PHB, PNPLA2, PNPT1, PPT1, PRPF19, PSMC2, PTPN23, RAB7A, RNF213, SDCBP, SLC25A17, SLC27A2, SORD, STBD1, SUPV3L1, TMUB1, TPP1, TRIM24, TRIM25, TSG101, UBA6, UBAC2, UBXN1, UCHL5, UFL1, VCP, VIMP, WFS1, ZC3HAV1] |
|  | GO 0006082 organic acid metabolic process | 420.0E-9 | 30.0E-6 | | 36.0E-9 | 300.0E-9 | [3, 4] | | 14.99 | 52.00 | | [ABCD3, ACAA1, ACADM, ACAT1, ACLY, ACO2, ACOT1, ACSF3, ALDH5A1, ALDH6A1, ALDOA, AMACR, ATP2B4, BCKDHA, CARS, CROT, DDAH1, DDAH2, DECR2, ECI2, ELOVL5, ETFA, ETFDH, FAAH, FARSA, FARSB, GATB, GCDH, GOT1, HARS2, HOGA1, IDH1, KARS, LARS2, LONP2, LTA4H, MARC1, MARS2, MCAT, MGST2, MLYCD, MTHFD1, MUT, PARK7, PDHA1, PDHB, PFKP, QARS, SCD, SLC25A17, SLC27A2, TECR] |
|  | GO 0016042 lipid catabolic process | 410.0E-6 | 4.0E-3 | | 36.0E-9 | 300.0E-9 | [4] | | 17.48 | 18.00 | | [ABCD3, ABHD12, ABHD16A, ABHD6, ACAA1, ACADM, CROT, ECI2, ETFA, ETFDH, FAAH, GCDH, LONP2, NAGA, PNPLA2, PPT1, SLC25A17, SLC27A2] |
|  | GO 0044282 small molecule catabolic process | 5.9E-6 | 170.0E-6 | | 36.0E-9 | 300.0E-9 | [4] | | 20.75 | 22.00 | | [ABCD3, ACAA1, ACADM, ACAT1, ACSF3, ALDH5A1, ALDH6A1, ATP2B4, BCKDHA, CROT, ECI2, ETFA, ETFDH, FAAH, GCDH, GOT1, HOGA1, IMPA1, LONP2, SLC25A17, SLC27A2, SORD] |
|  | GO 0016054 organic acid catabolic process | 3.6E-6 | 110.0E-6 | | 36.0E-9 | 300.0E-9 | [4, 5] | | 22.73 | 20.00 | | [ABCD3, ACAA1, ACADM, ACAT1, ACSF3, ALDH5A1, ALDH6A1, ATP2B4, BCKDHA, CROT, ECI2, ETFA, ETFDH, FAAH, GCDH, GOT1, HOGA1, LONP2, SLC25A17, SLC27A2] |
|  | GO 0036503 ERAD pathway | 130.0E-6 | 1.6E-3 | | 36.0E-9 | 300.0E-9 | [3, 4, 5, 6] | | 22.22 | 14.00 | | [BAG6, BCAP31, DNAJB2, KIAA0368, MAN1B1, NPLOC4, OS9, TMUB1, TRIM25, UBAC2, UBXN1, VCP, VIMP, WFS1] |
|  | GO 0043436 oxoacid metabolic process | 920.0E-9 | 45.0E-6 | | 36.0E-9 | 300.0E-9 | [4, 5] | | 15.26 | 47.00 | | [ABCD3, ACAA1, ACADM, ACLY, ACO2, ACOT1, ACSF3, ALDH5A1, ALDH6A1, ALDOA, AMACR, ATP2B4, CARS, CROT, DECR2, ECI2, ELOVL5, ETFA, ETFDH, FAAH, FARSA, FARSB, GATB, GCDH, GOT1, HARS2, HOGA1, IDH1, KARS, LARS2, LONP2, LTA4H, MARC1, MARS2, MCAT, MGST2, MLYCD, MTHFD1, PARK7, PDHA1, PDHB, PFKP, QARS, SCD, SLC25A17, SLC27A2, TECR] |
|  | GO 0006520 cellular amino acid metabolic process | 1.3E-3 | 9.6E-3 | | 36.0E-9 | 300.0E-9 | [3, 4, 6, 7] | | 17.95 | 14.00 | | [ALDH5A1, ALDH6A1, ATP2B4, CARS, FARSA, FARSB, GATB, GOT1, HARS2, KARS, LARS2, MARS2, MTHFD1, QARS] |
|  | GO 0006631 fatty acid metabolic process | 740.0E-6 | 6.0E-3 | | 36.0E-9 | 300.0E-9 | [4, 5, 7, 8] | | 15.71 | 22.00 | | [ABCD3, ACAA1, ACADM, ACOT1, ACSF3, CROT, DECR2, ECI2, ELOVL5, ETFA, ETFDH, FAAH, GCDH, LONP2, LTA4H, MCAT, MGST2, MLYCD, SCD, SLC25A17, SLC27A2, TECR] |
|  | GO 0046395 carboxylic acid catabolic process | 7.3E-6 | 190.0E-6 | | 36.0E-9 | 300.0E-9 | [5, 6, 7] | | 23.38 | 18.00 | | [ABCD3, ACAA1, ACADM, ACSF3, ALDH5A1, ALDH6A1, ATP2B4, CROT, ECI2, ETFA, ETFDH, FAAH, GCDH, GOT1, HOGA1, LONP2, SLC25A17, SLC27A2] |
|  | GO 0032787 monocarboxylic acid metabolic process | 620.0E-6 | 5.2E-3 | | 36.0E-9 | 300.0E-9 | [6, 7] | | 13.94 | 29.00 | | [ABCD3, ACAA1, ACADM, ACOT1, ACSF3, ALDOA, AMACR, CROT, DECR2, ECI2, ELOVL5, ETFA, ETFDH, FAAH, GCDH, HOGA1, LONP2, LTA4H, MCAT, MGST2, MLYCD, PARK7, PDHA1, PDHB, PFKP, SCD, SLC25A17, SLC27A2, TECR] |
|  | GO 0006633 fatty acid biosynthetic process | 12.0E-3 | 48.0E-3 | | 36.0E-9 | 300.0E-9 | [5, 6, 7, 8, 9] | | 16.39 | 10.00 | | [ABCD3, ACSF3, DECR2, ELOVL5, LTA4H, MCAT, MGST2, MLYCD, SCD, TECR] |
|  | GO 0019395 fatty acid oxidation | 55.0E-6 | 820.0E-6 | | 36.0E-9 | 300.0E-9 | [5, 6, 7, 8, 9] | | 28.95 | 11.00 | | [ABCD3, ACAA1, ACADM, CROT, ETFA, ETFDH, GCDH, LONP2, MLYCD, SLC25A17, SLC27A2] |
|  | GO 0072329 monocarboxylic acid catabolic process | 2.7E-6 | 100.0E-6 | | 36.0E-9 | 300.0E-9 | [6, 7, 8] | | 32.50 | 13.00 | | [ABCD3, ACAA1, ACADM, CROT, ECI2, ETFA, ETFDH, FAAH, GCDH, HOGA1, LONP2, SLC25A17, SLC27A2] |
|  | GO 0033539 fatty acid beta-oxidation using acyl-CoA dehydrogenase | 810.0E-6 | 6.4E-3 | | 36.0E-9 | 300.0E-9 | [7, 8, 9, 10, 11] | | 57.14 | 4.00 | | [ACADM, ETFA, ETFDH, GCDH] |
|  | GO 0043523 regulation of neuron apoptotic process | 5.2E-3 | 26.0E-3 | | 5.2E-3 | 6.3E-3 | [5, 6, 7] | | 21.05 | 8.00 | | [BAX, CTNNB1, NAE1, PARK7, PPT1, SET, SOD2, WFS1] |
|  | GO 0048518 positive regulation of biological process | 890.0E-6 | 6.9E-3 | | 42.0E-9 | 290.0E-9 | [1, 2, 3] | | 6.07 | 186.00 | | [AAK1, ABHD14B, ACSL3, ACTN4, ACTR3, ADAR, AGR2, AHCYL1, ANKLE2, AP1G1, ARL6IP5, ATP1B3, ATP2B4, ATP7A, BAG6, BAX, BCAP31, BNIP3L, BRD4, C14orf166, CC2D1A, CCT2, CCT4, CCT5, CCT6A, CCT7, CCT8, CD276, CDH1, CDK5RAP3, CENPE, CHERP, CIB1, CNOT1, CTNNB1, CTSH, CYB5R1, CYFIP1, DDRGK1, DLG1, DNAJA3, DNAJB2, DNM1L, DNMT1, EEF1D, EEF1E1, EEF2, EGFR, EIF5A, EPT1, ERBB2, ERP29, ETFA, ETFDH, EZR, FAF1, FASN, FLOT2, FN1, FXR1, GARS, GDF15, GIPC1, GNL3, GNL3L, GRB2, HSPA1A, HTRA2, HUWE1, IDE, ILK, IST1, JUP, KMT2D, LAMP1, LAMTOR1, LARP1, LMAN1, MACF1, MAP2K2, MAPT, MARK2, MLYCD, MMAB, MPP7, MTPN, MYADM, MYDGF, MYO18A, MYO6, NACC1, NAMPT, NBN, NCOA6, NDRG1, NDUFA2, NEDD4L, NOP10, NPEPPS, NSF, NUP62, PACSIN3, PAK2, PARK7, PDCD6IP, PELP1, PFN1, PHB, PHIP, PINX1, PNPLA2, PNPT1, PPIA, PPIE, PPP2CA, PPT1, PRDX5, PRKD1, PRMT1, PRMT5, PSMB7, PTPN11, PTPN23, PTPRJ, QARS, RAB3B, RAB3GAP2, RAB7A, RAB9A, RALA, RBM5, REST, RHOA, RNMT, RPL10, RPRD1B, RPS15A, RPS3, RPS4X, RPS6, RPSA, RRAS, RUVBL1, SCRIB, SDCBP, SEPT9, SHC1, SHTN1, SIN3A, SLC35B2, SLC44A2, SLC9A1, SMARCB1, SMARCC2, SNIP1, SPAG9, SPEN, SRI, STRN3, STX18, SUPV3L1, TBC1D5, TCP1, TFAM, TKFC, TMPRSS2, TNPO1, TOM1L1, TOR1A, TP53BP1, TPD52L1, TRIM25, TRIOBP, TSG101, U2AF1, UBE2V1, UFL1, USP47, UTRN, VAMP7, VCP, WASF2, WDR46, WFS1, YTHDF2, ZC3HAV1] |
|  | GO 0019222 regulation of metabolic process | 17.0E-6 | 370.0E-6 | | 42.0E-9 | 290.0E-9 | [2, 3] | | 5.65 | 165.00 | | [ABCE1, ABHD14B, ACSL3, ACTN4, ADAR, AGR2, AHCYL1, ANKLE2, ANXA7, ARFGEF3, ARL6IP5, ATP2B4, ATP7A, ATXN2, BAG6, BAX, BCAP31, BCCIP, BRD4, C14orf166, CCT2, CCT4, CCT5, CCT6A, CCT7, CCT8, CD276, CDH1, CDK11B, CDK5RAP3, CENPE, CHMP1A, CIB1, CNOT1, CTNNB1, CTSH, DDRGK1, DLG1, DNAJA3, DNAJB2, DNAJC3, DNMT1, EEF2, EGFR, EIF2A, EIF3B, EIF4G2, EIF5, EIF5B, EIF6, ENO1, ERBB2, ERP29, ETF1, ETFA, ETFDH, EZR, FAF1, FLOT2, FN1, FXR1, GARS, GNL3, GNL3L, GRB2, HSP90AA1, HSPA1A, HTRA2, ILK, IST1, JUP, KMT2D, LAMTOR1, LIG3, LONP2, MAP2K2, ME2, MLYCD, MMAB, MYADM, MYDGF, MYO1D, MYO6, NACC1, NAMPT, NBN, NCOA6, NSF, NUP62, OS9, OTUB1, PACSIN3, PAK2, PARK7, PDCD4, PELP1, PHB, PHF21A, PHIP, PINX1, PNPLA2, PNPT1, PPIE, PPP2CA, PQBP1, PRDX5, PRKD1, PTPRJ, QARS, RAB3GAP2, RAB7A, RBFOX2, RBM5, RCOR1, REST, RHOA, RNMT, RPA2, RPRD1B, RPS3, RPS4X, RRAS, RRP1B, SAP130, SCRIB, SDCBP, SERPINB6, SET, SFN, SHC1, SIGIRR, SIN3A, SLC27A1, SLC9A1, SMARCB1, SMARCC2, SNIP1, SOD2, SPEN, STRN3, SUPV3L1, SYNJ2BP, TBC1D5, TCP1, TFAM, TOM1L1, TP53BP1, TPD52L1, TRAP1, TRIM25, TSG101, UBAC2, UBE2V1, UBXN1, UCHL5, UFL1, USP47, VCP, VIMP, WFS1, WNK1, YEATS2, YTHDF2, YWHAE, ZC3HAV1] |
|  | GO 0048583 regulation of response to stimulus | 3.5E-3 | 18.0E-3 | | 42.0E-9 | 290.0E-9 | [2, 3] | | 5.70 | 100.00 | | [AAK1, ACTN4, AGR2, ANXA5, AP1G1, ARHGDIA, ARL6IP5, ATP2B4, BAG6, BAX, BCAP31, BRD4, CC2D1A, CDC37, CDK5RAP3, CIB1, CNOT1, CTSH, DDRGK1, DLG1, DNAJA3, DNAJC3, DNM1L, EEF1D, EEF1E1, EGFR, ELMOD2, ERBB2, ERP29, ETFA, ETFDH, EZR, FAF1, FLOT2, FN1, HSPA1A, HTRA2, ILK, IST1, ITPR1, KMT2D, LAMP1, LAMTOR1, MAP2K2, MDH1, MFN2, MMAB, MTPN, MYADM, MYDGF, NUP62, OPA1, OS9, OTUB1, PAK2, PARK7, PEA15, PEX13, PHB, PHIP, PNPT1, PPIF, PRKD1, PRMT5, PTPN11, PTPRJ, RAB7A, RHOA, RNF213, RNMT, RPA2, RPL10, RPS3, RPSA, RRAS, SDCBP, SHC1, SIN3A, SLC35B2, SLC44A2, SLC9A1, SMARCB1, SNRPB, SOD2, SRI, SYNJ2BP, TKFC, TMBIM1, TP53BP1, TPD52L1, TRIM25, TSG101, UBAC2, UBE2V1, UBXN1, UFL1, VAMP7, VIMP, WFS1, ZC3HAV1] |
|  | GO 0050794 regulation of cellular process | 1.6E-6 | 70.0E-6 | | 42.0E-9 | 290.0E-9 | [2, 3] | | 6.13 | 292.00 | | [AAK1, ABCE1, ABHD14B, ACSL3, ACTN1, ACTN4, ACTR3, ADAR, AGR2, AHCYL1, AIMP1, ALDOA, ANKLE2, ANXA5, AP1G1, ARFGEF3, ARHGDIA, ARL6IP5, ATP1B3, ATP2A2, ATP2B4, ATP6V0A1, ATXN2, ATXN2L, BAG6, BAIAP2, BAX, BCAP31, BCCIP, BET1L, BNIP3L, BRD4, C14orf166, CAPZB, CAST, CC2D1A, CCT2, CCT4, CCT5, CCT6A, CCT7, CCT8, CD276, CDC27, CDC37, CDC5L, CDH1, CDK11B, CDK5RAP3, CENPE, CHERP, CHMP1A, CIB1, CNOT1, CTNNB1, CTSA, CTSH, CYB5R1, CYFIP1, DDRGK1, DLG1, DNAJA3, DNAJB2, DNAJC1, DNAJC3, DNM1L, DNM2, DNMT1, DSG2, DSP, EEF1D, EEF1E1, EEF2, EGFR, EIF2A, |
|  | GO 0007154 cell communication | 1.4E-3 | 10.0E-3 | | 42.0E-9 | 290.0E-9 | [3] | | 5.76 | 121.00 | | [AAK1, ACTN4, AGR2, AHCYL1, AIMP1, ARHGDIA, ARL6IP5, ATP2A2, ATP2B4, BAG6, BAX, BCAP31, BRD4, CC2D1A, CDC37, CDC5L, CDK5RAP3, CHMP1A, CIB1, CNOT1, CTNNB1, CTSH, CYFIP1, DDRGK1, DLG1, DNAJA3, DNM1L, DNMT1, DSG2, DSP, EEF1D, EGFR, EPHB3, ERBB2, ERP29, ETFA, ETFDH, EZR, FAF1, FARP2, FASN, FLOT2, FN1, GARS, GNAI3, GRB2, HSPA1A, HTRA2, ILK, IMPA1, ITPR1, JUP, KMT2D, LAMP1, LAMTOR1, LARP1, MAP2K2, MARK2, MFN2, MMAB, MYADM, MYH14, MYO6, NAMPT, NDRG1, NUP62, OPA1, PAK2, PARK7, PEA15, PHB, PHIP, PPIF, PRKD1, PRMT5, PRPF19, PTPN11, PTPRJ, RAB3B, RAB7A, RAP1A, RBFOX2, REST, RHOA, RHOT1, RNF213, RNMT, RPL10, RPS3, RPS6, RPSA, RRAS, SDCBP, SFN, SHC1, SLC35B2, SLC44A2, SLC9A1, SMARCB1, SOD2, SRI, STRN, SYNJ2BP, TKFC, TMBIM1, TOLLIP, TOM1L1, TOM1L2, TOR1A, TP53BP1, TPD52L1, TRIM25, TSG101, UBE2V1, UFL1, VAMP7, VIMP, WFS1, WNK1, YWHAG, ZC3HAV1] |
|  | GO 0009893 positive regulation of metabolic process | 68.0E-6 | 1.0E-3 | | 42.0E-9 | 290.0E-9 | [2, 3, 4] | | 5.19 | 93.00 | | [ABHD14B, ACSL3, AGR2, ANKLE2, ARL6IP5, ATP2B4, ATP7A, BAG6, BAX, BCAP31, BRD4, C14orf166, CCT2, CCT4, CCT5, CCT6A, CCT7, CCT8, CD276, CDH1, CDK5RAP3, CENPE, CNOT1, CTNNB1, CTSH, DDRGK1, DNAJA3, DNAJB2, DNMT1, EEF2, EGFR, ERBB2, ERP29, ETFA, ETFDH, EZR, FN1, GARS, GNL3, GRB2, HSPA1A, HTRA2, ILK, IST1, KMT2D, MAP2K2, MLYCD, MYDGF, MYO6, NAMPT, NBN, NCOA6, NSF, NUP62, PACSIN3, PAK2, PARK7, PELP1, PHB, PHIP, PNPLA2, PNPT1, PPP2CA, PRDX5, PRKD1, QARS, RAB3GAP2, RAB7A, REST, RHOA, RNMT, RPRD1B, RPS3, RPS4X, SCRIB, SDCBP, SHC1, SIN3A, SLC9A1, SMARCB1, SMARCC2, STRN3, SUPV3L1, TBC1D5, TCP1, TFAM, TOM1L1, TP53BP1, TPD52L1, VCP, WFS1, YTHDF2, ZC3HAV1] |
|  | GO 0048522 positive regulation of cellular process | 3.8E-3 | 20.0E-3 | | 42.0E-9 | 290.0E-9 | [2, 3, 4] | | 6.14 | 172.00 | | [AAK1, ABHD14B, ACSL3, ACTN4, ACTR3, ADAR, AGR2, ANKLE2, AP1G1, ARL6IP5, ATP1B3, ATP2B4, BAG6, BAX, BCAP31, BNIP3L, BRD4, C14orf166, CC2D1A, CCT2, CCT4, CCT5, CCT6A, CCT7, CCT8, CD276, CDH1, CDK5RAP3, CENPE, CIB1, CNOT1, CTNNB1, CTSH, CYB5R1, CYFIP1, DDRGK1, DLG1, DNAJA3, DNAJB2, DNM1L, DNMT1, EEF1D, EEF1E1, EEF2, EGFR, EIF5A, EPT1, ERBB2, ERP29, ETFA, ETFDH, EZR, FAF1, FASN, FLOT2, FN1, FXR1, GARS, GDF15, GIPC1, GNL3, GRB2, HSPA1A, HTRA2, HUWE1, IDE, ILK, IST1, KMT2D, LAMP1, LAMTOR1, LARP1, LMAN1, MACF1, MAP2K2, MAPT, MARK2, MLYCD, MMAB, MPP7, MYADM, MYDGF, MYO18A, MYO6, NACC1, NAMPT, NBN, NCOA6, NDRG1, NEDD4L, NPEPPS, NSF, NUP62, PACSIN3, PAK2, PARK7, PDCD6IP, PELP1, PFN1, PHB, PHIP, PNPLA2, PNPT1, PPIA, PPIE, PPP2CA, PPT1, PRKD1, PRMT1, PRMT5, PSMB7, PTPN11, PTPN23, PTPRJ, QARS, RAB3B, RAB3GAP2, RAB7A, RAB9A, RALA, RBM5, REST, RHOA, RNMT, RPL10, RPRD1B, RPS15A, RPS3, RPS4X, RPS6, RPSA, RUVBL1, SCRIB, SDCBP, SEPT9, SHC1, SHTN1, SIN3A, SLC35B2, SLC44A2, SLC9A1, SMARCB1, SMARCC2, SNIP1, SPAG9, SPEN, SRI, STRN3, STX18, SUPV3L1, TBC1D5, TCP1, TFAM, TMPRSS2, TOM1L1, TOR1A, TP53BP1, TPD52L1, TRIM25, TRIOBP, TSG101, U2AF1, UBE2V1, UFL1, USP47, VAMP7, VCP, WASF2, WDR46, WFS1, YTHDF2, ZC3HAV1] |
|  | GO 0007165 signal transduction | 560.0E-6 | 4.9E-3 | | 42.0E-9 | 290.0E-9 | [3, 4] | | 5.58 | 110.00 | | [AAK1, ACTN4, AGR2, AHCYL1, ARHGDIA, ARL6IP5, ATP2A2, ATP2B4, BAG6, BAX, BCAP31, BRD4, CC2D1A, CDC37, CDC5L, CDK5RAP3, CIB1, CNOT1, CTNNB1, CTSH, CYFIP1, DDRGK1, DLG1, DNAJA3, DNM1L, DNMT1, EEF1D, EGFR, EPHB3, ERBB2, ERP29, ETFA, ETFDH, EZR, FAF1, FARP2, FASN, FLOT2, FN1, GARS, GNAI3, GRB2, HSPA1A, HTRA2, ILK, IMPA1, ITPR1, KMT2D, LAMP1, LAMTOR1, LARP1, MAP2K2, MARK2, MFN2, MMAB, MYADM, MYO6, NAMPT, NDRG1, NUP62, OPA1, PAK2, PARK7, PEA15, PHB, PHIP, PPIF, PRKD1, PRMT5, PRPF19, PTPN11, PTPRJ, RAB7A, RAP1A, RBFOX2, RHOA, RHOT1, RNF213, RNMT, RPL10, RPS3, RPS6, RPSA, RRAS, SDCBP, SFN, SHC1, SLC35B2, SLC44A2, SLC9A1, SMARCB1, SOD2, SRI, STRN, SYNJ2BP, TKFC, TMBIM1, TOLLIP, TOM1L1, TOM1L2, TP53BP1, TPD52L1, TRIM25, TSG101, UBE2V1, UFL1, VIMP, WFS1, WNK1, ZC3HAV1] |
|  | GO 0031323 regulation of cellular metabolic process | 24.0E-6 | 500.0E-6 | | 42.0E-9 | 290.0E-9 | [3, 4] | | 5.63 | 155.00 | | [ABCE1, ABHD14B, ACTN4, ADAR, AHCYL1, ANKLE2, ARFGEF3, ARL6IP5, ATP2B4, ATXN2, BAX, BCAP31, BCCIP, BRD4, C14orf166, CCT2, CCT4, CCT5, CCT6A, CCT7, CCT8, CD276, CDH1, CDK11B, CDK5RAP3, CENPE, CHMP1A, CIB1, CNOT1, CTNNB1, CTSH, DDRGK1, DLG1, DNAJA3, DNAJB2, DNAJC3, DNMT1, EEF2, EGFR, EIF2A, EIF3B, EIF4G2, EIF5, EIF5B, EIF6, ENO1, ERBB2, ERP29, ETF1, ETFA, ETFDH, EZR, FN1, FXR1, GARS, GNL3, GNL3L, GRB2, HSP90AA1, HSPA1A, HTRA2, ILK, IST1, JUP, KMT2D, LAMTOR1, LIG3, LONP2, MAP2K2, ME2, MLYCD, MMAB, MYADM, MYDGF, MYO1D, MYO6, NACC1, NAMPT, NBN, NCOA6, NSF, NUP62, OTUB1, PACSIN3, PAK2, PARK7, PDCD4, PELP1, PHB, PHF21A, PHIP, PINX1, PNPLA2, PNPT1, PPIE, PPP2CA, PQBP1, PRDX5, PRKD1, PTPRJ, QARS, RAB3GAP2, RBFOX2, RBM5, RCOR1, REST, RHOA, RNMT, RPA2, RPRD1B, RPS3, RPS4X, RRAS, RRP1B, SAP130, SCRIB, SDCBP, SERPINB6, SET, SFN, SHC1, SIGIRR, SIN3A, SLC27A1, SLC9A1, SMARCB1, SMARCC2, SNIP1, SOD2, SPEN, STRN3, SUPV3L1, SYNJ2BP, TBC1D5, TCP1, TFAM, TOM1L1, TP53BP1, TPD52L1, TRAP1, TRIM25, TSG101, UBE2V1, UBXN1, UCHL5, UFL1, USP47, VCP, VIMP, WFS1, WNK1, YEATS2, YTHDF2, YWHAE, ZC3HAV1] |
|  | GO 0060255 regulation of macromolecule metabolic process | 27.0E-6 | 550.0E-6 | | 42.0E-9 | 290.0E-9 | [3, 4] | | 5.60 | 152.00 | | [ABCE1, ABHD14B, ACTN4, ADAR, AGR2, AHCYL1, ANKLE2, ANXA7, ARL6IP5, ATP2B4, ATXN2, BAG6, BAX, BCAP31, BCCIP, BRD4, C14orf166, CCT2, CCT4, CCT5, CCT6A, CCT7, CCT8, CD276, CDH1, CDK11B, CDK5RAP3, CENPE, CHMP1A, CIB1, CNOT1, CTNNB1, CTSH, DDRGK1, DLG1, DNAJA3, DNAJB2, DNAJC3, DNMT1, EEF2, EGFR, EIF2A, EIF3B, EIF4G2, EIF5, EIF5B, EIF6, ENO1, ERBB2, ERP29, ETF1, ETFA, ETFDH, EZR, FAF1, FLOT2, FN1, FXR1, GARS, GNL3, GNL3L, HSP90AA1, HSPA1A, HTRA2, ILK, IST1, JUP, KMT2D, LIG3, MAP2K2, MMAB, MYADM, MYDGF, MYO6, NACC1, NAMPT, NBN, NCOA6, NSF, NUP62, OS9, OTUB1, PACSIN3, PAK2, PARK7, PDCD4, PELP1, PHB, PHF21A, PHIP, PINX1, PNPT1, PPIE, PPP2CA, PQBP1, PRDX5, PRKD1, PTPRJ, QARS, RAB3GAP2, RAB7A, RBFOX2, RBM5, RCOR1, REST, RHOA, RNMT, RPA2, RPRD1B, RPS3, RPS4X, RRAS, SAP130, SCRIB, SDCBP, SERPINB6, SET, SFN, SHC1, SIGIRR, SIN3A, SLC9A1, SMARCB1, SMARCC2, SNIP1, SOD2, SPEN, STRN3, SUPV3L1, SYNJ2BP, TBC1D5, TCP1, TFAM, TOM1L1, TP53BP1, TPD52L1, TRAP1, TRIM25, TSG101, UBAC2, UBE2V1, UBXN1, UCHL5, UFL1, USP47, VCP, VIMP, WFS1, YEATS2, YTHDF2, YWHAE, ZC3HAV1] |
|  | GO 0080090 regulation of primary metabolic process | 33.0E-6 | 620.0E-6 | | 42.0E-9 | 290.0E-9 | [3, 4] | | 5.63 | 152.00 | | [ABCE1, ABHD14B, ACTN4, ADAR, AHCYL1, ANKLE2, ARL6IP5, ATP2B4, BAG6, BAX, BCAP31, BCCIP, BRD4, C14orf166, CCT2, CCT4, CCT5, CCT6A, CCT7, CCT8, CD276, CDH1, CDK11B, CDK5RAP3, CENPE, CHMP1A, CIB1, CNOT1, CTNNB1, CTSH, DDRGK1, DLG1, DNAJA3, DNAJB2, DNAJC3, DNMT1, EEF2, EGFR, EIF2A, EIF3B, EIF4G2, EIF5, EIF5B, EIF6, ENO1, ERBB2, ERP29, ETF1, ETFA, ETFDH, EZR, FAF1, FLOT2, FN1, FXR1, GARS, GNL3, GNL3L, HSP90AA1, HSPA1A, HTRA2, ILK, IST1, JUP, KMT2D, LAMTOR1, LIG3, LONP2, MAP2K2, ME2, MLYCD, MMAB, MYADM, MYDGF, MYO6, NACC1, NAMPT, NBN, NCOA6, NSF, NUP62, OS9, OTUB1, PACSIN3, PAK2, PARK7, PDCD4, PELP1, PHB, PHF21A, PHIP, PINX1, PNPLA2, PNPT1, PPIE, PPP2CA, PQBP1, PRDX5, PRKD1, PTPRJ, QARS, RAB3GAP2, RAB7A, RBFOX2, RBM5, RCOR1, REST, RHOA, RNMT, RPA2, RPRD1B, RPS3, RPS4X, RRAS, SAP130, SDCBP, SERPINB6, SET, SFN, SHC1, SIGIRR, SIN3A, SLC27A1, SLC9A1, SMARCB1, SMARCC2, SNIP1, SOD2, SPEN, STRN3, SUPV3L1, SYNJ2BP, TCP1, TFAM, TOM1L1, TP53BP1, TPD52L1, TRAP1, TRIM25, TSG101, UBAC2, UBE2V1, UBXN1, UCHL5, UFL1, USP47, VCP, WFS1, YEATS2, YTHDF2, YWHAE, ZC3HAV1] |
|  | GO 0010604 positive regulation of macromolecule metabolic process | 140.0E-6 | 1.6E-3 | | 42.0E-9 | 290.0E-9 | [3, 4, 5] | | 5.21 | 88.00 | | [ABHD14B, AGR2, ANKLE2, ARL6IP5, ATP2B4, BAG6, BAX, BCAP31, BRD4, C14orf166, CCT2, CCT4, CCT5, CCT6A, CCT7, CCT8, CD276, CDH1, CDK5RAP3, CENPE, CNOT1, CTNNB1, CTSH, DDRGK1, DNAJA3, DNAJB2, DNMT1, EEF2, EGFR, ERBB2, ERP29, ETFA, ETFDH, EZR, FN1, GARS, GNL3, HSPA1A, HTRA2, ILK, IST1, KMT2D, MAP2K2, MYDGF, MYO6, NAMPT, NBN, NCOA6, NSF, NUP62, PACSIN3, PAK2, PARK7, PELP1, PHB, PHIP, PNPT1, PPP2CA, PRDX5, PRKD1, QARS, RAB3GAP2, RAB7A, REST, RHOA, RNMT, RPRD1B, RPS3, RPS4X, SCRIB, SDCBP, SHC1, SIN3A, SLC9A1, SMARCB1, SMARCC2, STRN3, SUPV3L1, TBC1D5, TCP1, TFAM, TOM1L1, TP53BP1, TPD52L1, VCP, WFS1, YTHDF2, ZC3HAV1] |
|  | GO 0031325 positive regulation of cellular metabolic process | 72.0E-6 | 1.0E-3 | | 42.0E-9 | 290.0E-9 | [3, 4, 5] | | 5.11 | 86.00 | | [ABHD14B, ANKLE2, ARL6IP5, ATP2B4, BAX, BCAP31, BRD4, C14orf166, CCT2, CCT4, CCT5, CCT6A, CCT7, CCT8, CD276, CDH1, CDK5RAP3, CENPE, CNOT1, CTNNB1, CTSH, DDRGK1, DNAJA3, DNAJB2, DNMT1, EEF2, EGFR, ERBB2, ERP29, ETFA, ETFDH, EZR, GARS, GNL3, GRB2, HSPA1A, HTRA2, ILK, IST1, KMT2D, MAP2K2, MLYCD, MYDGF, MYO6, NAMPT, NBN, NCOA6, NSF, NUP62, PACSIN3, PAK2, PARK7, PELP1, PHB, PHIP, PNPLA2, PNPT1, PPP2CA, PRKD1, QARS, RAB3GAP2, REST, RHOA, RNMT, RPRD1B, RPS3, RPS4X, SCRIB, SDCBP, SHC1, SIN3A, SLC9A1, SMARCB1, SMARCC2, STRN3, SUPV3L1, TBC1D5, TCP1, TFAM, TOM1L1, TP53BP1, TPD52L1, VCP, WFS1, YTHDF2, ZC3HAV1] |
|  | GO 0006887 exocytosis | 7.3E-3 | 34.0E-3 | | 7.3E-3 | 8.1E-3 | [4, 5, 6, 7] | | 15.22 | 14.00 | | [AP1G1, LAMP1, PDCD6IP, RAB11B, RAB7A, RAB9A, RALA, SDCBP, STEAP2, STXBP1, STXBP2, STXBP3, TSG101, VAMP7] |
|  | GO 1903551 regulation of extracellular exosome assembly | 6.5E-3 | 32.0E-3 | | 7.3E-3 | 8.1E-3 | [4, 5, 6, 7] | | 50.00 | 3.00 | | [PDCD6IP, SDCBP, TSG101] |
|  | GO 0017157 regulation of exocytosis | 12.0E-3 | 48.0E-3 | | 7.3E-3 | 8.1E-3 | [4, 5, 6, 7, 8] | | 16.39 | 10.00 | | [AP1G1, LAMP1, PDCD6IP, RAB7A, RAB9A, RALA, SDCBP, STXBP3, TSG101, VAMP7] |
|  | GO 0043320 natural killer cell degranulation | 3.4E-3 | 18.0E-3 | | 7.3E-3 | 8.1E-3 | [3, 4, 5, 6, 7, 8, 9, 10] | | 60.00 | 3.00 | | [AP1G1, LAMP1, VAMP7] |
|  | GO 0045921 positive regulation of exocytosis | 8.4E-3 | 39.0E-3 | | 7.3E-3 | 8.1E-3 | [4, 5, 6, 7, 8, 9] | | 19.51 | 8.00 | | [AP1G1, LAMP1, PDCD6IP, RAB7A, RAB9A, SDCBP, TSG101, VAMP7] |
|  | GO 0043302 positive regulation of leukocyte degranulation | 10.0E-3 | 45.0E-3 | | 7.3E-3 | 8.1E-3 | [4, 5, 6, 7, 8, 9, 10, 11] | | 42.86 | 3.00 | | [AP1G1, LAMP1, VAMP7] |
|  | GO 0048284 organelle fusion | 930.0E-6 | 7.1E-3 | | 230.0E-6 | 480.0E-6 | [4] | | 19.40 | 13.00 | | [BAX, BNIP1, EEA1, GDAP1, MFN1, MFN2, OPA1, RAB7A, SNAP29, VAMP7, VCP, VPS33A, VTI1B] |
|  | GO 0061025 membrane fusion | 1.5E-3 | 10.0E-3 | | 230.0E-6 | 480.0E-6 | [4] | | 20.37 | 11.00 | | [BAX, BNIP1, CD9, DNM1L, EEA1, GDAP1, MFN1, MFN2, OPA1, RAB7A, VAMP7] |
|  | GO 0034109 homotypic cell-cell adhesion | 180.0E-6 | 2.0E-3 | | 180.0E-6 | 420.0E-6 | [4, 5] | | 24.00 | 12.00 | | [ACTN1, CXADR, DSG2, DSP, ILK, JUP, MYH9, MYL12A, STXBP1, STXBP3, TLN1, VCL] |
|  | GO 0070527 platelet aggregation | 3.0E-3 | 17.0E-3 | | 180.0E-6 | 420.0E-6 | [5, 6, 7] | | 22.86 | 8.00 | | [ACTN1, ILK, MYH9, MYL12A, STXBP1, STXBP3, TLN1, VCL] |
|  | GO 0071569 protein ufmylation | 6.5E-3 | 32.0E-3 | | 6.5E-3 | 7.6E-3 | [8, 9] | | 50.00 | 3.00 | | [CDK5RAP3, DDRGK1, UFL1] |
|  | GO 0071681 cellular response to indole-3-methanol | 6.5E-3 | 32.0E-3 | | 6.5E-3 | 7.6E-3 | [5, 6] | | 50.00 | 3.00 | | [CDH1, CTNNB1, JUP] |
|  | GO 0044281 small molecule metabolic process | 2.8E-6 | 98.0E-6 | | 6.1E-9 | 83.0E-9 | [3] | | 11.89 | 86.00 | | [ABCD3, ACAA1, ACADM, ACAT1, ACLY, ACO2, ACOT1, ACSF3, ADCK3, AK1, AK3, ALDH3A2, ALDH5A1, ALDH6A1, ALDOA, AMACR, ATP2B4, ATP5B, ATP5O, BCKDHA, CAD, CARS, CBR1, COA6, COASY, COQ3, CROT, DDAH1, DDAH2, DECR2, DHCR7, DHRS4, DPM1, ECI2, ELOVL5, ETFA, ETFDH, FAAH, FARSA, FARSB, GARS, GATB, GCDH, GLB1, GNAI3, GOT1, GUK1, HARS2, HOGA1, HSPA1A, IDH1, IMPA1, KARS, LARS2, LONP2, LSS, LTA4H, MACROD1, MARC1, MARS2, MCAT, ME2, MGST2, MLYCD, MTHFD1, MUT, NDUFS1, OLA1, OXCT1, PARK7, PDHA1, PDHB, PECR, PFKP, PGD, PMVK, PRDX5, QARS, QTRT1, REST, SCD, SLC25A17, SLC27A2, SORD, TECR, VCP] |
|  | GO 0072350 tricarboxylic acid metabolic process | 1.4E-3 | 10.0E-3 | | 1.4E-3 | 2.2E-3 | [6, 7] | | 75.00 | 3.00 | | [ACLY, ACO2, IDH1] |
|  | GO 0007029 endoplasmic reticulum organization | 3.2E-3 | 17.0E-3 | | 78.0E-6 | 230.0E-6 | [4] | | 25.00 | 7.00 | | [BAG6, BNIP1, RAB10, RAB3GAP2, RTN4, SEC61A1, STX18] |
| **Down DEPs in** **MDB vs PDB** | | | | | | | | | | | | |
|  | GO:0002682 regulation of immune system process | 3.0E-3 | | 17.0E-3 | 960.0E-6 | 1.5E-3 | [2, 3] | 6.58 | | 40.00 | [ALKBH5, ANXA5, ATP1B1, BAX, CD276, CD81, CDC37, CDC73, CTR9, CTSH, DNAJA3, DUSP3, ELMOD2, ERBB2IP, EZR, FLOT2, HMGB2, LAMP1, MAFB, MDH1, MRPS10, MYDGF, OGT, PEX13, PHB, PRMT1, PTPN2, PTPRJ, RAC1, RAC2, RPL10, RPSA, SIN3A, SNRPB, TRIM27, VAMP7, VIMP, WDR61, ZC3HAV1, ZCCHC17] | |
|  | GO:0001775 cell activation | 1.8E-3 | | 12.0E-3 | 960.0E-6 | 1.5E-3 | [3] | 4.83 | | 14.00 | [ACTN1, CD276, CD81, DUSP3, FLOT2, GBF1, LAMP1, MYDGF, MYL12A, MYL9, PRMT5, RPSA, STXBP1, VAMP7] | |
|  | GO:0002684 positive regulation of immune system process | 1.5E-3 | | 10.0E-3 | 960.0E-6 | 1.5E-3 | [2, 3, 4] | 5.26 | | 19.00 | [BAX, CD276, CD81, DUSP3, ERBB2IP, EZR, FLOT2, HMGB2, LAMP1, OGT, PHB, PRMT1, PTPRJ, RAC1, RAC2, RPSA, SIN3A, VAMP7, ZC3HAV1] | |
|  | GO:0006414 translational elongation | 2.6E-3 | | 16.0E-3 | 140.0E-9 | 590.0E-9 | [5, 6, 7, 8] | 57.14 | | 4.00 | [GFM1, GFM2, MRPL44, TUFM] | |
|  | GO:0032543 mitochondrial translation | 130.0E-6 | | 1.7E-3 | 140.0E-9 | 590.0E-9 | [5, 6, 7, 8] | 40.91 | | 9.00 | [COA3, GATB, GATC, GFM1, GFM2, MRPL44, MTG1, PTCD3, QRSL1] | |
|  | GO:0043039 tRNA aminoacylation | 19.0E-6 | | 420.0E-6 | 140.0E-9 | 590.0E-9 | [5, 6, 7, 8, 9] | 50.00 | | 9.00 | [CARS, FARSB, GATB, GATC, HARS2, KARS, MARS2, QARS, QRSL1] | |
|  | GO:0070681 glutaminyl-tRNAGln biosynthesis via transamidation | 980.0E-6 | | 7.8E-3 | 140.0E-9 | 590.0E-9 | [6, 7, 8, 9, 10] | 100.00 | | 3.00 | [GATB, GATC, QRSL1] | |
|  | **GO:0043967 histone H4 acetylation** | 930.0E-6 | | 7.5E-3 | 520.0E-9 | 1.6E-6 | [8, 9, 11, 12] | 26.00 | | 13.00 | [BRCA1, BRD8, CTBP1, DMAP1, HCFC1, ING3, MEAF6, OGT, RUVBL1, RUVBL2, SMARCB1, TRRAP, YEATS4] | |
|  | **GO:0043968 histone H2A acetylation** | 3.2E-6 | | 100.0E-6 | 520.0E-9 | 1.6E-6 | [8, 9, 11, 12] | 66.67 | | 8.00 | [BRD8, DMAP1, ING3, MEAF6, RUVBL1, RUVBL2, TRRAP, YEATS4] | |
|  | **GO:0051573 negative regulation of histone H3-K9 methylation** | 8.4E-3 | | 37.0E-3 | 520.0E-9 | 1.6E-6 | [7, 8, 9, 10, 11, 12, 13] | 60.00 | | 3.00 | [BRCA1, DNMT1, SMARCB1] | |
|  | GO:0043933 macromolecular complex subunit organization | 2.2E-6 | | 73.0E-6 | 520.0E-9 | 1.6E-6 | [3] | 14.17 | | 152.00 | [ABL1, ACAD9, ACOT13, ACTR2, ACTR3, ADAR, ALDH5A1, AMFR, ARID2, ARPIN, ATF7IP, ATL2, ATXN2, ATXN2L, BAD, BAX, BAZ1B, BCOR, BRCA1, BRD2, BRD4, BRD7, BRD8, CAND1, CCT2, CDC73, CHD6, CLDN3, COA3, COA6, COG4, CTBP1, CTR9, DDX6, DERL1, DHRS4, DHX30, DMAP1, DNM1L, DNMT1, DPY30, DR1, EHMT2, EIF2A, EIF3A, EIF6, EPS15, FARSB, FASTKD2, FBL, FN1, FOXRED1, GARS, GEMIN5, GFM2, GRHPR, HCFC1, HIRA, HMOX1, HUWE1, IDE, ING3, JUN, KDM1A, KIF2C, MAPT, MAT2A, MBD2, MEAF6, MICAL3, MIPEP, MIS12, MMAB, MRRF, MTPN, MYADM, NACC1, NCLN, NDC1, NDUFAF4, NDUFS8, NOC2L, NPM1, NSD1, NUBPL, NUDT21, NUP133, NUP153, OGT, PFN1, PHB, PHF2, PICALM, PKN1, PNPT1, POGZ, POLE3, PQBP1, PRKD1, PRMT1, PRMT5, PRPF19, PRPF8, PTPN11, RBM14, RBM5, RCOR1, REST, RNF40, RPS3, RTN4, RUVBL1, RUVBL2, SAMM50, SAP130, SETD2, SIN3A, SKI, SMARCB1, SMARCD1, SMIM20, SNRPB, SNRPC, SOD2, SPTY2D1, SUPT6H, SUPV3L1, SURF1, TAF6L, TAF9, TFIP11, TIMM21, TNPO1, TOP1, TOR1A, TP53BP1, TPR, TRIM27, TRIM4, TRRAP, TTC19, UHRF1, VCP, VMA21, VRK1, WDR1, WDR61, WHSC1L1, YEATS2, YEATS4, YTHDC1, ZW10] | |
|  | **GO:0006325 chromatin organization** | 27.0E-6 | | 540.0E-6 | 520.0E-9 | 1.6E-6 | [4] | 16.99 | | 61.00 | [ARID2, BAZ1B, BCOR, BRCA1, BRD2, BRD4, BRD7, BRD8, CDC73, CHD6, CTBP1, CTR9, DMAP1, DNMT1, DPY30, DR1, EHMT2, FBL, HCFC1, HIRA, HUWE1, ING3, KDM1A, MBD2, MEAF6, NOC2L, NPM1, NSD1, OGT, PHB, PHF2, PKN1, POLE3, PRKD1, PRMT1, RBM14, RCOR1, REST, RNF40, RUVBL1, RUVBL2, SAP130, SETD2, SIN3A, SMARCB1, SMARCD1, SPTY2D1, SUPT6H, SUPV3L1, TAF6L, TAF9, TOP1, TP53BP1, TPR, TRRAP, UHRF1, VRK1, WDR61, WHSC1L1, YEATS2, YEATS4] | |
|  | GO:0043414 macromolecule methylation | 110.0E-6 | | 1.5E-3 | 520.0E-9 | 1.6E-6 | [3, 4, 5] | 21.93 | | 25.00 | [ATF7IP, BCOR, BRCA1, CHD6, CMTR1, CTR9, DNMT1, DPY30, EHMT2, ETF1, FBL, GSPT1, MEPCE, MPHOSPH8, NSD1, NSUN2, OGT, PRMT1, PRMT5, SETD2, SMARCB1, SNRPB, SUPT6H, WDR61, WHSC1L1] | |
|  | **GO:1902275 regulation of chromatin organization** | 410.0E-6 | | 4.1E-3 | 520.0E-9 | 1.6E-6 | [4, 5] | 22.73 | | 20.00 | [BCOR, BRCA1, BRD7, CTBP1, CTR9, DNMT1, KDM1A, NOC2L, NSD1, OGT, PHF2, PRKD1, RNF40, RUVBL2, SIN3A, SMARCB1, SPTY2D1, SUPT6H, TPR, WDR61] | |
|  | **GO:0016569 chromatin modification** | 30.0E-6 | | 570.0E-6 | 520.0E-9 | 1.6E-6 | [5] | 18.08 | | 49.00 | [BAZ1B, BCOR, BRCA1, BRD7, BRD8, CDC73, CHD6, CTBP1, CTR9, DMAP1, DNMT1, DPY30, DR1, EHMT2, FBL, HCFC1, HUWE1, ING3, KDM1A, MBD2, MEAF6, NOC2L, NSD1, OGT, PHB, PKN1, POLE3, PRKD1, PRMT1, RBM14, RCOR1, REST, RNF40, RUVBL1, RUVBL2, SAP130, SETD2, SIN3A, SMARCB1, SUPT6H, TAF6L, TAF9, TRRAP, UHRF1, VRK1, WDR61, WHSC1L1, YEATS2, YEATS4] | |
|  | **GO:1905268 negative regulation of chromatin organization** | 830.0E-6 | | 7.2E-3 | 520.0E-9 | 1.6E-6 | [4, 5, 6] | 33.33 | | 9.00 | [BCOR, BRCA1, CTBP1, DNMT1, NOC2L, PHF2, SIN3A, SMARCB1, SUPT6H] | |
|  | **GO:1905269 positive regulation of chromatin organization** | 920.0E-6 | | 7.4E-3 | 520.0E-9 | 1.6E-6 | [4, 5, 6] | 25.00 | | 14.00 | [BRCA1, BRD7, CTBP1, CTR9, DNMT1, KDM1A, OGT, PRKD1, RNF40, RUVBL2, SIN3A, SMARCB1, TPR, WDR61] | |
|  | GO:0006479 protein methylation | 460.0E-6 | | 4.5E-3 | 520.0E-9 | 1.6E-6 | [4, 5, 6, 7, 8] | 22.47 | | 20.00 | [BCOR, BRCA1, CHD6, CTR9, DNMT1, DPY30, EHMT2, ETF1, FBL, GSPT1, NSD1, OGT, PRMT1, PRMT5, SETD2, SMARCB1, SNRPB, SUPT6H, WDR61, WHSC1L1] | |
|  | **GO:0031056 regulation of histone modification** | 660.0E-6 | | 6.1E-3 | 520.0E-9 | 1.6E-6 | [5, 6, 7, 8] | 23.29 | | 17.00 | [BCOR, BRCA1, BRD7, CTBP1, CTR9, DNMT1, KDM1A, NOC2L, NSD1, OGT, PRKD1, RNF40, RUVBL2, SIN3A, SMARCB1, SUPT6H, WDR61] | |
|  | GO:0043543 protein acylation | 2.0E-3 | | 13.0E-3 | 520.0E-9 | 1.6E-6 | [6, 7] | 18.12 | | 27.00 | [BAG6, BRCA1, BRD7, BRD8, CTBP1, DMAP1, DR1, HCFC1, ING3, MEAF6, NOC2L, OGT, PARK7, POLE3, RUVBL1, RUVBL2, SAP130, SIN3A, SMARCB1, TAF6L, TAF9, TRRAP, TSPO, YEATS2, YEATS4, ZDHHC3, ZDHHC5] | |
|  | **GO:0016571 histone methylation** | 2.1E-3 | | 13.0E-3 | 520.0E-9 | 1.6E-6 | [5, 6, 7, 8, 9] | 22.22 | | 16.00 | [BCOR, BRCA1, CHD6, CTR9, DNMT1, DPY30, EHMT2, FBL, NSD1, OGT, PRMT1, SETD2, SMARCB1, SUPT6H, WDR61, WHSC1L1] | |
|  | **GO:0031058 positive regulation of histone modification** | 3.0E-3 | | 17.0E-3 | 520.0E-9 | 1.6E-6 | [5, 6, 7, 8, 9] | 24.00 | | 12.00 | [BRCA1, BRD7, CTBP1, CTR9, DNMT1, KDM1A, OGT, PRKD1, RNF40, RUVBL2, SMARCB1, WDR61] | |
|  | GO:0006473 protein acetylation | 1.3E-3 | | 10.0E-3 | 520.0E-9 | 1.6E-6 | [7, 8] | 19.20 | | 24.00 | [BAG6, BRCA1, BRD7, BRD8, CTBP1, DMAP1, DR1, HCFC1, ING3, MEAF6, NOC2L, OGT, PARK7, POLE3, RUVBL1, RUVBL2, SAP130, SIN3A, SMARCB1, TAF6L, TAF9, TRRAP, YEATS2, YEATS4] | |
|  | GO:0018205 peptidyl-lysine modification | 33.0E-6 | | 600.0E-6 | 520.0E-9 | 1.6E-6 | [7, 8] | 19.60 | | 39.00 | [BAG6, BCOR, BRCA1, BRD7, BRD8, CHD6, CTBP1, CTR9, DMAP1, DNMT1, DPY30, DR1, EHMT2, GNL3, GNL3L, HCFC1, ING3, MEAF6, NOC2L, NSD1, OGT, PARK7, PIAS2, POLE3, RUVBL1, RUVBL2, SAP130, SENP1, SETD2, SIN3A, SMARCB1, SUPT6H, TAF6L, TAF9, TRRAP, UBA2, WDR61, YEATS2, YEATS4] | |
|  | **GO:0031060 regulation of histone methylation** | 3.1E-3 | | 18.0E-3 | 520.0E-9 | 1.6E-6 | [6, 7, 8, 9, 10] | 28.13 | | 9.00 | [BCOR, BRCA1, CTR9, DNMT1, NSD1, OGT, SMARCB1, SUPT6H, WDR61] | |
|  | **GO:0034968 histone lysine methylation** | 2.0E-3 | | 12.0E-3 | 520.0E-9 | 1.6E-6 | [6, 7, 8, 9, 10] | 24.07 | | 13.00 | [BCOR, BRCA1, CHD6, CTR9, DNMT1, DPY30, EHMT2, NSD1, OGT, SETD2, SMARCB1, SUPT6H, WDR61] | |
|  | **GO:0031061 negative regulation of histone methylation** | 870.0E-6 | | 7.2E-3 | 520.0E-9 | 1.6E-6 | [6, 7, 8, 9, 10, 11] | 55.56 | | 5.00 | [BCOR, BRCA1, DNMT1, SMARCB1, SUPT6H] | |
|  | **GO:0016573 histone acetylation** | 200.0E-6 | | 2.3E-3 | 520.0E-9 | 1.6E-6 | [7, 8, 10, 11] | 22.68 | | 22.00 | [BRCA1, BRD7, BRD8, CTBP1, DMAP1, DR1, HCFC1, ING3, MEAF6, NOC2L, OGT, POLE3, RUVBL1, RUVBL2, SAP130, SIN3A, SMARCB1, TAF6L, TAF9, TRRAP, YEATS2, YEATS4] | |
|  | **GO:0035065 regulation of histone acetylation** | 11.0E-3 | | 46.0E-3 | 520.0E-9 | 1.6E-6 | [6, 7, 8, 9, 10, 11, 12] | 26.92 | | 7.00 | [BRCA1, BRD7, CTBP1, NOC2L, RUVBL2, SIN3A, SMARCB1] | |
|  | GO:0006265 DNA topological change | 8.4E-3 | | 37.0E-3 | 8.4E-3 | 9.4E-3 | [5, 6, 7] | 60.00 | | 3.00 | [ERCC3, TOP1, TOP2A] | |
|  | GO:0044281 small molecule metabolic process | 860.0E-6 | | 7.2E-3 | 43.0E-12 | 540.0E-12 | [3] | 13.69 | | 99.00 | [ABCD3, ACAA1, ACADM, ACLY, ACO2, ACOT1, ACOT2, ACSF3, ADCK3, AK1, AK3, ALDH3A2, ALDH5A1, ALDH6A1, ALDOA, AMACR, ASNS, ATP1B1, ATP2B4, ATP5B, ATP5O, BCKDHA, BRCA1, CAD, CARS, CBR1, COA6, COQ3, CROT, DDAH1, DECR2, DHCR7, DHRS4, DPM1, ETFA, ETFDH, FAAH, FARSB, GALK1, GARS, GATB, GATC, GBA, GCDH, GK, GLB1, GNAI3, GOT1, GPT2, GUK1, HARS2, HOGA1, HSPA8, KARS, LONP2, LTA4H, MARC1, MARS2, MCAT, ME1, ME2, MGST2, MLYCD, MTHFD1, MTHFD1L, MTHFS, MUT, NADK2, NDUFS1, OGT, OLA1, OXCT1, PARK7, PDHA1, PDHB, PECR, PFKFB2, PFKP, PGD, PMVK, PNPLA8, POLR2D, PRDX5, PTRH2, PYCR1, PYCR2, QARS, QRSL1, QTRT1, REST, SCD, SLC25A17, SLC27A2, SMPDL3A, SORD, SURF1, TECR, THEM4, VCP] | |
|  | GO:0044712 single-organism catabolic process | 120.0E-6 | | 1.6E-3 | 43.0E-12 | 540.0E-12 | [3] | 16.94 | | 52.00 | [ABCD3, ABHD12, ABHD16A, ABHD2, ABHD6, ACAA1, ACADM, ACSF3, ALDH5A1, ALDH6A1, ALDOA, AMFR, ATP2B4, BAG6, BCAP31, BCKDHA, BLVRB, CROT, DERL1, ETFA, ETFDH, FAAH, GBA, GCDH, GOT1, HMOX1, HOGA1, KIAA0368, LONP2, MAN1B1, MTHFS, NAGA, NPLOC4, OGT, OS9, PFKFB2, PFKP, PNPLA2, PNPLA8, PPT1, SLC25A17, SLC27A2, SMPDL3A, SORD, STBD1, TMUB1, TRIM25, UBAC2, UBXN1, VCP, VIMP, WFS1] | |
|  | GO:0055114 oxidation-reduction process | 60.0E-6 | | 900.0E-6 | 43.0E-12 | 540.0E-12 | [3] | 19.58 | | 37.00 | [ABCD3, ACAA1, ACADM, ACO2, ALDH3A2, ALKBH5, BLVRA, CBR1, COA6, CROT, DHRS4, ETFA, ETFDH, GCDH, GRHPR, GYS1, HMOX1, LONP2, MARC1, MGST1, MLYCD, NDUFS1, PARK7, PDHA1, PDHB, PECR, PGD, PPOX, PRDX5, PTRH2, SLC25A12, SLC25A17, SLC27A2, SQRDL, STBD1, TRAP1, VCP] | |
|  | GO:1901564 organonitrogen compound metabolic process | 160.0E-9 | | 8.8E-6 | 43.0E-12 | 540.0E-12 | [3] | 15.88 | | 115.00 | [ABCB6, ACADM, ADAR, AK3, ALDH5A1, ALDH6A1, ALDOA, AP2A1, APEH, ASNS, ATP1B1, ATP2B4, ATP5B, ATP5O, B4GAT1, BCKDHA, BLVRB, CAD, CARS, COA3, COA6, CRAT, CTSH, DDAH1, DNAJC3, EEF2, EGFR, EIF1, EIF2A, EIF2B4, EIF2S3, EIF3A, EIF3D, EIF3F, EIF3G, EIF3K, EIF4G2, EIF5, EIF5B, EIF6, ERBB2, ETHE1, FARSB, FXR1, GATB, GATC, GBA, GFM1, GFM2, GNAI3, GOT1, GPT2, GUK1, HAGH, HARS2, HMOX1, HOGA1, HSPA8, IDE, KARS, LARP1, LARP4B, LTA4H, MAP2K2, MARS2, ME1, ME2, MGST2, MRPL41, MRPL44, MTG1, MTHFD1, MTHFD1L, MTHFD2, MTHFS, MUT, NADK2, NCBP1, NDUFS1, NPM1, OGT, OLA1, ORMDL1, ORMDL2, PARK7, PFKFB2, PFKP, PGD, PICALM, POLDIP3, PPOX, PRDX5, PTCD3, PTRH2, PYCR1, PYCR2, QARS, QRSL1, QTRT1, RPS27L, RPS3, RPS4X, SLC27A1, SLC38A2, SMPDL3A, SNIP1, SRM, STAT3, SURF1, TPP1, TPR, TRAP1, TUFM, VCP, YTHDF2] | |
|  | GO:0006082 organic acid metabolic process | 20.0E-6 | | 410.0E-6 | 43.0E-12 | 540.0E-12 | [3, 4] | 17.29 | | 60.00 | [ABCD3, ACAA1, ACADM, ACLY, ACO2, ACOT1, ACOT2, ACSF3, ALDH5A1, ALDH6A1, ALDOA, AMACR, ASNS, ATP2B4, BCKDHA, BRCA1, CARS, CROT, DDAH1, DECR2, ETFA, ETFDH, FAAH, FARSB, GATB, GATC, GCDH, GOT1, GPT2, HARS2, HOGA1, KARS, LONP2, LTA4H, MARC1, MARS2, MCAT, ME1, MGST2, MLYCD, MTHFD1, MTHFD1L, MTHFS, MUT, OGT, PARK7, PDHA1, PDHB, PFKFB2, PFKP, PNPLA8, PYCR1, PYCR2, QARS, QRSL1, SCD, SLC25A17, SLC27A2, TECR, THEM4] | |
|  | GO:0016042 lipid catabolic process | 1.3E-3 | | 9.4E-3 | 43.0E-12 | 540.0E-12 | [4] | 20.39 | | 21.00 | [ABCD3, ABHD12, ABHD16A, ABHD2, ABHD6, ACAA1, ACADM, CROT, ETFA, ETFDH, FAAH, GBA, GCDH, LONP2, NAGA, PNPLA2, PNPLA8, PPT1, SLC25A17, SLC27A2, SMPDL3A] | |
|  | GO:0044282 small molecule catabolic process | 4.7E-3 | | 25.0E-3 | 43.0E-12 | 540.0E-12 | [4] | 18.87 | | 20.00 | [ABCD3, ACAA1, ACADM, ACSF3, ALDH5A1, ALDH6A1, ATP2B4, BCKDHA, CROT, ETFA, ETFDH, FAAH, GCDH, GOT1, HOGA1, LONP2, MTHFS, SLC25A17, SLC27A2, SORD] | |
|  | GO:0043436 oxoacid metabolic process | 3.6E-6 | | 100.0E-6 | 43.0E-12 | 540.0E-12 | [4, 5] | 18.51 | | 57.00 | [ABCD3, ACAA1, ACADM, ACLY, ACO2, ACOT1, ACOT2, ACSF3, ALDH5A1, ALDH6A1, ALDOA, AMACR, ASNS, ATP2B4, BRCA1, CARS, CROT, DECR2, ETFA, ETFDH, FAAH, FARSB, GATB, GATC, GCDH, GOT1, GPT2, HARS2, HOGA1, KARS, LONP2, LTA4H, MARC1, MARS2, MCAT, ME1, MGST2, MLYCD, MTHFD1, MTHFD1L, MTHFS, OGT, PARK7, PDHA1, PDHB, PFKFB2, PFKP, PNPLA8, PYCR1, PYCR2, QARS, QRSL1, SCD, SLC25A17, SLC27A2, TECR, THEM4] | |
|  | GO:0006520 cellular amino acid metabolic process | 780.0E-6 | | 7.0E-3 | 43.0E-12 | 540.0E-12 | [3, 4, 6, 7] | 23.08 | | 18.00 | [ALDH5A1, ALDH6A1, ASNS, ATP2B4, CARS, FARSB, GATB, GATC, GOT1, GPT2, HARS2, KARS, MARS2, MTHFD1, PYCR1, PYCR2, QARS, QRSL1] | |
|  | GO:0006631 fatty acid metabolic process | 9.4E-3 | | 40.0E-3 | 43.0E-12 | 540.0E-12 | [4, 5, 7, 8] | 17.14 | | 24.00 | [ABCD3, ACAA1, ACADM, ACOT1, ACOT2, ACSF3, BRCA1, CROT, DECR2, ETFA, ETFDH, FAAH, GCDH, LONP2, LTA4H, MCAT, MGST2, MLYCD, PNPLA8, SCD, SLC25A17, SLC27A2, TECR, THEM4] | |
|  | GO:0046395 carboxylic acid catabolic process | 1.5E-3 | | 10.0E-3 | 43.0E-12 | 540.0E-12 | [5, 6, 7] | 22.08 | | 17.00 | [ABCD3, ACAA1, ACADM, ACSF3, ALDH5A1, ALDH6A1, ATP2B4, CROT, ETFA, ETFDH, FAAH, GCDH, GOT1, HOGA1, LONP2, SLC25A17, SLC27A2] | |
|  | GO:0032787 monocarboxylic acid metabolic process | 3.2E-3 | | 18.0E-3 | 43.0E-12 | 540.0E-12 | [6, 7] | 16.35 | | 34.00 | [ABCD3, ACAA1, ACADM, ACOT1, ACOT2, ACSF3, ALDOA, AMACR, BRCA1, CROT, DECR2, ETFA, ETFDH, FAAH, GCDH, HOGA1, LONP2, LTA4H, MCAT, MGST2, MLYCD, MTHFD1L, OGT, PARK7, PDHA1, PDHB, PFKFB2, PFKP, PNPLA8, SCD, SLC25A17, SLC27A2, TECR, THEM4] | |
|  | GO:0019395 fatty acid oxidation | 870.0E-6 | | 7.2E-3 | 43.0E-12 | 540.0E-12 | [5, 6, 7, 8, 9] | 28.95 | | 11.00 | [ABCD3, ACAA1, ACADM, CROT, ETFA, ETFDH, GCDH, LONP2, MLYCD, SLC25A17, SLC27A2] | |
|  | GO:0072329 monocarboxylic acid catabolic process | 350.0E-6 | | 3.7E-3 | 43.0E-12 | 540.0E-12 | [6, 7, 8] | 30.00 | | 12.00 | [ABCD3, ACAA1, ACADM, CROT, ETFA, ETFDH, FAAH, GCDH, HOGA1, LONP2, SLC25A17, SLC27A2] | |
|  | GO:0033539 fatty acid beta-oxidation using acyl-CoA dehydrogenase | 2.6E-3 | | 16.0E-3 | 43.0E-12 | 540.0E-12 | [7, 8, 9, 10, 11] | 57.14 | | 4.00 | [ACADM, ETFA, ETFDH, GCDH] | |
|  | GO:0044712 single-organism catabolic process | 120.0E-6 | | 1.6E-3 | 120.0E-6 | 240.0E-6 | [3] | 16.94 | | 52.00 | [ABCD3, ABHD12, ABHD16A, ABHD2, ABHD6, ACAA1, ACADM, ACSF3, ALDH5A1, ALDH6A1, ALDOA, AMFR, ATP2B4, BAG6, BCAP31, BCKDHA, BLVRB, CROT, DERL1, ETFA, ETFDH, FAAH, GBA, GCDH, GOT1, HMOX1, HOGA1, KIAA0368, LONP2, MAN1B1, MTHFS, NAGA, NPLOC4, OGT, OS9, PFKFB2, PFKP, PNPLA2, PNPLA8, PPT1, SLC25A17, SLC27A2, SMPDL3A, SORD, STBD1, TMUB1, TRIM25, UBAC2, UBXN1, VCP, VIMP, WFS1] | |
|  | GO:0016042 lipid catabolic process | 1.3E-3 | | 9.4E-3 | 120.0E-6 | 240.0E-6 | [4] | 20.39 | | 21.00 | [ABCD3, ABHD12, ABHD16A, ABHD2, ABHD6, ACAA1, ACADM, CROT, ETFA, ETFDH, FAAH, GBA, GCDH, LONP2, NAGA, PNPLA2, PNPLA8, PPT1, SLC25A17, SLC27A2, SMPDL3A] | |
|  | GO:0044282 small molecule catabolic process | 4.7E-3 | | 25.0E-3 | 120.0E-6 | 240.0E-6 | [4] | 18.87 | | 20.00 | [ABCD3, ACAA1, ACADM, ACSF3, ALDH5A1, ALDH6A1, ATP2B4, BCKDHA, CROT, ETFA, ETFDH, FAAH, GCDH, GOT1, HOGA1, LONP2, MTHFS, SLC25A17, SLC27A2, SORD] | |
|  | GO:0036503 ERAD pathway | 1.0E-3 | | 8.3E-3 | 120.0E-6 | 240.0E-6 | [3, 4, 5, 6] | 23.81 | | 15.00 | [AMFR, BAG6, BCAP31, DERL1, KIAA0368, MAN1B1, NPLOC4, OS9, TMUB1, TRIM25, UBAC2, UBXN1, VCP, VIMP, WFS1] | |
|  | GO:0046395 carboxylic acid catabolic process | 1.5E-3 | | 10.0E-3 | 120.0E-6 | 240.0E-6 | [5, 6, 7] | 22.08 | | 17.00 | [ABCD3, ACAA1, ACADM, ACSF3, ALDH5A1, ALDH6A1, ATP2B4, CROT, ETFA, ETFDH, FAAH, GCDH, GOT1, HOGA1, LONP2, SLC25A17, SLC27A2] | |
|  | GO:0006284 base-excision repair | 11.0E-3 | | 46.0E-3 | 11.0E-3 | 11.0E-3 | [6, 7, 8] | 25.00 | | 8.00 | [HUWE1, LIG3, POLG2, POLR1B, RPA2, RPS3, UNG, USP47] | |
|  | GO:0006999 nuclear pore organization | 4.2E-3 | | 22.0E-3 | 2.1E-3 | 3.0E-3 | [5] | 41.67 | | 5.00 | [NDC1, NUP133, NUP153, RTN4, TPR] | |
|  | GO:0046931 pore complex assembly | 6.3E-3 | | 30.0E-3 | 2.1E-3 | 3.0E-3 | [5, 6] | 38.46 | | 5.00 | [BAD, NDC1, NUP153, RTN4, TPR] | |
|  | GO:0010256 endomembrane system organization | 60.0E-6 | | 910.0E-6 | 180.0E-6 | 340.0E-6 | [3] | 17.41 | | 51.00 | [ANKLE2, ASUN, ATL2, ATP1B1, ATP1B3, BAG6, BNIP1, CDH1, COG2, COG3, COG4, DLG1, DNM2, EZR, FLOT2, GBF1, GCC2, GOLGA5, GOLPH3L, HOOK1, JUP, LEMD2, LMAN1, MACF1, MYADM, MYO18A, PKP2, PKP3, PPFIA1, PPIL2, PRKD1, PRMT5, RAB10, RAB11A, RAB18, RAB2A, RAB3GAP2, RTN4, SEC61A1, SNX3, STX18, STX3, STX8, STXBP1, SYNE1, TMBIM1, TMED5, VRK1, VTI1A, VTI1B, ZW10] | |
|  | GO:0007030 Golgi organization | 18.0E-6 | | 400.0E-6 | 180.0E-6 | 340.0E-6 | [4] | 28.36 | | 19.00 | [ATL2, COG2, COG3, COG4, GBF1, GCC2, GOLGA5, GOLPH3L, LMAN1, MYO18A, PRKD1, PRMT5, RAB2A, STX18, SYNE1, TMED5, VRK1, VTI1A, ZW10] | |
|  | GO:0048193 Golgi vesicle transport | 7.8E-3 | | 35.0E-3 | 180.0E-6 | 340.0E-6 | [5] | 18.27 | | 19.00 | [ARF3, ATL2, BET1L, COG2, COG3, COG4, EPS15, GBF1, GCC2, GOLGA5, LAMP1, LMAN1, MYO18A, RAB2A, RP2, STEAP2, STX18, TMEM115, ZW10] | |
|  | GO:0090161 Golgi ribbon formation | 2.6E-3 | | 16.0E-3 | 180.0E-6 | 340.0E-6 | [5] | 45.45 | | 5.00 | [GCC2, MYO18A, PRMT5, TMED5, VTI1A] | |
|  | GO:0006890 retrograde vesicle-mediated transport, Golgi to ER | 11.0E-3 | | 45.0E-3 | 180.0E-6 | 340.0E-6 | [6] | 30.00 | | 6.00 | [ARF3, BET1L, COG3, COG4, GBF1, TMEM115] | |
|  | GO:0009653 anatomical structure morphogenesis | 33.0E-6 | | 590.0E-6 | 840.0E-12 | 6.0E-9 | [2, 3] | 6.22 | | 56.00 | [ABCC4, ABL1, ACTN1, ACTN4, ACTR2, ACTR3, ALDOA, ARPIN, ATP5B, ATP6V1D, BCOR, BRCA1, CEP131, CFAP20, DLG1, DNM1L, EFNA5, EZR, FITM2, FN1, HOXB13, HTATIP2, ITGA2, LAMA3, MACF1, MAP4, MAPT, MARK2, MPP5, MYADM, MYDGF, MYH14, NEDD4L, NUBPL, PCM1, PDZD8, PHIP, PKN1, PKP2, PRKD1, PTPN11, RAC1, RAC3, RHOA, RNMT, RPL10, RTN4, SCRIB, SDCBP, SGSM3, SUPV3L1, SYNE3, SYNJ2BP, TRIOBP, WARS, ZMYM4] | |
|  | GO:0050793 regulation of developmental process | 3.9E-6 | | 100.0E-6 | 840.0E-12 | 6.0E-9 | [2, 3] | 5.73 | | 49.00 | [ABL1, ACTN4, ALDOA, ARPIN, BCOR, BRCA1, CDC73, CTR9, DLG1, DNM1L, EFNA5, EPHA3, EZR, FITM2, FLOT2, HMGB2, HTATIP2, MACF1, MAFB, MAP4, MAPT, MARK2, MYADM, MYDGF, NEDD4L, OGT, PDZD8, PHIP, PKP2, PNPT1, PRKD1, PRMT1, PTRH2, RAC1, RAC3, REST, RHOA, RPL10, RTN4, SDCBP, SGSM3, SKI, SPEN, SYNE3, SYNJ2BP, TRIOBP, WARS, WDR61, ZMYM4] | |
|  | GO:0048646 anatomical structure formation involved in morphogenesis | 56.0E-6 | | 870.0E-6 | 840.0E-12 | 6.0E-9 | [2, 3, 4] | 4.74 | | 21.00 | [ABCC4, ACTN1, ACTR2, ATP5B, ATP6V1D, BRCA1, FN1, HOXB13, HTATIP2, ITGA2, LAMA3, MAP4, MYDGF, PCM1, PRKD1, RHOA, RPL10, SCRIB, SGSM3, SYNJ2BP, WARS] | |
|  | GO:0072358 cardiovascular system development | 200.0E-6 | | 2.3E-3 | 840.0E-12 | 6.0E-9 | [4, 5, 6] | 4.31 | | 14.00 | [ATP5B, BCOR, BRCA1, DSG2, HOXB13, HTATIP2, MYDGF, PKP2, PRKD1, PTPN11, RHOA, RPL10, SYNJ2BP, WARS] | |
|  | GO:0009653 anatomical structure morphogenesis | 33.0E-6 | | 590.0E-6 | 46.0E-12 | 460.0E-12 | [2, 3] | 6.22 | | 56.00 | [ABCC4, ABL1, ACTN1, ACTN4, ACTR2, ACTR3, ALDOA, ARPIN, ATP5B, ATP6V1D, BCOR, BRCA1, CEP131, CFAP20, DLG1, DNM1L, EFNA5, EZR, FITM2, FN1, HOXB13, HTATIP2, ITGA2, LAMA3, MACF1, MAP4, MAPT, MARK2, MPP5, MYADM, MYDGF, MYH14, NEDD4L, NUBPL, PCM1, PDZD8, PHIP, PKN1, PKP2, PRKD1, PTPN11, RAC1, RAC3, RHOA, RNMT, RPL10, RTN4, SCRIB, SDCBP, SGSM3, SUPV3L1, SYNE3, SYNJ2BP, TRIOBP, WARS, ZMYM4] | |
|  | GO:0050793 regulation of developmental process | 3.9E-6 | | 100.0E-6 | 46.0E-12 | 460.0E-12 | [2, 3] | 5.73 | | 49.00 | [ABL1, ACTN4, ALDOA, ARPIN, BCOR, BRCA1, CDC73, CTR9, DLG1, DNM1L, EFNA5, EPHA3, EZR, FITM2, FLOT2, HMGB2, HTATIP2, MACF1, MAFB, MAP4, MAPT, MARK2, MYADM, MYDGF, NEDD4L, OGT, PDZD8, PHIP, PKP2, PNPT1, PRKD1, PRMT1, PTRH2, RAC1, RAC3, REST, RHOA, RPL10, RTN4, SDCBP, SGSM3, SKI, SPEN, SYNE3, SYNJ2BP, TRIOBP, WARS, WDR61, ZMYM4] | |
|  | GO:0051239 regulation of multicellular organismal process | 4.4E-9 | | 400.0E-9 | 46.0E-12 | 460.0E-12 | [2, 3] | 5.20 | | 54.00 | [ABL1, ATP2A2, ATP2B4, BCOR, BRCA1, CD276, CDC73, CHID1, CTR9, DSG2, DSP, EPB41L4B, EPHA3, EZR, FN1, GBA, HMGB2, HTATIP2, JUP, MACF1, MAFB, MAPT, MARK2, MTG1, MTPN, MYADM, MYDGF, NDUFA2, NEDD4L, OGT, PARK7, PFN1, PKP2, PRDX5, PRKD1, PRMT1, PTRH2, RAB11A, RAC1, REST, RHOA, RNMT, RPSA, RTN4, SDCBP, SKI, SLC9A1, SPEN, SYNJ2BP, TRIM27, VAMP7, WARS, WDR61, ZC3HAV1] | |
|  | GO:0007275 multicellular organism development | 720.0E-9 | | 26.0E-6 | 46.0E-12 | 460.0E-12 | [3] | 6.32 | | 81.00 | [ABCB6, ABL1, ACTN1, ALDH3A2, ALDH5A1, ANKLE2, ATP2B4, ATP5B, ATP5F1, ATXN10, BCOR, BRCA1, CBX2, CDC73, CENPF, CKB, COPS2, CTR9, DDX1, DSG2, DSP, EIF2B4, EPHA3, EPHA5, ERCC3, EZR, FARP2, FLVCR1, FN1, GNB4, HMGB2, HOXB13, HTATIP2, IDE, ITGA2, JAGN1, LAMA3, LEMD2, LIG3, MACF1, MAFB, MAPT, MARK2, MYADM, MYDGF, MYH14, NCOA6, NDUFS3, NEDD4L, OGT, PIR, PKN1, PKP2, PLS3, POLR2D, PPT1, PRKD1, PRMT1, PTPN11, PTRH2, QARS, RAB11A, REST, RHOA, RNMT, RPL10, RPS4X, RTN4, SCRIB, SDCBP, SKI, SPEN, SYNJ2BP, TOR1A, TP53BP1, TPP1, WARS, WDR61, WFS1, YWHAE, ZNF148] | |
|  | GO:0051094 positive regulation of developmental process | 19.0E-6 | | 410.0E-6 | 46.0E-12 | 460.0E-12 | [2, 3, 4] | 4.49 | | 20.00 | [BRCA1, DNM1L, EPHA3, HMGB2, MACF1, MAPT, MARK2, MYADM, MYDGF, NEDD4L, OGT, PRKD1, PRMT1, PTRH2, RAC1, RAC3, RHOA, SDCBP, SPEN, TRIOBP] | |
|  | GO:0051240 positive regulation of multicellular organismal process | 510.0E-9 | | 21.0E-6 | 46.0E-12 | 460.0E-12 | [2, 3, 4] | 4.58 | | 28.00 | [BRCA1, CD276, EPB41L4B, EPHA3, HMGB2, MACF1, MAPT, MARK2, MTPN, MYDGF, NDUFA2, NEDD4L, OGT, PARK7, PFN1, PRDX5, PRKD1, PRMT1, PTRH2, RAB11A, RHOA, RNMT, RPSA, SDCBP, SLC9A1, SPEN, VAMP7, ZC3HAV1] | |
|  | GO:0051241 negative regulation of multicellular organismal process | 250.0E-6 | | 2.7E-3 | 46.0E-12 | 460.0E-12 | [2, 3, 4] | 4.88 | | 19.00 | [ATP2B4, BCOR, CDC73, CHID1, CTR9, EZR, FN1, GBA, MAFB, PRMT1, RAC1, REST, RHOA, RPSA, RTN4, SKI, SYNJ2BP, TRIM27, WDR61] | |
|  | GO:0048731 system development | 99.0E-9 | | 6.5E-6 | 46.0E-12 | 460.0E-12 | [3, 4] | 5.91 | | 70.00 | [ABCB6, ABL1, ACTN1, ALDH3A2, ALDH5A1, ANKLE2, ATP2B4, ATP5B, ATP5F1, ATXN10, BCOR, BRCA1, CDC73, CENPF, CKB, COPS2, CTR9, DSG2, DSP, EIF2B4, EPHA3, EPHA5, ERCC3, EZR, FARP2, FLVCR1, GNB4, HMGB2, HOXB13, HTATIP2, JAGN1, LEMD2, LIG3, MACF1, MAFB, MAPT, MARK2, MYDGF, MYH14, NCOA6, NDUFS3, NEDD4L, OGT, PIR, PKP2, PLS3, POLR2D, PPT1, PRKD1, PRMT1, PTPN11, PTRH2, QARS, RAB11A, REST, RHOA, RPL10, RTN4, SCRIB, SDCBP, SKI, SPEN, SYNJ2BP, TOR1A, TPP1, WARS, WDR61, WFS1, YWHAE, ZNF148] | |
|  | GO:2000026 regulation of multicellular organismal development | 14.0E-6 | | 320.0E-6 | 46.0E-12 | 460.0E-12 | [3, 4] | 4.99 | | 28.00 | [ABL1, BCOR, BRCA1, CDC73, CTR9, EPHA3, HMGB2, HTATIP2, MACF1, MAFB, MAPT, MARK2, MYADM, MYDGF, NEDD4L, OGT, PRKD1, PRMT1, PTRH2, REST, RHOA, RTN4, SDCBP, SKI, SPEN, SYNJ2BP, WARS, WDR61] | |
|  | GO:0045595 regulation of cell differentiation | 610.0E-6 | | 5.7E-3 | 46.0E-12 | 460.0E-12 | [3, 4, 5] | 5.77 | | 30.00 | [ABL1, ACTN4, CDC73, CTR9, EFNA5, EPHA3, FLOT2, HMGB2, MACF1, MAFB, MAPT, MARK2, MYADM, MYDGF, NEDD4L, OGT, PKP2, PRKD1, PRMT1, RAC1, RAC3, REST, RHOA, RPL10, RTN4, SDCBP, SKI, SPEN, TRIOBP, WDR61] | |
|  | GO:0006952 defense response | 680.0E-6 | | 6.3E-3 | 14.0E-6 | 31.0E-6 | [3] | 5.87 | | 31.00 | [ALKBH5, ANXA5, ATP1B1, BRD4, CDC37, CHID1, DNAJA3, ELMOD2, ERBB2IP, FAU, FLOT2, HMGB2, HMOX1, KLK3, LAMP1, LYST, MDH1, MLF2, MRPS10, PEX13, PHB, PTPN2, RPSA, SIN3A, SNRPB, TRIM25, TRIM27, VAMP7, VIMP, ZC3HAV1, ZCCHC17] | |
|  | GO:0051707 response to other organism | 6.3E-3 | | 30.0E-3 | 14.0E-6 | 31.0E-6 | [2, 4] | 6.25 | | 27.00 | [ADAR, ALKBH5, ANXA5, ATP1B1, BAIAP2, CCT5, EEF1G, ELMOD2, ENO1, ERBB2IP, FAU, HMGB2, KLK3, MDH1, MRPS10, NDUFA2, PEX13, PSMA2, RPS15A, RPSA, SIN3A, SLC25A21, SNRPB, SNX3, VIMP, ZC3HAV1, ZCCHC17] | |
|  | GO:0032101 regulation of response to external stimulus | 5.9E-3 | | 28.0E-3 | 14.0E-6 | 31.0E-6 | [3, 4] | 5.88 | | 22.00 | [ALKBH5, ANXA5, ATP1B1, BRD4, CHID1, ELMOD2, MDH1, MRPS10, PEX13, PHB, PRKD1, RAC1, RAC2, RNMT, RPL10, SIN3A, SNRPB, STX3, VAMP7, VIMP, ZC3HAV1, ZCCHC17] | |
|  | GO:0009617 response to bacterium | 10.0E-3 | | 42.0E-3 | 14.0E-6 | 31.0E-6 | [3, 5] | 4.64 | | 9.00 | [BAIAP2, ERBB2IP, FAU, HMGB2, KLK3, NDUFA2, RPSA, SNX3, VIMP] | |
|  | GO:0098542 defense response to other organism | 4.7E-3 | | 24.0E-3 | 14.0E-6 | 31.0E-6 | [3, 4, 5] | 5.24 | | 15.00 | [ALKBH5, ANXA5, ATP1B1, ELMOD2, FAU, HMGB2, KLK3, MDH1, MRPS10, PEX13, RPSA, SIN3A, SNRPB, ZC3HAV1, ZCCHC17] | |
|  | GO:0045333 cellular respiration | 1.1E-3 | | 8.5E-3 | 2.1E-6 | 5.7E-6 | [4, 5] | 32.14 | | 9.00 | [ACO2, COA6, NDUFS1, PARK7, PDHA1, PDHB, SLC25A12, TRAP1, VCP] | |
|  | GO:0009060 aerobic respiration | 8.2E-3 | | 36.0E-3 | 2.1E-6 | 5.7E-6 | [5, 6] | 44.44 | | 4.00 | [ACO2, PDHA1, PDHB, VCP] | |
|  | GO:0009141 nucleoside triphosphate metabolic process | 850.0E-6 | | 7.3E-3 | 2.1E-6 | 5.7E-6 | [5, 6, 7] | 22.78 | | 18.00 | [AK1, AK3, ALDOA, ATP1B1, ATP5B, ATP5O, COA6, GNAI3, HSPA8, NDUFS1, OGT, OLA1, PARK7, PFKFB2, PFKP, SMPDL3A, SURF1, VCP] | |
|  | GO:0006085 acetyl-CoA biosynthetic process | 8.2E-3 | | 36.0E-3 | 2.1E-6 | 5.7E-6 | [6, 7] | 44.44 | | 4.00 | [ACLY, MLYCD, PDHA1, PDHB] | |
|  | GO:0019395 fatty acid oxidation | 870.0E-6 | | 7.2E-3 | 2.1E-6 | 5.7E-6 | [5, 6, 7, 8, 9] | 28.95 | | 11.00 | [ABCD3, ACAA1, ACADM, CROT, ETFA, ETFDH, GCDH, LONP2, MLYCD, SLC25A17, SLC27A2] | |
|  | GO:0009167 purine ribonucleoside monophosphate metabolic process | 3.8E-3 | | 20.0E-3 | 2.1E-6 | 5.7E-6 | [7, 8, 9] | 21.43 | | 15.00 | [AK3, ALDOA, ATP1B1, ATP5B, ATP5O, COA6, HSPA8, NDUFS1, OGT, OLA1, PARK7, PFKFB2, PFKP, SURF1, VCP] | |
|  | GO:0009205 purine ribonucleoside triphosphate metabolic process | 490.0E-6 | | 4.7E-3 | 2.1E-6 | 5.7E-6 | [7, 8, 9] | 24.62 | | 16.00 | [AK3, ALDOA, ATP1B1, ATP5B, ATP5O, COA6, GNAI3, HSPA8, NDUFS1, OGT, OLA1, PARK7, PFKFB2, PFKP, SURF1, VCP] | |
|  | GO:0070584 mitochondrion morphogenesis | 8.2E-3 | | 36.0E-3 | 8.2E-3 | 9.3E-3 | [4, 5, 6] | 44.44 | | 4.00 | [DNM1L, MYH14, NUBPL, SUPV3L1] | |
|  | GO:0000380 alternative mRNA splicing, via spliceosome | 11.0E-3 | | 46.0E-3 | 8.8E-12 | 140.0E-12 | [8, 9, 10, 11, 12] | 25.00 | | 8.00 | [HNRNPM, NSRP1, PQBP1, RBM15B, RBM25, RBM5, RNPS1, TRA2B] | |
|  | GO:0071826 ribonucleoprotein complex subunit organization | 150.0E-6 | | 1.8E-3 | 8.8E-12 | 140.0E-12 | [4] | 22.55 | | 23.00 | [ADAR, ATXN2, ATXN2L, DDX6, DHX30, EIF2A, EIF3A, EIF6, FASTKD2, GEMIN5, GFM2, MMAB, MRRF, PQBP1, PRMT5, PRPF19, PRPF8, RBM5, SNRPB, SNRPC, TAF9, TFIP11, YTHDC1] | |
|  | GO:0022618 ribonucleoprotein complex assembly | 1.7E-3 | | 11.0E-3 | 8.8E-12 | 140.0E-12 | [4, 5, 6] | 20.41 | | 20.00 | [ADAR, ATXN2, ATXN2L, DDX6, DHX30, EIF2A, EIF3A, EIF6, FASTKD2, GEMIN5, MMAB, PQBP1, PRMT5, PRPF19, PRPF8, RBM5, SNRPB, SNRPC, TAF9, YTHDC1] | |
|  | GO:0006396 RNA processing | 4.2E-9 | | 440.0E-9 | 8.8E-12 | 140.0E-12 | [5, 6, 7] | 21.21 | | 63.00 | [ADAR, AHCYL1, ALKBH5, C14orf166, CCNT1, CDC73, CDK11A, CDK11B, CMTR1, CPSF3, CPSF3L, CTR9, DDX1, DDX27, DDX41, DDX54, EGFR, EXOSC10, GEMIN5, HNRNPM, INTS1, INTS2, INTS4, INTS5, INTS8, MAP2K2, NCBP1, NOL11, NOL9, NSRP1, NSUN2, NUDT21, PNPT1, POLR2A, PQBP1, PRMT5, PRPF19, PRPF8, RBM15B, RBM25, RBM5, RNF40, RNMT, RNPS1, RPL7, RPS28, RPS6, RPS7, RTCB, SNIP1, SNRNP200, SNRPB, SNRPC, SUPT5H, SUPV3L1, SYMPK, TFIP11, TRA2B, TRMT10C, TRMU, U2AF2, UTP20, YTHDC1] | |
|  | GO:0016071 mRNA metabolic process | 140.0E-6 | | 1.7E-3 | 8.8E-12 | 140.0E-12 | [5, 6, 7] | 18.57 | | 39.00 | [AHCYL1, ALKBH5, CCNT1, CDC73, CDK11A, CDK11B, CMTR1, CPSF3, CTR9, DDX41, EXOSC10, GEMIN5, HNRNPM, NCBP1, NSRP1, NUDT21, PARN, PNPT1, PQBP1, PRMT5, PRPF19, PRPF8, RBM15B, RBM25, RBM5, RNF40, RNMT, RNPS1, SNRNP200, SNRPB, SNRPC, SUPT5H, SUPV3L1, SYMPK, TFIP11, TRA2B, U2AF2, YTHDC1, ZC3HAV1] | |
|  | GO:0034660 ncRNA metabolic process | 13.0E-6 | | 320.0E-6 | 8.8E-12 | 140.0E-12 | [5, 6, 7] | 21.71 | | 33.00 | [ADAR, C14orf166, CARS, CPSF3L, DDX1, DDX27, EXOSC10, FARSB, GATB, GATC, HARS2, INTS1, INTS2, INTS4, INTS5, INTS8, KARS, MARS2, MEPCE, NOL11, NOL9, NSUN2, PNPT1, QARS, QRSL1, RPL7, RPS28, RPS6, RPS7, RTCB, SMARCB1, TRMU, UTP20] | |
|  | GO:1903311 regulation of mRNA metabolic process | 240.0E-6 | | 2.7E-3 | 8.8E-12 | 140.0E-12 | [5, 6, 7, 8] | 23.75 | | 19.00 | [AHCYL1, CCNT1, CDC73, CDK11A, CDK11B, CTR9, NCBP1, NSRP1, PNPT1, RBM15B, RBM25, RBM5, RNF40, RNPS1, SUPT5H, TRA2B, U2AF2, YTHDC1, ZC3HAV1] | |
|  | GO:0006397 mRNA processing | 1.2E-6 | | 42.0E-6 | 8.8E-12 | 140.0E-12 | [6, 7, 8] | 23.33 | | 35.00 | [AHCYL1, ALKBH5, CCNT1, CDC73, CDK11A, CDK11B, CMTR1, CPSF3, CTR9, DDX41, GEMIN5, HNRNPM, NCBP1, NSRP1, NUDT21, PNPT1, PQBP1, PRMT5, PRPF19, PRPF8, RBM15B, RBM25, RBM5, RNF40, RNMT, RNPS1, SNRNP200, SNRPB, SNRPC, SUPT5H, SYMPK, TFIP11, TRA2B, U2AF2, YTHDC1] | |
|  | GO:0031123 RNA 3'-end processing | 44.0E-6 | | 730.0E-6 | 8.8E-12 | 140.0E-12 | [6, 7, 8] | 34.21 | | 13.00 | [AHCYL1, CCNT1, CDC73, CPSF3, CTR9, NCBP1, NUDT21, PNPT1, RNF40, SUPT5H, SUPV3L1, SYMPK, TRMU] | |
|  | GO:0034470 ncRNA processing | 390.0E-6 | | 3.9E-3 | 8.8E-12 | 140.0E-12 | [6, 7, 8] | 22.11 | | 21.00 | [ADAR, C14orf166, CPSF3L, DDX1, DDX27, EXOSC10, INTS1, INTS2, INTS4, INTS5, INTS8, NOL11, NOL9, NSUN2, RPL7, RPS28, RPS6, RPS7, RTCB, TRMU, UTP20] | |
|  | GO:0050684 regulation of mRNA processing | 50.0E-6 | | 800.0E-6 | 8.8E-12 | 140.0E-12 | [5, 6, 7, 8, 9] | 28.33 | | 17.00 | [AHCYL1, CCNT1, CDC73, CDK11A, CDK11B, CTR9, NCBP1, NSRP1, RBM15B, RBM25, RBM5, RNF40, RNPS1, SUPT5H, TRA2B, U2AF2, YTHDC1] | |
|  | GO:0050686 negative regulation of mRNA processing | 8.3E-3 | | 37.0E-3 | 8.8E-12 | 140.0E-12 | [5, 6, 7, 8, 9, 10] | 31.58 | | 6.00 | [CCNT1, CTR9, RNF40, RNPS1, SUPT5H, U2AF2] | |
|  | GO:0031124 mRNA 3'-end processing | 39.0E-6 | | 670.0E-6 | 8.8E-12 | 140.0E-12 | [7, 8, 9] | 39.29 | | 11.00 | [AHCYL1, CCNT1, CDC73, CPSF3, CTR9, NCBP1, NUDT21, PNPT1, RNF40, SUPT5H, SYMPK] | |
|  | GO:0031440 regulation of mRNA 3'-end processing | 480.0E-6 | | 4.7E-3 | 8.8E-12 | 140.0E-12 | [6, 7, 8, 9, 10] | 43.75 | | 7.00 | [AHCYL1, CCNT1, CDC73, CTR9, NCBP1, RNF40, SUPT5H] | |
|  | GO:0000398 mRNA splicing, via spliceosome | 7.3E-3 | | 33.0E-3 | 8.8E-12 | 140.0E-12 | [7, 8, 9, 10, 11] | 18.45 | | 19.00 | [DDX41, GEMIN5, HNRNPM, NSRP1, PQBP1, PRMT5, PRPF19, PRPF8, RBM15B, RBM25, RBM5, RNPS1, SNRNP200, SNRPB, SNRPC, TFIP11, TRA2B, U2AF2, YTHDC1] | |
|  | GO:0006518 peptide metabolic process | 420.0E-12 | | 130.0E-9 | 46.0E-9 | 210.0E-9 | [4, 5] | 22.81 | | 60.00 | [ADAR, APEH, CARS, COA3, CTSH, DNAJC3, EEF2, EGFR, EIF1, EIF2A, EIF2B4, EIF2S3, EIF3A, EIF3D, EIF3F, EIF3G, EIF3K, EIF4G2, EIF5, EIF5B, EIF6, ERBB2, ETHE1, FARSB, FXR1, GATB, GATC, GFM1, GFM2, HAGH, HARS2, IDE, KARS, LARP1, LARP4B, LTA4H, MAP2K2, MARS2, MGST2, MRPL41, MRPL44, MTG1, NCBP1, NPM1, PARK7, PICALM, POLDIP3, PTCD3, QARS, QRSL1, RPS27L, RPS3, RPS4X, SNIP1, STAT3, TPP1, TPR, TRAP1, TUFM, YTHDF2] | |
|  | GO:0034248 regulation of cellular amide metabolic process | 1.4E-3 | | 10.0E-3 | 46.0E-9 | 210.0E-9 | [4, 5] | 17.96 | | 30.00 | [ADAR, COA3, DNAJC3, EEF2, EGFR, EIF1, EIF2A, EIF4G2, EIF5, EIF5B, EIF6, ERBB2, FXR1, LARP4B, MAP2K2, MTG1, NCBP1, NPM1, ORMDL1, ORMDL2, PICALM, POLDIP3, QARS, RPS3, RPS4X, SNIP1, STAT3, TPR, TRAP1, YTHDF2] | |
|  | GO:0043604 amide biosynthetic process | 1.6E-9 | | 250.0E-9 | 46.0E-9 | 210.0E-9 | [5] | 23.11 | | 55.00 | [ADAR, CARS, COA3, DNAJC3, EEF2, EGFR, EIF1, EIF2A, EIF2B4, EIF2S3, EIF3A, EIF3D, EIF3F, EIF3G, EIF3K, EIF4G2, EIF5, EIF5B, EIF6, ERBB2, FARSB, FXR1, GATB, GATC, GBA, GFM1, GFM2, HAGH, HARS2, KARS, LARP1, LARP4B, MAP2K2, MARS2, MGST2, MRPL41, MRPL44, MTG1, NCBP1, NPM1, ORMDL1, ORMDL2, POLDIP3, PTCD3, QARS, QRSL1, RPS27L, RPS3, RPS4X, SNIP1, STAT3, TPR, TRAP1, TUFM, YTHDF2] | |
|  | GO:0010608 posttranscriptional regulation of gene expression | 7.7E-3 | | 35.0E-3 | 46.0E-9 | 210.0E-9 | [5, 6] | 16.20 | | 29.00 | [ADAR, COA3, DHX9, DKC1, DNAJC3, EEF2, EGFR, EIF1, EIF2A, EIF4G2, EIF5, EIF5B, EIF6, ERBB2, FXR1, LARP4B, MAP2K2, MTG1, NCBP1, NPM1, POLDIP3, QARS, RPS3, RPS4X, SNIP1, STAT3, TPR, TRAP1, YTHDF2] | |
|  | GO:0043043 peptide biosynthetic process | 620.0E-12 | | 140.0E-9 | 46.0E-9 | 210.0E-9 | [5, 6] | 24.41 | | 52.00 | [ADAR, CARS, COA3, DNAJC3, EEF2, EGFR, EIF1, EIF2A, EIF2B4, EIF2S3, EIF3A, EIF3D, EIF3F, EIF3G, EIF3K, EIF4G2, EIF5, EIF5B, EIF6, ERBB2, FARSB, FXR1, GATB, GATC, GFM1, GFM2, HAGH, HARS2, KARS, LARP1, LARP4B, MAP2K2, MARS2, MGST2, MRPL41, MRPL44, MTG1, NCBP1, NPM1, POLDIP3, PTCD3, QARS, QRSL1, RPS27L, RPS3, RPS4X, SNIP1, STAT3, TPR, TRAP1, TUFM, YTHDF2] | |
|  | GO:0006412 translation | 1.4E-9 | | 260.0E-9 | 46.0E-9 | 210.0E-9 | [5, 6, 7] | 24.39 | | 50.00 | [ADAR, CARS, COA3, DNAJC3, EEF2, EGFR, EIF1, EIF2A, EIF2B4, EIF2S3, EIF3A, EIF3D, EIF3F, EIF3G, EIF3K, EIF4G2, EIF5, EIF5B, EIF6, ERBB2, FARSB, FXR1, GATB, GATC, GFM1, GFM2, HARS2, KARS, LARP1, LARP4B, MAP2K2, MARS2, MRPL41, MRPL44, MTG1, NCBP1, NPM1, POLDIP3, PTCD3, QARS, QRSL1, RPS27L, RPS3, RPS4X, SNIP1, STAT3, TPR, TRAP1, TUFM, YTHDF2] | |
|  | GO:0006413 translational initiation | 12.0E-6 | | 310.0E-6 | 46.0E-9 | 210.0E-9 | [3, 6, 7, 8] | 30.00 | | 18.00 | [DNAJC3, EIF1, EIF2B4, EIF2S3, EIF3A, EIF3D, EIF3F, EIF3G, EIF3K, EIF4G2, EIF5, EIF5B, EIF6, LARP1, NCBP1, NPM1, TPR, YTHDF2] | |
|  | GO:0019081 viral translation | 1.6E-3 | | 10.0E-3 | 46.0E-9 | 210.0E-9 | [4, 5, 6, 7, 8] | 50.00 | | 5.00 | [EIF3A, EIF3D, EIF3F, EIF3G, EIF6] | |
|  | GO:0006417 regulation of translation | 2.4E-3 | | 15.0E-3 | 46.0E-9 | 210.0E-9 | [5, 6, 7, 8] | 17.76 | | 27.00 | [ADAR, COA3, DNAJC3, EEF2, EGFR, EIF1, EIF2A, EIF4G2, EIF5, EIF5B, EIF6, ERBB2, FXR1, LARP4B, MAP2K2, MTG1, NCBP1, NPM1, POLDIP3, QARS, RPS3, RPS4X, SNIP1, STAT3, TPR, TRAP1, YTHDF2] | |
|  | GO:0006446 regulation of translational initiation | 4.9E-3 | | 25.0E-3 | 46.0E-9 | 210.0E-9 | [4, 6, 7, 8, 9] | 26.47 | | 9.00 | [DNAJC3, EIF1, EIF4G2, EIF5, EIF5B, NCBP1, NPM1, TPR, YTHDF2] | |
|  | GO:0002190 cap-independent translational initiation | 12.0E-3 | | 49.0E-3 | 46.0E-9 | 210.0E-9 | [5, 8, 9, 10] | 33.33 | | 5.00 | [EIF3A, EIF3D, EIF3F, EIF6, YTHDF2] | |
|  | GO:0034097 response to cytokine | 9.8E-3 | | 41.0E-3 | 9.8E-3 | 10.0E-3 | [4] | 5.51 | | 15.00 | [ADAR, BRCA1, CDC37, DNAJA3, ERBB2IP, GBA, HTRA2, JAGN1, JAK3, PHB, PNPT1, PTPN2, STAT3, TAF9, USP10] | |
|  | GO:0034641 cellular nitrogen compound metabolic process | 67.0E-6 | | 960.0E-6 | 560.0E-9 | 1.6E-6 | [3] | 11.91 | | 311.00 | [ABCB6, ABCE1, ACADM, ACTN4, ACTR5, ADAR, AHCYL1, AK1, AK3, ALDH6A1, ALDOA, ALKBH5, APEH, ATAD2, ATF7IP, ATP1B1, ATP2B4, ATP5B, ATP5O, BAX, BCOR, BLVRB, BRCA1, BRD2, BRD4, BRD7, BUD31, C14orf166, CAD, CARS, CBX2, CCNT1, CCT2, CCT4, CCT5, CCT6A, CCT7, CCT8, CD81, CDC73, CDCA5, CDH1, CDK11A, CDK11B, CDK5RAP2, CDK5RAP3, CENPF, CHD6, CHTF8, CMTR1, COA3, COA6, CPSF3, CPSF3L, CRAT, CREB1, CTBP1, CTR9, CTSH, DDRGK1, DDX1, DDX27, DDX41, DDX54, DHX33, DKC1, DLG1, DMAP1, DNAJA3, DNAJC2, DNAJC3, DNMT1, DNPEP, EEF1D, EEF2, EGFR, EHF, EHMT2, EIF1, EIF2A, EIF2B4, EIF2S3, EIF3A, EIF3D, EIF3F, EIF3G, EIF3K, EIF4G2, EIF5, EIF5B, EIF6, ENO1, ERBB2, ERBB2IP, ERCC3, ETF1, ETHE1, EXOSC10, EZR, FARSB, FXR1, GARS, GATAD2A, GATB, GATC, GBA, GEMIN5, GFM1, GFM2, GNAI3, GNL3, GNL3L, GTF3C2, GTPBP4, GUK1, HAGH, HARS2, HCFC1, HMGB2, HMOX1, HNRNPM, HSPA8, HUWE1, IDE, ING3, INTS1, INTS2, INTS4, INTS5, INTS8, JUN, JUP, KARS, KDM1A, KMT2D, LARP1, LARP4B, LIG3, LTA4H, MAP2K2, MARS2, MBD2, MCM2, MCM7, ME1, ME2, MEPCE, MGST2, MMAB, MPHOSPH8, MRPL41, MRPL44, MTG1, MTHFS, NABP2, NACC1, NADK2, NBN, NCBP1, NCOA6, NDUFS1, NKRF, NOC2L, NOL11, NOL9, NPAT, NPM1, NR2C2, NSD1, NSRP1, NSUN2, NUDT21, NUP62, OGT, OLA1, ORMDL1, ORMDL2, PARK7, PARN, PELP1, PFDN5, PFKFB2, PFKP, PGD, PHB, PHF12, PHF2, PHF21A, PHIP, PIAS2, PICALM, PINX1, PKN1, PLK1, PNPT1, POLA1, POLDIP3, POLG2, POLR1B, POLR2A, POLR2B, POLR2D, POLR2E, POLR2G, PPIE, PPOX, PQBP1, PRDX5, PRKD1, PRMT5, PRPF19, PRPF8, PSMA6, PSMD14, PTCD3, PTRH2, PURA, QARS, QRSL1, QTRT1, RBFOX2, RBM14, RBM15B, RBM25, RBM5, RCOR1, REST, RFC2, RNF40, RNMT, RNPS1, RPA2, RPL7, RPRD1B, RPS27L, RPS28, RPS3, RPS4X, RPS6, RPS7, RTCB, RTF1, RUVBL2, SAP130, SENP1, SET, SETD2, SETSIP, SIGIRR, SIN3A, SKI, SLC33A1, SLC38A2, SLC9A1, SMARCB1, SMARCD1, SMPDL3A, SNIP1, SNRNP200, SNRPB, SNRPC, SOD2, SP3, SPEN, SPTY2D1, SRM, STAG2, STAT3, STRN3, SUPT5H, SUPT6H, SUPV3L1, SURF1, SYMPK, TAF3, TAF9, TCP1, TFAM, TFB2M, TFIP11, TOM1L1, TOP1, TOP2A, TP53BP1, TPP1, TPR, TRA2B, TRAP1, TRIM25, TRIM27, TRMT10C, TRMU, TUFM, U2AF2, UBE2T, UBE2V1, UFL1, UHRF1, UNG, USP47, UTP20, VCP, WDR61, WFS1, WRAP53, YEATS2, YTHDC1, YTHDF2, ZC3HAV1, ZNF148, ZNF746] | |
|  | GO:0044249 cellular biosynthetic process | 5.2E-3 | | 26.0E-3 | 560.0E-9 | 1.6E-6 | [3] | 11.45 | | 268.00 | [ABCB6, ABCD3, ACADM, ACLY, ACSF3, ACTN4, ADAR, ADCK3, AK1, ALDOA, ALG8, ASNS, ATAD2, ATF7IP, ATP2B4, ATP5B, ATP5O, B4GAT1, BCOR, BRCA1, BRD2, BRD4, BRD7, BUD31, C14orf166, CAD, CARS, CBX2, CCNT1, CCT2, CCT4, CCT5, CCT6A, CCT7, CCT8, CD276, CD81, CDC73, CDH1, CDK5RAP2, CDK5RAP3, CENPF, CHD6, CHTF8, COA3, COG3, COQ3, CREB1, CTBP1, CTR9, DDRGK1, DECR2, DHX33, DKC1, DLG1, DMAP1, DNAJA3, DNAJC2, DNAJC3, DNMT1, DNPEP, DPM1, EEF1D, EEF2, EGFR, EHF, EHMT2, EIF1, EIF2A, EIF2B4, EIF2S3, EIF3A, EIF3D, EIF3F, EIF3G, EIF3K, EIF4G2, EIF5, EIF5B, EIF6, ENO1, EPT1, ERBB2, ERBB2IP, ERCC3, ETF1, EZR, FARSB, FXR1, GALNT1, GALNT2, GARS, GATAD2A, GATB, GATC, GBA, GFM1, GFM2, GNL3, GNL3L, GTF3C2, GTPBP4, GYS1, HAGH, HARS2, HCFC1, HMGB2, HOGA1, HSCB, HSPA8, IDI1, ING3, JUN, JUP, KARS, KDM1A, KMT2D, LARP1, LARP4B, LIG3, LTA4H, MAP2K2, MARS2, MAT2A, MBD2, MCAT, MCM2, MCM7, MGST2, MLYCD, MMAB, MPHOSPH8, MRPL41, MRPL44, MTG1, MTHFD1, MYDGF, NABP2, NACC1, NCBP1, NCOA6, NFS1, NKRF, NOC2L, NOL11, NPAT, NPM1, NR2C2, NSD1, NUP62, OGT, ORMDL1, ORMDL2, PARK7, PDHA1, PDHB, PELP1, PFDN5, PHB, PHF12, PHF2, PHF21A, PHIP, PIAS2, PICALM, PINX1, PKN1, PLK1, PNPLA8, POLA1, POLDIP3, POLR1B, POLR2A, POLR2B, POLR2D, POLR2E, POLR2G, POMGNT1, PPIE, PPOX, PRDX5, PRKD1, PRMT5, PSMA6, PSMD2, PTCD3, PTRH2, PUM3, PURA, PYCR1, PYCR2, QARS, QRSL1, QTRT1, RAB3GAP2, RBFOX2, RBM14, RBM15B, RCOR1, REST, RFC2, RNMT, RPA2, RPRD1B, RPS27L, RPS3, RPS4X, RTF1, RUVBL2, SAP130, SCD, SENP1, SET, SETD2, SETSIP, SIGIRR, SIN3A, SKI, SLC27A1, SLC27A2, SLC33A1, SLC9A1, SMARCB1, SMARCD1, SNIP1, SOD2, SP3, SPEN, SPTY2D1, SRM, STAG2, STAT3, STRN3, STT3A, SUPT5H, SUPT6H, SURF1, TAF3, TAF9, TCP1, TECR, TFAM, TFB2M, TMEM115, TMEM165, TOM1L1, TP53BP1, TPR, TRAP1, TRIM25, TRIM27, TSPO, TUFM, UBE2V1, UFL1, UHRF1, USP47, VCP, WDR61, WFS1, WRAP53, XXYLT1, YEATS2, YTHDF2, ZDHHC3, ZDHHC5, ZNF148, ZNF746] | |
|  | GO:1901576 organic substance biosynthetic process | 2.8E-3 | | 16.0E-3 | 560.0E-9 | 1.6E-6 | [3] | 11.53 | | 274.00 | [ABCB6, ABCD3, ACADM, ACLY, ACSF3, ACTN4, ADAR, ADCK3, AK1, ALDOA, ALG8, AP2A1, ASNS, ATAD2, ATF7IP, ATP2B4, ATP5B, ATP5O, B4GAT1, BCOR, BRCA1, BRD2, BRD4, BRD7, BUD31, C14orf166, CAD, CARS, CBX2, CCNT1, CCT2, CCT4, CCT5, CCT6A, CCT7, CCT8, CD276, CD81, CDC73, CDH1, CDK5RAP2, CDK5RAP3, CENPF, CHD6, CHTF8, COA3, COG3, COQ3, CREB1, CTBP1, CTR9, CYP51A1, DDRGK1, DECR2, DHCR7, DHX33, DKC1, DLG1, DMAP1, DNAJA3, DNAJC2, DNAJC3, DNMT1, DNPEP, DPM1, EEF1D, EEF2, EGFR, EHF, EHMT2, EIF1, EIF2A, EIF2B4, EIF2S3, EIF3A, EIF3D, EIF3F, EIF3G, EIF3K, EIF4G2, EIF5, EIF5B, EIF6, ENO1, EPT1, ERBB2, ERBB2IP, ERCC3, ETF1, EZR, FARSB, FXR1, GALNT1, GALNT2, GARS, GATAD2A, GATB, GATC, GBA, GFM1, GFM2, GK, GLB1, GNL3, GNL3L, GTF3C2, GTPBP4, GYS1, HAGH, HARS2, HCFC1, HMGB2, HOGA1, HSPA8, IDI1, ING3, JUN, JUP, KARS, KDM1A, KMT2D, LARP1, LARP4B, LIG3, LTA4H, MAP2K2, MARS2, MBD2, MCAT, MCM2, MCM7, MGST2, MLYCD, MMAB, MPDU1, MPHOSPH8, MRPL41, MRPL44, MTG1, MTHFD1, MYDGF, NABP2, NACC1, NCBP1, NCOA6, NKRF, NOC2L, NOL11, NPAT, NPM1, NR2C2, NSD1, NUP62, OGT, ORMDL1, ORMDL2, PARK7, PDHA1, PDHB, PELP1, PFDN5, PHB, PHF12, PHF2, PHF21A, PHIP, PIAS2, PICALM, PINX1, PKN1, PLK1, PMVK, PNPLA8, POLA1, POLDIP3, POLR1B, POLR2A, POLR2B, POLR2D, POLR2E, POLR2G, POMGNT1, PPIE, PPOX, PRDX5, PRKD1, PRMT5, PSMA6, PSMD2, PTCD3, PTRH2, PUM3, PURA, PYCR1, PYCR2, QARS, QRSL1, QTRT1, RAB3GAP2, RBFOX2, RBM14, RBM15B, RCOR1, REST, RFC2, RNMT, RPA2, RPRD1B, RPS27L, RPS3, RPS4X, RTF1, RUVBL2, SAP130, SCD, SENP1, SET, SETD2, SETSIP, SIGIRR, SIN3A, SKI, SLC27A1, SLC27A2, SLC33A1, SLC9A1, SMARCB1, SMARCD1, SNIP1, SOD2, SORD, SP3, SPEN, SPTY2D1, SRM, STAG2, STAT3, STRN3, STT3A, SUPT5H, SUPT6H, SURF1, TAF3, TAF9, TCP1, TECR, TFAM, TFB2M, TMEM115, TMEM165, TOM1L1, TP53BP1, TPR, TRAP1, TRIM25, TRIM27, TSPO, TUFM, UBE2V1, UFL1, UHRF1, USP47, VCP, VIMP, WDR61, WFS1, WRAP53, XXYLT1, YEATS2, YTHDF2, ZDHHC3, ZDHHC5, ZNF148, ZNF746] | |
|  | GO:0009059 macromolecule biosynthetic process | 11.0E-3 | | 45.0E-3 | 560.0E-9 | 1.6E-6 | [4] | 11.47 | | 230.00 | [ACTN4, ADAR, ALG8, AP2A1, ATAD2, ATF7IP, ATP2B4, B4GAT1, BCOR, BRCA1, BRD2, BRD4, BRD7, BUD31, C14orf166, CARS, CBX2, CCNT1, CCT2, CCT4, CCT5, CCT6A, CCT7, CCT8, CD276, CD81, CDC73, CDH1, CDK5RAP2, CDK5RAP3, CENPF, CHD6, CHTF8, COA3, COG3, CREB1, CTBP1, CTR9, DDRGK1, DHX33, DKC1, DLG1, DMAP1, DNAJA3, DNAJC2, DNAJC3, DNMT1, DNPEP, DPM1, EEF1D, EEF2, EGFR, EHF, EHMT2, EIF1, EIF2A, EIF2B4, EIF2S3, EIF3A, EIF3D, EIF3F, EIF3G, EIF3K, EIF4G2, EIF5, EIF5B, EIF6, ENO1, ERBB2, ERBB2IP, ERCC3, ETF1, EZR, FARSB, FXR1, GALNT1, GALNT2, GARS, GATAD2A, GATB, GATC, GFM1, GFM2, GNL3, GNL3L, GTF3C2, GTPBP4, GYS1, HARS2, HCFC1, HMGB2, HSPA8, ING3, JUN, JUP, KARS, KDM1A, KMT2D, LARP1, LARP4B, LIG3, MAP2K2, MARS2, MBD2, MCM2, MCM7, MMAB, MPHOSPH8, MRPL41, MRPL44, MTG1, MYDGF, NABP2, NACC1, NCBP1, NCOA6, NKRF, NOC2L, NOL11, NPAT, NPM1, NR2C2, NSD1, NUP62, OGT, ORMDL1, ORMDL2, PARK7, PELP1, PFDN5, PHB, PHF12, PHF2, PHF21A, PHIP, PIAS2, PICALM, PINX1, PKN1, PLK1, POLA1, POLDIP3, POLR1B, POLR2A, POLR2B, POLR2D, POLR2E, POLR2G, POMGNT1, PPIE, PRDX5, PRKD1, PRMT5, PSMA6, PSMD2, PTCD3, PTRH2, PUM3, PURA, QARS, QRSL1, RAB3GAP2, RBFOX2, RBM14, RBM15B, RCOR1, REST, RFC2, RNMT, RPA2, RPRD1B, RPS27L, RPS3, RPS4X, RTF1, RUVBL2, SAP130, SENP1, SET, SETD2, SETSIP, SIGIRR, SIN3A, SKI, SLC33A1, SLC9A1, SMARCB1, SMARCD1, SNIP1, SOD2, SP3, SPEN, SPTY2D1, STAG2, STAT3, STRN3, STT3A, SUPT5H, SUPT6H, TAF3, TAF9, TCP1, TFAM, TFB2M, TMEM115, TMEM165, TOM1L1, TP53BP1, TPR, TRAP1, TRIM25, TRIM27, TSPO, TUFM, UBE2V1, UFL1, UHRF1, USP47, VCP, VIMP, WDR61, WFS1, WRAP53, XXYLT1, YEATS2, YTHDF2, ZDHHC3, ZDHHC5, ZNF148, ZNF746] | |
|  | GO:0010467 gene expression | 1.7E-3 | | 11.0E-3 | 560.0E-9 | 1.6E-6 | [4] | 11.73 | | 251.00 | [ADAR, AHCYL1, ALKBH5, ANXA7, APH1A, APH1B, ATAD2, ATF7IP, ATP2B4, BAD, BCOR, BPNT1, BRCA1, BRD2, BRD4, BRD7, BUD31, C14orf166, CARS, CBX2, CCNT1, CD81, CDC73, CDH1, CDK11A, CDK11B, CDK5RAP2, CDK5RAP3, CENPF, CHCHD4, CHD6, CMTR1, COA3, CPSF3, CPSF3L, CREB1, CTBP1, CTR9, CTSH, DDRGK1, DDX1, DDX27, DDX41, DDX54, DHX33, DHX9, DKC1, DLG1, DMAP1, DNAJA3, DNAJC2, DNAJC3, DNMT1, DNPEP, EEF1D, EEF2, EGFR, EHF, EHMT2, EIF1, EIF2A, EIF2B4, EIF2S3, EIF3A, EIF3D, EIF3F, EIF3G, EIF3K, EIF4G2, EIF5, EIF5A, EIF5B, EIF6, ENO1, EPB41L4B, ERBB2, ERBB2IP, ERCC3, ERP29, ETF1, EXOSC10, EZR, FARSB, FLOT2, FN1, FXR1, FYTTD1, GARS, GATAD2A, GATB, GATC, GEMIN5, GFM1, GFM2, GNL3, GTF3C2, HARS2, HCFC1, HMGB2, HNRNPM, HSPA8, ING3, INTS1, INTS2, INTS4, INTS5, INTS8, JUN, JUP, KARS, KDM1A, KMT2D, KRT17, LARP1, LARP4B, LONP2, MAP2K2, MARS2, MBD2, MPHOSPH8, MRPL41, MRPL44, MTG1, MYADM, NACC1, NCBP1, NCOA6, NCSTN, NKRF, NOC2L, NOL11, NOL9, NPAT, NPM1, NR2C2, NSD1, NSRP1, NSUN2, NUDT21, NUP133, NUP62, OGT, PARK7, PDCD5, PELP1, PFDN5, PHB, PHF12, PHF2, PHF21A, PHIP, PIAS2, PICALM, PKN1, PLK1, PMPCA, PNPT1, POLDIP3, POLR2A, POLR2B, POLR2D, POLR2E, POLR2G, PPIE, PQBP1, PRDX5, PRKD1, PRMT5, PRPF19, PRPF8, PSMA6, PTCD3, PTRH2, QARS, QRSL1, RBFOX2, RBM14, RBM15B, RBM25, RBM5, RCOR1, REST, RNF40, RNMT, RNPS1, RPL7, RPRD1B, RPS27L, RPS28, RPS3, RPS4X, RPS6, RPS7, RTCB, RTF1, RUVBL2, SAP130, SENP1, SET, SETD2, SETSIP, SIGIRR, SIN3A, SKI, SLC33A1, SLC9A1, SMARCB1, SMARCD1, SNIP1, SNRNP200, SNRPB, SNRPC, SOD2, SP3, SPEN, SPTY2D1, STAT3, STRN3, SUPT5H, SUPT6H, SUPV3L1, SYMPK, TAF3, TAF9, TFAM, TFB2M, TFIP11, TOP1, TP53BP1, TPR, TRA2B, TRAP1, TRIM25, TRIM27, TRMT10C, TRMU, TUFM, U2AF2, UBE2V1, UFL1, UHRF1, USP47, UTP20, WDR61, WFS1, XPNPEP3, YEATS2, YTHDC1, YTHDF2, ZNF148, ZNF746] | |
|  | GO:0034645 cellular macromolecule biosynthetic process | 10.0E-3 | | 42.0E-3 | 560.0E-9 | 1.6E-6 | [4, 5] | 11.51 | | 223.00 | [ADAR, ALG8, ATAD2, ATF7IP, ATP2B4, B4GAT1, BCOR, BRCA1, BRD2, BRD4, BRD7, BUD31, C14orf166, CARS, CBX2, CCNT1, CCT2, CCT4, CCT5, CCT6A, CCT7, CCT8, CD81, CDC73, CDH1, CDK5RAP2, CDK5RAP3, CENPF, CHD6, CHTF8, COA3, COG3, CREB1, CTBP1, CTR9, DDRGK1, DHX33, DKC1, DLG1, DMAP1, DNAJA3, DNAJC2, DNAJC3, DNMT1, DNPEP, DPM1, EEF1D, EEF2, EGFR, EHF, EHMT2, EIF1, EIF2A, EIF2B4, EIF2S3, EIF3A, EIF3D, EIF3F, EIF3G, EIF3K, EIF4G2, EIF5, EIF5B, EIF6, ENO1, ERBB2, ERBB2IP, ERCC3, ETF1, EZR, FARSB, FXR1, GALNT1, GALNT2, GARS, GATAD2A, GATB, GATC, GFM1, GFM2, GNL3, GNL3L, GTF3C2, GTPBP4, GYS1, HARS2, | |
|  |  |  | |  |  |  |  |  | |  | HCFC1, HMGB2, HSPA8, ING3, JUN, JUP, KARS, KDM1A, KMT2D, LARP1, LARP4B, LIG3, MAP2K2, MARS2, MBD2, MCM2, MCM7, MMAB, MPHOSPH8, MRPL41, MRPL44, MTG1, NABP2, NACC1, NCBP1, NCOA6, NKRF, NOC2L, NOL11, NPAT, NPM1, NR2C2, NSD1, NUP62, OGT, PARK7, PELP1, PFDN5, PHB, PHF12, PHF2, PHF21A, PHIP, PIAS2, PICALM, PINX1, PKN1, PLK1, POLA1, POLDIP3, POLR1B, POLR2A, POLR2B, POLR2D, POLR2E, POLR2G, POMGNT1, PPIE, PRDX5, PRKD1, PRMT5, PSMA6, PSMD2, PTCD3, PTRH2, PUM3, PURA, QARS, QRSL1, RAB3GAP2, RBFOX2, RBM14, RBM15B, RCOR1, REST, RFC2, RNMT, RPA2, RPRD1B, RPS27L, RPS3, RPS4X, RTF1, RUVBL2, SAP130, SENP1, SET, SETD2, SETSIP, SIGIRR, SIN3A, SKI, SLC33A1, SLC9A1, SMARCB1, SMARCD1, SNIP1, SOD2, SP3, SPEN, SPTY2D1, STAG2, STAT3, STRN3, STT3A, SUPT5H, SUPT6H, TAF3, TAF9, TCP1, TFAM, TFB2M, TMEM115, TMEM165, TOM1L1, TP53BP1, TPR, TRAP1, TRIM25, TRIM27, TSPO, TUFM, UBE2V1, UFL1, UHRF1, USP47, VCP, WDR61, WFS1, WRAP53, XXYLT1, YEATS2, YTHDF2, ZDHHC3, ZDHHC5, ZNF148, ZNF746] | |
|  | GO:1901987 regulation of cell cycle phase transition | 5.7E-3 | | 27.0E-3 | 5.7E-3 | 6.8E-3 | [4, 5, 6] | 17.52 | | 24.00 | [ATP2B4, BRD4, BRD7, CDC5L, CDC73, CDCA5, CDK5RAP3, CENPE, CENPF, EGFR, ERCC3, NAE1, NBN, NPM1, PLK1, POLR1B, PRPF19, RAB11A, RPA2, RPS27L, TP53BP1, TPR, USP47, ZW10] | |
|  | GO:0008104 protein localization | 420.0E-9 | | 18.0E-6 | 9.0E-9 | 45.0E-9 | [3] | 14.50 | | 156.00 | [AAK1, ABL1, ADAR, ASUN, ATP1B1, ATP1B3, ATP6V1D, BAG6, BARD1, BCAP31, CCT2, CCT4, CCT5, CCT6A, CCT7, CCT8, CD81, CDCA5, CDH1, CDK5RAP3, CENPF, CEP131, CHCHD4, CHERP, COG3, CYB5R1, DCP1A, DDX1, DDX42, DERL1, DHX9, DKC1, DLG1, DMAP1, DNAJC1, DNM1L, DNM2, EGFR, EIF5A, EPT1, ERBB2, ERCC3, ERP29, EZR, FLOT2, FN1, GBF1, GCC2, GDAP1, GNL3, GNL3L, GOLPH3L, HID1, HK2, HOOK1, HSPA8, HTRA2, HUWE1, IPO5, JUN, JUP, KDM1A, LAMP1, LAMTOR1, LMAN1, LONP2, MACF1, MFN2, MMAB, MTCH2, MYADM, MYO18A, MYO1C, NABP2, NDC1, NEDD4L, NPEPPS, NPLOC4, NPM1, NRAS, NSF, OS9, PARK7, PARL, PCM1, PDCD5, PEX13, PEX16, PICALM, PINX1, PKP2, PKP3, PLK1, PMPCA, POLR2D, PPFIA1, PPIA, PPIL2, PPT1, PREB, PRPF19, RAB10, RAB11A, RAB11B, RAB3GAP2, RAB7A, RAB9A, RAC2, RAP1A, REST, RPL10, RUVBL1, RUVBL2, SAMM50, SCARB2, SCRIB, SEC61A1, SEC62, SEC63, SIN3A, SLC9A1, SNIP1, SNX3, SRP54, STAT3, STX3, STX8, STXBP1, SYNE1, SYNE2, SYNE3, TAF3, TAP1, TAX1BP3, TCP1, TIMM10, TIMM21, TMBIM1, TOMM34, TOR1A, TP53BP1, TPR, TRIM27, U2AF1, U2AF2, UBAC2, UFL1, USP36, VCP, VIMP, VTI1B, WDR46, WRAP53, XPO1, XPO7, ZW10] | |
|  | GO:0051649 establishment of localization in cell | 3.3E-6 | | 98.0E-6 | 9.0E-9 | 45.0E-9 | [3] | 14.85 | | 121.00 | [AAAS, ACTN4, ADAR, ALKBH5, ATL2, ATP5O, BARD1, BCAP31, BET1L, BORCS5, CDCA5, CDCA8, CDH1, CENPE, CENPF, CEP131, CHCHD4, CHERP, COG3, CYB5R1, DCTN1, DERL1, DLG1, DMAP1, DYNC1H1, EGFR, EIF5A, EPS15, EPT1, ERBB2, EZR, FYTTD1, GBF1, GCC2, GDAP1, GOSR1, HID1, HOOK1, HSPA8, HTATIP2, HTRA2, HUWE1, IPO5, JUN, JUP, KIF22, KIF2C, LAMP1, LMAN1, LONP2, LYST, MAD1L1, MAP4, MFN2, MYADM, MYO1C, NCBP1, NOC2L, NPEPPS, NPLOC4, NPM1, NSF, NSRP1, NUP133, NUP153, OS9, PARK7, PARL, PCM1, PDCD5, PDCD6IP, PEX13, PEX16, PINX1, PMPCA, POLDIP3, PREB, RAB11A, RAB2A, RAB3B, RAB7A, RAC2, RBM15B, RHOT1, RPL10, RUVBL1, SAMM50, SCARB2, SEC61A1, SEC62, SEC63, SETD2, SLC9A1, SNIP1, SNX3, SNX5, SPAG9, SRP54, STAT3, STX18, STX8, SYNE2, TAP1, TIMM10, TIMM21, TOMM34, TOR1A, TPR, TRIM27, U2AF1, U2AF2, UBAC2, USP36, VAMP7, VCP, VIMP, VTI1A, WDR46, XPO1, XPO7, ZW10] | |
|  | GO:0070727 cellular macromolecule localization | 2.1E-9 | | 280.0E-9 | 9.0E-9 | 45.0E-9 | [3] | 16.38 | | 133.00 | [ADAR, ASUN, ATP1B1, ATP1B3, ATP6V1D, BAG6, BARD1, BCAP31, CCT2, CCT4, CCT5, CCT6A, CCT7, CCT8, CD81, CDCA5, CDH1, CDK5RAP3, CEP131, CHCHD4, CHERP, COG3, CYB5R1, DCP1A, DDX1, DERL1, DHX9, DKC1, DLG1, DMAP1, EGFR, EIF5A, EPT1, ERBB2, EXOSC10, EZR, FLOT2, GBF1, GCC2, GDAP1, GNL3, GNL3L, HID1, HK2, HOOK1, HSPA8, HTRA2, HUWE1, IPO5, JUN, JUP, KDM1A, LAMP1, LAMTOR1, LMAN1, LONP2, MACF1, MFN2, MMAB, MTCH2, MYADM, MYO1C, NABP2, NDC1, NEDD4L, NPEPPS, NPLOC4, NRAS, NSF, OS9, PARK7, PARL, PCM1, PDCD5, PEX13, PEX16, PINX1, PKP2, PKP3, PLK1, PMPCA, PPFIA1, PPIL2, PREB, PRPF19, RAB10, RAB11A, RAB11B, RAB3GAP2, RAB7A, RAC2, RPL10, RUVBL1, RUVBL2, SAMM50, SCARB2, SEC61A1, SEC62, SEC63, SIN3A, SLC9A1, SNIP1, SRP54, STAT3, STX3, STX8, STXBP1, SYNE1, SYNE2, SYNE3, TAF3, TAP1, TAX1BP3, TCP1, TIMM10, TIMM21, TMBIM1, TOMM34, TOR1A, TP53BP1, TPR, U2AF1, U2AF2, UBAC2, USP36, VCP, VIMP, VTI1B, WDR46, WRAP53, XPO1, XPO7, ZW10] | |
|  | GO:0032880 regulation of protein localization | 8.3E-3 | | 37.0E-3 | 9.0E-9 | 45.0E-9 | [3, 4] | 13.35 | | 75.00 | [AAK1, BARD1, BCAP31, CCT2, CCT4, CCT5, CCT6A, CCT7, CCT8, CDCA5, CDH1, CDK5RAP3, CEP131, CHERP, CYB5R1, DKC1, DLG1, DMAP1, DNAJC1, DNM1L, EGFR, EPT1, ERBB2, ERP29, EZR, FN1, GBF1, GCC2, GNL3, GNL3L, GOLPH3L, HTRA2, HUWE1, JUP, KDM1A, MYO18A, MYO1C, NEDD4L, NPEPPS, OS9, PARK7, PARL, PCM1, PDCD5, PICALM, PINX1, POLR2D, PPFIA1, PPIA, RAB11B, RAB9A, RAC2, REST, RPL10, RUVBL1, SIN3A, SLC9A1, SNIP1, SNX3, STX3, STX8, TAX1BP3, TCP1, TMBIM1, TOR1A, TPR, TRIM27, U2AF1, U2AF2, UBAC2, UFL1, USP36, VTI1B, WDR46, WRAP53] | |
|  | GO:0045184 establishment of protein localization | 67.0E-6 | | 970.0E-6 | 9.0E-9 | 45.0E-9 | [3, 4] | 14.16 | | 114.00 | [ABL1, ADAR, BAG6, BARD1, BCAP31, CCT2, CCT4, CCT5, CCT6A, CCT7, CCT8, CDH1, CENPF, CEP131, CHCHD4, CHERP, CYB5R1, DERL1, DKC1, DLG1, DMAP1, DNAJC1, DNM1L, DNM2, EGFR, EIF5A, EPT1, ERBB2, ERP29, EZR, FLOT2, FN1, GCC2, GDAP1, GOLPH3L, HID1, HK2, HOOK1, HSPA8, HTRA2, HUWE1, IPO5, JUN, JUP, LAMP1, LMAN1, LONP2, MACF1, MFN2, MYADM, MYO18A, MYO1C, NABP2, NPEPPS, NPLOC4, NRAS, NSF, OS9, PARK7, PARL, PCM1, PDCD5, PEX13, PEX16, PKP2, PKP3, PLK1, PMPCA, POLR2D, PPFIA1, PPIA, PPT1, PREB, RAB10, RAB11A, RAB11B, RAB3GAP2, RAB7A, RAC2, RAP1A, REST, RPL10, RUVBL1, RUVBL2, SAMM50, SCARB2, SEC61A1, SEC62, SEC63, SLC9A1, SNIP1, SNX3, SRP54, STAT3, SYNE3, TAP1, TCP1, TIMM10, TIMM21, TMBIM1, TOMM34, TOR1A, TPR, TRIM27, U2AF1, U2AF2, UBAC2, USP36, VCP, VIMP, WDR46, WRAP53, XPO1, XPO7] | |
|  | GO:0046907 intracellular transport | 3.5E-6 | | 99.0E-6 | 9.0E-9 | 45.0E-9 | [3, 4] | 15.37 | | 105.00 | [AAAS, ACTN4, ADAR, ALKBH5, ATL2, ATP5O, BARD1, BCAP31, BET1L, BORCS5, CDH1, CEP131, CHCHD4, CHERP, COG3, CYB5R1, DCTN1, DERL1, DMAP1, EGFR, EIF5A, EPS15, EPT1, ERBB2, EZR, FYTTD1, GBF1, GCC2, GDAP1, GOSR1, HID1, HOOK1, HSPA8, HTATIP2, HTRA2, HUWE1, IPO5, JUN, JUP, LAMP1, LMAN1, LONP2, LYST, MFN2, MYADM, MYO1C, NCBP1, NOC2L, NPEPPS, NPLOC4, NPM1, NSF, NSRP1, NUP133, NUP153, OS9, PARK7, PARL, PCM1, PDCD5, PEX13, PEX16, PMPCA, POLDIP3, PREB, RAB2A, RAB7A, RAC2, RBM15B, RHOT1, RPL10, RUVBL1, SAMM50, SCARB2, SEC61A1, SEC62, SEC63, SETD2, SLC9A1, SNIP1, SNX3, SNX5, SPAG9, SRP54, STAT3, STX18, STX8, TAP1, TIMM10, TIMM21, TOMM34, TPR, TRIM27, U2AF1, U2AF2, UBAC2, USP36, VAMP7, VCP, VIMP, VTI1A, WDR46, XPO1, XPO7, ZW10] | |
|  | GO:0060341 regulation of cellular localization | 3.7E-3 | | 20.0E-3 | 9.0E-9 | 45.0E-9 | [3, 4] | 13.99 | | 68.00 | [BARD1, BCAP31, CCT2, CCT4, CCT5, CCT6A, CCT7, CCT8, CDCA5, CDH1, CDK5RAP3, CEP131, CHERP, CYB5R1, DKC1, DLG1, DMAP1, EGFR, EPT1, ERBB2, EZR, GBF1, GCC2, GNL3, GNL3L, HTRA2, HUWE1, JUP, KDM1A, LAMP1, MAD1L1, MYO1C, NEDD4L, NPEPPS, NUP153, OS9, PARK7, PARL, PCM1, PDCD5, PINX1, PPFIA1, RAB11B, RAB3B, RAC2, RPL10, RUVBL1, SETD2, SIN3A, SLC9A1, SNIP1, SNX3, STX18, STX3, STX8, TAX1BP3, TCP1, TMBIM1, TOR1A, TPR, U2AF1, U2AF2, UBAC2, USP36, VAMP7, VTI1B, WDR46, WRAP53] | |
|  | GO:0006839 mitochondrial transport | 140.0E-6 | | 1.7E-3 | 9.0E-9 | 45.0E-9 | [4] | 19.41 | | 33.00 | [ACAA2, ATP5O, BAD, CHCHD4, CYB5R1, EPT1, FLVCR1, GDAP1, HTRA2, HUWE1, MFN2, MICU2, NPEPPS, PARL, PDCD5, PMPCA, PNPT1, PPIF, RAC2, RHOT1, RUVBL1, SAMM50, SLC25A12, SLC25A24, SNIP1, THEM4, TIMM10, TIMM21, TOMM34, U2AF1, U2AF2, USP36, WDR46] | |
|  | GO:1903829 positive regulation of cellular protein localization | 820.0E-6 | | 7.2E-3 | 9.0E-9 | 45.0E-9 | [2, 3, 4, 5, 6] | 16.67 | | 42.00 | [BCAP31, CCT2, CCT4, CCT5, CCT6A, CCT7, CCT8, CDH1, CDK5RAP3, CEP131, CHERP, CYB5R1, DKC1, DLG1, DMAP1, EGFR, EPT1, ERBB2, EZR, GNL3, GNL3L, HTRA2, HUWE1, JUP, MYO1C, NPEPPS, PARK7, PCM1, PDCD5, PINX1, RAC2, RUVBL1, SLC9A1, SNIP1, STX3, TCP1, TPR, U2AF1, U2AF2, USP36, WDR46, WRAP53] | |
|  | GO:1903827 regulation of cellular protein localization | 290.0E-6 | | 3.1E-3 | 9.0E-9 | 45.0E-9 | [4, 5] | 15.75 | | 60.00 | [BARD1, BCAP31, CCT2, CCT4, CCT5, CCT6A, CCT7, CCT8, CDCA5, CDH1, CDK5RAP3, CEP131, CHERP, CYB5R1, DKC1, DLG1, DMAP1, EGFR, EPT1, ERBB2, EZR, GBF1, GCC2, GNL3, GNL3L, HTRA2, HUWE1, JUP, KDM1A, MYO1C, NEDD4L, NPEPPS, OS9, PARK7, PARL, PCM1, PDCD5, PINX1, PPFIA1, RAB11B, RAC2, RPL10, RUVBL1, SIN3A, SLC9A1, SNIP1, STX3, STX8, TAX1BP3, TCP1, TMBIM1, TOR1A, TPR, U2AF1, U2AF2, UBAC2, USP36, VTI1B, WDR46, WRAP53] | |
|  | GO:0006886 intracellular protein transport | 430.0E-6 | | 4.3E-3 | 9.0E-9 | 45.0E-9 | [4, 5, 6] | 15.09 | | 67.00 | [ADAR, BARD1, BCAP31, CDH1, CEP131, CHCHD4, CHERP, CYB5R1, DERL1, DMAP1, EGFR, EIF5A, EPT1, ERBB2, GCC2, GDAP1, HID1, HSPA8, HTRA2, HUWE1, IPO5, JUN, JUP, LMAN1, LONP2, MFN2, MYADM, MYO1C, NPEPPS, NPLOC4, NSF, OS9, PARK7, PARL, PCM1, PDCD5, PEX13, PEX16, PMPCA, PREB, RAB7A, RAC2, RPL10, RUVBL1, SAMM50, SCARB2, SEC61A1, SEC62, SEC63, SLC9A1, SNIP1, SRP54, STAT3, TAP1, TIMM10, TIMM21, TOMM34, TPR, U2AF1, U2AF2, UBAC2, USP36, VCP, VIMP, WDR46, XPO1, XPO7] | |
|  | GO:0033365 protein localization to organelle | 7.7E-9 | | 600.0E-9 | 9.0E-9 | 45.0E-9 | [5] | 18.13 | | 91.00 | [ADAR, ASUN, ATP6V1D, BAG6, CCT2, CCT4, CCT5, CCT6A, CCT7, CCT8, CD81, CDCA5, CDH1, CDK5RAP3, CEP131, CHCHD4, CHERP, COG3, CYB5R1, DCP1A, DDX1, DHX9, DKC1, DMAP1, EGFR, EPT1, EZR, GBF1, GCC2, GDAP1, GNL3, GNL3L, HK2, HOOK1, HSPA8, HTRA2, HUWE1, IPO5, JUN, JUP, LAMP1, LONP2, MFN2, MMAB, MTCH2, NABP2, NPEPPS, NRAS, OS9, PARK7, PARL, PCM1, PDCD5, PEX13, PEX16, PINX1, PLK1, PMPCA, RAB10, RAB11A, RAB3GAP2, RAB7A, RAC2, RPL10, RUVBL1, RUVBL2, SAMM50, SCARB2, SEC61A1, SEC62, SEC63, SIN3A, SLC9A1, SNIP1, SRP54, STAT3, SYNE1, TAF3, TCP1, TIMM10, TIMM21, TOMM34, TOR1A, TPR, U2AF1, U2AF2, UBAC2, USP36, WDR46, WRAP53, ZW10] | |
|  | GO:0072594 establishment of protein localization to organelle | 1.0E-6 | | 38.0E-6 | 9.0E-9 | 45.0E-9 | [4, 5, 6] | 18.48 | | 63.00 | [ADAR, CCT2, CCT4, CCT5, CCT6A, CCT7, CCT8, CDH1, CHCHD4, CHERP, CYB5R1, DKC1, DMAP1, EGFR, EPT1, GCC2, GDAP1, HK2, HOOK1, HSPA8, HTRA2, HUWE1, IPO5, JUN, JUP, LAMP1, LONP2, MFN2, NABP2, NPEPPS, NRAS, PARL, PDCD5, PEX13, PEX16, PMPCA, RAB10, RAB11A, RAB3GAP2, RAB7A, RAC2, RPL10, RUVBL1, RUVBL2, SAMM50, SCARB2, SEC61A1, SEC62, SEC63, SLC9A1, SNIP1, SRP54, STAT3, TCP1, TIMM10, TIMM21, TOMM34, TPR, U2AF1, U2AF2, USP36, WDR46, WRAP53] | |
|  | GO:1900182 positive regulation of protein localization to nucleus | 4.8E-3 | | 24.0E-3 | 9.0E-9 | 45.0E-9 | [3, 4, 5, 6, 7, 8] | 20.24 | | 17.00 | [CCT2, CCT4, CCT5, CCT6A, CCT7, CCT8, CDH1, CDK5RAP3, CHERP, DMAP1, EGFR, JUP, PARK7, PINX1, SLC9A1, TCP1, TPR] | |
|  | GO:0006605 protein targeting | 8.9E-3 | | 38.0E-3 | 9.0E-9 | 45.0E-9 | [5, 6, 7] | 14.33 | | 48.00 | [ADAR, CDH1, CHCHD4, CHERP, CYB5R1, DMAP1, EGFR, EPT1, ERBB2, GCC2, GDAP1, HSPA8, HTRA2, HUWE1, IPO5, JUN, JUP, LONP2, MFN2, MYADM, MYO1C, NPEPPS, PARL, PDCD5, PEX13, PEX16, PMPCA, RAB7A, RAC2, RPL10, RUVBL1, SAMM50, SCARB2, SEC61A1, SEC62, SEC63, SLC9A1, SNIP1, SRP54, STAT3, TIMM10, TIMM21, TOMM34, TPR, U2AF1, U2AF2, USP36, WDR46] | |
|  | GO:0070585 protein localization to mitochondrion | 2.2E-3 | | 13.0E-3 | 9.0E-9 | 45.0E-9 | [6] | 18.38 | | 25.00 | [CHCHD4, CYB5R1, EPT1, GDAP1, HK2, HOOK1, HTRA2, HUWE1, MFN2, MTCH2, NPEPPS, PARL, PDCD5, PMPCA, RAC2, RUVBL1, SAMM50, SNIP1, TIMM10, TIMM21, TOMM34, U2AF1, U2AF2, USP36, WDR46] | |
|  | GO:1904816 positive regulation of protein localization to chromosome, telomeric region | 99.0E-12 | | 92.0E-9 | 9.0E-9 | 45.0E-9 | [3, 4, 5, 6, 7, 8, 9] | 91.67 | | 11.00 | [CCT2, CCT4, CCT5, CCT6A, CCT7, CCT8, DKC1, GNL3, GNL3L, TCP1, WRAP53] | |
|  | GO:0006626 protein targeting to mitochondrion | 5.2E-3 | | 26.0E-3 | 9.0E-9 | 45.0E-9 | [5, 6, 7, 8] | 18.18 | | 22.00 | [CHCHD4, CYB5R1, EPT1, GDAP1, HTRA2, HUWE1, MFN2, NPEPPS, PARL, PDCD5, PMPCA, RAC2, RUVBL1, SAMM50, SNIP1, TIMM10, TIMM21, TOMM34, U2AF1, U2AF2, USP36, WDR46] | |
|  | GO:0070198 protein localization to chromosome, telomeric region | 150.0E-9 | | 9.2E-6 | 9.0E-9 | 45.0E-9 | [7] | 48.28 | | 14.00 | [CCT2, CCT4, CCT5, CCT6A, CCT7, CCT8, DKC1, GNL3, GNL3L, MMAB, NABP2, PINX1, TCP1, WRAP53] | |
|  | GO:0007004 telomere maintenance via telomerase | 1.7E-3 | | 11.0E-3 | 9.0E-9 | 45.0E-9 | [7, 8, 9] | 26.83 | | 11.00 | [CCT2, CCT4, CCT5, CCT6A, CCT7, CCT8, DKC1, GNL3L, PINX1, TCP1, WRAP53] | |
|  | GO:1990542 mitochondrial transmembrane transport | 3.1E-3 | | 18.0E-3 | 7.4E-3 | 8.6E-3 | [5] | 33.33 | | 7.00 | [ATP5O, CHCHD4, MICU2, PDCD5, SAMM50, TIMM10, TIMM21] | |
|  | GO:0044743 intracellular protein transmembrane import | 11.0E-3 | | 46.0E-3 | 7.4E-3 | 8.6E-3 | [5, 6, 7, 8] | 26.92 | | 7.00 | [CHCHD4, PDCD5, PEX13, PEX16, SAMM50, TIMM10, TIMM21] | |
|  | GO:0006486 protein glycosylation | 11.0E-3 | | 47.0E-3 | 11.0E-3 | 12.0E-3 | [3, 6, 7] | 19.23 | | 15.00 | [ALG8, B4GAT1, COG3, DPM1, GALNT1, GALNT2, OGT, POMGNT1, PSMD2, PUM3, STT3A, TMEM115, TMEM165, VCP, XXYLT1] | |
|  | GO:0006996 organelle organization | 260.0E-12 | | 120.0E-9 | 260.0E-12 | 2.1E-9 | [3] | 14.60 | | 217.00 | [ABCC4, ABCD3, ABL1, ACAA2, ACAD9, ACBD5, ACTN1, ACTR2, ACTR3, ANKLE2, ARHGDIA, ARPIN, ASUN, ATL2, ATP6V1D, ATXN2, ATXN2L, BAD, BAG6, BAIAP2, BAX, BNIP1, BRD2, CCT2, CCT4, CCT5, CCT6A, CCT7, CCT8, CDC27, CDCA5, CDCA8, CDK5RAP2, CENPE, CEP131, CHCHD4, CKAP5, COA3, COG2, COG3, COG4, CYB5R1, DDX1, DDX6, DHX30, DKC1, DLG1, DNM1L, DYNC1H1, EFNA5, EIF2A, EIF6, EMC6, EPB41L4B, EPHA3, EPT1, ERCC3, EZR, FASTKD2, FITM2, FOXRED1, GATB, GATC, GBF1, GCC2, GDAP1, GFM1, GFM2, GNL3, GNL3L, GOLGA5, GOLPH3L, HIRA, HK2, HOOK1, HTRA2, HUWE1, IMMT, KIF22, KIF2C, LEMD2, LIG3, LMAN1, LONP2, MAP4, MAPT, MFN1, MFN2, MICAL3, MIS12, MKI67, MRPL44, MRRF, MTG1, MTPN, MYADM, MYH10, MYH14, MYO18A, NABP2, NBN, NCAPD2, NCAPH, NDC1, NDUFAF4, NDUFS1, NDUFS8, NOC2L, NPEPPS, NPM1, NUBPL, NUP133, NUP153, OPA1, PARL, PCM1, PDCD5, PDCD6IP, PDZD8, PEX11G, PEX13, PEX16, PFDN2, PFN1, PHB, PHIP, PINX1, PKN1, PKP2, PLK1, PMPCA, PNPT1, POGZ, POLR1B, PPFIA1, PPIA, PPIF, PPT1, PQBP1, PRKD1, PRMT5, PTCD3, PURA, QRSL1, RAB10, RAB11A, RAB18, RAB2A, RAB3GAP2, RAB7A, RAC1, RAC2, RAC3, RALA, RBM14, RHOA, RHOT1, RPA2, RPS3, RTN4, RUVBL1, RUVBL2, SAMM50, SDCBP, SEC61A1, SFN, SGSM3, C2, SMIM20, SNIP1, SNX3, SPTY2D1, STAG2, STX12, STX18, | |
|  | GO:0048513 animal organ development | 180.0E-9 | | 9.3E-6 | 46.0E-12 | 460.0E-12 | [3, 4, 5] | 5.06 | | 40.00 | [ABCB6, ACTN1, ATP5F1, BCOR, CDC73, CENPF, CKB, COPS2, CTR9, DSG2, DSP, EIF2B4, EZR, FLVCR1, GNB4, HMGB2, JAGN1, LEMD2, MAFB, MYDGF, MYH14, NCOA6, NDUFS3, OGT, PIR, PKP2, PLS3, POLR2D, PPT1, PRMT1, PTPN11, PTRH2, QARS, RHOA, RPL10, SDCBP, WDR61, WFS1, YWHAE, ZNF148] | |
|  | GO:0045597 positive regulation of cell differentiation | 5.4E-3 | | 27.0E-3 | 46.0E-12 | 460.0E-12 | [3, 4, 5, 6] | 5.41 | | 16.00 | [EPHA3, HMGB2, MACF1, MAPT, MARK2, MYADM, NEDD4L, OGT, PRKD1, PRMT1, RAC1, RAC3, RHOA, SDCBP, SPEN, TRIOBP] | |
|  | GO:0040012 regulation of locomotion | 8.5E-3 | | 37.0E-3 | 1.1E-3 | 1.6E-3 | [2, 3] | 6.39 | | 28.00 | [ACTN4, ARID2, ARPIN, CFAP20, CTSH, EGFR, EPB41L4B, FAM60A, FN1, GTPBP4, MYADM, MYO1C, PFN1, PKN1, PRKD1, PTPRJ, RAB11A, RAC1, RAC2, RHOA, RNMT, RPL10, RTN4, SDCBP, SLK, SPAG9, STX3, SYNJ2BP] | |
|  | GO:0006928 movement of cell or subcellular component | 860.0E-6 | | 7.3E-3 | 1.1E-3 | 1.6E-3 | [3] | 6.51 | | 47.00 | [ACTN1, ACTN4, AIMP1, ARID2, ARPIN, BORCS5, CD9, CEP131, CFAP20, CTSH, DSG2, DSP, EGFR, EPB41L4B, FAM60A, FN1, GBF1, GTPBP4, HMGB2, ITGA2, JUP, LAMP1, MAP4, MYADM, MYH10, MYO18A, MYO1C, PCM1, PFN1, PKN1, PKP2, PRKD1, PTPRJ, RAB11A, RAC1, RAC2, RHOA, RHOT1, RNMT, RPL10, RTN4, S100P, SCRIB, SDCBP, SLK, SPAG9, SYNJ2BP] | |
|  | GO:0048870 cell motility | 67.0E-6 | | 950.0E-6 | 1.1E-3 | 1.6E-3 | [2, 3, 4] | 5.60 | | 35.00 | [ACTN4, AIMP1, ARID2, ARPIN, CFAP20, CTSH, EGFR, EPB41L4B, FAM60A, FN1, GBF1, GTPBP4, HMGB2, ITGA2, JUP, MYADM, MYO18A, MYO1C, PFN1, PKN1, PRKD1, PTPRJ, RAB11A, RAC1, RAC2, RHOA, RNMT, RPL10, RTN4, S100P, SCRIB, SDCBP, SLK, SPAG9, SYNJ2BP] | |
|  | GO:0006403 RNA localization | 26.0E-9 | | 1.8E-6 | 3.6E-9 | 22.0E-9 | [3] | 33.33 | | 25.00 | [ALKBH5, CCT2, CCT4, CCT5, CCT6A, CCT7, CCT8, DKC1, EIF5A, EXOSC10, FBL, FYTTD1, MMAB, NCBP1, NOP10, NUP133, NUP153, PNPT1, POLDIP3, RUVBL1, RUVBL2, SETD2, TCP1, TPR, WRAP53] | |
|  | GO:0031647 regulation of protein stability | 18.0E-6 | | 410.0E-6 | 3.6E-9 | 22.0E-9 | [3] | 20.57 | | 36.00 | [AAK1, ATP1B1, ATP1B3, BAG6, CCT2, CCT4, CCT5, CCT6A, CCT7, CCT8, CD81, CDC73, COG3, CTSA, CTSH, DERL1, DNAJA3, FLOT2, GNL3L, GTPBP4, HCFC1, HSPA8, NCLN, NOMO2, PARK7, PFN1, PHB, PINX1, PLK1, PRKD1, STX12, TAF9, TCP1, TPR, TRIM24, WFS1] | |
|  | GO:0050821 protein stabilization | 1.4E-3 | | 10.0E-3 | 3.6E-9 | 22.0E-9 | [4] | 20.19 | | 21.00 | [AAK1, ATP1B1, ATP1B3, CCT2, CCT4, CCT5, CCT6A, CCT7, CCT8, COG3, DNAJA3, FLOT2, GTPBP4, HCFC1, PARK7, PFN1, PHB, STX12, TAF9, TCP1, WFS1] | |
|  | GO:0051276 chromosome organization | 230.0E-6 | | 2.5E-3 | 3.6E-9 | 22.0E-9 | [4] | 17.23 | | 46.00 | [BRD2, CCT2, CCT4, CCT5, CCT6A, CCT7, CCT8, CDC27, CDCA5, CDCA8, CENPE, DDX1, DKC1, ERCC3, GNL3, GNL3L, HIRA, KIF22, KIF2C, MIS12, NABP2, NBN, NCAPD2, NCAPH, NOC2L, NPM1, PDCD6IP, PINX1, PLK1, POGZ, POLR1B, PURA, RAB11A, RPA2, RUVBL2, SMC2, SPTY2D1, STAG2, SUPV3L1, TCP1, TOP1, TOP2A, TP53BP1, TPR, WRAP53, ZW10] | |
|  | GO:1903829 positive regulation of cellular protein localization | 820.0E-6 | | 7.2E-3 | 3.6E-9 | 22.0E-9 | [2, 3, 4, 5, 6] | 16.67 | | 42.00 | [BCAP31, CCT2, CCT4, CCT5, CCT6A, CCT7, CCT8, CDH1, CDK5RAP3, CEP131, CHERP, CYB5R1, DKC1, DLG1, DMAP1, EGFR, EPT1, ERBB2, EZR, GNL3, GNL3L, HTRA2, HUWE1, JUP, MYO1C, NPEPPS, PARK7, PCM1, PDCD5, PINX1, RAC2, RUVBL1, SLC9A1, SNIP1, STX3, TCP1, TPR, U2AF1, U2AF2, USP36, WDR46, WRAP53] | |
|  | GO:1903827 regulation of cellular protein localization | 290.0E-6 | | 3.1E-3 | 3.6E-9 | 22.0E-9 | [4, 5] | 15.75 | | 60.00 | [BARD1, BCAP31, CCT2, CCT4, CCT5, CCT6A, CCT7, CCT8, CDCA5, CDH1, CDK5RAP3, CEP131, CHERP, CYB5R1, DKC1, DLG1, DMAP1, EGFR, EPT1, ERBB2, EZR, GBF1, GCC2, GNL3, GNL3L, HTRA2, HUWE1, JUP, KDM1A, MYO1C, NEDD4L, NPEPPS, OS9, PARK7, PARL, PCM1, PDCD5, PINX1, PPFIA1, RAB11B, RAC2, RPL10, RUVBL1, SIN3A, SLC9A1, SNIP1, STX3, STX8, TAX1BP3, TCP1, TMBIM1, TOR1A, TPR, U2AF1, U2AF2, UBAC2, USP36, VTI1B, WDR46, WRAP53] | |
|  | GO:0015931 nucleobase-containing compound transport | 3.3E-3 | | 19.0E-3 | 3.6E-9 | 22.0E-9 | [5] | 22.81 | | 13.00 | [ALKBH5, EIF5A, FYTTD1, NCBP1, NUP133, NUP153, PNPT1, POLDIP3, SETD2, SLC25A17, SLC25A24, SLC35B2, TPR] | |
|  | GO:0090666 scaRNA localization to Cajal body | 98.0E-6 | | 1.3E-3 | 3.6E-9 | 22.0E-9 | [5] | 100.00 | | 4.00 | [CCT2, CCT4, TCP1, WRAP53] | |
|  | GO:0090671 telomerase RNA localization to Cajal body | 3.4E-9 | | 390.0E-9 | 3.6E-9 | 22.0E-9 | [5] | 70.59 | | 12.00 | [CCT2, CCT4, CCT5, CCT6A, CCT7, CCT8, DKC1, NOP10, RUVBL1, RUVBL2, TCP1, WRAP53] | |
|  | GO:0032204 regulation of telomere maintenance | 6.6E-3 | | 31.0E-3 | 3.6E-9 | 22.0E-9 | [3, 4, 5, 6, 7, 8] | 22.03 | | 13.00 | [CCT2, CCT4, CCT5, CCT6A, CCT7, CCT8, GNL3, GNL3L, NABP2, NBN, PINX1, TCP1, WRAP53] | |
|  | GO:0033044 regulation of chromosome organization | 6.8E-3 | | 32.0E-3 | 3.6E-9 | 22.0E-9 | [5, 6] | 18.63 | | 19.00 | [CCT2, CCT4, CCT5, CCT6A, CCT7, CCT8, CDC27, CDCA5, CENPE, GNL3, GNL3L, NABP2, NBN, PINX1, PLK1, SPTY2D1, TCP1, TPR, WRAP53] | |
|  | GO:1900182 positive regulation of protein localization to nucleus | 4.8E-3 | | 24.0E-3 | 3.6E-9 | 22.0E-9 | [3, 4, 5, 6, 7, 8] | 20.24 | | 17.00 | [CCT2, CCT4, CCT5, CCT6A, CCT7, CCT8, CDH1, CDK5RAP3, CHERP, DMAP1, EGFR, JUP, PARK7, PINX1, SLC9A1, TCP1, TPR] | |
|  | GO:0000723 telomere maintenance | 7.5E-3 | | 34.0E-3 | 3.6E-9 | 22.0E-9 | [5, 6, 7] | 19.75 | | 16.00 | [CCT2, CCT4, CCT5, CCT6A, CCT7, CCT8, DKC1, GNL3, GNL3L, NABP2, NBN, PINX1, POLR1B, RPA2, TCP1, WRAP53] | |
|  | GO:0034502 protein localization to chromosome | 4.2E-6 | | 100.0E-6 | 3.6E-9 | 22.0E-9 | [6] | 32.14 | | 18.00 | [CCT2, CCT4, CCT5, CCT6A, CCT7, CCT8, CDCA5, DKC1, GNL3, GNL3L, MMAB, NABP2, PINX1, PLK1, RUVBL2, TCP1, WRAP53, ZW10] | |
|  | GO:0070199 establishment of protein localization to chromosome | 2.2E-6 | | 75.0E-6 | 3.6E-9 | 22.0E-9 | [5, 6, 7] | 50.00 | | 11.00 | [CCT2, CCT4, CCT5, CCT6A, CCT7, CCT8, DKC1, NABP2, RUVBL2, TCP1, WRAP53] | |
|  | GO:0071897 DNA biosynthetic process | 4.9E-3 | | 25.0E-3 | 3.6E-9 | 22.0E-9 | [5, 6, 7] | 19.35 | | 18.00 | [CCT2, CCT4, CCT5, CCT6A, CCT7, CCT8, CHTF8, DKC1, GNL3L, NABP2, PINX1, POLA1, RFC2, TCP1, TOM1L1, TP53BP1, VCP, WRAP53] | |
|  | GO:1904816 positive regulation of protein localization to chromosome, telomeric region | 99.0E-12 | | 92.0E-9 | 3.6E-9 | 22.0E-9 | [3, 4, 5, 6, 7, 8, 9] | 91.67 | | 11.00 | [CCT2, CCT4, CCT5, CCT6A, CCT7, CCT8, DKC1, GNL3, GNL3L, TCP1, WRAP53] | |
|  | GO:2001252 positive regulation of chromosome organization | 2.5E-3 | | 15.0E-3 | 3.6E-9 | 22.0E-9 | [5, 6, 7] | 24.49 | | 12.00 | [CCT2, CCT4, CCT5, CCT6A, CCT7, CCT8, GNL3, NABP2, NBN, TCP1, TPR, WRAP53] | |
|  | GO:0071426 ribonucleoprotein complex export from nucleus | 10.0E-3 | | 44.0E-3 | 3.6E-9 | 22.0E-9 | [4, 7, 8] | 23.68 | | 9.00 | [ALKBH5, EIF5A, FYTTD1, NCBP1, NUP133, POLDIP3, SETD2, TPR, XPO1] | |
|  | GO:2000278 regulation of DNA biosynthetic process | 2.5E-3 | | 15.0E-3 | 3.6E-9 | 22.0E-9 | [5, 6, 7, 8] | 21.62 | | 16.00 | [CCT2, CCT4, CCT5, CCT6A, CCT7, CCT8, CHTF8, DKC1, GNL3L, NABP2, PINX1, RFC2, TCP1, TOM1L1, TP53BP1, WRAP53] | |
|  | GO:0070198 protein localization to chromosome, telomeric region | 150.0E-9 | | 9.2E-6 | 3.6E-9 | 22.0E-9 | [7] | 48.28 | | 14.00 | [CCT2, CCT4, CCT5, CCT6A, CCT7, CCT8, DKC1, GNL3, GNL3L, MMAB, NABP2, PINX1, TCP1, WRAP53] | |
|  | GO:2000573 positive regulation of DNA biosynthetic process | 3.8E-3 | | 20.0E-3 | 3.6E-9 | 22.0E-9 | [5, 6, 7, 8, 9] | 24.44 | | 11.00 | [CCT2, CCT4, CCT5, CCT6A, CCT7, CCT8, CHTF8, DKC1, RFC2, TCP1, WRAP53] | |
|  | GO:1904871 positive regulation of protein localization to Cajal body | 690.0E-9 | | 27.0E-6 | 3.6E-9 | 22.0E-9 | [4, 5, 6, 7, 8, 9, 10, 11] | 87.50 | | 7.00 | [CCT2, CCT4, CCT5, CCT6A, CCT7, CCT8, TCP1] | |
|  | GO:0007004 telomere maintenance via telomerase | 1.7E-3 | | 11.0E-3 | 3.6E-9 | 22.0E-9 | [7, 8, 9] | 26.83 | | 11.00 | [CCT2, CCT4, CCT5, CCT6A, CCT7, CCT8, DKC1, GNL3L, PINX1, TCP1, WRAP53] | |
|  | GO:0032212 positive regulation of telomere maintenance via telomerase | 3.7E-3 | | 20.0E-3 | 3.6E-9 | 22.0E-9 | [5, 6, 7, 8, 9, 10, 11] | 29.63 | | 8.00 | [CCT2, CCT4, CCT5, CCT6A, CCT7, CCT8, TCP1, WRAP53] | |
|  | GO:1903405 protein localization to nuclear body | 690.0E-9 | | 27.0E-6 | 3.6E-9 | 22.0E-9 | [8] | 87.50 | | 7.00 | [CCT2, CCT4, CCT5, CCT6A, CCT7, CCT8, TCP1] | |
|  | GO:0051186 cofactor metabolic process | 39.0E-6 | | 660.0E-6 | 39.0E-6 | 86.0E-6 | [3] | 21.58 | | 30.00 | [ABCB6, ACLY, ACOT1, ACOT2, ADCK3, ALDOA, BLVRB, CBR1, COQ3, HMOX1, HSCB, MAT2A, ME1, ME2, MLYCD, MTHFD1L, MTHFD2, MTHFS, NADK2, NFS1, OGT, PDHA1, PDHB, PFKFB2, PFKP, PGD, PPOX, PPT1, PRDX5, VCP] | |
|  | GO:0006732 coenzyme metabolic process | 880.0E-6 | | 7.2E-3 | 39.0E-6 | 86.0E-6 | [4] | 20.56 | | 22.00 | [ACLY, ACOT1, ACOT2, ADCK3, ALDOA, COQ3, MAT2A, ME1, ME2, MLYCD, MTHFD1L, MTHFD2, MTHFS, NADK2, OGT, PDHA1, PDHB, PFKFB2, PFKP, PGD, PRDX5, VCP] | |
|  | GO:0006733 oxidoreduction coenzyme metabolic process | 2.5E-3 | | 15.0E-3 | 39.0E-6 | 86.0E-6 | [5] | 24.49 | | 12.00 | [ADCK3, ALDOA, COQ3, ME1, ME2, NADK2, OGT, PFKFB2, PFKP, PGD, PRDX5, VCP] | |
|  | GO:0046496 nicotinamide nucleotide metabolic process | 8.3E-3 | | 37.0E-3 | 39.0E-6 | 86.0E-6 | [6, 7, 8, 9] | 23.26 | | 10.00 | [ALDOA, ME1, ME2, NADK2, OGT, PFKFB2, PFKP, PGD, PRDX5, VCP] | |
|  | GO:0006368 transcription elongation from RNA polymerase II promoter | 260.0E-6 | | 2.7E-3 | 260.0E-6 | 450.0E-6 | [7, 8, 9, 10, 11] | 42.11 | | 8.00 | [BRD4, CDC73, CTR9, RTF1, SETD2, SUPT5H, SUPT6H, WDR61] | |
|  | GO:0051239 regulation of multicellular organismal process | 4.4E-9 | | 400.0E-9 | 4.4E-9 | 24.0E-9 | [2, 3] | 5.20 | | 54.00 | [ABL1, ATP2A2, ATP2B4, BCOR, BRCA1, CD276, CDC73, CHID1, CTR9, DSG2, DSP, EPB41L4B, EPHA3, EZR, FN1, GBA, HMGB2, HTATIP2, JUP, MACF1, MAFB, MAPT, MARK2, MTG1, MTPN, MYADM, MYDGF, NDUFA2, NEDD4L, OGT, PARK7, PFN1, PKP2, PRDX5, PRKD1, PRMT1, PTRH2, RAB11A, RAC1, REST, RHOA, RNMT, RPSA, RTN4, SDCBP, SKI, SLC9A1, SPEN, SYNJ2BP, TRIM27, VAMP7, WARS, WDR61, ZC3HAV1] | |
|  | GO:0051241 negative regulation of multicellular organismal process | 250.0E-6 | | 2.7E-3 | 4.4E-9 | 24.0E-9 | [2, 3, 4] | 4.88 | | 19.00 | [ATP2B4, BCOR, CDC73, CHID1, CTR9, EZR, FN1, GBA, MAFB, PRMT1, RAC1, REST, RHOA, RPSA, RTN4, SKI, SYNJ2BP, TRIM27, WDR61] | |
|  | GO:0001817 regulation of cytokine production | 160.0E-6 | | 1.8E-3 | 4.4E-9 | 24.0E-9 | [3, 4] | 4.11 | | 13.00 | [BRCA1, CD276, CHID1, EZR, FN1, GBA, MYDGF, NDUFA2, PARK7, RAC1, RPSA, TRIM27, ZC3HAV1] | |
|  | GO:2000026 regulation of multicellular organismal development | 14.0E-6 | | 320.0E-6 | 4.4E-9 | 24.0E-9 | [3, 4] | 4.99 | | 28.00 | [ABL1, BCOR, BRCA1, CDC73, CTR9, EPHA3, HMGB2, HTATIP2, MACF1, MAFB, MAPT, MARK2, MYADM, MYDGF, NEDD4L, OGT, PRKD1, PRMT1, PTRH2, REST, RHOA, RTN4, SDCBP, SKI, SPEN, SYNJ2BP, WARS, WDR61] | |
|  | GO:0051276 chromosome organization | 230.0E-6 | | 2.5E-3 | 640.0E-6 | 1.0E-3 | [4] | 17.23 | | 46.00 | [BRD2, CCT2, CCT4, CCT5, CCT6A, CCT7, CCT8, CDC27, CDCA5, CDCA8, CENPE, DDX1, DKC1, ERCC3, GNL3, GNL3L, HIRA, KIF22, KIF2C, MIS12, NABP2, NBN, NCAPD2, NCAPH, NOC2L, NPM1, PDCD6IP, PINX1, PLK1, POGZ, POLR1B, PURA, RAB11A, RPA2, RUVBL2, SMC2, SPTY2D1, STAG2, SUPV3L1, TCP1, TOP1, TOP2A, TP53BP1, TPR, WRAP53, ZW10] | |
|  | GO:0071103 DNA conformation change | 1.1E-3 | | 8.6E-3 | 640.0E-6 | 1.0E-3 | [5] | 21.95 | | 18.00 | [BRD2, CDCA5, DDX1, ERCC3, HIRA, NBN, NCAPD2, NCAPH, NOC2L, NPM1, PURA, SMC2, SPTY2D1, SUPV3L1, TOP1, TOP2A, TP53BP1, TPR] | |
|  | GO:0032204 regulation of telomere maintenance | 6.6E-3 | | 31.0E-3 | 640.0E-6 | 1.0E-3 | [3, 4, 5, 6, 7, 8] | 22.03 | | 13.00 | [CCT2, CCT4, CCT5, CCT6A, CCT7, CCT8, GNL3, GNL3L, NABP2, NBN, PINX1, TCP1, WRAP53] | |
|  | GO:0033044 regulation of chromosome organization | 6.8E-3 | | 32.0E-3 | 640.0E-6 | 1.0E-3 | [5, 6] | 18.63 | | 19.00 | [CCT2, CCT4, CCT5, CCT6A, CCT7, CCT8, CDC27, CDCA5, CENPE, GNL3, GNL3L, NABP2, NBN, PINX1, PLK1, SPTY2D1, TCP1, TPR, WRAP53] | |
|  | GO:0000723 telomere maintenance | 7.5E-3 | | 34.0E-3 | 640.0E-6 | 1.0E-3 | [5, 6, 7] | 19.75 | | 16.00 | [CCT2, CCT4, CCT5, CCT6A, CCT7, CCT8, DKC1, GNL3, GNL3L, NABP2, NBN, PINX1, POLR1B, RPA2, TCP1, WRAP53] | |
|  | GO:0034502 protein localization to chromosome | 4.2E-6 | | 100.0E-6 | 640.0E-6 | 1.0E-3 | [6] | 32.14 | | 18.00 | [CCT2, CCT4, CCT5, CCT6A, CCT7, CCT8, CDCA5, DKC1, GNL3, GNL3L, MMAB, NABP2, PINX1, PLK1, RUVBL2, TCP1, WRAP53, ZW10] | |
|  | GO:2000278 regulation of DNA biosynthetic process | 2.5E-3 | | 15.0E-3 | 640.0E-6 | 1.0E-3 | [5, 6, 7, 8] | 21.62 | | 16.00 | [CCT2, CCT4, CCT5, CCT6A, CCT7, CCT8, CHTF8, DKC1, GNL3L, NABP2, PINX1, RFC2, TCP1, TOM1L1, TP53BP1, WRAP53] | |
|  | GO:0070198 protein localization to chromosome, telomeric region | 150.0E-9 | | 9.2E-6 | 640.0E-6 | 1.0E-3 | [7] | 48.28 | | 14.00 | [CCT2, CCT4, CCT5, CCT6A, CCT7, CCT8, DKC1, GNL3, GNL3L, MMAB, NABP2, PINX1, TCP1, WRAP53] | |
|  | GO:0051503 adenine nucleotide transport | 3.6E-3 | | 20.0E-3 | 3.6E-3 | 4.8E-3 | [7, 8] | 75.00 | | 3.00 | [SLC25A17, SLC25A24, SLC35B2] | |
|  | GO:0051642 centrosome localization | 1.2E-3 | | 9.2E-3 | 870.0E-6 | 1.4E-3 | [4] | 66.67 | | 4.00 | [ASUN, DLG1, EZR, SYNE2] | |
|  | GO:0030953 astral microtubule organization | 8.4E-3 | | 37.0E-3 | 870.0E-6 | 1.4E-3 | [4, 6, 7] | 60.00 | | 3.00 | [DLG1, EZR, RAB11A] | |
|  | GO:0051640 organelle localization | 9.7E-3 | | 41.0E-3 | 9.7E-3 | 10.0E-3 | [3] | 16.56 | | 26.00 | [ACTN4, ASUN, BORCS5, CDCA5, CDCA8, CENPE, CENPF, DLG1, DYNC1H1, EZR, GBF1, KIF22, KIF2C, LAMP1, MAD1L1, MAP4, MFN2, PDCD6IP, PINX1, RAB11A, RHOT1, SYNE2, TOR1A, VAMP7, XPO1, ZW10] | |
|  | GO:0051656 establishment of organelle localization | 1.2E-3 | | 9.4E-3 | 9.7E-3 | 10.0E-3 | [3, 4] | 19.35 | | 24.00 | [ACTN4, BORCS5, CDCA5, CDCA8, CENPE, CENPF, DLG1, DYNC1H1, EZR, GBF1, KIF22, KIF2C, LAMP1, MAD1L1, MAP4, PDCD6IP, PINX1, RAB11A, RHOT1, SYNE2, TOR1A, VAMP7, XPO1, ZW10] | |
|  | GO:0007029 endoplasmic reticulum organization | 1.1E-3 | | 8.5E-3 | 1.1E-3 | 1.7E-3 | [4] | 32.14 | | 9.00 | [ATL2, BAG6, BNIP1, RAB10, RAB18, RAB3GAP2, RTN4, SEC61A1, STX18] | |
|  | GO:0023051 regulation of signaling | 30.0E-6 | | 580.0E-6 | 350.0E-9 | 1.1E-6 | [2, 3] | 7.00 | | 96.00 | [AAK1, ACAA2, ACTN4, ARHGDIA, ARL6IP5, ATP2B4, BAD, BAX, BCAP31, BRCA1, BRD4, BUD31, CD81, CDC37, CDC73, CDK5RAP3, DDRGK1, DLG1, DNAJA3, DNM1L, DUSP3, EEF1D, EGFR, ERBB2, ERBB2IP, ERP29, ETFA, ETFDH, EZR, FLOT2, FN1, GBA, HMOX1, HTRA2, ING3, ITPR1, KDM1A, KMT2D, LAMTOR1, MAP2K2, MFN2, MMAB, MYADM, MYO1C, NDUFS3, NOC2L, NSF, NUP62, OGT, OPA1, PAK2, PARK7, PDCD5, PFDN5, PHB, PHIP, PIAS2, PKN1, POLR2D, PPIF, PRKD1, PRMT5, PTPN2, PTPRJ, RAB3B, RAB7A, REST, RHOA, RHOC, RPL10, RPS3, RPSA, SCRIB, SDCBP, SGSM3, SKI, SLC35B2, SLC9A1, SMARCB1, SNX3, SOD2, SYNJ2BP, TMBIM1, TOR1A, TP53BP1, TRIM25, TSG101, UBE2V1, UFL1, UFM1, USP10, VAMP7, VIMP, WFS1, ZC3HAV1, ZDHHC13] | |
|  | GO:0007154 cell communication | 210.0E-9 | | 10.0E-6 | 350.0E-9 | 1.1E-6 | [3] | 7.14 | | 150.00 | [AAK1, ABHD2, ABL1, ACAA2, ACTN4, AHCYL1, AIMP1, AMFR, ARHGAP1, ARHGDIA, ARL6IP5, ASNS, ATP2A2, ATP2B4, BAD, BAG6, BAX, BCAP31, BRCA1, BRD4, BUD31, CD81, CDC37, CDC5L, CDC73, CDK5RAP3, CTSH, DDRGK1, DERL1, DLG1, DNAJA3, DNM1L, DNMT1, DNPEP, DSG2, DSP, DUSP3, EEF1D, EFNA5, EGFR, EHMT2, EPHA3, ERBB2, ERBB2IP, ERP29, ETFA, ETFDH, EZR, FARP2, FASN, FLOT2, FN1, GARS, GBA, GNAI3, GRB2, HMOX1, HTRA2, ING3, ITGA2, ITPR1, JAGN1, JAK3, JUN, JUP, KDM1A, KMT2D, KRT17, LAMP1, LAMTOR1, LARP1, MAP2K2, MARK2, MFN2, MMAB, MYADM, MYH14, MYO1C, NDUFS3, NOC2L, NUP62, OGT, OPA1, PAK2, PARK7, PDCD5, PFDN5, PHB, PHIP, PIAS2, PKN1, PKP2, POLR2D, PPIF, PRKD1, PRMT5, PRPF19, PTPN11, PTPN2, PTPRJ, RAB3B, RAB7A, RAP1A, RBFOX2, REPS1, REST, RHOA, RHOC, RHOT1, RNMT, RPL10, RPS27L, RPS3, RPS6, RPSA, SDCBP, SENP1, SFN, SGSM3, SKI, SLC33A1, SLC35B2, SLC9A1, SMARCB1, SNX3, SOD2, SPG7, STAT3, SYNJ2BP, TAX1BP3, TFPT, THEM4, TMBIM1, TMCO1, TOM1L1, TOM1L2, TOR1A, TP53BP1, TPR, TRIM25, TSG101, UBE2V1, UFL1, UFM1, USP10, VAMP7, VIMP, WFS1, ZC3HAV1, ZDHHC13] | |
|  | GO:0007165 signal transduction | 110.0E-9 | | 7.1E-6 | 350.0E-9 | 1.1E-6 | [3, 4] | 6.94 | | 137.00 | [AAK1, ABHD2, ABL1, ACAA2, ACTN4, AHCYL1, AMFR, ARHGAP1, ARHGDIA, ARL6IP5, ATP2A2, ATP2B4, BAD, BAG6, BAX, BCAP31, BRCA1, BRD4, BUD31, CD81, CDC37, CDC5L, CDC73, CDK5RAP3, CTSH, DDRGK1, DERL1, DLG1, DNAJA3, DNM1L, DNMT1, DNPEP, DUSP3, EEF1D, EFNA5, EGFR, EPHA3, ERBB2, ERBB2IP, ERP29, ETFA, ETFDH, EZR, FARP2, FASN, FLOT2, FN1, GARS, GBA, GNAI3, GRB2, HMOX1, HTRA2, ING3, ITGA2, ITPR1, JAGN1, JAK3, JUN, KDM1A, KMT2D, KRT17, LAMP1, LAMTOR1, LARP1, MAP2K2, MARK2, MFN2, MMAB, MYADM, MYO1C, NDUFS3, NOC2L, NUP62, OGT, OPA1, PAK2, PARK7, PDCD5, PFDN5, PHB, PHIP, PIAS2, PKN1, PPIF, PRKD1, PRMT5, PRPF19, PTPN11, PTPN2, PTPRJ, RAB7A, RAP1A, RBFOX2, REPS1, RHOA, RHOC, RHOT1, RNMT, RPL10, RPS27L, RPS3, RPS6, RPSA, SDCBP, SENP1, SFN, SGSM3, SKI, SLC33A1, SLC35B2, SLC9A1, SMARCB1, SNX3, SOD2, SPG7, STAT3, SYNJ2BP, TAX1BP3, TFPT, THEM4, TMBIM1, TMCO1, TOM1L1, TOM1L2, TP53BP1, TPR, TRIM25, TSG101, UBE2V1, UFL1, UFM1, USP10, VIMP, WFS1, ZC3HAV1, ZDHHC13] | |
|  | GO:0010646 regulation of cell communication | 27.0E-6 | | 550.0E-6 | 350.0E-9 | 1.1E-6 | [3, 4] | 6.94 | | 94.00 | [AAK1, ACAA2, ACTN4, ARHGDIA, ARL6IP5, ATP2B4, BAD, BAX, BCAP31, BRCA1, BRD4, BUD31, CD81, CDC37, CDC73, CDK5RAP3, DDRGK1, DLG1, DNAJA3, DNM1L, DUSP3, EEF1D, EGFR, ERBB2, ERBB2IP, ERP29, ETFA, ETFDH, EZR, FLOT2, FN1, GBA, HMOX1, HTRA2, ING3, ITPR1, KDM1A, KMT2D, LAMTOR1, MAP2K2, MFN2, MMAB, MYADM, MYO1C, NDUFS3, NOC2L, NUP62, OGT, OPA1, PAK2, PARK7, PDCD5, PFDN5, PHB, PHIP, PIAS2, PKN1, POLR2D, PPIF, PRKD1, PRMT5, PTPN2, PTPRJ, RAB3B, RAB7A, REST, RHOA, RHOC, RPL10, RPS3, RPSA, SDCBP, SGSM3, SKI, SLC35B2, SLC9A1, SMARCB1, SNX3, SOD2, SYNJ2BP, TMBIM1, TOR1A, TP53BP1, TRIM25, TSG101, UBE2V1, UFL1, UFM1, USP10, VAMP7, VIMP, WFS1, ZC3HAV1, ZDHHC13] | |
|  | GO:0009966 regulation of signal transduction | 37.0E-6 | | 650.0E-6 | 350.0E-9 | 1.1E-6 | [3, 4, 5] | 6.90 | | 89.00 | [AAK1, ACAA2, ACTN4, ARHGDIA, ARL6IP5, ATP2B4, BAD, BAX, BCAP31, BRCA1, BRD4, BUD31, CD81, CDC37, CDC73, CDK5RAP3, DDRGK1, DLG1, DNAJA3, DNM1L, DUSP3, EEF1D, EGFR, ERBB2, ERBB2IP, ERP29, ETFA, ETFDH, EZR, FLOT2, FN1, GBA, HMOX1, HTRA2, ING3, ITPR1, KDM1A, KMT2D, LAMTOR1, MAP2K2, MFN2, MMAB, MYADM, MYO1C, NDUFS3, NOC2L, NUP62, OGT, OPA1, PAK2, PARK7, PDCD5, PFDN5, PHB, PHIP, PIAS2, PKN1, PPIF, PRKD1, PRMT5, PTPN2, PTPRJ, RAB7A, RHOA, RHOC, RPL10, RPS3, RPSA, SDCBP, SGSM3, SKI, SLC35B2, SLC9A1, SMARCB1, SNX3, SOD2, SYNJ2BP, TMBIM1, TP53BP1, TRIM25, TSG101, UBE2V1, UFL1, UFM1, USP10, VIMP, WFS1, ZC3HAV1, ZDHHC13] | |
|  | GO:0007166 cell surface receptor signaling pathway | 550.0E-9 | | 22.0E-6 | 350.0E-9 | 1.1E-6 | [4, 5] | 5.57 | | 51.00 | [AAK1, ABL1, BAD, BAX, BRCA1, CDC37, CDC73, DNAJA3, DUSP3, EFNA5, EGFR, EPHA3, ERBB2, ETFA, ETFDH, EZR, FASN, GARS, GRB2, HMOX1, HTRA2, ING3, ITGA2, JAGN1, JAK3, JUN, MYO1C, NUP62, OGT, PAK2, PARK7, PFDN5, PHIP, PRKD1, PTPN11, PTPN2, PTPRJ, RAB7A, RHOA, RNMT, RPL10, RPSA, SDCBP, SKI, SLC33A1, SNX3, SPG7, STAT3, SYNJ2BP, TMBIM1, TSG101] | |
|  | GO:0007167 enzyme linked receptor protein signaling pathway | 6.5E-3 | | 31.0E-3 | 350.0E-9 | 1.1E-6 | [5, 6] | 5.87 | | 21.00 | [ABL1, EFNA5, EGFR, EPHA3, ERBB2, GARS, GRB2, ING3, JUN, MYO1C, OGT, PRKD1, PTPN11, PTPN2, PTPRJ, RAB7A, SDCBP, SKI, SLC33A1, STAT3, TSG101] | |
|  | GO:0070848 response to growth factor | 1.1E-3 | | 8.7E-3 | 300.0E-9 | 1.0E-6 | [4] | 4.58 | | 13.00 | [EEF1A1, ERBB2, GARS, HTRA2, ING3, JUN, MYO1C, PDCD5, PRKD1, SDCBP, SKI, SLC33A1, TPR] | |
|  | GO:0007166 cell surface receptor signaling pathway | 550.0E-9 | | 22.0E-6 | 300.0E-9 | 1.0E-6 | [4, 5] | 5.57 | | 51.00 | [AAK1, ABL1, BAD, BAX, BRCA1, CDC37, CDC73, DNAJA3, DUSP3, EFNA5, EGFR, EPHA3, ERBB2, ETFA, ETFDH, EZR, FASN, GARS, GRB2, HMOX1, HTRA2, ING3, ITGA2, JAGN1, JAK3, JUN, MYO1C, NUP62, OGT, PAK2, PARK7, PFDN5, PHIP, PRKD1, PTPN11, PTPN2, PTPRJ, RAB7A, RHOA, RNMT, RPL10, RPSA, SDCBP, SKI, SLC33A1, SNX3, SPG7, STAT3, SYNJ2BP, TMBIM1, TSG101] | |
|  | GO:0007167 enzyme linked receptor protein signaling pathway | 6.5E-3 | | 31.0E-3 | 300.0E-9 | 1.0E-6 | [5, 6] | 5.87 | | 21.00 | [ABL1, EFNA5, EGFR, EPHA3, ERBB2, GARS, GRB2, ING3, JUN, MYO1C, OGT, PRKD1, PTPN11, PTPN2, PTPRJ, RAB7A, SDCBP, SKI, SLC33A1, STAT3, TSG101] | |
|  | GO:0007178 transmembrane receptor protein serine/threonine kinase signaling pathway | 12.0E-3 | | 49.0E-3 | 300.0E-9 | 1.0E-6 | [6, 7] | 4.00 | | 6.00 | [GARS, ING3, JUN, SDCBP, SKI, SLC33A1] | |
|  | GO:0009888 tissue development | 3.8E-3 | | 20.0E-3 | 3.8E-3 | 4.9E-3 | [3] | 6.40 | | 33.00 | [ACADVL, ALDH3A2, ALDOC, ANXA7, BCCIP, BCOR, CBR1, DSG2, DSP, EHF, ERCC3, EZR, FN1, GSTK1, ITGA2, LAMA3, LEMD2, MPP5, MYADM, MYH14, PGK1, PKN1, PKP2, PLOD1, PTRH2, RAP1A, RHOA, RNMT, RPL10, SCRIB, SDCBP, SETSIP, TPP1] | |
|  | GO:0007163 establishment or maintenance of cell polarity | 5.5E-3 | | 27.0E-3 | 5.5E-3 | 6.7E-3 | [3] | 23.40 | | 11.00 | [CKAP5, EZR, FLOT2, GBF1, KIF2C, MAP4, MARK2, MYO18A, SCRIB, WDR1, ZW10] | |
|  | GO:0035088 establishment or maintenance of apical/basal cell polarity | 4.9E-3 | | 25.0E-3 | 5.5E-3 | 6.7E-3 | [5] | 50.00 | | 4.00 | [EZR, MARK2, SCRIB, WDR1] | |
|  | GO:0010256 endomembrane system organization | 60.0E-6 | | 910.0E-6 | 1.3E-6 | 3.6E-6 | [3] | 17.41 | | 51.00 | [ANKLE2, ASUN, ATL2, ATP1B1, ATP1B3, BAG6, BNIP1, CDH1, COG2, COG3, COG4, DLG1, DNM2, EZR, FLOT2, GBF1, GCC2, GOLGA5, GOLPH3L, HOOK1, JUP, LEMD2, LMAN1, MACF1, MYADM, MYO18A, PKP2, PKP3, PPFIA1, PPIL2, PRKD1, PRMT5, RAB10, RAB11A, RAB18, RAB2A, RAB3GAP2, RTN4, SEC61A1, SNX3, STX18, STX3, STX8, STXBP1, SYNE1, TMBIM1, TMED5, VRK1, VTI1A, VTI1B, ZW10] | |
|  | GO:0061024 membrane organization | 200.0E-6 | | 2.3E-3 | 1.3E-6 | 3.6E-6 | [3] | 15.79 | | 63.00 | [ACAA2, ANKLE2, ASUN, ATP1B1, ATP1B3, BAD, BAG6, BAX, BNIP1, CD9, CDH1, DLG1, DNM1L, DNM2, ERBB2, EZR, FLOT2, GBF1, GCC2, GDAP1, IMMT, JUP, LEMD2, MACF1, MFN1, MFN2, MYADM, MYO18A, MYO1C, OPA1, PDCD5, PDCD6IP, PEX16, PKP2, PKP3, PPFIA1, PPIF, PPIL2, PPT1, RAB10, RAB11A, RAB11B, RAB3GAP2, RAB7A, RHOA, RHOT1, SAMM50, SEC61A1, SEC62, SEC63, SNX3, STX18, STX3, STX8, STXBP1, SYNE3, THEM4, TIMM10, TMBIM1, TSG101, VAMP7, VTA1, VTI1B] | |
|  | GO:0044802 single-organism membrane organization | 520.0E-6 | | 4.9E-3 | 1.3E-6 | 3.6E-6 | [3, 4] | 15.77 | | 56.00 | [ACAA2, ANKLE2, ASUN, ATP1B1, ATP1B3, BAD, BAG6, BAX, BNIP1, CD9, CDH1, DLG1, DNM1L, DNM2, ERBB2, EZR, FLOT2, GCC2, GDAP1, IMMT, JUP, LEMD2, MACF1, MFN1, MFN2, MYADM, MYO1C, OPA1, PDCD5, PEX16, PKP2, PKP3, PPFIA1, PPIF, PPIL2, PPT1, RAB10, RAB11A, RAB11B, RAB3GAP2, RAB7A, RHOT1, SAMM50, SEC61A1, SEC62, SEC63, STX18, STX3, STX8, STXBP1, SYNE3, THEM4, TIMM10, TMBIM1, VAMP7, VTI1B] | |
|  | GO:0030433 ER-associated ubiquitin-dependent protein catabolic process | 11.0E-3 | | 46.0E-3 | 13.0E-6 | 31.0E-6 | [4, 5, 6, 7, 8, 9, 10, 11] | 22.22 | | 10.00 | [AMFR, BCAP31, DERL1, KIAA0368, MAN1B1, OS9, TMUB1, VCP, VIMP, WFS1] | |
|  | GO:0042127 regulation of cell proliferation | 5.4E-3 | | 27.0E-3 | 5.4E-3 | 6.8E-3 | [3, 4] | 6.95 | | 46.00 | [AIMP1, ARID2, ATP5A1, BAD, BRD7, CD276, CD81, CDC73, CEP131, CHERP, DNAJA3, EGFR, EIF5A, EPB41L4B, ERBB2, FN1, GDF15, GTPBP4, HMGB2, HMOX1, JUP, KIF20B, KMT2D, MYDGF, NACC1, NPM1, NUP62, PBRM1, PDCD5, PHB, PKN1, PRKD1, PTPN2, PTPRJ, REST, RPL10, RPRD1B, RPS15A, RPS4X, SDCBP, SKI, SOD2, STX3, SYNJ2BP, TP53BP1, UFL1] | |
|  | GO:0008284 positive regulation of cell proliferation | 8.9E-3 | | 39.0E-3 | 5.4E-3 | 6.8E-3 | [3, 4, 5] | 6.19 | | 24.00 | [BAD, CD276, CD81, CEP131, EGFR, EIF5A, ERBB2, FN1, GDF15, HMGB2, HMOX1, KIF20B, KMT2D, MYDGF, NACC1, NPM1, PKN1, PRKD1, RPRD1B, RPS15A, RPS4X, SDCBP, STX3, UFL1] | |
|  | GO:0023051 regulation of signaling | 30.0E-6 | | 580.0E-6 | 140.0E-9 | 570.0E-9 | [2, 3] | 7.00 | | 96.00 | [AAK1, ACAA2, ACTN4, ARHGDIA, ARL6IP5, ATP2B4, BAD, BAX, BCAP31, BRCA1, BRD4, BUD31, CD81, CDC37, CDC73, CDK5RAP3, DDRGK1, DLG1, DNAJA3, DNM1L, DUSP3, EEF1D, EGFR, ERBB2, ERBB2IP, ERP29, ETFA, ETFDH, EZR, FLOT2, FN1, GBA, HMOX1, HTRA2, ING3, ITPR1, KDM1A, KMT2D, LAMTOR1, MAP2K2, MFN2, MMAB, MYADM, MYO1C, NDUFS3, NOC2L, NSF, NUP62, OGT, OPA1, PAK2, PARK7, PDCD5, PFDN5, PHB, PHIP, PIAS2, PKN1, POLR2D, PPIF, PRKD1, PRMT5, PTPN2, PTPRJ, RAB3B, RAB7A, REST, RHOA, RHOC, RPL10, RPS3, RPSA, SCRIB, SDCBP, SGSM3, SKI, SLC35B2, SLC9A1, SMARCB1, SNX3, SOD2, SYNJ2BP, TMBIM1, TOR1A, TP53BP1, TRIM25, TSG101, UBE2V1, UFL1, UFM1, USP10, VAMP7, VIMP, WFS1, ZC3HAV1, ZDHHC13] | |
|  | GO:0048583 regulation of response to stimulus | 3.9E-6 | | 100.0E-6 | 140.0E-9 | 570.0E-9 | [2, 3] | 7.13 | | 125.00 | [AAK1, ABL1, ACAA2, ACTN4, ALKBH5, ANXA5, ARHGDIA, ARL6IP5, ATP1B1, ATP2B4, BAD, BAG6, BAX, BCAP31, BRCA1, BRD4, BUD31, CD81, CDC37, CDC73, CDK5RAP3, CHID1, CTSH, DDRGK1, DLG1, DNAJA3, DNAJC3, DNM1L, DUSP3, EEF1D, EGFR, ELMOD2, ERBB2, ERBB2IP, ERP29, ETFA, ETFDH, EZR, FLOT2, FN1, GBA, HK2, HMOX1, HTRA2, ING3, ITPR1, KDM1A, KMT2D, LAMP1, LAMTOR1, MAP2K2, MDH1, MFN2, MMAB, MRPS10, MTPN, MYADM, MYDGF, MYO1C, NDUFS3, NOC2L, NPM1, NUP62, OGT, OPA1, OS9, PAK2, PARK7, PDCD5, PEX13, PFDN5, PHB, PHIP, PIAS2, PKN1, PNPT1, POLR1B, PPIF, PRKD1, PRMT5, PTPN11, PTPN2, PTPRJ, PYCR1, RAB7A, RAC1, RAC2, RHOA, RHOC, RNMT, RPA2, RPL10, RPS3, RPSA, SDCBP, SGSM3, SIN3A, SKI, SLC35B2, SLC9A1, SMARCB1, SNRPB, SNX3, SOD2, STK26, STX3, SYNJ2BP, TAF9, TMBIM1, TP53BP1, TRIM25, TRIM27, TSG101, UBAC2, UBE2V1, UBXN1, UFL1, UFM1, USP10, VAMP7, VIMP, WFS1, ZC3HAV1, ZCCHC17, ZDHHC13] | |
|  | GO:0007154 cell communication | 210.0E-9 | | 10.0E-6 | 140.0E-9 | 570.0E-9 | [3] | 7.14 | | 150.00 | [AAK1, ABHD2, ABL1, ACAA2, ACTN4, AHCYL1, AIMP1, AMFR, ARHGAP1, ARHGDIA, ARL6IP5, ASNS, ATP2A2, ATP2B4, BAD, BAG6, BAX, BCAP31, BRCA1, BRD4, BUD31, CD81, CDC37, CDC5L, CDC73, CDK5RAP3, CTSH, DDRGK1, DERL1, DLG1, DNAJA3, DNM1L, DNMT1, DNPEP, DSG2, DSP, DUSP3, EEF1D, EFNA5, EGFR, EHMT2, EPHA3, ERBB2, ERBB2IP, ERP29, ETFA, ETFDH, EZR, FARP2, FASN, FLOT2, FN1, GARS, GBA, GNAI3, GRB2, HMOX1, HTRA2, ING3, ITGA2, ITPR1, JAGN1, JAK3, JUN, JUP, KDM1A, KMT2D, KRT17, LAMP1, LAMTOR1, LARP1, MAP2K2, MARK2, MFN2, MMAB, MYADM, MYH14, MYO1C, NDUFS3, NOC2L, NUP62, OGT, OPA1, PAK2, PARK7, PDCD5, PFDN5, PHB, PHIP, PIAS2, PKN1, PKP2, POLR2D, PPIF, PRKD1, PRMT5, PRPF19, PTPN11, PTPN2, PTPRJ, RAB3B, RAB7A, RAP1A, RBFOX2, REPS1, REST, RHOA, RHOC, RHOT1, RNMT, RPL10, RPS27L, RPS3, RPS6, RPSA, SDCBP, SENP1, SFN, SGSM3, SKI, SLC33A1, SLC35B2, SLC9A1, SMARCB1, SNX3, SOD2, SPG7, STAT3, SYNJ2BP, TAX1BP3, TFPT, THEM4, TMBIM1, TMCO1, TOM1L1, TOM1L2, TOR1A, TP53BP1, TPR, TRIM25, TSG101, UBE2V1, UFL1, UFM1, USP10, VAMP7, VIMP, WFS1, ZC3HAV1, ZDHHC13] | |
|  | GO:0023056 positive regulation of signaling | 3.1E-3 | | 18.0E-3 | 140.0E-9 | 570.0E-9 | [2, 3, 4] | 7.02 | | 55.00 | [AAK1, ACTN4, ARL6IP5, BAD, BAX, BCAP31, BRD4, BUD31, CD81, CDC73, CDK5RAP3, DDRGK1, DNM1L, EEF1D, EGFR, ERBB2, ERP29, ETFA, ETFDH, HMOX1, HTRA2, ING3, KMT2D, LAMTOR1, MAP2K2, MMAB, MYO1C, NSF, NUP62, PAK2, PARK7, PDCD5, PHB, PKN1, POLR2D, PRKD1, PRMT5, PTPRJ, RAB3B, RHOA, RHOC, RPL10, RPS3, RPSA, SCRIB, SDCBP, SLC35B2, SLC9A1, SMARCB1, TP53BP1, TRIM25, UBE2V1, VAMP7, ZC3HAV1, ZDHHC13] | |
|  | GO:0023057 negative regulation of signaling | 1.5E-3 | | 10.0E-3 | 140.0E-9 | 570.0E-9 | [2, 3, 4] | 6.30 | | 37.00 | [ACAA2, ATP2B4, BRCA1, CDK5RAP3, DLG1, DNAJA3, DUSP3, ERBB2IP, EZR, GBA, HMOX1, ITPR1, KDM1A, MFN2, MYADM, NDUFS3, NOC2L, OPA1, PARK7, PFDN5, PHB, PHIP, PIAS2, PPIF, PTPN2, PTPRJ, RAB7A, REST, RPL10, SKI, SOD2, SYNJ2BP, TMBIM1, TSG101, USP10, VIMP, WFS1] | |
|  | GO:0048584 positive regulation of response to stimulus | 130.0E-6 | | 1.7E-3 | 140.0E-9 | 570.0E-9 | [2, 3, 4] | 6.69 | | 66.00 | [AAK1, ACTN4, ARL6IP5, BAD, BAG6, BAX, BCAP31, BRCA1, BRD4, BUD31, CD81, CDC73, CDK5RAP3, DDRGK1, DNM1L, DUSP3, EEF1D, EGFR, ERBB2, ERBB2IP, ERP29, ETFA, ETFDH, EZR, FLOT2, HK2, HMOX1, HTRA2, ING3, KMT2D, LAMP1, LAMTOR1, MAP2K2, MMAB, MYO1C, NUP62, PAK2, PARK7, PDCD5, PHB, PKN1, PRKD1, PRMT5, PTPN11, PTPRJ, RAC1, RAC2, RHOA, RHOC, RNMT, RPL10, RPS3, RPSA, SDCBP, SIN3A, SLC35B2, SLC9A1, SMARCB1, STX3, TAF9, TP53BP1, TRIM25, UBE2V1, VAMP7, ZC3HAV1, ZDHHC13] | |
|  | GO:0048585 negative regulation of response to stimulus | 960.0E-6 | | 7.7E-3 | 140.0E-9 | 570.0E-9 | [2, 3, 4] | 6.52 | | 46.00 | [ACAA2, ATP2B4, BRCA1, BRD4, CDK5RAP3, CHID1, DLG1, DNAJA3, DNAJC3, DUSP3, ERBB2IP, EZR, GBA, HMOX1, ITPR1, KDM1A, MFN2, MYADM, NDUFS3, NOC2L, OPA1, OS9, PARK7, PFDN5, PHB, PHIP, PIAS2, PPIF, PTPN2, PTPRJ, PYCR1, RAB7A, RNMT, RPL10, RPS3, SKI, SOD2, SYNJ2BP, TMBIM1, TRIM27, TSG101, UBAC2, UBXN1, USP10, VIMP, WFS1] | |
|  | GO:0070887 cellular response to chemical stimulus | 10.0E-3 | | 44.0E-3 | 140.0E-9 | 570.0E-9 | [3] | 7.91 | | 91.00 | [ABHD2, ABL1, ACAA2, ADAR, AHCYL1, AMFR, ARL6IP5, ATP2B4, BAD, BAX, BRCA1, BUD31, CDC37, CDH1, CDK5RAP3, CHD6, CTSH, DDRGK1, DERL1, DNAJA3, EEF1A1, EGFR, EPHA3, ERBB2, ERBB2IP, EZR, FASN, GARS, GBA, GBF1, GOT1, GRB2, HMGB2, HMOX1, HTRA2, ING3, JAGN1, JAK3, JUN, JUP, KMT2D, LAMTOR1, MAP2K2, MGST1, MYO1C, NDUFA2, NPEPPS, NPM1, OGT, PARK7, PDCD5, PELP1, PHB, PIAS2, PKN1, PNPT1, PPIF, PRDX5, PRKD1, PTPN11, PTPN2, PYCR1, PYCR2, RAC1, RAC2, RAP1A, RBFOX2, REST, RNMT, RPL10, RPS3, RPSA, RUVBL2, SDCBP, SKI, SLC25A24, SLC33A1, SLC9A1, SMARCB1, SNIP1, SOD2, STAT3, STK26, TP53BP1, TRIM24, UFL1, UFM1, USP10, VCP, VIMP, WFS1] | |
|  | GO:0007165 signal transduction | 110.0E-9 | | 7.1E-6 | 140.0E-9 | 570.0E-9 | [3, 4] | 6.94 | | 137.00 | [AAK1, ABHD2, ABL1, ACAA2, ACTN4, AHCYL1, AMFR, ARHGAP1, ARHGDIA, ARL6IP5, ATP2A2, ATP2B4, BAD, BAG6, BAX, BCAP31, BRCA1, BRD4, BUD31, CD81, CDC37, CDC5L, CDC73, CDK5RAP3, CTSH, DDRGK1, DERL1, DLG1, DNAJA3, DNM1L, DNMT1, DNPEP, DUSP3, EEF1D, EFNA5, EGFR, EPHA3, ERBB2, ERBB2IP, ERP29, ETFA, ETFDH, EZR, FARP2, FASN, FLOT2, FN1, GARS, GBA, GNAI3, GRB2, HMOX1, HTRA2, ING3, ITGA2, ITPR1, JAGN1, JAK3, JUN, KDM1A, KMT2D, KRT17, LAMP1, LAMTOR1, LARP1, MAP2K2, MARK2, MFN2, MMAB, MYADM, MYO1C, NDUFS3, NOC2L, NUP62, OGT, OPA1, PAK2, PARK7, PDCD5, PFDN5, PHB, PHIP, PIAS2, PKN1, PPIF, PRKD1, PRMT5, PRPF19, PTPN11, PTPN2, PTPRJ, RAB7A, RAP1A, RBFOX2, REPS1, RHOA, RHOC, RHOT1, RNMT, RPL10, RPS27L, RPS3, RPS6, RPSA, SDCBP, SENP1, SFN, SGSM3, SKI, SLC33A1, SLC35B2, SLC9A1, SMARCB1, SNX3, SOD2, SPG7, STAT3, SYNJ2BP, TAX1BP3, TFPT, THEM4, TMBIM1, TMCO1, TOM1L1, TOM1L2, TP53BP1, TPR, TRIM25, TSG101, UBE2V1, UFL1, UFM1, USP10, VIMP, WFS1, ZC3HAV1, ZDHHC13] | |
|  | GO:0010646 regulation of cell communication | 27.0E-6 | | 550.0E-6 | 140.0E-9 | 570.0E-9 | [3, 4] | 6.94 | | 94.00 | [AAK1, ACAA2, ACTN4, ARHGDIA, ARL6IP5, ATP2B4, BAD, BAX, BCAP31, BRCA1, BRD4, BUD31, CD81, CDC37, CDC73, CDK5RAP3, DDRGK1, DLG1, DNAJA3, DNM1L, DUSP3, EEF1D, EGFR, ERBB2, ERBB2IP, ERP29, ETFA, ETFDH, EZR, FLOT2, FN1, GBA, HMOX1, HTRA2, ING3, ITPR1, KDM1A, KMT2D, LAMTOR1, MAP2K2, MFN2, MMAB, MYADM, MYO1C, NDUFS3, NOC2L, NUP62, OGT, OPA1, PAK2, PARK7, PDCD5, PFDN5, PHB, PHIP, PIAS2, PKN1, POLR2D, PPIF, PRKD1, PRMT5, PTPN2, PTPRJ, RAB3B, RAB7A, REST, RHOA, RHOC, RPL10, RPS3, RPSA, SDCBP, SGSM3, SKI, SLC35B2, SLC9A1, SMARCB1, SNX3, SOD2, SYNJ2BP, TMBIM1, TOR1A, TP53BP1, TRIM25, TSG101, UBE2V1, UFL1, UFM1, USP10, VAMP7, VIMP, WFS1, ZC3HAV1, ZDHHC13] | |
|  | GO:0006796 phosphate-containing compound metabolic process | 1.4E-3 | | 10.0E-3 | 140.0E-9 | 570.0E-9 | [4] | 7.71 | | 109.00 | [AAK1, ABHD12, ABL1, ACP6, ADAR, ADCK3, AK1, AK3, ALDOA, ALDOC, ANKLE2, ARL6IP5, ATP1B1, ATP2B4, ATP5B, ATP5O, BARD1, BAZ1B, BCCIP, BRD4, CCNT1, CD81, CDK11A, CDK11B, CDK5RAP3, CENPE, COA6, CREB1, CTSH, DDRGK1, DLG1, DNAJA3, DNAJC3, DPM1, DUSP3, EEF1A2, EGFR, EPHA4, EPT1, ERBB2, ERP29, ETFA, ETFDH, EZR, FN1, GARS, GBA, GK, GNAI3, GTPBP4, GUK1, HSPA8, MAP2K2, MARK2, MCM7, ME1, ME2, MMAB, MPHOSPH10, MYADM, MYO1D, NADK2, NBN, NDUFS1, NPM1, NSD1, OGT, OLA1, PAK2, PARK7, PFKFB2, PFKP, PGD, PHB, PKN1, PLK1, PLPP6, PNPLA8, PPP2R2A, PPP2R4, PRDX5, PRKACB, PRKAR2B, PRKD1, PTPMT1, PTPN11, PTPN2, PTPRJ, PTRH2, RAC1, RHOA, RPRD1B, RPS3, RRP1B, SDCBP, SLC27A1, SLK, SMPDL3A, STK26, SURF1, SYMPK, SYNJ2BP, TOM1L1, TPR, TRIM27, TSG101, VCP, VRK1, YWHAE] | |
|  | GO:0009966 regulation of signal transduction | 37.0E-6 | | 650.0E-6 | 140.0E-9 | 570.0E-9 | [3, 4, 5] | 6.90 | | 89.00 | [AAK1, ACAA2, ACTN4, ARHGDIA, ARL6IP5, ATP2B4, BAD, BAX, BCAP31, BRCA1, BRD4, BUD31, CD81, CDC37, CDC73, CDK5RAP3, DDRGK1, DLG1, DNAJA3, DNM1L, DUSP3, EEF1D, EGFR, ERBB2, ERBB2IP, ERP29, ETFA, ETFDH, EZR, FLOT2, FN1, GBA, HMOX1, HTRA2, ING3, ITPR1, KDM1A, KMT2D, LAMTOR1, MAP2K2, MFN2, MMAB, MYADM, MYO1C, NDUFS3, NOC2L, NUP62, OGT, OPA1, PAK2, PARK7, PDCD5, PFDN5, PHB, PHIP, PIAS2, PKN1, PPIF, PRKD1, PRMT5, PTPN2, PTPRJ, RAB7A, RHOA, RHOC, RPL10, RPS3, RPSA, SDCBP, SGSM3, SKI, SLC35B2, SLC9A1, SMARCB1, SNX3, SOD2, SYNJ2BP, TMBIM1, TP53BP1, TRIM25, TSG101, UBE2V1, UFL1, UFM1, USP10, VIMP, WFS1, ZC3HAV1, ZDHHC13] | |
|  | GO:0010647 positive regulation of cell communication | 1.5E-3 | | 10.0E-3 | 140.0E-9 | 570.0E-9 | [3, 4, 5] | 6.82 | | 53.00 | [AAK1, ACTN4, ARL6IP5, BAD, BAX, BCAP31, BRD4, BUD31, CD81, CDC73, CDK5RAP3, DDRGK1, DNM1L, EEF1D, EGFR, ERBB2, ERP29, ETFA, ETFDH, HMOX1, HTRA2, ING3, KMT2D, LAMTOR1, MAP2K2, MMAB, MYO1C, NUP62, PAK2, PARK7, PDCD5, PHB, PKN1, POLR2D, PRKD1, PRMT5, PTPRJ, RAB3B, RHOA, RHOC, RPL10, RPS3, RPSA, SDCBP, SLC35B2, SLC9A1, SMARCB1, TP53BP1, TRIM25, UBE2V1, VAMP7, ZC3HAV1, ZDHHC13] | |
|  | GO:0010648 negative regulation of cell communication | 1.9E-3 | | 12.0E-3 | 140.0E-9 | 570.0E-9 | [3, 4, 5] | 6.36 | | 37.00 | [ACAA2, ATP2B4, BRCA1, CDK5RAP3, DLG1, DNAJA3, DUSP3, ERBB2IP, EZR, GBA, HMOX1, ITPR1, KDM1A, MFN2, MYADM, NDUFS3, NOC2L, OPA1, PARK7, PFDN5, PHB, PHIP, PIAS2, PPIF, PTPN2, PTPRJ, RAB7A, REST, RPL10, SKI, SOD2, SYNJ2BP, TMBIM1, TSG101, USP10, VIMP, WFS1] | |
|  | GO:0007166 cell surface receptor signaling pathway | 550.0E-9 | | 22.0E-6 | 140.0E-9 | 570.0E-9 | [4, 5] | 5.57 | | 51.00 | [AAK1, ABL1, BAD, BAX, BRCA1, CDC37, CDC73, DNAJA3, DUSP3, EFNA5, EGFR, EPHA3, ERBB2, ETFA, ETFDH, EZR, FASN, GARS, GRB2, HMOX1, HTRA2, ING3, ITGA2, JAGN1, JAK3, JUN, MYO1C, NUP62, OGT, PAK2, PARK7, PFDN5, PHIP, PRKD1, PTPN11, PTPN2, PTPRJ, RAB7A, RHOA, RNMT, RPL10, RPSA, SDCBP, SKI, SLC33A1, SNX3, SPG7, STAT3, SYNJ2BP, TMBIM1, TSG101] | |
|  | GO:0009967 positive regulation of signal transduction | 1.3E-3 | | 9.4E-3 | 140.0E-9 | 570.0E-9 | [3, 4, 5, 6] | 6.69 | | 50.00 | [AAK1, ACTN4, ARL6IP5, BAD, BAX, BCAP31, BRD4, BUD31, CD81, CDC73, CDK5RAP3, DDRGK1, DNM1L, EEF1D, EGFR, ERBB2, ERP29, ETFA, ETFDH, HMOX1, HTRA2, ING3, KMT2D, LAMTOR1, MAP2K2, MMAB, MYO1C, NUP62, PAK2, PARK7, PDCD5, PHB, PKN1, PRKD1, PRMT5, PTPRJ, RHOA, RHOC, RPL10, RPS3, RPSA, SDCBP, SLC35B2, SLC9A1, SMARCB1, TP53BP1, TRIM25, UBE2V1, ZC3HAV1, ZDHHC13] | |
|  | GO:0035556 intracellular signal transduction | 6.9E-3 | | 32.0E-3 | 140.0E-9 | 570.0E-9 | [4, 5] | 7.73 | | 86.00 | [ABL1, ACTN4, ARHGAP1, ARHGDIA, ARL6IP5, ATP2A2, ATP2B4, BAD, BAG6, BAX, BCAP31, BRCA1, BRD4, CD81, CDC5L, CDK5RAP3, CTSH, DDRGK1, DLG1, DNAJA3, DNM1L, DNMT1, DUSP3, EEF1D, EGFR, ERBB2, ERBB2IP, ERP29, ETFA, ETFDH, EZR, FARP2, FN1, GBA, GRB2, HMOX1, HTRA2, ITPR1, JUN, KDM1A, LAMTOR1, LARP1, MAP2K2, MARK2, MFN2, MMAB, MYADM, NDUFS3, NOC2L, NUP62, OGT, OPA1, PARK7, PHB, PKN1, PPIF, PRKD1, PRPF19, PTPN2, PTPRJ, RAP1A, REPS1, RHOA, RHOC, RPL10, RPS27L, RPS3, RPS6, SFN, SGSM3, SLC35B2, SLC9A1, SOD2, STAT3, SYNJ2BP, TAX1BP3, THEM4, TP53BP1, TPR, TRIM25, TSG101, UBE2V1, USP10, VIMP, ZC3HAV1, ZDHHC13] | |
|  | GO:0016310 phosphorylation | 790.0E-6 | | 7.0E-3 | 140.0E-9 | 570.0E-9 | [5] | 7.12 | | 73.00 | [AAK1, ABL1, ADAR, ADCK3, AK1, ALDOA, ANKLE2, ARL6IP5, ATP2B4, BARD1, BAZ1B, BCCIP, BRD4, CCNT1, CD81, CDK11A, CDK11B, CDK5RAP3, CENPE, COA6, CREB1, CTSH, DDRGK1, DLG1, DNAJA3, DNAJC3, DUSP3, EEF1A2, EGFR, EPHA4, ERBB2, ERP29, ETFA, ETFDH, EZR, FN1, GBA, GTPBP4, MAP2K2, MARK2, MCM7, MMAB, MYADM, NBN, NPM1, NSD1, OGT, PAK2, PARK7, PFKFB2, PFKP, PHB, PKN1, PLK1, PRKACB, PRKAR2B, PRKD1, PTPN2, PTPRJ, RAC1, RHOA, RPS3, SDCBP, SLK, STK26, SURF1, SYNJ2BP, TOM1L1, TPR, TRIM27, TSG101, VCP, VRK1] | |
|  | GO:1902531 regulation of intracellular signal transduction | 9.6E-3 | | 41.0E-3 | 140.0E-9 | 570.0E-9 | [4, 5, 6] | 7.44 | | 61.00 | [ACTN4, ARHGDIA, ARL6IP5, ATP2B4, BAX, BCAP31, BRD4, CD81, CDK5RAP3, DDRGK1, DLG1, DNAJA3, DNM1L, DUSP3, EEF1D, EGFR, ERBB2, ERBB2IP, ERP29, ETFA, ETFDH, EZR, FN1, GBA, HMOX1, ITPR1, KDM1A, LAMTOR1, MAP2K2, MFN2, MMAB, MYADM, NDUFS3, NOC2L, NUP62, OGT, OPA1, PARK7, PHB, PKN1, PPIF, PRKD1, PTPN2, PTPRJ, RHOA, RHOC, RPL10, RPS3, SGSM3, SLC35B2, SLC9A1, SOD2, SYNJ2BP, TP53BP1, TRIM25, TSG101, UBE2V1, USP10, VIMP, ZC3HAV1, ZDHHC13] | |
|  | GO:0019220 regulation of phosphate metabolic process | 700.0E-6 | | 6.3E-3 | 140.0E-9 | 570.0E-9 | [5, 6] | 6.84 | | 61.00 | [ABL1, ADAR, ANKLE2, ARL6IP5, ATP2B4, BARD1, BCCIP, BRD4, CD81, CDK5RAP3, CENPE, DDRGK1, DLG1, DNAJA3, DNAJC3, DUSP3, EEF1A2, EGFR, ERBB2, ERP29, ETFA, ETFDH, EZR, FN1, GBA, GTPBP4, MAP2K2, MCM7, ME1, ME2, MPHOSPH10, MYADM, MYO1D, NBN, NPM1, NSD1, OGT, PAK2, PARK7, PHB, PKN1, PLK1, PPP2R4, PRKAR2B, PRKD1, PTPN2, PTPRJ, PTRH2, RAC1, RHOA, RPS3, RRP1B, SDCBP, SLC27A1, SYMPK, SYNJ2BP, TOM1L1, TRIM27, TSG101, VCP, YWHAE] | |
|  | GO:1902533 positive regulation of intracellular signal transduction | 3.4E-3 | | 19.0E-3 | 140.0E-9 | 570.0E-9 | [4, 5, 6, 7] | 6.38 | | 34.00 | [ACTN4, ARL6IP5, BAX, BCAP31, BRD4, CD81, CDK5RAP3, DDRGK1, DNM1L, EEF1D, EGFR, ERBB2, ERP29, ETFA, ETFDH, HMOX1, LAMTOR1, MAP2K2, MMAB, NUP62, PHB, PKN1, PRKD1, PTPRJ, RHOA, RHOC, RPS3, SLC35B2, SLC9A1, TP53BP1, TRIM25, UBE2V1, ZC3HAV1, ZDHHC13] | |
|  | GO:0045937 positive regulation of phosphate metabolic process | 63.0E-6 | | 930.0E-6 | 140.0E-9 | 570.0E-9 | [5, 6, 7] | 5.40 | | 31.00 | [ABL1, ANKLE2, ARL6IP5, ATP2B4, CD81, CENPE, DDRGK1, EEF1A2, EGFR, ERBB2, ERP29, ETFA, ETFDH, GBA, MAP2K2, NBN, PAK2, PARK7, PHB, PKN1, PLK1, PPP2R4, PRKD1, PTRH2, RAC1, RHOA, RPS3, SDCBP, SYMPK, TOM1L1, VCP] | |
|  | GO:0000165 MAPK cascade | 9.1E-3 | | 39.0E-3 | 140.0E-9 | 570.0E-9 | [4, 5, 6, 8, 9] | 6.08 | | 22.00 | [ARL6IP5, CD81, CDK5RAP3, CTSH, DLG1, DUSP3, EGFR, ERBB2, ERP29, ETFA, ETFDH, EZR, FN1, GBA, MAP2K2, PHB, PKN1, PTPRJ, RPS3, SYNJ2BP, TPR, TSG101] | |
|  | GO:0006468 protein phosphorylation | 72.0E-6 | | 1.0E-3 | 140.0E-9 | 570.0E-9 | [6, 7] | 6.43 | | 60.00 | [AAK1, ABL1, ADAR, ARL6IP5, ATP2B4, BAZ1B, BCCIP, BRD4, CCNT1, CD81, CDK11A, CDK11B, CDK5RAP3, CENPE, CREB1, CTSH, DDRGK1, DLG1, DNAJA3, DNAJC3, DUSP3, EGFR, EPHA4, ERBB2, ERP29, ETFA, ETFDH, EZR, FN1, GBA, GTPBP4, MAP2K2, MARK2, MMAB, MYADM, NBN, NPM1, NSD1, PAK2, PARK7, PHB, PKN1, PLK1, PRKACB, PRKAR2B, PRKD1, PTPN2, PTPRJ, RAC1, RHOA, RPS3, SDCBP, SLK, STK26, SYNJ2BP, TOM1L1, TPR, TRIM27, TSG101, VRK1] | |
|  | GO:0042325 regulation of phosphorylation | 2.9E-3 | | 17.0E-3 | 140.0E-9 | 570.0E-9 | [6, 7] | 6.89 | | 51.00 | [ABL1, ADAR, ANKLE2, ARL6IP5, ATP2B4, BARD1, BCCIP, BRD4, CD81, CDK5RAP3, CENPE, DDRGK1, DLG1, DNAJA3, DNAJC3, DUSP3, EEF1A2, EGFR, ERBB2, ERP29, ETFA, ETFDH, EZR, FN1, GBA, GTPBP4, MAP2K2, MCM7, MYADM, NBN, NPM1, NSD1, OGT, PAK2, PARK7, PHB, PKN1, PLK1, PRKAR2B, PRKD1, PTPN2, PTPRJ, RAC1, RHOA, RPS3, SDCBP, SYNJ2BP, TOM1L1, TRIM27, TSG101, VCP] | |
|  | GO:0001932 regulation of protein phosphorylation | 1.1E-3 | | 8.4E-3 | 140.0E-9 | 570.0E-9 | [6, 7, 8] | 6.50 | | 45.00 | [ABL1, ADAR, ARL6IP5, ATP2B4, BCCIP, BRD4, CD81, CDK5RAP3, CENPE, DDRGK1, DLG1, DNAJA3, DNAJC3, DUSP3, EGFR, ERBB2, ERP29, ETFA, ETFDH, EZR, FN1, GBA, GTPBP4, MAP2K2, MYADM, NBN, NPM1, NSD1, PAK2, PARK7, PHB, PKN1, PLK1, PRKAR2B, PRKD1, PTPN2, PTPRJ, RAC1, RHOA, RPS3, SDCBP, SYNJ2BP, TOM1L1, TRIM27, TSG101] | |
|  | GO:0042327 positive regulation of phosphorylation | 100.0E-6 | | 1.4E-3 | 140.0E-9 | 570.0E-9 | [6, 7, 8] | 5.19 | | 26.00 | [ABL1, ARL6IP5, ATP2B4, CD81, CENPE, DDRGK1, EEF1A2, EGFR, ERBB2, ERP29, ETFA, ETFDH, MAP2K2, NBN, PAK2, PARK7, PHB, PKN1, PLK1, PRKD1, RAC1, RHOA, RPS3, SDCBP, TOM1L1, VCP] | |
|  | GO:0001934 positive regulation of protein phosphorylation | 76.0E-6 | | 1.0E-3 | 140.0E-9 | 570.0E-9 | [6, 7, 8, 9] | 5.00 | | 24.00 | [ABL1, ARL6IP5, ATP2B4, CD81, CENPE, DDRGK1, EGFR, ERBB2, ERP29, ETFA, ETFDH, MAP2K2, NBN, PAK2, PARK7, PHB, PKN1, PLK1, PRKD1, RAC1, RHOA, RPS3, SDCBP, TOM1L1] | |
|  | GO:0043410 positive regulation of MAPK cascade | 5.1E-3 | | 25.0E-3 | 140.0E-9 | 570.0E-9 | [5, 6, 7, 8, 9, 10, 11] | 4.74 | | 11.00 | [ARL6IP5, CD81, EGFR, ERBB2, ERP29, ETFA, ETFDH, MAP2K2, PHB, PKN1, RPS3] | |
|  | GO:0007030 Golgi organization | 18.0E-6 | | 400.0E-6 | 1.3E-6 | 3.6E-6 | [4] | 28.36 | | 19.00 | [ATL2, COG2, COG3, COG4, GBF1, GCC2, GOLGA5, GOLPH3L, LMAN1, MYO18A, PRKD1, PRMT5, RAB2A, STX18, SYNE1, TMED5, VRK1, VTI1A, ZW10] | |
|  | GO:0072657 protein localization to membrane | 7.4E-3 | | 34.0E-3 | 1.3E-6 | 3.6E-6 | [4, 5] | 15.81 | | 34.00 | [ATP1B1, ATP1B3, BAG6, CDH1, DLG1, ERBB2, EZR, FLOT2, GCC2, JUP, MACF1, MYADM, MYO1C, PDCD5, PEX16, PKP2, PKP3, PPFIA1, PPIL2, RAB10, RAB11A, RAB11B, RAB3GAP2, SAMM50, SEC61A1, SEC62, SEC63, STX3, STX8, STXBP1, SYNE3, TIMM10, TMBIM1, VTI1B] | |
|  | GO:0090150 establishment of protein localization to membrane | 6.9E-3 | | 32.0E-3 | 1.3E-6 | 3.6E-6 | [4, 5, 6] | 16.88 | | 27.00 | [BAG6, CDH1, DLG1, ERBB2, EZR, FLOT2, GCC2, JUP, MACF1, MYADM, MYO1C, PDCD5, PEX16, PKP2, PKP3, PPFIA1, RAB10, RAB11A, RAB11B, RAB3GAP2, SAMM50, SEC61A1, SEC62, SEC63, SYNE3, TIMM10, TMBIM1] | |
|  | GO:0022406 membrane docking | 12.0E-3 | | 49.0E-3 | 12.0E-3 | 12.0E-3 | [3] | 33.33 | | 5.00 | [EZR, PEX13, PEX16, RAB7A, SNX3] | |
|  | GO:0044712 single-organism catabolic process | 120.0E-6 | | 1.6E-3 | 13.0E-6 | 31.0E-6 | [3] | 16.94 | | 52.00 | [ABCD3, ABHD12, ABHD16A, ABHD2, ABHD6, ACAA1, ACADM, ACSF3, ALDH5A1, ALDH6A1, ALDOA, AMFR, ATP2B4, BAG6, BCAP31, BCKDHA, BLVRB, CROT, DERL1, ETFA, ETFDH, FAAH, GBA, GCDH, GOT1, HMOX1, HOGA1, KIAA0368, LONP2, MAN1B1, MTHFS, NAGA, NPLOC4, OGT, OS9, PFKFB2, PFKP, PNPLA2, PNPLA8, PPT1, SLC25A17, SLC27A2, SMPDL3A, SORD, STBD1, TMUB1, TRIM25, UBAC2, UBXN1, VCP, VIMP, WFS1] | |
|  | GO:0034976 response to endoplasmic reticulum stress | 1.1E-3 | | 8.6E-3 | 13.0E-6 | 31.0E-6 | [4] | 19.26 | | 26.00 | [AMFR, BAG6, BAX, BCAP31, CDK5RAP3, DERL1, DNAJC3, ERP44, JUN, KIAA0368, MAN1B1, NPLOC4, OPA1, OS9, PARK7, TMCO1, TMUB1, TP53BP1, TRIM25, UBAC2, UBXN1, UFL1, UFM1, VCP, VIMP, WFS1] | |
|  | GO:0036503 ERAD pathway | 1.0E-3 | | 8.3E-3 | 13.0E-6 | 31.0E-6 | [3, 4, 5, 6] | 23.81 | | 15.00 | [AMFR, BAG6, BCAP31, DERL1, KIAA0368, MAN1B1, NPLOC4, OS9, TMUB1, TRIM25, UBAC2, UBXN1, VCP, VIMP, WFS1] | |
|  | GO:0030970 retrograde protein transport, ER to cytosol | 9.2E-3 | | 39.0E-3 | 13.0E-6 | 31.0E-6 | [4, 5, 6, 7, 8] | 28.00 | | 7.00 | [BCAP31, DERL1, NPLOC4, OS9, UBAC2, VCP, VIMP] | |
|  | GO:0032527 protein exit from endoplasmic reticulum | 2.6E-3 | | 15.0E-3 | 13.0E-6 | 31.0E-6 | [5, 6, 7] | 27.03 | | 10.00 | [BCAP31, DERL1, GCC2, LMAN1, NPLOC4, OS9, PREB, UBAC2, VCP, VIMP] | |
|  | GO:0033108 mitochondrial respiratory chain complex assembly | 110.0E-6 | | 1.5E-3 | 110.0E-6 | 230.0E-6 | [5, 6, 7] | 35.48 | | 11.00 | [ACAD9, COA3, FOXRED1, NDUFAF4, NDUFS8, NUBPL, SAMM50, SMIM20, SURF1, TIMM21, TTC19] | |
|  | GO:0017004 cytochrome complex assembly | 4.5E-3 | | 24.0E-3 | 110.0E-6 | 230.0E-6 | [6, 7] | 35.29 | | 6.00 | [COA3, COA6, SMIM20, SURF1, TIMM21, TTC19] | |
|  | GO:0032981 mitochondrial respiratory chain complex I assembly | 8.3E-3 | | 37.0E-3 | 110.0E-6 | 230.0E-6 | [5, 6, 7, 8] | 31.58 | | 6.00 | [ACAD9, FOXRED1, NDUFAF4, NDUFS8, NUBPL, TIMM21] | |
|  | GO:0048518 positive regulation of biological process | 1.0E-3 | | 8.4E-3 | 11.0E-6 | 28.0E-6 | [1, 2, 3] | 8.58 | | 263.00 | [AAK1, ABL1, ACTN4, ACTR2, ACTR3, ADAR, AHCYL1, ANKLE2, ARL6IP5, ARPIN, ASNS, ATAD2, ATF7IP, ATP1B1, ATP1B3, ATP2B4, ATP7A, BAD, BAG6, BARD1, BAX, BCAP31, BPNT1, BRCA1, BRD4, BRD7, BUD31, C14orf166, CCNT1, CCT2, CCT4, CCT5, CCT6A, CCT7, CCT8, CD276, CD81, CDC73, CDCA5, CDH1, CDK5RAP2, CDK5RAP3, CENPE, CEP131, CFAP20, CHD6, CHERP, CHTF8, COA3, CREB1, CTBP1, CTR9, CTSH, CYB5R1, DDRGK1, DERL1, DHX33, DKC1, DLG1, DMAP1, DNAJA3, DNAJC2, DNM1L, DNMT1, DNPEP, DUSP3, EEF1A2, EEF1D, EEF2, EGFR, EHF, EIF5A, EPB41L4B, EPHA3, EPHA4, EPT1, ERBB2, ERBB2IP, ERCC3, ERP29, ETFA, ETFDH, EZR, FASN, FLOT2, FN1, FXR1, GARS, GBA, GDF15, GIPC1, GNL3, GNL3L, GOLPH3L, GRB2, HCFC1, HK2, HMGB2, HMOX1, HTRA2, HUWE1, IDE, ING3, JUN, JUP, KDM1A, KIF20B, KMT2D, KRT17, LAMP1, LAMTOR1, LARP1, LARP4B, LMAN1, MACF1, MAP2K2, MAPT, MARK2, MIPEP, MLYCD, MMAB, MTCH1, MTPN, MYADM, MYDGF, MYO18A, MYO1C, NABP2, NACC1, NBN, NCBP1, NCOA6, NDUFA2, NEDD4L, NOL11, NOP10, NPAT, NPEPPS, NPM1, NR2C2, NSD1, NSF, NUP62, OGT, PAK2, PARK7, PCM1, PDCD5, PDCD6IP, PELP1, PFDN2, PFN1, PHB, PHF2, PHIP, PICALM, PINX1, PKN1, PKP4, PLK1, PNPLA2, PNPT1, POLDIP3, POLR2A, POLR2D, PPIA, PPIE, PPIH, PPP2R4, PPT1, PRDX5, PRKD1, PRMT1, PRMT5, PSMA6, PTPN11, PTPRJ, PTRH2, QARS, RAB11A, RAB3B, RAB3GAP2, RAB7A, RAB9A, RAC1, RAC2, RAC3, RALA, RBM14, RBM5, REST, RFC2, RHOA, RHOC, RNF40, RNMT, RNPS1, RPL10, RPRD1B, RPS15A, RPS3, RPS4X, RPS6, RPSA, RTF1, RUVBL1, RUVBL2, SCRIB, SDCBP, SENP1, SETSIP, SIN3A, SLC35B2, SLC9A1, SMARCB1, SMARCD1, SNIP1, SP3, SPAG9, SPEN, SPG7, STAT3, STRN3, STX18, STX3, SUPT5H, SUPT6H, SUPV3L1, SYMPK, TAF9, TCP1, TFAM, TFB2M, TNPO1, TOM1L1, TOP2A, TOR1A, TP53BP1, TPR, TRA2B, TRIM25, TRIM27, TRIOBP, TSG101, U2AF1, U2AF2, UBE2V1, UFL1, UHRF1, USP36, USP47, UTRN, VAMP7, VCP, WASF2, WDR46, WDR61, WFS1, WRAP53, YTHDF2, ZC3HAV1, ZDHHC13, ZNF746] | |
|  | GO:0048519 negative regulation of biological process | 3.8E-3 | | 20.0E-3 | 11.0E-6 | 28.0E-6 | [1, 2, 3] | 8.51 | | 205.00 | ABCE1, ABL1, ACAA2, ACTN1, ACTN4, ADAR, AIMP1, ANKLE2, ANXA7, AP2A1, ARID2, ARPIN, ASNS, ATF7IP, ATP2B4, ATP5A1, ATXN2, BAD, BARD1, BCOR, BPNT1, BRCA1, BRD4, BRD7, CBX2, CCNT1, CDC5L, CDC73, CDH1, CDK5RAP3, CENPF, CHERP, CHID1, CREB1, CTBP1, CTR9, CTSA, DLG1, DMAP1, DNAJA3, DNAJC3, DNM2, DNMT1, DNPEP, DUSP3, EFNA5, EGFR, EHMT2, EIF6, ENO1, ERBB2IP, ERP29, ETF1, EZR, FAM60A, FLOT2, FN1, FXR1, GATAD2A, GBA, GNL3L, GTPBP4, HMGB2, HMOX1, HSPA8, ITPR1, JUN, KDM1A, KRT17, LIG3, MAFB, MAP2K2, MAP4, MBD2, MFN2, MMAB, MPHOSPH10, MPHOSPH8, MTPN, MYADM, MYO18A, MYO1D, NABP2, NACC1, NAE1, NBN, NDUFS3, NEDD4L, NKRF, NOC2L, NPM1, NUP153, NUP62, OPA1, ORMDL1, ORMDL2, OS9, OSBPL8, PAK2, PARK7, PBRM1, PDCD5, PFDN5, PFN1, PHB, PHF12 PHF2, PHF21A, PHIP, PIAS2, PICALM, PINX1, PKN1, PKP2, PLK1, PNPLA2, PNPT1, POLR1B, PPFIA1, PPIF, PPP2R4, PPT1, PRCC, PRDX5, PRKAR2B, PRKD1, PRMT1, PRPF19, PTPN2, PTPRJ, PTRH2, PYCR1, RAB7A, RAC1, RBFOX2, RBM14, RBM15B, RCOR1, REST, RHOA, RNF40, RNMT, RNPS1, RPA2, RPL10, RPS27L, RPS3, RPSA, RRP1B, RTN4, SAP130, SCRIB, SDCBP, SERPINB6, SET, SFN, SIGIRR, SIN3A, SKI, SLC27A1, SMARCB1, SNIP1, SNX3, SOD2, SP3, SPEN, STAG2, STAT3, STRN3, SUPT5H, SUPT6H, SUPV3L1, SYNJ2BP, TAF3, TAF9, TAX1BP3, TFIP11, TMBIM1, TMEM109, TOM1L1, TOM1L2, TP53BP1, TPR, TRAP1, TRIM27, TSG101, U2AF2, UBAC2, UBE2V1, UBXN1, UFL1, UHRF1, USP10, USP47, VIMP, WASF2, WDR61, WFS1, YEATS2, YRDC, YWHAE, ZC3HAV1, ZNF148, ZNF746, ZW10] | |
|  | GO:0050794 regulation of cellular process | 32.0E-6 | | 590.0E-6 | 11.0E-6 | 28.0E-6 | [2, 3] | 8.81 | | 420.00 | [AAK1, ABCE1, ABHD2, ABL1, ACAA2, ACTN1, ACTN4, ACTR2, ACTR3, ADAR, AHCYL1, AIMP1, ALDOA, ALKBH5, AMFR, ANKLE2, ANXA5, ARHGAP1, ARHGDIA, ARID2, ARL6IP5, ARPIN, ASNS, ASUN, ATAD2, ATF7IP, ATP1B1, ATP1B3, ATP2A2, ATP2B4, ATP5A1, ATP6V0A1, ATXN2, ATXN2L, BAD, BAG6, BAIAP2, BARD1, BAX, BCAP31, BCCIP, BCOR, BET1L, BPNT1, BRCA1, BRD2, BRD4, BRD7, BUD31, C14orf166, CBX2, CCNT1, CCT2, CCT4, CCT5, CCT6A, CCT7, CCT8, CD276, CD81, CDC27, CDC37, CDC5L, CDC73, CDCA5, CDH1, CDK11A, CDK11B, CDK5RAP2, CDK5RAP3, CENPE, CENPF, CEP131, CFAP20, CHD6, CHERP, CHTF8, COA3, CREB1, CTBP1, CTR9, CTSA, CTSH, CYB5R1, DDRGK1, DERL1, DHX33, DKC1, DLG1, DMAP1, DNAJA3, DNAJC1, DNAJC2, DNAJC3, DNM1L, DNM2, DNMT1, DNPEP, DSG2, DSP, DUSP3, EEF1A2, EEF1D, EEF2, EFNA5, EGFR, EHF, EHMT2, EIF1, EIF2A, EIF4G2, EIF5, EIF5A, EIF5B, EIF6, ELMOD2, ENO1, EPB41L4B, EPHA3, EPT1, ERBB2, ERBB2IP, ERCC3, ERP29, ETF1, ETFA, ETFDH, EZR, FAM60A, FARP2, FASN, FITM2, FLOT2, FN1, FXR1, GARS, GATAD2A, GBA, GBF1, GDF15, GIPC1, GNAI3, GNL3, GNL3L, GOLPH3L, GRB2, GTPBP4, HCFC1, HK2, HMGB2, HMOX1, HSPA8, HTRA2, HUWE1, IDE, ING3, ITGA2, ITPR1, JAGN1, JAK3, JUN, JUP, KDM1A, KIF20B, KIF2C, KMT2D, KRT17, LAMP1, LAMTOR1, LARP1, LARP4B, LIG3, LMAN1, LONP2, MACF1, MAD1L1, MAFB, MAP2K2, MAP4, MAPT, MARK2, MBD2, MCM7, MDH1, ME1, ME2, MFN2, MKI67, MLYCD, MMAB, MPHOSPH10, MPHOSPH8, MRPS10, MTCH1, MTG1, MTPN, MYADM, MYDGF, MYO18A, MYO1C, MYO1D, NABP2, NACC1, NAE1, NBN, NCBP1, NCLN, NCOA6, NDUFS3, NEDD4L, NKRF, NOC2L, NOL11, NPAT, NPEPPS, NPM1, NR2C2, NSD1, NSF, NSRP1, NUP62, OGT, OPA1, ORMDL1, ORMDL2, OS9, PAK2, PARK7, PARL, PBRM1, PDCD5, PDCD6IP, PDZD8, PELP1, PEX13, PFDN2, PFDN5, PFN1, PHB, PHF12, PHF2, PHF21A, PHIP, PIAS2, PICALM, PINX1, PKN1, PKP2, PKP4, PLK1, PNPLA2, PNPT1, POLDIP3, POLR1B, POLR2A, POLR2D, PPFIA1, PPIA, PPIE, PPIF, PPIH, PPP2R4, PPT1, PQBP1, PRCC, PRDX5, PRKAR2B, PRKD1, PRMT1, PRMT5, PRPF19, PSMA6, PSMD14, PTPN11, PTPN2, PTPRJ, PTRH2, PUM3, PYCR1, QARS, RAB11A, RAB3B, RAB3GAP2, RAB7A, RAB9A, RAC1, RAC2, RAC3, RALA, RAP1A, RBFOX2, RBM14, RBM15B, RBM25, RBM5, RCOR1, REPS1, REST, RFC2, RHOA, RHOC, RHOT1, RNF40, RNMT, RNPS1, RPA2, RPL10, RPRD1B, RPS15A, RPS27L, RPS3, RPS4X, RPS6, RPSA, RRP1B, RTF1, RTN4, RUVBL1, RUVBL2, SAP130, SCAF1, SCRIB, SDCBP, SENP1, SERPINB6, SET, SETSIP, SFN, SGSM3, SIGIRR, SIN3A, SKI, SLC25A24, SLC27A1, SLC33A1, SLC35B2, SLC9A1, SLK, SMARCB1, SMARCD1, SNIP1, SNRPB, SNX3, SOD2, SP3, SPAG9, SPEN, SPG7, SPTY2D1, STAG2, STAT3, STK26, STRN3, STX18, STX3, STX8, SUPT5H, SUPT6H, SUPV3L1, SYMPK, SYNE3, SYNJ2BP, TAF3, TAF9, TAP1, TAX1BP3, TCP1, TFAM, TFB2M, TFIP11, TFPT, THEM4, TMBIM1, TMCO1, TMEM109, TOM1L1, TOM1L2, TOP2A, TOR1A, TP53BP1, TPR, TRA2B, TRAP1, TRIM24, TRIM25, TRIM27, TRIOBP, TSG101, U2AF1, U2AF2, UBAC2, UBE2V1, UBXN1, UFL1, UFM1, UHRF1, USP10, USP36, USP47, VAMP7, VCP, VIMP, VTI1B, WASF2, WDR1, WDR46, WDR61, WFS1, WRAP53, YEATS2, YTHDC1, YTHDF2, YWHAE, ZC3HAV1, ZCCHC17, ZDHHC13, ZMYM4, ZNF148, ZNF746, ZW10] | |
|  | GO:0048522 positive regulation of cellular process | 4.8E-3 | | 24.0E-3 | 11.0E-6 | 28.0E-6 | [2, 3, 4] | 8.68 | | 243.00 | [AAK1, ABL1, ACTN4, ACTR2, ACTR3, ADAR, ANKLE2, ARL6IP5, ARPIN, ASNS, ATAD2, ATF7IP, ATP1B1, ATP1B3, ATP2B4, BAD, BAG6, BARD1, BAX, BCAP31, BRCA1, BRD4, BRD7, BUD31, C14orf166, CCNT1, CCT2, CCT4, CCT5, CCT6A, CCT7, CCT8, CD276, CD81, CDC73, CDCA5, CDH1, CDK5RAP2, CDK5RAP3, CENPE, CEP131, CFAP20, CHD6, CHTF8, COA3, CREB1, CTBP1, CTR9, CTSH, CYB5R1, DDRGK1, DERL1, DHX33, DKC1, DLG1, DMAP1, DNAJA3, DNAJC2, DNM1L, DNMT1, DNPEP, DUSP3, EEF1A2, EEF1D, EEF2, EGFR, EHF, EIF5A, EPB41L4B, EPHA3, EPT1, ERBB2, ERCC3, ERP29, ETFA, ETFDH, EZR, FASN, FLOT2, FN1, FXR1, GARS, GBA, GDF15, GIPC1, GNL3, GOLPH3L, GRB2, HK2, HMGB2, HMOX1, HTRA2, HUWE1, IDE, ING3, JUN, KDM1A, KIF20B, KMT2D, KRT17, LAMP1, LAMTOR1, LARP1, LARP4B, LMAN1, MACF1, MAP2K2, MAPT, MARK2, MLYCD, MMAB, MTCH1, MYADM, MYDGF, MYO18A, MYO1C, NABP2, NACC1, NBN, NCBP1, NCOA6, NEDD4L, NOL11, NPAT, NPEPPS, NPM1, NR2C2, NSD1, NSF, NUP62, OGT, PAK2, PARK7, PDCD5, PDCD6IP, PELP1, PFDN2, PFN1, PHB, PHF2, PHIP, PICALM, PKN1, PKP4, PLK1, PNPLA2, PNPT1, POLDIP3, POLR2A, POLR2D, PPIA, PPIE, PPIH, PPP2R4, PPT1, PRKD1, PRMT1, PRMT5, PTPN11, PTPRJ, PTRH2, QARS, RAB11A, RAB3B, RAB3GAP2, RAB7A, RAB9A, RAC1, RAC2, RAC3, RALA, RBM14, RBM5, REST, RFC2, RHOA, RHOC, RNF40, RNMT, RNPS1, RPL10, RPRD1B, RPS15A, RPS3, RPS4X, RPS6, RPSA, RTF1, RUVBL1, RUVBL2, SCRIB, SDCBP, SENP1, SETSIP, SIN3A, SLC35B2, SLC9A1, SMARCB1, SMARCD1, SNIP1, SP3, SPAG9, SPEN, STAT3, STRN3, STX18, STX3, SUPT5H, SUPT6H, SUPV3L1, SYMPK, TAF9, TCP1, TFAM, TFB2M, TOM1L1, TOP2A, TOR1A, TP53BP1, TPR, TRA2B, TRIM25, TRIM27, TRIOBP, TSG101, U2AF1, U2AF2, UBE2V1, UFL1, UHRF1, USP36, USP47, VAMP7, VCP, WASF2, WDR46, WDR61, WFS1, WRAP53, YTHDF2, ZC3HAV1, ZDHHC13, ZNF746] | |
|  | GO:0048523 negative regulation of cellular process | 7.8E-3 | | 35.0E-3 | 11.0E-6 | 28.0E-6 | [2, 3, 4] | 8.56 | | 192.00 | [ABCE1, ABL1, ACAA2, ACTN1, ACTN4, ADAR, AIMP1, ANKLE2, ARID2, ARPIN, ASNS, ATF7IP, ATP2B4, ATP5A1, ATXN2, BAD, BARD1, BCOR, BPNT1, BRCA1, BRD4, BRD7, CBX2, CCNT1, CDC5L, CDC73, CDH1, CDK5RAP3, CENPF, CHERP, CREB1, CTBP1, CTR9, CTSA, DLG1, DMAP1, DNAJA3, DNAJC3, DNM2, DNMT1, DNPEP, DUSP3, EFNA5, EGFR, EHMT2, EIF6, ENO1, ERBB2IP, ERP29, ETF1, EZR, FAM60A, FN1, FXR1, GATAD2A, GBA, GNL3L, GTPBP4, HMGB2, HMOX1, HSPA8, ITPR1, JUN, KDM1A, LIG3, MAFB, MAP2K2, MAP4, MBD2, MFN2, MMAB, MPHOSPH10, MPHOSPH8, MTPN, MYADM, MYO18A, MYO1D, NABP2, NACC1, NAE1, NBN, NDUFS3, NEDD4L, NKRF, NOC2L, NPM1, NUP62, OPA1, ORMDL1, ORMDL2, OS9, PAK2, PARK7, PBRM1, PDCD5, PFDN5, PFN1, PHB, PHF12, PHF2, PHF21A, PHIP, PIAS2, PICALM, PINX1, PKN1, PKP2, PLK1, PNPT1, POLR1B, PPFIA1, PPIF, PPP2R4, PPT1, PRCC, PRDX5, PRKAR2B, PRKD1, PRMT1, PRPF19, PTPN2, PTPRJ, PTRH2, PYCR1, RAB7A, RBFOX2, RBM14, RBM15B, RCOR1, REST, RHOA, RNF40, RNMT, RNPS1, RPA2, RPL10, RPS27L, RPS3, RRP1B, RTN4, SAP130, SCRIB, SDCBP, SERPINB6, SET, SFN, SIN3A, SKI, SLC27A1, SMARCB1, SNIP1, SNX3, SOD2, SP3, SPEN, STAG2, STAT3, STRN3, SUPT5H, SUPT6H, SUPV3L1, SYNJ2BP, TAF3, TAF9, TFIP11, TMBIM1, TMEM109, TOM1L1, TOM1L2, TP53BP1, TPR, TRAP1, TRIM27, TSG101, U2AF2, UBAC2, UBE2V1, UBXN1, UFL1, UHRF1, USP10, USP47, VIMP, WASF2, WDR61, WFS1, YEATS2, YWHAE, ZC3HAV1, ZNF148, ZNF746, ZW10] | |
|  | GO:0051310 metaphase plate congression | 2.6E-3 | | 15.0E-3 | 9.7E-3 | 10.0E-3 | [4, 5, 6] | 25.58 | | 11.00 | [CDCA5, CDCA8, CENPE, CENPF, KIF22, KIF2C, MAD1L1, PDCD6IP, PINX1, RAB11A, ZW10] | |
|  | GO:0007080 mitotic metaphase plate congression | 10.0E-3 | | 44.0E-3 | 9.7E-3 | 10.0E-3 | [4, 5, 6, 7, 8] | 23.68 | | 9.00 | [CDCA5, CDCA8, CENPE, KIF22, KIF2C, PDCD6IP, PINX1, RAB11A, ZW10] | |
|  | GO:0002682 regulation of immune system process | 3.0E-3 | | 17.0E-3 | 14.0E-6 | 31.0E-6 | [2, 3] | 6.58 | | 40.00 | [ALKBH5, ANXA5, ATP1B1, BAX, CD276, CD81, CDC37, CDC73, CTR9, CTSH, DNAJA3, DUSP3, ELMOD2, ERBB2IP, EZR, FLOT2, HMGB2, LAMP1, MAFB, MDH1, MRPS10, MYDGF, OGT, PEX13, PHB, PRMT1, PTPN2, PTPRJ, RAC1, RAC2, RPL10, RPSA, SIN3A, SNRPB, TRIM27, VAMP7, VIMP, WDR61, ZC3HAV1, ZCCHC17] | |
|  | GO:0002684 positive regulation of immune system process | 1.5E-3 | | 10.0E-3 | 14.0E-6 | 31.0E-6 | [2, 3, 4] | 5.26 | | 19.00 | [BAX, CD276, CD81, DUSP3, ERBB2IP, EZR, FLOT2, HMGB2, LAMP1, OGT, PHB, PRMT1, PTPRJ, RAC1, RAC2, RPSA, SIN3A, VAMP7, ZC3HAV1] | |

**Supplementary table 5**

| **GENE NAME** | **PROTEIN NAME** | **PATHWAY** | **GO:BIOLOGICAL PROCESS** | **REFERENCES** |
| --- | --- | --- | --- | --- |
| ADAMTS1 | A disintegrin and metalloproteinase with thrombospondin motifs 1 | TGF-β1/ADAMTS-1 signaling pathway; Cell adesion | Negative regulation of cell proliferation; integrin-mediated signaling; negative regulation of cell proliferation | "**The metalloproteinase ADAMTS1: A comprehensive review of its role in tumorigenic and metastatic pathways**" , I de Arao Tao et al., IJC (2013), 133, 2263. "**ADAMTS1, a putative anti-angiogenic factor, is decreased in human prostate cancer**", H Gustavsson et al. (2009), BJU International, 104, 1786. |
| AKT | RAC-alpha serine/threonine-protein kinase | PI3K | Activation-induced cell death of T cells; aging ; anoikis;  apoptotic mitochondrial changes; cell differentiation; cell  projection organization; cell proliferation; cellular protein modification process; cellular response to DNA damage stimulus | "**Combined AKT and MEK Pathway Blockade in Pre-Clinical Models of Enzalutamide-Resistant Prostate Cancer.**" P Toren et al. (2016), PlosOne, 11(4):e0152861; "**Crosstalking between androgen and PI3K/AKT signaling pathways in prostate cancer cells.**" SH Lee et al. (2015), J Biol Chem., 290(5):2759; "**Long-term androgen-ablation causes increased resistance to PI3K/Akt pathway inhibition in prostate cancer cells.**", K Pfeil et al. (2004), Prostate, 58, 259. |
| ALDH3A2 | Fatty aldehyde dehydrogenase | Metabolism pathways glycolysis/ gluconeogenesis | Cellular aldehyde metabolic process; oxidation-reduction  process; epidermis development; fatty acid alpha- oxidation; phytol metabolic process; sesquiterpenoid  metabolic process; sphingolipid biosynthetic process | "**High aldehyde dehydrogenase activity identifies tumor-initiating and metastasis-initiating cells in human prostate cancer.**" C. van den Hoogen et al. (2010), Cancer Res, 70,5163; "**The role of tumor metabolism as a driver of prostate cancer progression and lethal disease: results from a nested case-control study**.", RS Kelly et al. (2016) Cancer Metab., 4, 22. |
| ASPH | Aspartyl/asparaginyl beta-hydroxylase | NOTCH | Cellular response to calcium ion; cellular response to  calcium ion; positive regulation of intracellular protein  transport; positive regulation of proteolysis; positive  regulation of transcription, DNA-templated; regulation of release of sequestered calcium ion into cytosol by  sarcoplasmic reticulum; response to ATP | "**Aspartate β-Hydroxylase expression promotes a malignant pancreatic cellular phenotype**." X Dong et al. (2015), Oncotarget, 6,1231; "**Role of Notch signaling pathway in pancreatic cancer**." J Gao et al., (2017), Am. J. Cancer Res. 7(2),173; "**Aspartate-β-hydroxylase (ASPH): A potential therapeutic target in human malignant gliomas.**" LM Sturla et al. (2016), Helyon, 2,e00203; "**Activation of signal transduction pathways during hepatic oncogenesis.**", W Chung et al. (2016), Cancer Lett. 370, 1; **"Notch signaling: An emerging therapeutic target for cancer treatment**". X. Yuan et al. (2015), Cancer Lett. 369,20-27. |
| CANX | Calnexin | Protein processing in endoplasmic reticulum; Phagosome | Protein folding; protein secretion; chaperone-mediated  protein folding; clathrin-dependent endocytosis; aging | "**Calnexin is a novel sero-diagnostic marker for lung cancer.**" M Kobayashi et al. (2015), Lung Cancer, 90,342; "**Calnexin, an ER-induced protein, is a prognostic marker and potential therapeutic target in colorectal cancer.**" D Ryan et al. (2016), J. Transl. Med., 14, 196. |
| EHF | ETS homologous factor | IL-6/JAK/STAT3 | DNA binding; transcriptional activator activity, RNA  polymerase II core promoter proximal region sequence- specific binding; transcription factor activity, sequence- specific DNA binding | "**The ETS factor ESE3/EHF represses IL-6 preventing STAT3 activation and expansion of the prostate cancer stem-like compartment.**", D Albino et al. (2016),7,76756; "**ESE3/EHF controls epithelial cell differentiation and its loss leads to prostate tumors with mesenchymal and stem-like features.**" D Albino et al. (2012), Cancer Res., 72, 2889. |
| EPHA3 | Ephrin type-A receptor 3 | MAPK | Cell adhesion; cell migration; regulation of actin  cytoskeleton organization; regulation of epithelial to  mesenchymal transition; regulation of focal adhesion  assembly | "**EphA3, induced by PC-1/PrLZ, contributes to the malignant progression of prostate cancer.**" R Wu et al. (2014), Onc. Rep., 32, 2657; "**EphA3 biology and cancer.**", PW Janes et al. (2014), Growth Factors, 32,176;"**Eph receptors and ephrins: therapeutic opportunities.**" A Barquilla et al. (2015), Annu. Rev. Pharmacol. Toxicol., 55, 465. |
| **GENE NAME** | **PROTEIN NAME** | **PATHWAY** | **GO:BIOLOGICAL PROCESS** | **REFERENCES** |
| ETV1 | ETS translocation variant 1 | Transcriptional mysregulation in cancer | Positive regulation of transcription from RNA polymerase II; transcription from RNA ; axon guidance;  mechanosensory behavior;muscle organ development ;  peripheral nervous system neuron development | "**Induction of prostatic intraepithelial neoplasia and modulation of androgen receptor by ETS variant 1/ETS-related protein 81.**", S Shin et al. (2009), Cancer Res, 69, 8102; "**ETV1 is a novel androgen receptor-regulated gene that mediates prostate cancer cell invasion**." C Cai et al. (2007), Mol. Endocrinol., 21,1835; "**ETV1 directs androgen metabolism and confers aggressive prostate cancer in targeted mice and patients**." E Baena et al. (2013), Gene Dev., 27,683. |
| hnRNP U | Heterogeneous nuclear ribonucleoprotein U | Splicesome; NF-κB ; AKT/GSK3β/βTrCP/ hnRNP-U | mRNA splicing, via spliceosome; cardiac muscle cell  development; cellular response to dexamethasone  stimulus; circadian regulation of gene expression; CRD- mediated mRNA; stabilization; gene expression; RNA  processing | "**Splicing factors of SR and hnRNP families as regulators of apoptosis in cancer.**" H Kędzierska (2017), Cancer Lett. 396, 53; "**The hnRNP family: insights into their role in health and disease.**", T Geuens et al (2016), Hum. Genet.,135, 851. |
| MAPK14 | p38MAPK | MAPK | Activation of MAPK activity; p38MAPK cascade; signal  transduction in response to DNA damage; intracellular  signal transduction; Ras protein signal transduction;  negative regulation of canonical Wnt signaling pathway;  apoptotic process; cell surface receptor signaling  pathway; chemotaxis; DNA damage checkpoint; positive  regulation of reactive oxygen species metabolic process | "**Upregulation of MAPK pathway is associated with survival in castrate-resistant prostate cancer.**" R Mukherjee et al., Br. J. Cancer (2011), 104,1920; "**p38MAPK activation is involved in androgen-independent proliferation of human prostate cancer cells by regulating IL-6 secretion.**", Y Shida et al., BBRC (2007), 353,744; "**Epidermal growth factor induces p38 MAPK-dependent G0/G1-to-S transition in prostate cancer cells upon androgen deprivation conditions.**" G Rodríguez-Berriguete et al. (2016), J. Growth Factors, 34 ,5. |
| NCAM2 | Neural cell adhesion molecule 2 | Cell adhesion | Neuron cell-cell adhesion; axonal fasciculation; sensory  perception of smell | "**Neural cell adhesion molecule 2 as a target molecule for prostate and breast cancer gene therapy.**" S Takahashi et al. (2011), Cancer Sci. 102,808; "**Expression analysis onto microarrays of randomly selected cDNA clones highlights HOXB13 as a marker of human prostate cancer.**" S Edward et al. (2005) Br. J. Cancer, 92(2), 376. |
| PARP1 | Poly [ADP-ribose] polymerase 1 | Base excision repair | ATP generation from poly-ADP-D-ribose; cellular  response to DNA damage stimulus; DNA repair; double- strand break repair; negative regulation of transcription  from RNA polymerase II promoter; positive regulation of transcription from RNA polymerase II promoter; protein modification process | "**Dual roles of PARP-1 promote cancer growth and progression.**" M Schiewer et al. (2012), Cancer Discov., 2, 1134; "**Role of PARP-1 in prostate cancer.**" D Deshmukh and Y Qiu (2015) Am. J. Clin. Exp. Urol,. 3, 1-12; "**PARP-1 regulates epithelial-mesenchymal transition (EMT) in prostate tumorigenesis.**" H Pu et al- (2014), Carinogenesis, 35, 2592. |
| PEBP1 | Raf kinase inhibitor protein (RKIP) | RAS/RAF/MAPK | MAPK cascade; negative regulation of peptidase activity; nucleotide binding; RNA binding; protein binding; lipid binding | " **Raf kinase inhibitory protein: a signal transduction modulator and metastasis suppressor.**" AE Granovsky (2008), Cell Res. 18, 452; "**Loss of Raf kinase inhibitory protein induces radioresistance in prostate cancer.**" KM Woods Ignatoski et al. (2008), Int. J. Radiat. Oncol. Biol. Phys., 72, 153 |
| QKI | Quaking | RAS/RAF/MAPK | mRNA processing; mRNA transport; RNA splicing;  positive regulation of gene expression | "**The tumor suppressing effects of QKI-5 in prostate cancer: a novel diagnostic and prognostic protein.**" Y Zhao et al. (2014), Cancer Biol. Ther.,15, 108; "**A large-scale analysis of alternative splicing reveals a key role of QKI in lung cancer.**" FJ de Miguel et al. (2016), Mol. Oncol., 10, 1437. |
| UTRN | Utrophin | Apoptosis | Positive regulation of cell-matrix adhesion; muscle  contraction; muscle organ development; neuromuscular  junction development | "**UTRN on chromosome 6q24 is mutated in multiple tumors.**" Y li et al (2007), Oncogene, 26, 6220; **"Inhibition of Human Glioma Cell Proliferation Caused by Knockdown of Utrophin Using a Lentivirus-Mediated System.**" SH Shen et al (2016), Cancer Biother Radiopharm., 31, 133. |

**Supplementary table 6**

| **Cellular process** | **Cell Survival** | | | | | | | **Cell Fate** | | | | | **Prostate Cancer** | | **DNA Repair** | | | |
| --- | --- | --- | --- | --- | --- | --- | --- | --- | --- | --- | --- | --- | --- | --- | --- | --- | --- | --- |
| **KEGG Pathways** | **Apoptosis** | **Phagosome** | **MAPK** | **RAS** | **PI3K-AKT** | **TGF** | **JAK/STAT** | **Transcriptional mysregulation in cancer** | **Splicesome** | **NOTCH** | **Methabolic pathway** | **Cell adhesion** | **Endocrine resistance** | **Prostate cancer** | **Homologous recombination** | **Base excision repair** | **Nucleotide excision repair** | **Mismatch repair** |
| **DEGs AND DEPs** | CFLAR, SPTAN1, LMNA, LMNB2, CAPN1, CTSK, DAB2IP, PTPN13, TUBA4A, BAD, JUN, NRAS, BAX, HTRA2, TUBA1A, TUBA1C, ITPR1, CTSD, CTSH, MAP2K2 | HLAB, RAB5C, SFTPA2, EEA1, ATP6V0A2, ITGA2, ATP6V1D, TP6AP1, TUBA4A, HLADMA, THBS1, DYNC1H1, STX12, SEC61A1, LAMP1, LAMP2, ATP6V1B2, ATP6V1C1, STX18, ATP6V0A1, RAC1,TAP1, TUBA1A, RAB7A, **CANX** | RRAS, BRAF, LAMTOR3 RPS6KA3, RPS6KA6, TGFBR1, HSPA1A, MAPKAPK2, NRAS, ECSIT, JUN, DUSP3, HSPA8, RRAS2, PPM1A, PRKACB, GNG12, RAP1A, EGF, EGFR, GRB2, MAP2K2, MAPT, RAC1, PAK2, TAOK1 | PLA2G16, EGFR, GRB2, GNB2, GNG5, RHOA, PAK2, PRKACB, GNG12, MAP2K2, PTPN11, RAB5C, RAC1, RRAS, SHC1, CALM2, GNB5, RRAS2, IGF1R, RALA, EGF, RAP1A | IFNAR1, LAMC1, GNG5, PRKAA1, DDIT4, PPP2CA, HSP90AA1, GSK3B, CDKN1A, FOXO3, YWHAG, EFNA5, NRAS, JAK3, ITGA2, PKN1, THEM4, BRCA1, BAD, CREB1, IGF1R, THBS1, GNB5, EGF, EGFR, GRB2, MAP2K2, RAC1, LAMA3, LAMB2, GNB2, GNG12, RPS6, PPP2R2A, CDC37, YWHAE, GYS1, FN1, LPAR3 | AMH, PPP2CA, TGFBR1, CUL1, BMPR1B, TFDP1, THBS1, RHOA | CDKN1A, GRB2, IFNAR1, IL10RB, PTPN11, FHL1 | CDKN1A, FUS, H3F3B, HOXA10, IGFBP3, JUP, MLLT3, PRCC, SPINT1, SSX1, SSX2, SS18, ZBTB16, SLC45A3, **ETV1**, IGF1R, TMPRSS2, SIN3A, JMJD1C | U2SURP, HSPA1A, SF3B6, SRSF1, DDX23, BCAS2, PPIH, PRPF8, SNRNP27, U2AF2, SNRNP200, HSPA8, NCBP1, PPIL1, PLRG1, RBM25, TRA2B, SNRPC, BUD31, PQBP1, SMNDC1, PPIE, CHERP, TXNL4A, LSM6, DDX42, CCDC12, LSM3, PRPF19, HNRNPM, CDC40, LSM2, SRSF2, SRSF4, SNRPB, SNRPE, U2AF1, EFTUD2, CDC5L | JAG1, NOTCH1, APH1A, CTBP2, NCSTN | ACOT8, PIGK, NAMPT, AKR1A1, CDS1, AGPAT2, ATP5L, PMVK, AHCYL1, DHRS4, PLA2G16, MAN1B1, HOGA1, CYP2U1, AFMID, MBOAT2, COX4I1, COX15, CPS1, CYP51A1, DAD1, DHCR7, DNMT1, DTYMK, ENO1, AK1, AK4, ACSL1, ACSL3, FAH, FASN, FECH, ALDOA, PLCB1, AHCYL2, MLYCD, FOLH1, AMACR, LCLAT1, HIBCH, GCDH, GCSH, GLS2, GLDC, MCAT, GMDS, GOT1, GUK1, ACAA1, HADH, HEXB, ACACA, MMAB, IDH1, IDH3B, IDI1, ACADS, ACADSB, IMPA1, IMPDH2, INPP5A, ACADVL, ACAT1, LAMA3, LSS, LTA4H, MAN2A1, MDH1, ME1, NDUFA2, ACLY, NDUFA10, NDUFB8, NDUFS6, NDUFV3, OAT, ACO2, PAFAH1B1, RRM2B, ATP5B, PCCA, PCCB, COQ6, LAP3, ATP5F1, PDHA1, PDHB, PFKP, PGK1, ATP6V1B2, ATP6V1C1, PIGF, PIK3C2A, PI4KA, PLCB4, ATP6V0A1, PMM2, ATP5O, POLE3, NANS, POLR2E, POLR2G, PON2, AUH, PPT1, CNDP2, CHPT1, PNPLA2, PSPH, PYGB, QARS, QDPR, BCKDHA, BCKDHB, ACSM3, SAT1, MSMO1, SRR, ACOT1, BLVRA, SMS, SORD, SUOX, BTD, TDO2, UGDH, UGT2B15, UQCRC2, ALDH5A1, ALG9, UXS1, PTDSS2, ACOX3, POLR1B, NTPCR, ALDH4A1, CBR1, SUCLG2, SUCLA2, DPM1, NFS1, H6PD, GYS1, NDUFA6, PPCDC, FLAD1, PIGL, NDUFA11, NDUFS3, NDUFV1, NDUFA12, CEL, CERS1, ADAM2, COMT, DGKB, ALDH1A3, IVD, MPI, MTR, AADAT, CHPF2, DEGS1, CKB, AHCY, ALDH1B1, **ALDH3A2**, ALDOC, GANAB, GLB1, ACADM, MAOA, MUT, NDUFA3, NDUFA4, NDUFB3, NDUFB9, NDUFS1, NDUFS2, NDUFS8, ATP5I, PGD, POLR1E, BLVRB, TM7SF2, UQCRFS1, TUSC3, GRHPR, POLR1C, GCLC, GPT2, ASAH1 | HLA-DMA, CLDN3, ALCAM, GLG1, CD276, CDH1, **NCAM2** | CDKN1A, JAG1, NOTCH1, SHC1, BRAF, JUN, NRAS, BAD, IGF1R, EGFR, ERBB2, GRB2, PRKACB, MAP2K2, BAX | IGF1R, CDKN1A, GSK3B, CTNNB1, LEF1, BRAF, HSP90AA1, BAD, CREB1, NRAS, EGF, EGFR, ERBB2, GRB2, MAP2K2, KLK3 | RBBP8, XRCC2, RAD51, BARD1, BRCA1, NBN, RPA2 | XRCC1, LIG3, POLE3 | RFC2, CUL4B, POLE3, RPA2, ERCC3 | RFC2, RPA2 |
| **Cellular process** | **Cell Survival** | | | | | | | **Cell Fate** | | | | | **Prostate Cancer** | | **DNA Repair** | | | |
| **KEGG Pathways** | **Apoptosis** | **Phagosome** | **MAPK** | **RAS** | **PI3K-AKT** | **TGF** | **JAK/STAT** | **Transcriptional mysregulation in cancer** | **Splicesome** | **NOTCH** | **Methabolic pathway** | **Cell adhesion** | **Endocrine resistance** | **Prostate cancer** | **Homologous recombination** | **Base excision repair** | **Nucleotide excision repair** | **Mismatch repair** |
| **Proteins associated with CRPC phenotype** | **UTRN** | **CANX** | **QKI, p38MAPK, EPHA3** | **QKI, RKIP** | **AKT** | **ADAMTS1** | **EHF** | **ETV1, EHF** | **hnRNP U** | **ASPH** | **ALDH3A2** | **NCAM2, QKI** | **ADAMTS1, AKT, p38MAPK, PARP1, RKIP** | **ALDH3A2, EPHA3, ETV1, EHF, NCAM2, QKI** |  | **PARP1** |  |  |
| **NUMBER** | **21** | **25** | **29** | **24** | **40** | **9** | **7** | **20** | **40** | **6** | **192** | **8** | **20** | **22** | **7** | **4** | **5** | **2** |
